# Efficacy and safety of IBI351 (fulzerasib) monotherapy in KRAS^G12C^ inhibitor-naïve Chinese patients with *KRAS*^G12C^-mutated metastatic colorectal cancer: a pooled analysis from phase I part of two studies

**DOI:** 10.1038/s41392-025-02315-7

**Published:** 2025-07-25

**Authors:** Ying Yuan, Yanhong Deng, Yongdong Jin, Zengqing Guo, Yueyin Pan, Cunji Wang, Zhiwu Wang, Yi Hu, Dong Hua, Xiangjiao Meng, Zhiye Zhang, Mingfang Zhao, Xiaorong Dong, Dingzhi Huang, Xiaoyan Li, Lian Liu, Meili Sun, Huijuan Wang, Xiuwen Wang, Nong Yang, Mingjun Zhang, Sheng Hu, Dongde Wu, Jingjing Huang, Sujie Zhang, Mengna Huang, Kefeng Ding

**Affiliations:** 1 Department of Medical Oncology, Key Laboratory of Cancer Prevention and Intervention, Ministry of Education, Second Affiliated Hospital of Zhejiang University School of Medicine, Hangzhou, Zhejiang China; 2 Department of Medical Oncology, The Sixth Affiliated Hospital of Sun Yat-sen University, Guangzhou, China; 3 Department of Medical Oncology, Sichuan Cancer Hospital, Chengdu, China; 4 Department of Special Demand Ward/Department of Clinical Nutrition, Fujian Cancer Hospital, Fuzhou, China; 5 Department of Tumor Chemotherapy, Anhui Provincial Hospital, Hefei, China; 6 Department of Oncology, Hunan Traditional Chinese Medical College No.1 Affiliated Hospital, Zhuzhou, China; 7 The Second Department of Radiochemotherapy, Tangshan People’s Hospital, Tangshan, China; 8 Department of Medical Oncology, Chinese PLA General Hospital, Beijing, China; 9 Department of Oncology, Wuxi People’s Hospital, Wuxi, China; 10 Thoracic Radiation Oncology Ward IV, Shandong Provincial Cancer Hospital, Jinan, China; 11 Department of Medical Oncology, The First Affiliated Hospital of Henan University of Science and Technology, Luoyang, China; 12 Department of Medical Oncology, The First Hospital of China Medical University, Shenyang, China; 13 Department of Thoracic Oncology, Union Hospital Affiliated to Tongji Medical College of Huazhong University of Science and Technology, Wuhan, China; 14 Department of Pulmonary Oncology, Tianjin Medical University Cancer Institute and Hospital, Tianjin, China; 15 Department of Medical Oncology, Beijing Tiantan Hospital of Capital Medical University, Beijing, China; 16 Department of Medical Oncology, Qilu Hospital of Shandong University, Jinan, China; 17 Department of Medical Oncology, Central Hospital Affiliated to Shandong First Medical University, Jinan, China; 18 Department of Medical Oncology, The Affiliated Cancer Hospital of Zhengzhou University/Henan Cancer Hospital, Zhengzhou, China; 19 Department of Medical Oncology, Qilu Hospital of Shandong University (Qingdao), Qingdao, China; 20 Department of Pulmonary Gastroenterology, The Second People’s Hospital of Hunan Province, Changsha, China; 21 Department of Oncology, The Second Affiliated Hospital of Anhui Medical University, Hefei, China; 22 Hepatopancreatobiliary Surgery Department, Hubei Cancer Hospital, Wuhan, China; 23 Innovent Biologics Co., Ltd., Suzhou, China; 24 Department of Colorectal Surgery and Oncology, Center for Medical Research and Innovation in Digestive System Tumors, Ministry of Education, Second Affiliated Hospital of Zhejiang University School of Medicine, Hangzhou, Zhejiang China

**Keywords:** Gastrointestinal cancer, Drug development

## Abstract

IBI351 (also known as fulzerasib or GFH925), an irreversible covalent inhibitor of KRAS^G12C^, has demonstrated promising anti-tumour activity in patients with solid tumours. In this study, data were pooled from the phase I part of two clinical studies (NCT05005234 and NCT05497336), aiming to evaluate the efficacy and safety of IBI351 monotherapy in KRAS^G12C^ inhibitor-naïve Chinese patients with *KRAS*^G12C^-mutated metastatic colorectal cancer (CRC). The objective response rate (ORR) was the primary endpoint. Secondary endpoints included disease control rate (DCR), progression-free survival (PFS), and overall survival (OS). As of December 13, 2023, 56 patients treated with IBI351 monotherapy were included. The median duration of treatment was 7.7 months (range: 0.3–16.7). The confirmed ORR was 44.6% (95% CI: 31.3–58.5), with a DCR of 87.5% (95% CI: 75.9–94.8). With a median follow-up of 13.8 months, the median PFS was 8.1 months (95% CI: 5.5–13.8). The median OS was 17.0 months (95% CI: 12.6–not reached). Treatment-related adverse events (TRAEs) occurred in 53 patients (94.6%), with grade 3 TRAEs in 14 patients (25.0%). No grade 4 or 5 TRAEs were observed. The most common grade 3 TRAEs were anaemia (*n* = 4, 7.1%) and gamma-glutamyltransferase increased (*n* = 3, 5.4%). TRAEs led to dose interruption in 12 patients (21.4%) and dose reduction in six patients (10.7%). No TRAEs resulted in treatment discontinuation. IBI351 demonstrated encouraging clinical efficacy and a manageable safety profile in KRAS^G12C^ inhibitor-naïve Chinese patients with *KRAS*^G12C^-mutated metastatic CRC.

## Introduction

Colorectal cancer (CRC) ranks as the second most commonly diagnosed cancer and the fourth leading cause of cancer-related mortality in China.^1^ In 2022, it accounted for approximately 517,000 newly diagnosed cases and 240,000 deaths.^2^ Over the past two decades, China has witnessed a significant increase in the incidence and mortality rates of CRC among both females and males.^1^

CRC is a molecularly heterogeneous disease, involving a number of genes that contribute to the transformation from benign neoplasia to invasive carcinoma, and ultimately to metastatic CRC.^3^ Among these genes, the Kirsten rat sarcoma viral oncogene homologue (KRAS), a GTPase and member of RAS family, plays a pivotal role in oncogenesis, driving tumour development in ~50% of CRC patients.^4^ *KRAS* mutations occur relatively early in tumourigenesis and contribute to the progression from adenoma to carcinoma. Specifically, the glycine-to-cysteine mutation at position 12 (G12C) of KRAS impairs guanosine triphosphate (GTP) hydrolysis, thereby locking KRAS in an active, GTP-bound state. Hyperactivation of KRAS^G12C^ drives oncogenic signalling across multiple downstream pathways, directly promoting tumour cell survival, proliferation, and metastasis.^5^ *KRAS*^G12C^ mutations occur in about 3%–4% of CRC patients and are associated with poor clinical outcomes.^6^

According to National Comprehensive Cancer Network (NCCN) and Chinese Society of Clinical Oncology (CSCO) guidelines, patients with metastatic CRC are recommended to undergo tests of microsatellite instability (MSI), *KRAS*/neuroblastoma ras viral oncogene homologue (*NRAS*), B-Raf proto-oncogene serine/threonine kinase (*BRAF*), epidermal growth factor receptor 2 (HER-2), neurotrophic tyrosine receptor kinase (*NTRK*), and polymerase epsilon/delta (*POLE/POLD1*), among others.^7,8^ Immune checkpoint inhibitors have been established as first-line palliative treatment in deficient mismatch repair (dMMR)/microsatellite instability-high (MSI-H) metastatic CRC. For proficient mismatch repair (pMMR)/microsatellite stability (MSS) metastatic CRC, standard first-line therapy includes fluoropyrimidine-based chemotherapy (with oxaliplatin or irinotecan) or in combination with biological antibodies (anti-vascular endothelial growth factor [VEGF] antibody or anti-epidermal growth factor receptor [EGFR] antibody).^7,8^ Targeted therapies for patients carrying actionable molecular variations, such as *BRAF*^V600E^ mutation, *KRAS*^G12C^ mutation, and HER-2 overexpression or amplification, are currently utilised in second-line palliative setting and beyond. Notably, targeted therapies against these specific variants are currently being evaluated in phase III studies as potential first-line treatment options.^9–11^ For example, the phase-III MOUNTAINEER-03 trial compared the efficacy of tucatinib and trastuzumab plus mFOLFOX6 with standard regimens (mFOLFOX6, mFOLFOX6 plus bevacizumab, or mFOLFOX6 plus cetuximab).^9^ For metastatic CRC patients with *BRAF*^V600E^ mutations, the ongoing phase III BREAKWATER study compared the efficacy between encorafenib plus cetuximab (chemotherapy-free), encorafenib plus cetuximab with chemotherapy (mFOLFOX6 or FOLFIRI), and standard first-line regimens.^10^ Traditional standard therapeutic approaches for *KRAS*^G12C^-mutated unresectable or metastatic CRC consist of chemotherapy alone or in combination with anti-VEGF antibodies. However, the clinical benefits observed have been modest at best,^12^ necessitating the development of novel therapies.

*KRAS* mutations were historically considered “undruggable”.^13^ The discovery of the cysteine pocket enabled the development of selective KRAS^G12C^ inhibitors.^14,15^ Patients with CRC harbouring *KRAS*^G12C^ mutations often have worse survival outcomes. Recent progress has led to the successful development of targeted therapies for KRAS^G12C^, offering a new therapeutic approach for this patient population. Notably, two KRAS^G12C^ inhibitors─sotorasib and adagrasib─have been approved in the U.S. for the treatment of adults with previously treated *KRAS*^G12C^-mutated CRC. As of August 21, 2024, fulzerasib (also known as IBI351 or GFH925), as the first KRAS^G12C^ inhibitor in China, has been approved for the treatment of adults with *KRAS*^G12C^-mutated advanced non-small cell lung cancer (NSCLC) who have received at least one prior systemic therapy. Despite these therapeutic advancements, KRAS^G12C^ inhibitors remain unapproved for CRC treatment in China.

IBI351, an oral small-molecule covalent inhibitor of KRAS^G12C^, has demonstrated promising efficacy and durable response in patients with *KRAS*^G12C^-mutated advanced solid tumours (NSCLC comprising 94.3% of cases [166/176]).^16^ However, its efficacy and safety profile in *KRAS*^G12C^-mutated metastatic CRC patients are underexplored. To address this gap, we conducted a pooled study from two phase I trials to evaluate the efficacy and safety of IBI351 monotherapy in KRAS^G12C^ inhibitor-naïve Chinese patients with *KRAS*^G12C^-mutated metastatic colorectal cancer.

## Results

### Baseline demographics

**Fig. 1:**
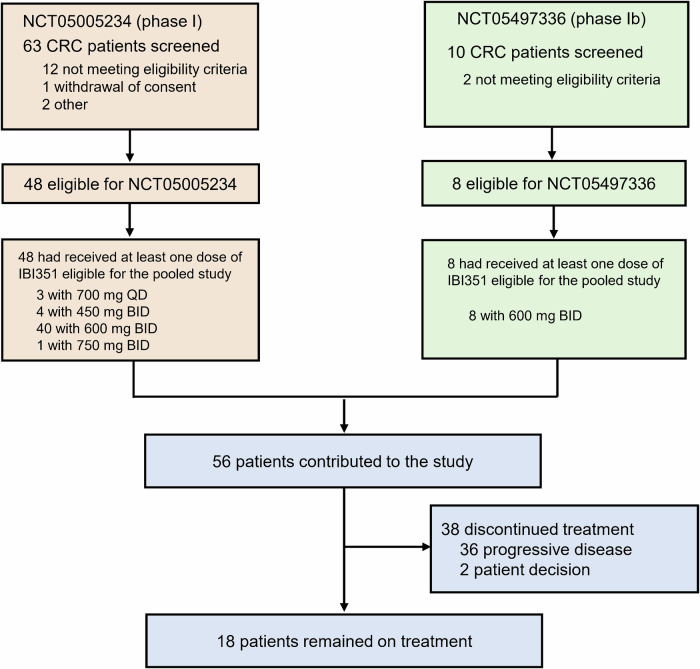
Trial profile. Two trials contributed patients to this pooled analysis QD, once daily; BID, twice daily. For phase I of NCT05005234, a total of 63 CRC patients were screened for eligibility. Following the exclusion of 15 patients (12 failing to meet eligibility criteria, 1 withdrawing informed consent, and 2 excluded for other reasons), 48 patients were enrolled and received at least one dose of IBI351. In the phase Ib of NCT05497336, 10 CRC patients were screened, with 2 excluded due to ineligibility criteria, resulting in 8 patients included. These 8 patients received at least one dose of IBI351. Our pooled analysis included patients with metastatic CRC who received at least one dose of IBI351 across both trials. This yielded a combined cohort of 48 patients from phase I of NCT05005234 and 8 from phase Ib of NCT05497336

In the pooled analysis, a total of 56 KRAS^G12C^ inhibitor-naïve patients with *KRAS*^G12C^-mutated metastatic CRC (overall population) treated with IBI351 monotherapy were included (Fig. 1). More than 60% of patients failed at least 2 lines of prior treatment (Table 1). IBI351 was administered orally at doses of 700 mg once daily (QD, *n* = 3), or 450 mg (*n* = 4)/600 mg (*n* = 48)/750 mg (*n* = 1) twice daily (BID). The median treatment duration was 7.7 months (range: 0.3–16.7). A total of 38 patients (67.9%) experienced treatment discontinuation due to progressive disease (PD) (*n* = 36, 64.3%) and patient decision (*n* = 2, 3.6%) (Fig. 1). In the overall population, the median age was 58 years (range: 32–76). 60.7% of patients were males, 73.2% had an Eastern Cooperative Oncology Group Performance Status (ECOG PS) score of 1, 78.6% had lung metastases, and 60.7% had liver metastases. Most patients were non-smokers (66.1%). Baseline characteristics are summarised in Table 1.

**Table 1:** Baseline characteristics

Characteristics	Overall (*N* = 56)
Age	
Median (range), years	58 (32–76)
Age stratification, *n* (%)	
$<$60 years	32 (57.1)
$\ge$60 years	24 (42.9)
Gender, *n* (%)	
Male	34 (60.7)
Female	22 (39.3)
TNM stage, *n* (%)	
Stage IV	56 (100)
ECOG PS, *n* (%)	
0	15 (26.8)
1	41 (73.2)
Metastatic disease, *n* (%)	
Lung	44 (78.6)
Liver	34 (60.7)
Bone	12 (21.4)
Brain	2 (3.6)
Adrenal	2 (3.6)
Smoking history, *n* (%)	
Never	37 (66.1)
Current	4 (7.1)
Former	14 (25.0)
Unknown	1 (1.8)
Prior lines of treatment, *n* (%)	
0	1 (1.8)
1	21 (37.5)
2	15 (26.8)
≥3	19 (33.9)
Prior treatment, *n* (%)	
5-Fluorouracil	55 (98.2)
Oxaliplatin	48 (85.7)
Irinotecan	41 (73.2)

*ECGO PS* Eastern Cooperative Oncology Group Performance Status, *TNM* tumour, node, and metastasis

### Anti-tumour activity

**Fig. 2:**
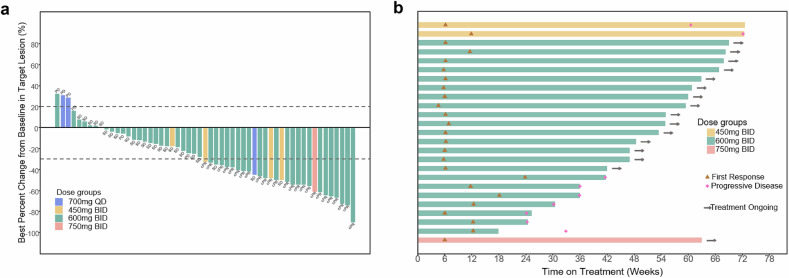
Response to IBI351 monotherapy in *KRAS*^G12C^-mutated metastatic CRC patients **a** Waterfall plot of the best percentage change in the sum of diameters of target lesion(s) from baseline in patients who had post-baseline tumour assessments (*n* = 55). **b** Swimmer plot of treatment duration in patients with confirmed objective response (*n* = 25). CRC colorectal cancer, SD stable disease, PD progressive disease, cPR confirmed partial response, QD once daily, BID twice daily

**Fig. 3:**
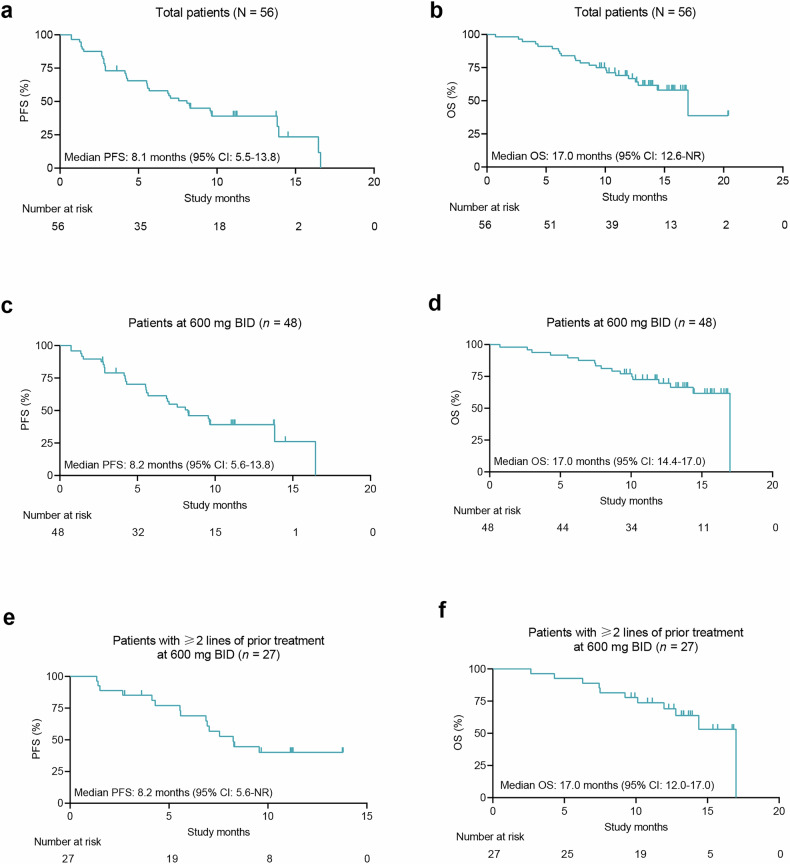
Kaplan–Meier plots of survival curves in *KRAS*^G12C^-mutated metastatic CRC patients treated with IBI351 monotherapy. Progression-free survival (**a**) and overall survival (**b**) in the overall population. Progression-free survival (**c**) and overall survival (**d**) in patients treated with IBI351 at RP2D (600 mg BID). Progression-free survival (**e**) and overall survival (**f**) in patients who had ≥2 lines of prior treatment treated with IBI351 at RP2D. BID twice daily, RP2D recommended phase II dose, CRC colorectal cancer, CI confidence interval, PFS progression-free survival, OS overall survival, NR not reached. Bars indicate censored data

Of the 56 patients, 55 patients experienced post-baseline tumour assessments. Target lesion shrinkage of any magnitude was observed in 46 patients (Fig. 2a). No patients achieved a confirmed best overall response of complete response (CR), 25 had a confirmed partial response (PR), 24 had stable disease (SD), and six had PD (Table 2, Fig. 2b). The confirmed objective response rate (ORR) was 44.6% (95% confidence interval [CI]: 31.3–58.5) with a disease control rate (DCR) of 87.5% (95% CI: 75.9–94.8). The median time to response (TTR) was 1.4 months (range: 1.1–5.5, Table 2). Most patients who had a confirmed PR (64.0%, 16/25) remained on treatment at the data cutoff date (Fig. 2b). The median duration of response (DoR) was 12.6 months (95% CI: 12.6–13.9) and PD or death occurred in nine of 25 patients who had a confirmed PR. With a median follow-up of 13.8 months (95% CI: 11.1–13.8), the median progression-free survival (PFS) was 8.1 months (95% CI: 5.5–13.8) (Table 2, Fig. 3a). The median overall survival (OS) was 17.0 months (95% CI: 12.6–not reached [NR]) (Table 2, Fig. 3b).

**Table 2:** Overall summary of efficacy

Efficacy	600 mg BID (*n* = 48)	600 mg BID with $\ge$2 lines of prior treatment (*n* = 27)	Overall (*N* = 56)
Best of response, *n* (%)			
PR	22 (45.8)	17 (63.0)	25 (44.6)
SD	21 (43.8)	7 (25.9)	24 (42.9)
PD	4 (8.3)	3 (11.1)	6 (10.7)
Not evaluated^a^	1 (2.1)	0 (0.0)	1 (1.8)
Confirmed ORR, *n* (%)	22 (45.8)	17 (63.0)	25 (44.6)
95% CI	31.4–60.8	42.4–80.6	31.3–58.5
DCR, *n* (%)	43 (89.6)	24 (88.9)	49 (87.5)
95% CI	77.3–96.5	70.8–97.6	75.9–94.8
TTR			
Median (range), months	1.4 (1.1–5.5)	1.5 (1.1–5.5)	1.4 (1.1–5.5)
DoR^b^			
Median (95% CI), months	NR (4.8–NR)	NR (4.1–NR)	12.6 (12.6–13.9)
12-month DoR rate (95% CI), %	68.2 (44.6–83.4)	58.8 (32.5–77.8)	72.0 (50.1–85.5)
PFS			
Median (95% CI), months	8.2 (5.6–13.8)	8.2 (5.6–NR)	8.1 (5.5–13.8)
12-month PFS rate (95% CI), %	39.2 (25.1–52.9)	40.2 (21.2–58.4)	39.1 (26.1–51.8)
OS			
Median (95% CI), months	17.0 (14.4–17.0)	17.0 (12.0–17.0)	17.0 (12.6–NR)
12-month OS rate (95% CI), %	69.6 (54.0–80.9)	69.1 (47.3–83.3)	66.7 (52.3–77.6)

*BID* twice daily, *CR* complete response, *PR* partial response, *SD* stable response, *PD* progressive disease, *ORR* objective response rate, *CI* confidence interval, *DCR* disease control rate, *TTR* time to response, *DoR* duration of response, *PFS* progression-free survival, *OS* overall survival, *NR* not reached

^a^indicates the absence of post-baseline tumour response assessment

^b^DoR was assessed in patients who had a confirmed PR

Among the 48 patients who received IBI351 at the recommended phase II dose (RP2D) of 600 mg BID, the confirmed ORR was 45.8% (95% CI: 31.4–60.8), with a DCR of 89.6% (95% CI: 77.3–96.5). The median TTR was 1.4 months (range: 1.1–5.5). The median DoR was not reached, with PD or death observed in seven of 22 patients who had a confirmed PR. With a median follow-up of 11.3 months (95% CI: 11.1–13.8), the median PFS was 8.2 months (95% CI: 5.6–13.8) (Table 2, Fig. 3c). The median OS was 17.0 months (95% CI: 14.4–17.0) (Table 2, Fig. 3d).

A total of 34 patients had received ≥2 lines of prior treatment, including three treated with IBI351 at 700 mg QD, four at 450 mg BID, and 27 at 600 mg BID. Among those patients who had received ≥2 lines of prior treatment and were treated with IBI351 at the RP2D of 600 mg BID (*n* = 27), the confirmed ORR reached 63.0% (95% CI: 42.4–80.6) with a DCR of 88.9% (95% CI: 70.8–97.6). With a median follow-up of 11.2 months (95% CI: 9.7–13.8), the median PFS was 8.2 months (95% CI: 5.6–NR) (Table 2, Fig. 3e). The median OS was 17.0 months (95% CI: 12.0–17.0) (Table 2, Fig. 3f).

### Safety

No new safety signals were reported in this pooled analysis in comparison with previous reports.^16,17^ Among the 56 patients across all dose levels, treatment-emergent adverse events of any grade were reported in 55 (98.2%) patients. Treatment-related adverse events (TRAEs) occurred in 53 (94.6%) patients. The most common TRAEs included anaemia (*n* = 28, 50.0%), white blood cell count decreased (*n* = 18, 32.1%), blood bilirubin increased (*n* = 17, 30.4%), and pruritus (*n* = 15, 26.8%). Grade 3 TRAEs were reported in 14 (25.0%) patients (Table 3), with no grade 4 TRAEs or deaths observed. The most common grade 3 TRAEs were anaemia (*n* = 4, 7.1%) and gamma-glutamyltransferase increased (*n* = 3, 5.4%) (Table 3). Three patients (5.4%) underwent treatment-related serious adverse events, all of whom experienced grade 3 anaemia. Dose interruption and reduction due to TRAEs occurred in 12 (21.4%, Table 3, Supplementary Table 1) and six patients (10.7%, Table 3, Supplementary Table 2), respectively. Among the six patients who required dose reduction, four had one dose level reduced, one had two dose levels reduced, and another had three dose levels reduced (Supplementary Table 2). Consequently, the median number of dose levels reduced was one. No TRAEs led to treatment discontinuation (Table 3). A similar safety profile was observed in patients treated with IBI351 at 600 mg BID (Table 3). The comprehensive list of TRAEs is provided in Supplementary Table 3.

**Table 3:** Summary of adverse events with IBI351 monotherapy in patients with *KRAS*^G12C^-mutated unresectable or metastatic CRC

AE	600 mg BID (*n* = 48)		Overall (*N* = 56)	
	Any grade	Grade 3^a^	Any grade	Grade 3^a^
TEAEs, *n* (%)	47 (97.9)	20 (41.7)	55 (98.2)	23 (41.1)
TRAEs, *n* (%)	45 (93.8)	12 (25.0)	53 (94.6)	14 (25.0)
TRSAE, *n* (%)	3 (6.3)	3 (6.3)	3 (5.4)	3 (5.4)
TRAEs occurring in ≥10% of patients or grade ≥3 in any group, *n* (%)				
Anaemia	24 (50.0)	4 (8.3)	28 (50.0)	4 (7.1)
White blood cell count decreased	17 (35.4)	1 (2.1)	18 (32.1)	1 (1.8)
Blood bilirubin increased	14 (29.2)	0 (0.0)	17 (30.4)	0 (0.0)
Pruritus	15 (31.3)	0 (0.0)	15 (26.8)	0 (0.0)
Neutrophil count decreased	13 (27.1)	1 (2.1)	13 (23.2)	1 (1.8)
Aspartate aminotransferase increased	7 (14.6)	0 (0.0)	12 (21.4)	0 (0.0)
Protein urine present	10 (20.8)	0 (0.0)	12 (21.4)	0 (0.0)
Alanine aminotransferase increased	6 (12.5)	0 (0.0)	11 (19.6)	0 (0.0)
Gamma-glutamyltransferase increased	6 (12.5)	1 (2.1)	10 (17.9)	3 (5.4)
Asthenia	8 (16.7)	1 (2.1)	10 (17.9)	1 (1.8)
Hypoalbuminaemia	9 (18.8)	0 (0.0)	9 (16.1)	0 (0.0)
Hypoaesthesia	8 (16.7)	0 (0.0)	9 (16.1)	0 (0.0)
Oedema peripheral	8 (16.7)	0 (0.0)	9 (16.1)	0 (0.0)
Blood alkaline phosphatase increased	4 (8.3)	0 (0.0)	7 (12.5)	2 (3.6)
Bilirubin conjugated increased	5 (10.4)	0 (0.0)	6 (10.7)	0 (0.0)
Blood lactate dehydrogenase increased	6 (12.5)	0 (0.0)	6 (10.7)	0 (0.0)
Platelet count decreased	6 (12.5)	1 (2.1)	6 (10.7)	1 (1.8)
Hyperphosphataemia	5 (10.4)	0 (0.0)	6 (10.7)	0 (0.0)
Rash	4 (8.3)	0 (0.0)	6 (10.7)	0 (0.0)
Electrocardiogram QT prolonged	4 (8.3)	1 (2.1)	5 (8.9)	1 (1.8)
Lymphocyte count decreased	4 (8.3)	2 (4.2)	4 (7.1)	2 (3.6)
Peripheral sensory neuropathy	2 (4.2)	2 (4.2)	3 (5.4)	2 (3.6)
TRAEs leading to dose interruption	11 (22.9)		12 (21.4)	
TRAEs leading to dose reduction	6 (12.5)		6 (10.7)	
TRAEs leading to treatment discontinuation	0 (0.0)		0 (0.0)	
TRAEs leading to death	0 (0.0)		0 (0.0)	

*AE* adverse event, *BID* twice daily, *CRC* colorectal cancer, *TEAE* treatment-emergent adverse event, *TRAE* treatment-related adverse event, *TRSAE* treatment-related serious adverse event

^a^No grade 4 or 5 TRAEs were reported

## Discussion

KRAS was historically regarded as an “undruggable” target until the development of KRAS^G12C^ allele-specific inhibitors. The discovery of the switch II pocket within the KRAS protein has rapidly accelerated the development of KRAS^G12C^ inhibitors, such as sotorasib, adagrasib, divarasib, garsorasib (D-1553), and D3S-001.^18^ IBI351, as a novel, irreversible covalent KRAS^G12C^ inhibitor, has demonstrated promising anti-tumour activity in patients with advanced solid tumours.^16^ This report, based on pooled data from the phase I part of two studies, aims to evaluate the efficacy and safety of IBI351 monotherapy in KRAS^G12C^ inhibitor-naïve Chinese patients with *KRAS*^G12C^-mutated metastatic CRC.

The primary objective of this pooled study was to assess ORR. IBI351 demonstrated encouraging anti-tumour activity among *KRAS*^G12C^-mutated metastatic CRC patients. In the overall population, the investigator-assessed confirmed ORR was 44.6% (95% CI: 31.3–58.5). It was 7.1% (95% CI: 1.5–19.5) with sotorasib (180/360/720/960 mg QD) in the phase I CodeBreaK100 trial^19^ and 29.1% (95% CI: 17.6–42.9) with divarasib (50/100/200/400 mg QD) in a phase I study^20^ for the treatment of *KRAS*^G12C^-mutated metastatic CRC (Supplementary Table 4). Recently, D3S-001 (50–900 mg QD) has demonstrated impressive efficacy in a phase I/II study,^21^ with a confirmed ORR of 77.8% (95% CI: 40.0–97.2) and a DCR of 88.9% for the treatment of KRAS^G12C^ inhibitor-naïve advanced/metastatic CRC patients carrying *KRAS*^G12C^ mutations (*n* = 9) (Supplementary Table 4). In the subgroup of patients treated with IBI351 at RP2D (600 mg BID), the investigator-assessed confirmed ORR reached 45.8% (95% CI: 31.4–60.8), which was 12.9% (95% CI: 5.7–23.9) with sotorasib at 960 mg QD in the phase II CodeBreaK100 study,^22^ 19% (95% CI: 8–33) with adagrasib at 600 mg BID in the phase I/II KRYSTAL-1 study,^23^ 35.9% (95% CI: 21.2–52.8) with divarasib at 400 mg QD in a phase I study,^20^ and 19.2% (95% CI, 6.6–39.4) with garsorasib (600 mg BID) in a phase II study^24^ (Supplementary Table 4). In this study, approximately 34% of enrolled patients had received ≥3 lines of prior treatment, and around 70% had an ECOG PS score of 1. In comparison, studies involving sotorasib, adagrasib, and divarasib reported 61%–73% patients with ≥3 lines of prior treatment and 48%–57% with an ECOG PS score of 1.^20,22,23^ A total of 19 patients who had received ≥3 lines of prior treatment were included in the current study. Post-hoc analyses showed a confirmed ORR of 47.4% with a DCR of 78.9% in this subgroup. Due to the small sample size, further large-cohort clinical trials are needed to comprehensively evaluate IBI351 as a late-line therapy in *KRAS*^G12C^-mutated metastatic CRC patients. As a secondary endpoint, the median PFS reached 8.2 months (95% CI: 5.6–NR) in the subset of patients treated with IBI351 at RP2D, which was 4.0 months (95% CI: 2.8–4.2) with sotorasib at 960 mg QD in the phase II CodeBreaK100 study,^22^ 5.6 months (95% CI: 4.1–8.3) with adagrasib at 600 mg BID in a phase I/II KRYSTAL-1 study,^23^ 6.9 months (95% CI: 5.3–9.1) with divarasib at 400 mg QD in a phase I study,^20^ and 5.5 months (95% CI: 2.9–11.6) with garsorasib at 600 mg BID in a phase II study^24^ (Supplementary Table 4). This study showed a median OS of 17.0 months (95% CI: 14.4–17.0) in patients who received IBI351 at 600 mg BID. Notably, 29 patients remained on treatment at the data cutoff date. The median OS may further extend with a longer follow-up. Longer-term efficacy and safety data will be reported as follow-up continues to fully evaluate the durability of response to IBI351 monotherapy.

In the current clinical practice, treatment options for Chinese patients with *KRAS*^G12C^-mutated metastatic CRC in third-line setting or beyond remain limited. These options include regorafenib, fruquintinib, and trifluridine-tipiracil, either as monotherapy or in combination with bevacizumab. However, the clinical benefits of these therapies are unsatisfactory, with an ORR of 1.0%–6.1%, PFS of 2.0–5.6 months, and OS of 6.0–10.8 months.^25–28^ In contrast, IBI351 at RP2D demonstrated an investigator-assessed confirmed ORR of 63.0% (95% CI: 42.4–80.6), a median PFS of 8.2 months (95% CI: 5.6–NR), and median OS not mature (17.0 months [95% CI: 12.0–17.0]) in patients who received ≥2 lines of prior treatment (*n* = 27). The higher ORR observed in the ≥2 lines subgroup (*n* = 27) compared to those receiving IBI351 at RP2D across prior treatment lines (*n* = 48) might be partly explained by the higher percentages of patients aged <60 years (63.0% vs. 53.1%) and with an ECOG PS score of 0 (33.3% vs. 29.2%) in the ≥2 lines subgroup. Given the relatively small sample size of the study, further prospective studies with larger cohorts are necessary to clarify this point.

Some studies have shown additive effects by combining KRAS^G12C^ inhibitors with an anti-EGFR antibody to prevent reactivation of the RAS-MAPK pathway, which has been identified as a predominant mechanism of resistance to KRAS^G12C^ inhibitors in CRC.^29^ Combined blockade of KRAS^G12C^ and EGFR can overcome this adaptive resistance reported in both in vitro and in vivo studies.^29^ Current evidence suggests consistent improvements in ORR with combination therapies across KRAS^G12C^ inhibitors,^23,30,31^ while PFS and OS data remain less conclusive. Adagrasib combined with cetuximab has shown a more favourable confirmed ORR (46% [95% CI: 28–66] vs. 19% [95% CI: 8–33]) and median PFS (6.9 [95% CI: 5.4–8.1] vs. 5.6 months [95% CI: 4.1–8.3]) over adagrasib monotherapy.^23^ Similar results have also been observed in divarasib plus cetuximab vs. divarasib alone (confirmed ORR: 62.5% [95% CI: 40.6– 81.2] vs. 35.9% [95% CI: 21.2–52.8]; median PFS: 8.1 [95% CI: 5.5–12.3] vs. 6.9 months [95% CI: 5.3–9.1]).^20,30^ The phase III CodeBreaK300 study in patients with chemorefractory *KRAS*^G12C^-mutated metastatic CRC, sotorasib 960 mg QD combined with panitumumab, has demonstrated a longer PFS (5.6 vs. 2.0 months) and higher ORR (26.4% vs. 0%) than standard treatment with trifluridine-tipiracil or regorafenib.^31^ These findings highlight the ORR improvements with the combination of KRAS^G12C^ inhibitor and anti-EGFR antibody in patients with *KRAS*^G12C^-mutated metastatic CRC. Whether combination therapies can improve PFS and/or OS require further investigations.

Recently, a phase Ib study of CodeBreaK101, evaluating the combination of sotorasib, panitumumab, and FOLFIRI, has demonstrated an acceptable safety profile with promising efficacy (confirmed ORR: 75.0% [95% CI: 58.8–87.3]; DCR: 92.5% [95% CI: 76.1–98.4]) in treatment-naïve patients with *KRAS*^G12C^-mutated metastatic CRC^32^; however, PFS and OS data remain immature. These findings suggest the potential of KRAS^G12C^ inhibitors as a first-line treatment option for *KRAS*^G12C^-mutated metastatic CRC patients. Nevertheless, further investigations are needed to determine whether first-line use of KRAS^G12C^ inhibitors can provide survival benefits.

In this work, 94.6% of patients experienced TRAEs, with 25.0% having grade 3. No grade 4/5 TRAEs were reported. IBI351 presented a distinct TRAE profile compared to existing KRAS^G12C^ inhibitors. Sotorasib, adagrasib, divarasib, and garsorasib are associated with a high incidence of treatment-related gastrointestinal (GI) events, such as diarrhoea, nausea, and vomiting. In this study, IBI351 demonstrated a relatively low incidence (7.1%, *n* = 4) of GI toxicity, including two patients with nausea and one each with diarrhoea and constipation (Supplementary Table 3). The incidences of IBI351-related nausea and diarrhoea (3.6% and 1.8%) reported in CRC were comparable to those reported in NSCLC (7.8% nausea and 2.6% diarrhoea)^33^ and solid tumours^16^ (primarily NSCLC, 4.5% for both nausea and diarrhoea). The most common TRAE has been reported as diarrhoea (21.0%–34.0%) for sotorasib,^22,34,35^ diarrhoea (66%)^23^ or nausea (49.2%–76%) for adagrasib,^36,37^ nausea (74%) for divarasib,^20^ and rash (71.4%)^24^ or aspartate aminotransferase (AST) increased (44.3%–53%)^38,39^ for garsorasib, respectively, while it was anaemia (50.0%) for IBI351. AST increased was also frequently observed in IBI351 (21.4%, *n* = 12, Table 3). Dose interruption due to TRAEs occurred in 21.4% of patients, which was comparable to or slightly lower than that reported in CRC patients who received adagrasib (45%)^23^ or divarasib (20%).^30^ In addition, dose reduction due to TRAEs occurred in 10.7% of patients, which was numerically lower than that in adagrasib (39.0%)^23^ or divarasib (14%).^30^ In addition, TRAEs leading to treatment discontinuation were not observed in this study, which was reported in sotorasib with up to 10%,^19,22,34,35^ adagrasib with up to 6.9%,^37,40^ divarasib with 3%,^20^ and garsorasib with up to 7%^24,38^ of patients.

The encouraging efficacy and low incidence of GI events observed with IBI351 might be attributed to its unique chemical structure. Calculated LogP (cLogP), which reflects a compound’s hydrophobicity or lipophilicity, plays a key role in determining aqueous solubility. Molecules with lower cLogP values tend to have higher aqueous solubility. Preclinical findings (data not shown) indicate a lower cLogP (<4.4) of IBI351 compared to sotorasib. This lower cLogP improves metabolic stability, reduces plasma protein binding, and subsequently increases the concentration of the drug at target sites, ultimately enhancing IBI351 efficacy. This may explain its relatively higher ORR in clinical settings. Additionally, IBI351 features a closed piperazine ring, whereas other existing KRAS^G12C^ inhibitors, such as sotorasib, adagrasib, and divarasib, carry an open piperazine ring. The closed piperazine ring enhances the stability of IBI351, making it resistant to cleavage and maintaining chemical stability even under acidic conditions. This structural feature likely contributes to its mild gastrointestinal side effects in clinical use. In contrast, the open piperazine rings in the three aforementioned KRAS^G12C^ inhibitors make them more susceptible to degradation under acidic conditions, potentially explaining their relatively stronger gastrointestinal adverse effects.

In this study, anaemia was the most common TRAE, reported in 28 (50.0%) patients. The incidence was comparable to that reported in *KRAS*^G12C^-mutated advanced NSCLC (44.8%),^33^ and numerically higher than that observed in sotorasib (13.2%)^19^ as well as adagrasib (16%).^23^ To preliminarily investigate the reason behind the high incidence of anaemia, post-hoc analyses were conducted to evaluate the effect of baseline haemoglobin level on the incidence of anaemia. Among those patients who treated with IBI351 at R2PD and had post-baseline haemoglobin assessments (*n* = 47), 34, 12, and one patient(s) had a baseline haemoglobin level of ≥130 g/L (without anaemia), 100–130 g/L (grade 1 anaemia), and 80–100 g/L (grade 2 anaemia), respectively. Of the 34 patients without anaemia at baseline, 25 (73.5%, 25/34) patients experienced significant drops from normal haemoglobin levels during the trial, with grade 1 anaemia in 21 (61.8%, 21/34) patients, grade 2 in three (8.8%, 3/34) patients, and grade 3 in one (2.9%, 1/34) patient. Of the 12 patients with grade 1 anaemia at baseline, six (50.0%, 6/12) patients experienced significant drops in the haemoglobin level, including grade 2 anaemia in four (33.3%) patients and grade 3 in two (16.7%) patients. In addition, the patient with grade 2 anaemia at baseline progressed to grade 3 during the trial. Collectively, among the 47 patients treated with IBI351 at R2PD who had post-baseline haemoglobin assessments, four patients experienced grade 3 anaemia. These included one patient without baseline anaemia, two patients with grade 1 anaemia at baseline, and one patient with grade 2 anaemia at baseline; all received 600 mg BID. Of these four patients, two patients required blood transfusions due to treatment-related anaemia. The first patient, with baseline grade 1 anaemia, progressed to grade 3 anaemia during the treatment period. Following dose reduction (to 450 mg BID) and blood transfusion, the anaemia resolved to grade 1. The second patient, without anaemia at baseline, developed grade 3 anaemia during treatment. After treatment interruption and blood transfusion, the condition resolved to grade 2. No treatment discontinuation due to anaemia occurred. These findings demonstrated that the decrease in haemoglobin levels was associated with the use of IBI351, and the incidence of grade 3 anaemia was affected by the baseline haemoglobin level. Although IBI351 had a higher incidence of any grade anaemia, a comparable incidence of grade 3 anaemia was observed between IBI351 (7.1%) and other existing KRAS^G12C^ inhibitors, such as sotorasib (4.7%),^19^ and adagrasib (9%).^23^ Neutrophil count decreased, as one of the common TRAEs, had a higher incidence (23.2%) than that reported in sotorasib, adagrasib, and divarasib, respectively (all less than 10%).^19,20,22,23^ However, only one patient experienced a grade 3 neutrophil count decrease, which was reversible after administration of human-granulocyte stimulating factor. Overall, IBI351-related anaemia and neutrophil count decreased events were manageable.

Given the low incidence of GI events (nausea, diarrhoea, and vomiting) observed with IBI351, its combination with anti-EGFR antibody and fluoropyrimidine-based chemotherapy (FOLFOX or FOLFIRI) presents a promising treatment approach. However, due to the relatively high incidence of anaemia associated with IBI351, special attention should be paid to potential myelosuppression when it is used in combination with chemotherapy.

Comprehensive gene profiling in tissue and/or blood has the potential to offer additional insights for evaluating personalised treatment response. Due to the limited number of tumour tissue samples obtained at progression, comprehensive gene profiling was not explored in this study. Further studies are warranted to elucidate the mechanisms underlying acquired resistance to IBI351 and to identify predictive biomarkers for survival outcomes in patients receiving IBI351.

There are some limitations associated with this study: enrolment was restricted to Chinese patients; the non-randomised design lacked active controls and blinding; patient-reported outcomes or quality-of-life measures were not assessed. Furthermore, as both source trials are ongoing, this subpopulation may be subject to selection biases. Nevertheless, consistent with preclinical data demonstrating IBI351’s favourable aqueous solubility and acid stability compared to other KRAS^G12C^ inhibitors, IBI351 showed promising preliminary efficacy with low GI toxicity in KRAS^G12C^ inhibitor-naïve Chinese patients with *KRAS*^G12C^-mutated metastatic CRC. Large-scale randomised controlled trials are warranted to definitively establish the efficacy and safety of IBI351 in this population.

In conclusion, IBI351 monotherapy demonstrated encouraging efficacy and a manageable safety profile in KRAS^G12C^ inhibitor-naïve Chinese patients with *KRAS*^G12C^-mutated metastatic CRC. These findings warrant further clinical development of IBI351 monotherapy or combination therapy as a potential treatment option for patients with *KRAS*^G12C^-mutated metastatic CRC. A phase III study is planned to evaluate IBI351 combined with cetuximab in metastatic CRC, with results to be reported in due course.

## Materials and methods

### Study oversight

Two clinical trials (ClinicalTrials.gov identifiers: NCT05005234 and NCT05497336), incorporated into the pooled study, were conducted in accordance with the Declaration of Helsinki and International Council for Harmonisation Guidelines for Good Clinical Practice. The study protocols received approval from the institutional review board or independent ethics committee at each participating centre. All participants provided written informed consent before undergoing any study procedures.

### Patients

This study pooled efficacy and safety data from a multi-centre, open-label, phase I/II study in KRAS^G12C^ inhibitor-naïve patients with *KRAS*^G12C^-mutated advanced solid tumours (NCT05005234) and a multi-centre, open-label, phase Ib/III study in KRAS^G12C^ inhibitor-naïve patients with *KRAS*^G12C^-mutated metastatic CRC (NCT05497336). Patients aged 18 years or older with histologically confirmed *KRAS*^G12C^-mutated metastatic CRC and an ECOG PS score of 0–1, who had received at least one dose of single-agent IBI351 in both trials, were eligible for this pooled study. As a result, 48 CRC patients from phase I of NCT05005234 and eight from phase Ib of NCT05497336 were included (Fig. 1). The RP2D of IBI351 was established at 600 mg BID, based on the results of a phase Ia study (dose escalation and expansion; NCT05005234), as previously described.^16^ Further details regarding the study design, eligibility criteria, and treatment protocols of these two trials are provided in Supplementary Materials and Protocols.

### Efficacy assessment

Tumour response was assessed using enhanced computed tomography or magnetic resonance imaging according to Response Evaluation Criteria in Solid Tumors (RECIST) version 1.1. Assessments were conducted every six weeks from the first dose until week 48, and every 12 weeks thereafter, until PD, initiation of new anti-tumour therapy, death, withdrawal of informed consent, loss to follow-up, or study completion. CR and PR were confirmed at least four weeks after the first documented response. The primary endpoint of this pooled study was ORR assessed by local investigators. Secondary endpoints included DCR, DoR, TTR, PFS, and OS per RECIST v1.1. These Efficacy endpoints are detailed in the Supplementary Materials and protocols.

### Safety assessment

A formal sample size calculation was not conducted for this pooled analysis. Safety analyses were based on the assessments of adverse events, vital signs, laboratory abnormalities, ECOG PS, and electrocardiograms. Safety data were collected from the first dose until 30 days after the last dose or the initiation of new anti-tumour therapy, whichever occurred first. Adverse events were coded per Medical Dictionary for Regulatory Activities (MedDRA) v26.0 and graded according to the National Cancer Institute Common Terminology Criteria for Adverse Events v5.0.

### Statistical analysis

Continuous and categorical variables were described using median (range) and count (percentage), respectively. The 95% CIs for ORR and DCR were calculated using the Clopper–Pearson method. TTR was descriptively analysed. The Kaplan–Meier (KM) method was used to estimate DoR, PFS, and OS, with medians and their 95% CIs provided. KM curves for PFS and OS were plotted using GraphPad Prism software version 9 (https://www.graphpad.com/scientific-software/prism/). All statistical analyses were conducted using SAS version 9.4 (SAS Institute, Cary, NC, USA) and R version 4.3.2 (https://www.r-project.org/). The pooled analysis incorporated data from both trials with a cutoff date of December 13, 2023.

## Supplementary information

Supplementary materials

Protocol for NCT05005234

Protocol for NCT05497336

## Data Availability

All data and materials that underlie the results reported here are available within the submitted materials.
