# Supplementary material for: Efficacy and safety of IBI351 (fulzerasib) monotherapy in KRASG12C inhibitor-naïve Chinese patients with KRASG12C-mutated metastatic colorectal cancer: a pooled analysis from phase I part of two studies
Source: Signal Transduct Target Ther. 2025 Jul 25;10:241. doi: 10.1038/s41392-025-02315-7 (PMC12297441; doi:10.1038/s41392-025-02315-7)
Supplement: Supplementary file 1 — Supplementary materials [file 41392_2025_2315_MOESM1_ESM.docx]

**Supplementary Materials for**

Efficacy and safety of IBI351 (fulzerasib) monotherapy in KRAS^G12C^ inhibitor-naïve Chinese patients with *KRAS*^G12C^-mutated metastatic colorectal cancer: a pooled analysis from phase I part of two studies

Ying Yuan, Yanhong Deng, Yongdong Jin, Zengqing Guo, Yueyin Pan, Cunji Wang, Zhiwu Wang, Yi Hu, Dong Hua, Xiangjiao Meng, Zhiye Zhang, Mingfang Zhao, Xiaorong Dong, Dingzhi Huang, Xiaoyan Li, Lian Liu, Meili Sun, Huijuan Wang, Xiuwen Wang, Nong Yang, Mingjun Zhang, Sheng Hu, Dongde Wu, Jingjing Huang, Sujie Zhang, Mengna Huang, Kefeng Ding

Correspondence to: [dingkefeng@zju.edu.cn](mailto:dingkefeng@zju.edu.cn)

This PDF file includes:

Materials and Methods

Tables S1 to S4

Materials and Methods

**Study design and patient eligibility of the phase I/II study**

In this phase I/II study (ClinicalTrials.gov identifier: NCT05005234), phase I evaluated IBI351 monotherapy in two parts: phase Ia, which included dose escalation and expansion to evaluate safety, tolerability, maximum tolerated dose (MTD), and recommended phase II dose (RP2D) in patients with advanced solid tumours harboring *KRAS*^G12C^ mutation. An accelerated titration design (assessing 250 mg once daily [QD]) and then a Bayesian optimal interval (BOIN) design (assessing 450/700/900 mg QD, and 450/600/750 mg twice daily [BID]) were used in phase Ia dose escalation. Phase Ib and phase II were designed to evaluate the anti-tumour efficacy of IBI351 (also known as fulzerasib or GFH925) monotherapy and/or IBI351 combined with cetuximab in patients with *KRASG12C*-mutated advanced colorectal cancer (CRC) or other solid tumours, and IBI351 monotherapy in non-small cell lung cancer, respectively. The subset of CRC patients who received IBI351 monotherapy across all dose levels in the phase I study were included in the pooled analysis.

In phase I, patient inclusion criteria were as follows: aged at least 18 years; with pathologically documented metastatic solid tumours; refusing, intolerant to, or refractory to standard therapy; with *KRAS*^G12C^ mutation; with at least one evaluable lesion according to Response Evaluation Criteria in Solid Tumors version 1.1 (RECIST v1.1); Eastern Cooperative Oncology Group performance status (ECOG PS) of 0─1; adequate hematological and organ function. Key exclusion criteria included previously treated with KRAS^G12C^ inhibitor; unstable brain metastases; significant gastrointestinal diseases, such as intractable hiccup, nausea, vomiting, severe gastrointestinal ulcers, cirrhosis, active gastrointestinal bleeding, or other diseases that affect swallowing tablets or significantly affect oral drug absorption.

In phase Ia, the primary endpoints were safety and the incidence of dose-limiting toxicity (DLT). The secondary endpoints were pharmacokinetic parameters and objective response rate (ORR), disease control rate (DCR), duration of response (DoR), time to response (TTR), progression-free survival (PFS), and overall survival (OS). In phase Ib, the primary endpoint were ORR and safety profiles; the secondary endpoints included DCR, DoR, TTR, PFS, 6-month PFS rate, 12-month PFS rate, and 12-month OS rate.

**Study design and patient eligibility of the phase Ib/III study**

For the phase Ib/III study (ClinicalTrials.gov identifier: NCT05497336), phase Ib involved dose escalation and expansion to evaluate the safety, tolerability, recommended dose, and preliminary efficacy of IBI351 (450 mg and 600 mg orally administered, BID) combined with cetuximab (500 mg/m^2^ intravenously administered, every two weeks) in *KRAS*^G12C^*-*mutated metastatic CRC. Among the three cohorts in the phase Ib study, patients in cohort A and cohort B received a combination of IBI351 and cetuximab; patients in cohort C received IBI351 monotherapy (600 mg BID). Phase III was seamlessly designed to compare the efficacy and safety of the combination therapy versus oxaliplatin-based mFOLFOX6 regimen or irinotecan-based FOLFIRI with or without bevacizumab in *KRAS*^G12C^-mutated metastatic CRC. Patients in cohort C of the phase Ib study were included in the pooled analysis.

In phase Ib, patient inclusion criteria were as follows: aged 18 to 75 years; pathologically documented, unresectable and metastatic colorectal cancer; with *KRAS*^G12C^ mutation; with at least one measurable lesion according to RECIST v1.1; refusing, intolerant to, or refractory to systemic therapy; ECOG PS of 0─1; adequate hematological and organ function. Key exclusion criteria included previously treated with KRAS^G12C^ inhibitor; unstable brain metastases diagnosed by investigators; significant gastrointestinal diseases, such as intractable hiccup, nausea, vomiting, severe gastrointestinal ulcers, cirrhosis, active gastrointestinal bleeding, or other diseases that affect swallowing tablets or significantly affect oral drug absorption.

The primary endpoints of phase Ib was the incidence of DLT and ORR. Secondary endpoints included safety profiles, pharmacokinetics characteristics, PFS, OS, DCR, DoR, and TTR.

**Definitions**

ORR as per RECIST v1.1 was calculated as the proportion of patients with a confirmed best overall response of complete response (CR) or partial response (PR). TTR was defined as the time from the date of the first dose of IBI351 to the first documentation of an objective response (CR or PR) that was subsequently confirmed. DoR was defined as the time from the first documentation of objective tumour response (CR or PR) that was subsequently confirmed until the date of first documented disease progression based on RECIST v1.1 or death due to any cause, whichever occurred first. PFS was defined as the time from the date of the first dose of study drug to death due to any cause or disease progression based on RECIST v1.1, whichever occurred first. OS was defined as the time from the date of first dose of IBI351 to death due to any cause. In this study, DoR and TTR were assessed in patients who had a confirmed objective response (*n* = 25). PFS and OS were evaluated in all enrolled patients (*n* = 56).

**Table S1.**

**List of TRAEs leading to dose interruption**

| **TRAE** | **700 mg QD (*n* = 3)** | **450 mg BID (*n* = 4)** | **600 mg BID (*n* = 48)** | **750 mg BID (*n* = 1)** | **Total (N = 56)** |
| --- | --- | --- | --- | --- | --- |
| Any grade TRAEs, *n* (%) | 1 (33.3) | 0 | 11 (22.9) | 0 | 12 (21.4) |
| Grade 2 | 0 | 0 | 1 (2.1) | 0 | 1 (1.8) |
| Grade 3 | 1 (33.3) | 0 | 10 (20.8) | 0 | 11 (19.6) |
| White blood cell count decreased, *n* (%) | 0 | 0 | 3 (6.3) | 0 | 3 (5.4) |
| Grade 2 | 0 | 0 | 2 (4.2) | 0 | 2 (3.6) |
| Grade 3 | 0 | 0 | 1 (2.1) | 0 | 1 (1.8) |
| Gamma-glutamyltransferase increased, *n* (%) | 1 (33.3) | 0 | 1 (2.1) | 0 | 2 (3.6) |
| Grade 3 | 1 (33.3) | 0 | 1 (2.1) | 0 | 2 (3.6) |
| Neutrophil count decreased, *n* (%) | 0 | 0 | 2 (4.2) | 0 | 2 (3.6) |
| Grade 1 | 0 | 0 | 1 (2.1) | 0 | 1 (1.8) |
| Grade 3 | 0 | 0 | 1 (2.1) | 0 | 1 (1.8) |
| Alanine aminotransferase increased, *n* (%) | 1 (33.3) | 0 | 0 | 0 | 1 (1.8) |
| Grade 1 | 1 (33.3) | 0 | 0 | 0 | 1 (1.8) |
| Aspartate aminotransferase increased, *n* (%) | 1 (33.3) | 0 | 0 | 0 | 1 (1.8) |
| Grade 1 | 1 (33.3) | 0 | 0 | 0 | 1 (1.8) |
| Platelet count decreased, *n* (%) | 0 | 0 | 1 (2.1) | 0 | 1 (1.8) |
| Grade 3 | 0 | 0 | 1 (2.1) | 0 | 1 (1.8) |
| Blood alkaline phosphatase increased, *n* (%) | 1 (33.3) | 0 | 0 | 0 | 1 (1.8) |
| Grade 2 | 1 (33.3) | 0 | 0 | 0 | 1 (1.8) |
| Anaemia, *n* (%) | 0 | 0 | 4 (8.3) | 0 | 4 (7.1) |
| Grade 3 | 0 | 0 | 4 (8.3) | 0 | 4 (7.1) |
| Peripheral sensory neuropathy, *n* (%) | 0 | 0 | 2 (4.2) | 0 | 2 (3.6) |
| Grade 3 | 0 | 0 | 2 (4.2) | 0 | 2 (3.6) |
| Asthenia, *n* (%) | 0 | 0 | 1 (2.1) | 0 | 1 (1.8) |
| Grade 3 | 0 | 0 | 1 (2.1) | 0 | 1 (1.8) |

TRAE, treatment-related adverse event; QD, once daily; BID, twice daily.

**Table S2.**

**Summary of TRAEs leading to dose reduction**

| **Clinical trial** | **Patient ID** | **Target dose** | **No. of dose level reduced** | **Dose level reduced to** | **TRAE leading to dose reduction** |
| --- | --- | --- | --- | --- | --- |
| NCT05005234 | 07010 | 600 mg BID | 1 | 450 mg BID | Grade 3 platelet count decreased |
|  | 07016 | 600 mg BID | 1 | 450 mg BID | Grade 1 anaemia |
|  | 09020 | 600 mg BID | 3 | 600 mg QD | Grade 2 peripheral sensory neuropathy |
|  | 45002 | 600 mg BID | 2 | 750 mg QD | Grade 2 asthenia and grade 2 pruritus |
|  | 50003 | 600 mg BID | 1 | 450 mg BID | Grade 3 gamma-glutamyltransferase increased |
| NCT05497336 | 01004 | 600 mg BID | 1 | 450 mg BID | Grade 3 anaemia |

TRAE, treatment-related adverse event; BID, twice daily; QD, once daily.

**Table S3.**

**Full list of treatment-related adverse events**

| **TRAE** | **700 mg QD**  **(*n* = 3)** | **450 mg BID**  **(*n* = 4)** | **600 mg BID**  **(*n* = 48)** | **750 mg BID**  **(*n* = 1)** | **Total**  **(N = 56)** |
| --- | --- | --- | --- | --- | --- |
| Any grade TRAE, n (%) | 3 (100) | 4 (100) | 45 (93.8) | 1 (100) | 53 (94.6) |
| Anaemia | 2 (66.7) | 2 (50.0) | 24 (50.0) | 0 | 28 (50.0) |
| White blood cell count decreased | 0 | 1 (25.0) | 17 (35.4) | 0 | 18 (32.1) |
| Blood bilirubin increased | 1 (33.3) | 1 (25.0) | 14 (29.2) | 1 (100) | 17 (30.4) |
| Pruritus | 0 | 0 | 15 (31.3) | 0 | 15 (26.8) |
| Neutrophil count decreased | 0 | 0 | 13 (27.1) | 0 | 13 (23.2) |
| Aspartate aminotransferase increased | 2 (66.7) | 3 (75.0) | 7 (14.6) | 0 | 12 (21.4) |
| Protein urine present | 0 | 2 (50.0) | 10 (20.8) | 0 | 12 (21.4) |
| Alanine aminotransferase increased | 2 (66.7) | 3 (75.0) | 6 (12.5) | 0 | 11 (19.6) |
| Gamma-glutamyltransferase increased | 2 (66.7) | 2 (50.0) | 6 (12.5) | 0 | 10 (17.9) |
| Asthenia | 0 | 2 (50.0) | 8 (16.7) | 0 | 10 (17.9) |
| Hypoalbuminaemia | 0 | 0 | 9 (18.8) | 0 | 9 (16.1) |
| Hypoaesthesia | 0 | 1 (25.0) | 8 (16.7) | 0 | 9 (16.1) |
| Oedema peripheral | 0 | 1 (25.0) | 8 (16.7) | 0 | 9 (16.1) |
| Blood alkaline phosphatase increased | 1 (33.3) | 2 (50.0) | 4 (8.3) | 0 | 7 (12.5) |
| Bilirubin conjugated increased | 0 | 1 (25.0) | 5 (10.4) | 0 | 6 (10.7) |
| Blood lactate dehydrogenase increased | 0 | 0 | 6 (12.5) | 0 | 6 (10.7) |
| Platelet count decreased | 0 | 0 | 6 (12.5) | 0 | 6 (10.7) |
| Hyperphosphataemia | 0 | 0 | 5 (10.4) | 1 (100) | 6 (10.7) |
| Rash | 1 (33.3) | 1 (25.0) | 4 (8.3) | 0 | 6 (10.7) |
| Electrocardiogram QT prolonged | 0 | 1 (25.0) | 4 (8.3) | 0 | 5 (8.9) |
| Hypertriglyceridaemia | 0 | 2 (50.0) | 3 (6.3) | 0 | 5 (8.9) |
| Pain in extremity | 0 | 0 | 5 (10.4) | 0 | 5 (8.9) |
| Lymphocyte count decreased | 0 | 0 | 4 (8.3) | 0 | 4 (7.1) |
| Lipase increased | 0 | 0 | 4 (8.3) | 0 | 4 (7.1) |
| Blood bilirubin unconjugated increased | 0 | 1 (25.0) | 3 (6.3) | 0 | 4 (7.1) |
| Bile acids increased | 0 | 0 | 3 (6.3) | 0 | 3 (5.4) |
| High density lipoprotein decreased | 0 | 0 | 3 (6.3) | 0 | 3 (5.4) |
| Hypocalcaemia | 0 | 0 | 3 (6.3) | 0 | 3 (5.4) |
| Peripheral sensory neuropathy | 0 | 1 (25.0) | 2 (4.2) | 0 | 3 (5.4) |
| Arthralgia | 0 | 0 | 3 (6.3) | 0 | 3 (5.4) |
| Weight increased | 0 | 0 | 2 (4.2) | 0 | 2 (3.6) |
| Urobilinogen urine increased | 0 | 0 | 2 (4.2) | 0 | 2 (3.6) |
| White blood cells urine positive | 0 | 0 | 2 (4.2) | 0 | 2 (3.6) |
| Urine bilirubin increased | 0 | 0 | 2 (4.2) | 0 | 2 (3.6) |
| Electrocardiogram ST-T change | 0 | 0 | 2 (4.2) | 0 | 2 (3.6) |
| Electrocardiogram T wave abnormal | 0 | 0 | 2 (4.2) | 0 | 2 (3.6) |
| Activated partial thromboplastin time prolonged | 0 | 0 | 2 (4.2) | 0 | 2 (3.6) |
| Blood phosphorus increased | 0 | 0 | 2 (4.2) | 0 | 2 (3.6) |
| Blood fibrinogen increased | 0 | 0 | 2 (4.2) | 0 | 2 (3.6) |
| Blood creatine phosphokinase increased | 0 | 0 | 2 (4.2) | 0 | 2 (3.6) |
| Hypoproteinaemia | 0 | 0 | 2 (4.2) | 0 | 2 (3.6) |
| Hypokalaemia | 0 | 0 | 2 (4.2) | 0 | 2 (3.6) |
| Decreased appetite | 0 | 0 | 2 (4.2) | 0 | 2 (3.6) |
| Hypercholesterolaemia | 0 | 0 | 2 (4.2) | 0 | 2 (3.6) |
| Dysgeusia | 0 | 0 | 2 (4.2) | 0 | 2 (3.6) |
| Dizziness | 0 | 1(25.0) | 1 (2.1) | 0 | 2 (3.6) |
| Nausea | 0 | 0 | 2 (4.2) | 0 | 2 (3.6) |
| Sinus tachycardia | 1(33.3) | 0 | 1 (2.1) | 0 | 2 (3.6) |
| Urinary tract infection | 0 | 0 | 1 (2.1) | 1 (100) | 2 (3.6) |
| Low density lipoprotein increased | 0 | 0 | 1 (2.1) | 0 | 1 (1.8) |
| Urine leukocyte esterase positive | 0 | 0 | 1 (2.1) | 0 | 1 (1.8) |
| Protein urine present | 0 | 0 | 1 (2.1) | 0 | 1 (1.8) |
| Electrocardiogram P wave abnormal | 0 | 0 | 1 (2.1) | 0 | 1 (1.8) |
| Electrocardiogram PR shortened | 0 | 0 | 1 (2.1) | 0 | 1 (1.8) |
| Electrocardiogram ST segment depression | 0 | 0 | 1 (2.1) | 0 | 1 (1.8) |
| Electrocardiogram T wave inversion | 0 | 0 | 1 (2.1) | 0 | 1 (1.8) |
| Electrocardiogram T wave amplitude decreased | 0 | 0 | 1 (2.1) | 0 | 1 (1.8) |
| Electrocardiogram high voltage | 0 | 0 | 1 (2.1) | 0 | 1 (1.8) |
| Protein total decreased | 0 | 0 | 1 (2.1) | 0 | 1 (1.8) |
| Very low density lipoprotein increased | 0 | 0 | 1 (2.1) | 0 | 1 (1.8) |
| Amylase increased | 0 | 0 | 1 (2.1) | 0 | 1 (1.8) |
| White blood cell count increased | 0 | 0 | 1 (2.1) | 0 | 1 (1.8) |
| Blood homocysteine increased | 0 | 0 | 1 (2.1) | 0 | 1 (1.8) |
| Blood urea increased | 0 | 0 | 1 (2.1) | 0 | 1 (1.8) |
| Blood creatinine increased | 0 | 0 | 1 (2.1) | 0 | 1 (1.8) |
| Hypoglycaemia | 0 | 0 | 1 (2.1) | 0 | 1 (1.8) |
| Hyponatraemia | 0 | 0 | 1 (2.1) | 0 | 1 (1.8) |
| Hyperkalaemia | 0 | 1 (25.0) | 0 | 0 | 1 (1.8) |
| Rash maculo-papular | 0 | 0 | 1 (2.1) | 0 | 1 (1.8) |
| Ecchymosis | 0 | 0 | 1 (2.1) | 0 | 1 (1.8) |
| Pigmentation disorder | 0 | 0 | 1 (2.1) | 0 | 1 (1.8) |
| Drug eruption | 0 | 0 | 1 (2.1) | 0 | 1 (1.8) |
| Taste disorder | 1 (33.3) | 0 | 0 | 0 | 1 (1.8) |
| Parosmia | 1 (33.3) | 0 | 0 | 0 | 1 (1.8) |
| Peripheral motor neuropathy | 0 | 0 | 1 (2.1) | 0 | 1 (1.8) |
| Paraesthesia | 0 | 0 | 0 | 1 (100) | 1 (1.8) |
| Hypersomnia | 0 | 1 (25.0) | 0 | 0 | 1 (1.8) |
| Pyrexia | 0 | 0 | 1 (2.1) | 0 | 1 (1.8) |
| Pain | 0 | 0 | 1 (2.1) | 0 | 1 (1.8) |
| Face oedema | 0 | 0 | 1 (2.1) | 0 | 1 (1.8) |
| Limb discomfort | 0 | 0 | 1 (2.1) | 0 | 1 (1.8) |
| Constipation | 0 | 0 | 1 (2.1) | 0 | 1 (1.8) |
| Diarrhoea | 0 | 0 | 1 (2.1) | 0 | 1 (1.8) |
| Supraventricular extrasystoles | 0 | 0 | 1 (2.1) | 0 | 1 (1.8) |
| Ventricular extrasystoles | 0 | 0 | 1 (2.1) | 0 | 1 (1.8) |
| Atrial fibrillation | 0 | 0 | 1 (2.1) | 0 | 1 (1.8) |
| Sinus arrhythmia | 0 | 0 | 1 (2.1) | 0 | 1 (1.8) |
| Cough | 0 | 0 | 1 (2.1) | 0 | 1 (1.8) |
| Productive cough | 0 | 0 | 1 (2.1) | 0 | 1 (1.8) |
| Pneumonitis | 0 | 0 | 1 (2.1) | 0 | 1 (1.8) |
| Breast hyperplasia | 0 | 0 | 1 (2.1) | 0 | 1 (1.8) |
| Gynaecomastia | 0 | 0 | 1 (2.1) | 0 | 1 (1.8) |
| Renal failure | 0 | 0 | 1 (2.1) | 0 | 1 (1.8) |

TRAE, treatment-related adverse event; QD, once daily; BID, twice daily.

**Table S4.**

**Summary of efficacies of IBI351 and other existing KRAS^G12C^ inhibitors as monotherapy in *KRAS*^G12C^-mutated metastatic colorectal cancer**

| **Treatment** | **Clinical trial** | **No. of patients** | **Dose level** | **Confirmed ORR** | **Median PFS (months)** |
| --- | --- | --- | --- | --- | --- |
| Sotorasib^19^ | NCT03600883 (CodeBreaK100), Phase I | 42 | 180/360/720/960 mg QD | 7.1% (95% CI: 1.5–19.5) | 4.0 (range: 0.0+ to 11.1+) |
| Sotorasib^22^ | NCT03600883 (CodeBreaK100), Phase II | 62 | 960 mg QD | 12.9% (95% CI: 5.7–23.9) | 4.0 (95% CI: 2.8–4.2) |
| Adagrasib^23^ | NCT03785249 (KRYSTAL-1), Phase I/II | 44 | 600 mg BID | 19% (95% CI: 8–33) | 5.6 (95% CI: 4.1–8.3) |
| Divarasib^20^ | NCT04449874, Phase I | 55 | 50/100/200/400 mg QD | 29.1% (95% CI: 17.6–42.9) | 5.6 (95% CI: 4.1–8.2) |
|  |  | 39 | 400 mg QD | 35.9% (95% CI: 21.2–52.8) | 6.9 (95% CI: 5.3–9.1) |
| Garsorasib^24^ | NCT04585035, Phase II | 26 | 600 mg BID | 19.2% (95% CI: 6.6–39.4) | 5.5 (95% CI: 2.9–11.6) |
| D3S-001^21^ | NCT05410145, Phase I/II | 9 | 50–900 mg QD | 77.8% (95% CI: 40.0–97.2) | Not reported |
| IBI351 | NCT05497336 and NCT05005234, Phase I | 56 | 450/600/750 mg BID and 700 mg QD | 44.6% (95% CI: 31.3–58.5) | 8.2 (95% CI: 5.6–13.8) |
| (This study) |  | 34 | 600 mg BID | 45.8% (95% CI: 31.4–60.8) | 8.2 (95% CI: 5.6–NR) |

No., number; QD, once daily; BID, twice daily; ORR, objective response rate; CI, confidence interval; NR, not reached; PFS, progression-free survival.
