# Supplementary material for: Efficacy and safety of IBI351 (fulzerasib) monotherapy in KRASG12C inhibitor-naïve Chinese patients with KRASG12C-mutated metastatic colorectal cancer: a pooled analysis from phase I part of two studies
Source: Signal Transduct Target Ther. 2025 Jul 25;10:241. doi: 10.1038/s41392-025-02315-7 (PMC12297441; doi:10.1038/s41392-025-02315-7)
Supplement: Supplementary file 2 — Protocol for NCT05005234 [file 41392_2025_2315_MOESM2_ESM.pdf]

Clinical Study Protocol

**Study title:**	An open-label, multicenter, phase I/II clinical study evaluating the safety/tolerability, pharmacokinetics and effectiveness of GFH925 in patients with advanced solid tumors with KRAS G12C mutation
**Protocol No.:**	GFH925X1101
**Version No. & Version Date:**	9 Oct 2022/Version 6.0
**Product Name:**	GFH925
**Study Phase:**	Phase I/II
**Sponsor:**	Innovent Biologics (Suzhou) Co., Ltd.<br>168 Dongping Street, Suzhou Industrial Park, Jiangsu Province, China
**Sponsor Contact:**	[redacted]

Confidentiality Statement

This document contains confidential information of Innovent Biologics (Suzhou) Co., Ltd. The contents of this document may not be disclosed to anyone other than the investigator (s), study advisor (s), or related personnel, institutional review board (s)/independent ethics committee (IEC). The information in this document may not be used for any purpose other than the evaluation or conduct of this clinical investigation without written permission from the sponsor.

SPONSOR SIGNATURE PAGE

Title of the study: An open-label, multicenter, phase I/II clinical study evaluating the safety/tolerability, pharmacokinetics and effectiveness of GFH925 in patients with advanced solid tumors with KRAS G12C mutation

Protocol No.: GFH925X1101

**Position**	**Name**	**Signature (printed)**	**Date**
Medical Science Director		________________	________________
Associate Director, Biostatistics		________________	________________

Investigator Signature Page

Protocol Title: An open-label, multicenter, phase I/II clinical study evaluating the safety/tolerability, pharmacokinetics and effectiveness of GFH925 in patients with advanced solid tumors with KRAS G12C mutation

Protocol No.: GFH925X1101

This protocol is a trade secret of Innovent Biologics (Suzhou) Co., Ltd. I have read and fully understand this protocol and commit to conduct this study in accordance with this protocol and Good Clinical Practice and in compliance with applicable laws and regulations, as well as the Declaration of Helsinki. I also promise not to disclose any confidential information in this study to any third party without the written consent of Innovent Biologics (Suzhou) Co., Ltd.

**Investigator Guidance Note:**<br>Please sign and date this signature page, print the investigator 's name, title, and name of the site conducting the study and return to Innovent Biologics (Suzhou) Co. after signing.

I have read all the contents of this protocol and promise to conduct this study as required:

Investigator Signature:

Date

Printed Name: _

Investigator Title: _

Tel.

Site Name/Address:

Synopsis

**Study Number**	GFH925X1101
**Study Sponsor**	Innovent Biologics (Suzhou) Co., Ltd.
**Study Drug**	GFH925
**Study Title**	An open-label, multicenter, phase I/II clinical study evaluating the safety/tolerability, pharmacokinetics and effectiveness of GFH925 in patients with advanced solid tumors with KRAS G12C mutation
**Study Phase**	Phase I/II
**Version Number / Date**	V6.0/ Oct 9, 2022
**Study Duration**	Estimated 36 months
**Study objectives/endpoints**	This study is a Phase I/II study.<br>The objectives and endpoints of Phase Ia include:

Study objectives	Study endpoints
Primary objectives	Primary endpoints
• To evaluate the safety/tolerability of GFH925 in patients with KRAS G12C-mutated advanced solid tumors<br>• To estimate the maximum tolerated dose (MTD) and/or recommended Phase II dose (RP2D) of GFH925	• Incidence and severity of adverse events (AEs) and serious adverse events (SAEs); changes in laboratory tests, vital signs, physical examinations, electrocardiograms (ECGs)<br>• Incidence of dose-limiting toxicity (DLT) events
Secondary objectives	Secondary endpoints
• To evaluate the pharmacokinetic (PK) profile of GFH925 in patients with KRAS G12C -mutated advanced solid tumors<br>• To preliminarily evaluate the efficacy of GFH925 in patients with advanced solid tumors with KRAS G12C mutation	• PK parameters of GFH925 include but are not limited to: C_{max}, T_{max}, AUC, $t_{1/2}$, CL/F and Vd/F<br>• Objective response rate (ORR) per Response Evaluation Criteria in Solid Tumors (RECIST) 1.1, disease control rate (DCR), duration of response (DoR), time to response (TTR), progression-free survival (PFS)

	• Overall survival (OS)
Exploratory objectives	Exploratory endpoints
• To explore potential mechanisms of primary and secondary resistance to GFH925	• Correlation between efficacy and genetic mutation/gene fusion in tumor tissue and peripheral blood samples obtained at baseline and/or disease progression

The objectives and endpoints of Phase Ib include:

**Study objectives**	**Study endpoints**
**Primary objectives**	**Primary endpoints**
• To evaluate the efficacy of GFH925 in subjects with KRAS G12C-mutated advanced colorectal cancer or other solid tumors	• ORR per RECIST 1.1
**Secondary objectives**	**Secondary endpoints**
• To evaluate the efficacy of GFH925 in subjects with KRAS G12C-mutated advanced colorectal cancer or other solid tumors using additional efficacy measures<br>• To evaluate the safety of GFH925 in subjects with KRAS G12C-mutated advanced colorectal cancer or other solid tumors<br>• To evaluate the PK profile of GFH925 in subjects with KRAS G12C-mutated advanced colorectal cancer or other solid tumors	• DCR, DoR, TTR, PFS per RECIST 1.1, progression-free survival rate at 6 and 12 months<br>• OS, overall survival rate at 12 months<br>• Incidence and severity of AEs, SAEs, AEs leading to treatment interruption, and AEs leading to treatment discontinuation<br>• Plasma concentration (including C_{trough}) after multiple dose administration in subjects
**Exploratory objectives**	**Exploratory endpoints**

	• To explore potential mechanisms of primary and secondary resistance to GFH925	• Correlation between efficacy and genetic mutation/gene fusion in tumor tissue and peripheral blood samples obtained at baseline and/or disease progression
	[redacted]	
	[redacted]	[redacted]
	[redacted]	[redacted]
**Study design**	This study is the first-in-human trial of GFH925 which consists of two	

	parts: Phase I and Phase II.<br>**Phase Ia: Dose escalation and dose expansion study of GFH925 in subjects with advanced solid tumors.**<br>The Phase Ia part is an open-label, multicenter dose escalation and dose expansion study in which eligible subjects with KRAS G12C-mutated advanced solid tumors will be enrolled to receive GFH925 monotherapy to evaluate the safety/tolerability of GFH925 in KRAS G12C-mutated advanced solid tumor subjects and to estimate the MTD and/or RP2D of GFH925, as well as to evaluate the pharmacokinetic profile.<br>Subjects will receive daily oral treatment with GFH925 at the designated dose after enrollment and the first 21 days of treatment will serve as the DLT observation period. In the absence of DLT events, subjects will continue to receive treatment and will be allowed to undergo dose escalation as permitted by this protocol and treatment will continue until disease progression or intolerable toxicity or other conditions that lead to withdrawal or discontinuation from treatment.<br>During the dose escalation phase, 4 sequentially increasing QD dose levels (Cohorts 1-4): 250 mg QD, 450 mg QD, 700 mg QD, 900 mg QD, and 4 sequentially increasing BID dose levels (Cohorts 5-8): 450 mg BID, 600 mg BID, 750 mg BID, 900 mg BID are initially proposed. After completion of DLT observation and confirmation of safety data in the Cohort 3 (700 mg QD) dose group, dose escalation will be performed simultaneously in Cohort 4 (900 mg QD) and Cohort 5 (450 mg BID) (900 mg total) (see Table 4).<br>Adjustments to the dose regimen (dose and interval) will be made during dose escalation based on various factors including preclinical in vitro and in vivo activity, clinical PK, safety, efficacy data, and data from clinical trials with the same therapeutic target. The anticipated benefit of GFH925 will be assessed dynamically and comprehensively, which may lead to actions including but not limited to, cessation of dose escalation at the discretion of the sponsor and the investigator upon confirmation of drug absorption saturation.<br>Dose expansion phase: The sponsor may select potentially effective dose groups based on PK, preliminary efficacy and safety data, and further expand the enrollment of up to 25 NSCLC subjects after completion of DLT observation and confirmation of safety.<br>**Definition of DLT**

	DLT is defined as an adverse event related to the study drug GFH925 occurring within 21 days of the first dose, and meeting the following severity criteria. Severity of adverse events are graded according to Common Terminology Criteria for Adverse Events (CTCAE) version 5.0.<br>Non-hematological toxicity:<br>- Vomiting or diarrhea ≥ Grade 4;<br>- Vomiting or diarrhea ≥ Grade 3, for more than 3 days despite optimal medical support;<br>- Nausea ≥ grade 3, for more than 3 days despite optimal medical support;<br>- Other ≥ Grade 3 non-hematologic adverse events.<br>Hematologic toxicity:<br>- Grade 4 neutrophil count (ANC) decreasing for more than 5 days; or Grade 3 febrile neutrophil count decreasing (ANC < 1.0 × 10^9/L with body temperature > 38.3℃); or neutrophil count decreasing with infection requiring clinical intervention;<br>- Grade 4 thrombocytopenia or Grade 3 thrombocytopenia with bleeding requiring clinical intervention;<br>- Grade 4 anemia.<br>In addition, in the event of any other treatment-related toxicity that may be considered as DLT, the investigators and the sponsor will decide jointly whether it is DLT or not after discussion; toxicities beyond the DLT observation period but meeting the above definition will also be important factors in confirming the safety of the current dose.<br>**Dose escalation rules**<br>Accelerated titration will be used at the first dose level and Bayesian optimal interval (BOIN) design will be used for subsequent dose levels. That is, 1 subject will be enrolled at the first dose level, and if there is no study drug-related adverse event with severity ≥ Grade 2 during the DLT observation period , the study will enroll new subjects at the second dose level, and BOIN design will be used for this group and subsequent dose escalation; otherwise, if the first subject develop other adverse events with severity ≥ Grade 2 related to the study drug, the accelerated titration method will be abandoned and we will directly employ the BOIN design.<br>The target toxicity rate for the maximum tolerated dose in the dose escalation part of this study is 0.3, with a planned maximum sample size

	of 45. The MTD is defined as the dose level at which the incidence of DLTs is closest to the target toxicity rate (i.e., 0.3) within 21 days of the subject receiving the first dose of study drug. Subjects will be enrolled and treated at 3 to 6 per group. BOIN is designed to use the following optimization rules for dose escalation and de-escalation decisions:<br>- If the estimated DLT rate at the current dose is ⩽ 0.236, escalate the dose to the next higher dose level;<br>- If the estimated DLT rate at the current dose is ⩾ 0.359, de-escalate the dose to the next lower dose level;<br>- Otherwise, stay at the current dose.<br>Subjects will be assigned to the lowest starting dose level using the BOIN design. Afterwards, the next group of subjects will be assigned a dose level according to the dose escalation and de-escalation rules shown in the table below. If none of the rules are met (i.e., escalate, de-escalate, or eliminate dose), the current dose will be used to treat the next group of subjects. The dose escalation part will be terminated when there are ⩾ 9 evaluable subjects at the recommended next dose level.

BOIN DESIGN DOSE ESCALATION AND DE-ESCALATION DECISION RULES (Target Toxicity Rate = 0.3)

DECISION	Total number of subjects treated at current dose								
	1	2	3	4	5	6	7	8	9
Escalate if number of subjects with DLT ⩽	0	0	0	0	1	1	1	1	2
De-escalate if number of DLT subjects ⩾	1	1	2	2	2	3	3	3	4
Eliminate if number of subjects with DLT ⩾	NA	NA	3	3	4	4	5	5	5

Dose expansion rules:

The sponsor may select a potentially effective dose group based on PK, preliminary efficacy and safety data, and further expand the enrollment of up to 25 NSCLC subjects after completion of DLT observation and confirmation of safety.

Phase Ib part:

Phase Ib part is an open-label, multicenter single-arm study. Following achievement of the recommended Phase II dose (RP2D) in Phase Ia, 40 to 200 subjects with advanced colorectal cancer or other solid tumors with KRAS G12C mutations will be enrolled and

	administered with GFH925 tablets at RP2D in 21-day cycles to evaluate the efficacy.<br>• CRC cohort: 20 to 80 subjects with advanced colorectal cancer with KRAS G12C mutation will be enrolled;<br>• Cohort for non-CRC solid tumors: 20 to 120 subjects with advanced tumors other than colorectal cancer with KRAS G12C mutations will be enrolled.<br>**Phase II part: Efficacy study of GFH925 monotherapy in advanced NSCLC.**<br>[redacted]

	[redacted]<br>**Definition of study completion**<br>The study will be considered complete when the last subject has been treated for 2 years or has reached end of treatment, whichever comes first.
**Inclusion criteria**	Subjects participating in this study are required to meet all of the following criteria:<br>1. Volunteer to participate in the study and sign the informed consent form.<br>2. Aged 18 years or older at the time of signing the informed consent form.<br>3. Histologically or cytologically confirmed advanced tumors meeting the following requirements:<br>• Phase I: advanced NSCLC or other tumors, which progressed after standard treatment, or subjects were intolerant to or refused to receive standard treatment;<br>• [redacted].<br>4. Have valid report documenting the presence of KRAS G12C mutation:<br>• Phase I: With documented KRAS G12C mutation and can provide qualified archival tumor tissue sample or undergo biopsy prior to treatment; if archival tissue or biopsy is not available, enrollment is allowed based on other written documents approved by the investigator and the sponsor;<br>• [redacted]<br>5. Subjects in Phase Ia and Phase II must have one measurable lesion (per RECIST 1.1). For Phase Ib, subjects with at least one radiographically evaluable lesion may be enrolled.<br>6. Adequate organ function, including:

	• Hematopoietic function, i.e., absolute neutrophil count (ANC) ≥ 1.5 $\times 10^9$/L, platelet count ≥ 75 × 10^9/L, hemoglobin ≥ 9 g/dL. Subjects should not receive blood transfusion or granulocyte colony-stimulating factor, thrombopoietin, erythropoietin, and other treatments within 14 days before hematology laboratory examination.<br>• Liver function, i.e., serum total bilirubin (TBIL) < 1.5 × upper limit of normal (ULN), aspartate transferase (AST) and alanine aminotransferase (ALT) < 2.5× ULN; subject with Gilbert's syndrome must have total bilirubin < 2 × ULN; subject with liver metastases must have AST and ALT < 5.0 × ULN; TBIL < 3.0 × ULN is permitted if the subject's direct bilirubin (DBIL) suggests extrahepatic obstruction.<br>•Renal function, i.e., serum creatinine (Cr) ≤ 1.5 × ULN or creatinine clearance (CrCl) ≥ 60 mL/min calculated using the Cockcroft-Gault formula (see Appendix 5) when Cr > 1.5 × ULN.<br>•Coagulation function, i.e., prothrombin time (PT) and activated partial thromboplastin time (APTT) < 1.5 × ULN and international normalized ratio (INR) < 1.5 or within target range for anticoagulant therapy.<br>7. Subjects with toxic reaction caused by prior anticancer therapy need to have recovered to baseline level (except residual alopecia) or ≤ Grade 1 (neurotoxicity ≤ Grade 2 acceptable). Subjects with endocrine-related immune-related adverse events (irAEs) caused by prior immunotherapy, such as immune-related hypothyroidism, which are stable and asymptomatic after treatment and still require stable doses of hormone replacement or physiological doses of corticosteroids, can be enrolled after the investigator's assessment that these irAEs do not affect the study drug administration and safety assessment.<br>8. Eastern Cooperative Oncology Group (ECOG) performance status score (PS) 0 ~ 1.<br>9. Expected survival ≥ 12 weeks.<br>10. Female subjects or male subjects of childbearing potential must take effective contraceptive measures from the time of signing the informed consent form to 30 days after the last dose of GFH925. Female subjects of childbearing potential should have a negative blood pregnancy test within 7 days (inclusive) prior to initiation of study treatment.

	11.The investigators deem the subject able to communicate well, attend regular follow-up visits, and complete the study according to the protocol.<br>[redacted]
**Exclusion criteria**	Subjects who meet any of the following criteria could not be enrolled in the study:<br>1. Significant cardiovascular system disease, such as:<br>• Definite cardiovascular abnormality events within 6 months, such as myocardial infarction, angina pectoris, heart failure, severe arrhythmia, or angioplasty, vascular stent implantation, coronary artery bypass surgery;<br>• Clinically significant QT/QTcF interval prolongation (QTcF > 470 ms in females or > 450 ms in males).<br>2. Subjects with unstable brain metastases diagnosed by investigators. Subjects with brain metastases incidentally discovered during screening may also be considered for enrollment if they are asymptomatic and do not require treatment; subjects with stable controlled brain metastases diagnosed by investigators with stable hormonal doses, (prednisone doses ≤ 10 mg/day or corresponding equivalent doses if other steroids are used) may be allowed to enroll.<br>3. Significant gastrointestinal diseases, such as intractable hiccup, nausea, vomiting, severe gastrointestinal ulcers, cirrhosis, active gastrointestinal bleeding, or other diseases that affect swallowing tablets or significantly affect oral drug absorption; subjects with severe portal hypertension caused by the presence of Budd-Chiari syndrome or portal emboli in subjects with liver cancer also need to be excluded.<br>4. Presence of serious acute or chronic infections, including:<br>• Active infection requiring systemic therapy;<br>• Positive human immunodeficiency virus antibody (HIV-Ab) at baseline;<br>• Active hepatitis B virus infection (hepatitis B surface antigen HBsAg positive, and HBV-DNA positive); HBsAg negative, HBcAb positive, further examination of HBV-DNA levels is

	required. Subjects with positive HBV-DNA will be excluded from enrollment;<br>• Active hepatitis C virus infection (hepatitis C antibody HCV-Ab positive and HCV-RNA positive);<br>• Active tuberculosis.<br>5. Pleural and peritoneal effusion or pericardial effusion with repeated drainage or significant symptoms.<br>6. Other uncontrolled systemic diseases even after standard treatment, such as uncontrolled hypertension (systolic blood pressure ≥160 mmHg or diastolic blood pressure ≥ 100 mmHg) and diabetes.<br>7. Other malignancies within 2 years before entering the study, except for appropriately treated cervical carcinoma in situ, focal cutaneous squamous cell carcinoma, basal cell carcinoma, prostate cancer not requiring treatment, ductal carcinoma in situ of the breast, and superficial non-muscle invasive urothelial carcinoma.<br>8. Current clinically significant interstitial lung disease, radiation pneumonitis, or drug-related pneumonia requiring treatment.<br>9. [redacted]<br>10. Prior treatment with KRAS G12C inhibitors.<br>11. Therapeutic or palliative radiation therapy within 14 days prior to initiation of study treatment in this study.<br>12.Chemotherapy, targeted therapy, endocrine therapy, immunotherapy, other investigational drugs or investigational device therapies and other anti-tumor therapies within 28 days or 5 half-lives (whichever is shorter) before initiation of study treatment in this study, except for maintenance endocrine therapy. Subjects who have received proprietary Chinese medicines with clear anti-tumor effect within 7 days prior to initiation of study treatment in this study.<br>13. Surgical procedures (excluding needle biopsy) performed within 28 days prior to enrollment that may affect treatment administration or study assessments in this study.<br>14. Use of strong inhibitors or strong inducers of CYP3A4 or P-gp (see Appendix 6) within 14 days or 5 half-lives of the GFH925 (whichever is longer) prior to initiation of study treatment in this study or took traditional Chinese medicine within 7 days prior to initiation of study treatment in this study.

	15. Received known CYP2D6 and CYP3A4 sensitive substrate within 14 days or 5 half-lives of the GFH925 (whichever is longer) prior to initiation of study treatment, and the substrate has a narrow therapeutic window; enrollment will not be allowed unless approved by the investigators and the sponsor (see Appendix 6).<br>16. Received known proton pump inhibitors and H2 receptor inhibitors within 7 days prior to initiation of study treatment in this study (see Section 5.5.2.1).<br>17. Pregnant or lactating women.<br>18. Known allergy to the study drug or any component of its formulation.<br>19. Other conditions that the investigators consider inappropriate for participation in this study.
**Study Drugs, Strength and Route of Administration**	**GFH925**<br>- Strength: [redacted]<br>- Route of Administration: Oral
**Statistical Methods**	**Sample Size**<br>**Phase I Study:** Phase Ia is a dose escalation and dose expansion study. No statistical hypothesis testing is planned.<br>**Dose escalation part:**<br>Except for the accelerated titration dose group, subjects will be enrolled to receive treatment at 3 to 6 per group, with a planned maximum sample size of approximately 45 subjects. Additional subjects may be enrolled for treatment if additional dose levels or other dosing schedules are to be explored beyond the planned dose levels.<br>**Dose expansion part:**<br>Up to 200 subjects are expected to be enrolled.<br>No statistical hypothesis testing is planned for the Phase Ib study. Forty to two hundred subjects are expected to enroll.<br>[redacted]<br>[redacted]<br>[redacted]

	[redacted]<br><br>**Statistical Analysis Methods**<br>Unless otherwise specified, study data from different study phases will be summarized by dose levels and cohorts (if applicable).<br>Statistical analysis will be performed using SAS 9.4 or higher version.<br>Descriptive statistical methods will be used to summarize the baseline characteristics, demographic data, exposure to study treatment, and other characteristics of the subjects. Unless otherwise specified, continuous data will be described using number of subjects, mean, standard deviation, maximum, minimum, and median; categorical data will be described using frequency and percentage.<br>The frequency and percentage of subjects with treatment-emergent adverse events, adverse events related to study treatment, and serious adverse events will be summarized, and all adverse events will be listed. For Phase Ia dose escalation part, DLTs will be summarized and listed in addition to the above analyses. Other safety endpoints will also be summarized and shift tables will be provided to present the changes in safety endpoints pre- and post-treatment-administration. Listings will also be provided.<br>Efficacy endpoints in the Phase Ia study include investigator-assessed ORR, DCR, DoR, TTR, and PFS, as well as OS. The primary efficacy endpoint of Phase Ib is investigator-assessed ORR. [redacted]

	[redacted].<br>PK parameters will be calculated using non-compartmental analysis, and plasma concentrations at each blood sampling time point and PK parameters will be summarized. Plasma concentration-time curves will be plotted. Listings will also be provided.<br>[redacted]

Table 1. Study Flow Chart: Phase Ia Study - Treatment Phase

**Study Phase**	**Screening Period**	**Treatment period**								**Remarks**
		**Cycle 1**					**Cycle 2**		**Subsequent cycles**	
**Visit Day (Days)**	**D-28 to D-1**	**D1**	**D2**	**D3**	**D10**	**D21**	**D1**	**D10**	**D1**	21 days per cycle
**Visit Window**	**NA**	**0**	**0**	**0**	**± 3**	**± 3**	**± 3**	**± 3**	**± 3**	Visit windows will be calculated based on C1D1 on the day of the first dose of study.
**Informed Consent**	×									
**Inclusion and exclusion criteria**	×	×								Inclusion and exclusion criteria will be reviewed again at baseline.
**Demography**	×									
**Tumor tissue samples**	×									Archival formalin-fixed paraffin-embedded (FFPE) or biopsy samples.
**Oncologic history**	×									Including tumor diagnosis, previous treatment and other information.
**Other medical history**	×									All active conditions will be collected, as well as any conditions considered clinically significant by the investigators.
**Prior Therapy**	×									Including medication and treatment measures, information will be collected within 30 days prior to the first dose.
**Viral Serology Testing**	×									Including hepatitis B surface antigen (HBsAg), hepatitis B surface antibody (HBsAb), hepatitis B core antibody (HBcAb), HBV surface

Study Phase	Screening Period	Treatment period								Remarks
		Cycle 1					Cycle 2		Subsequent cycles	
**Visit Day (Days)**	**D-28 to D-1**	**D1**	**D2**	**D3**	**D10**	**D21**	**D1**	**D10**	**D1**	21 days per cycle<br>Visit windows will be calculated based on C1D1 on the day of the first dose of study.
**Visit Window**	**NA**	**0**	**0**	**0**	**± 3**	**± 3**	**± 3**	**± 3**	**± 3**	
										antigen/HCV antibody/HIV, and HBV-DNA and HCV- RNA tests if necessary.
**Pregnancy test**	×	×							×	Serum pregnancy test will be performed at baseline and urine pregnancy test could be performed for the rest of the study. Results obtained within 7 days of baseline testing need not be repeated; tests should be done every 6 weeks thereafter.
**Physical examination**	×	×	×	×	×	×	×	×	×	
**ECOG**	×	×								ECOG PS score will be assessed at baseline, and when necessary for the rest of the treatment period.
**Vital signs**	×	×		×	×	×		×	×	
**Hematology**	×	×		×	×	×		×	×	C1D1 baseline data should be completed within 1 day before the first dose. If the test results are available within 7 days before the first dose, it is not necessary to repeat the test on C1D1. Laboratory tests will be performed on C1D1 unless the subject's clinical condition changed significantly;
**Urinalysis**	×	×		×	×	×		×	×	
**Blood chemistry**	×	×		×	×	×		×	×	
**Coagulation function**	×	×			×	×		×	×	

<table>
<tr><th rowspan="2">Study Phase</th><th rowspan="2">Screening Period</th><th colspan="8">Treatment period</th><th rowspan="2">Remarks</th></tr>
<tr><th colspan="5">Cycle 1</th><th colspan="2">Cycle 2</th><th>Subsequent cycles</th></tr>
<tr><td>Visit Day (Days)</td><td>D-28 to D-1</td><td>D1</td><td>D2</td><td>D3</td><td>D10</td><td>D21</td><td>D1</td><td>D10</td><td>D1</td><td rowspan="3">21 days per cycle
Visit windows will be calculated based on C1D1 on the day of the first dose of study.
In addition to protocol-specified visit tests, the investigators may repeat as clinically indicated.</td></tr>
<tr><td>Visit Window</td><td>NA</td><td>0</td><td>0</td><td>0</td><td>± 3</td><td>± 3</td><td>± 3</td><td>± 3</td><td>± 3</td></tr>
<tr><td></td><td></td><td></td><td></td><td></td><td></td><td></td><td></td><td></td><td></td></tr>
<tr><td>Electrocardiogram</td><td>×</td><td>×</td><td></td><td>×</td><td>×</td><td>×</td><td>×</td><td></td><td>×</td><td>Three examinations are required during the screening period as detailed in Section 6.1.2.2 ; Rest for at least 5 minutes before examination to avoid interference.</td></tr>
<tr><td>Oncologic imaging</td><td>×</td><td colspan="8">×</td><td>Imaging within 4 weeks before the first dose is acceptable at baseline and does not need to be repeated. Imaging should be performed every 6 weeks ± 1 week during the first 48 weeks before the first dose of the following cycle (weeks 7, 13, …); and every 12 weeks ± 1 week thereafter.</td></tr>
<tr><td>Study treatment</td><td></td><td colspan="8">×</td><td>GFH925 tablets, dispensed at designated visits and administered orally daily as planned (pause on Cycle 1 Day 2 for subjects in the dose escalation phase).</td></tr>
<tr><td>PK blood sampling</td><td></td><td colspan="8">×</td><td>Please refer to PK blood sampling schedule.</td></tr>
<tr><td>Biomarkers</td><td></td><td>×</td><td colspan="7">×</td><td>Plasma and tumor tissue samples will be collected at baseline and at the first tumor progression.</td></tr>
</table>

Study Phase	Screening Period	Treatment period								Remarks
		Cycle 1					Cycle 2		Subsequent cycles	
**Visit Day (Days)**	**D-28 to D-1**	**D1**	**D2**	**D3**	**D10**	**D21**	**D1**	**D10**	**D1**	21 days per cycle<br>Visit windows will be calculated based on C1D1 on the day of the first dose of study.
**Visit Window**	**NA**	**0**	**0**	**0**	**± 3**	**± 3**	**± 3**	**± 3**	**± 3**	
**Concomitant medications**		×								Additional concomitant medications and treatment measures will be collected after initiation of study treatment.
**AE collection**	×	×								AEs will be collected from the time of informed consent through 30 days after the last dose of study treatment.
**SAE collection**	×	×								SAEs will be collected from the time of informed consent through 30 days after the last dose of study treatment.

Table 2 Study Flow Chart: Phase Ib and Phase II Studies － Treatment Phase

Study Phase	Screening Period	Treatment period						Remarks
		Cycle 1			Cycle 2	Cycle 3	Subsequent cycles	
**Visit Day (Days)**	**D-28 to D-1**	**D1**	**D8**	**D14**	**D1**	**D1**	**D1**	21 days per cycle
**Visit Window**	**NA**	**0**	**± 3**	**± 3**	**± 3**	**± 3**	**± 3**	Visit windows will be calculated based on C1D1 on the day of the first dose of study.
**Informed Consent**	×							
**Inclusion and exclusion criteria**	×	×						Inclusion and exclusion criteria will be reviewed again at baseline.
**Demography**	×							
**Tumor tissue samples**	×							Archival formalin-fixed paraffin-embedded (FFPE) or
**Oncologic history**	×							Including tumor diagnosis, previous treatment and other information.
**Other medical history**	×							All active conditions will be collected, as well as any conditions considered clinically significant by the investigators.
**Prior Therapy**	×							Including medication and treatment measures, information will be collected within 30 days prior to the first dose.

**Study Phase**	**Screening Period**	**Treatment period**						**Remarks**
		**Cycle 1**			**Cycle 2**	**Cycle 3**	**Subsequent cycles**	
**Visit Day (Days)**	**D-28 to D-1**	**D1**	**D8**	**D14**	**D1**	**D1**	**D1**	21 days per cycle
**Visit Window**	**NA**	**0**	**± 3**	**± 3**	**± 3**	**± 3**	**± 3**	Visit windows will be calculated based on C1D1 on the day of the first dose of study.
**Viral Serology Testing**	×							Including hepatitis B surface antigen (HBsAg), hepatitis B surface antibody (HBsAb), hepatitis B core antibody (HBcAb), HBV surface antigen/HCV antibody/HIV, and HBV-DNA and HCV- RNA tests if necessary.
**Pregnancy test**	×	×					×	Serum pregnancy test will be performed at baseline and urine pregnancy test could be performed for the rest of the study. Results obtained within 7 days of baseline testing need not be repeated; tests should be done every 6 weeks thereafter.
**Physical examination**	×	×	×	×	×	×	×	
**ECOG**	×	×						ECOG PS score will be assessed at baseline, and when necessary for the rest of the treatment period.
**Vital signs**	×	×	×	×	×	×	×	

**Study Phase**	**Screening Period**	**Treatment period**						**Remarks**
		**Cycle 1**			**Cycle 2**	**Cycle 3**	**Subsequent cycles**	
**Visit Day (Days)**	**D-28 to D-1**	**D1**	**D8**	**D14**	**D1**	**D1**	**D1**	21 days per cycle
**Visit Window**	**NA**	**0**	**± 3**	**± 3**	**± 3**	**± 3**	**± 3**	Visit windows will be calculated based on C1D1 on the day of the first dose of study.
**Hematology**	×	×	×	×	×	×	×	C1D1 baseline data should be completed within 1 day before the first dose. If the test results are available within 7 days before the first dose, it is not necessary to repeat the test on C1D1. Laboratory tests will be performed on C1D1 unless the subject's clinical condition changed significantly;
**Urinalysis**	×	×	×	×	×	×	×	In addition to protocol-specified visit tests, the investigators may repeat as clinically indicated.
**Blood chemistry**	×	×	×	×	×	×	×	
**Coagulation function**	×	×	×	×	×	×	×	
**Electrocardiogram**	×	×	×	×	×	×	×	Three examinations are required during the screening period as detailed in Section 6.1.2.2;<br>Rest for at least 5 minutes before examination to avoid interference.

**Study Phase**	**Screening Period**	**Treatment period**						**Remarks**
		**Cycle 1**			**Cycle 2**	**Cycle 3**	**Subsequent cycles**	
**Visit Day (Days)**	**D-28 to D-1**	**D1**	**D8**	**D14**	**D1**	**D1**	**D1**	21 days per cycle
**Visit Window**	**NA**	**0**	**± 3**	**± 3**	**± 3**	**± 3**	**± 3**	Visit windows will be calculated based on C1D1 on the day of the first dose of study.
**Oncologic imaging**	×	×						Imaging within 4 weeks before the first dose is acceptable at baseline and does not need to be repeated. Imaging should be performed every 6 weeks ± 1 week during the first 48 weeks before the first dose of the following cycle (weeks 7, 13, …); and every 12 weeks ± 1 week thereafter.
**Study treatment**		×						GFH925 tablets, dispensed at designated visits and administered orally daily as planned.
**PK blood sampling**		×						Please refer to PK blood sampling schedule.
**Biomarkers**		×	×					Plasma and tumor tissue samples will be collected at baseline and at the first tumor progression.
**Concomitant medications**		×						Additional concomitant medications and treatment measures will be collected after initiation of study treatment.
**AE collection**	×	×						AEs will be collected from the time of informed consent through 30 days after the last dose of study treatment.

**Study Phase**	**Screening Period**	**Treatment period**						**Remarks**
		**Cycle 1**			**Cycle 2**	**Cycle 3**	**Subsequent cycles**	
**Visit Day (Days)**	**D-28 to D-1**	**D1**	**D8**	**D14**	**D1**	**D1**	**D1**	21 days per cycle
**Visit Window**	**NA**	**0**	**± 3**	**± 3**	**± 3**	**± 3**	**± 3**	Visit windows will be calculated based on C1D1 on the day of the first dose of study.
**SAE collection**	×	×						SAEs will be collected from the time of informed consent through 30 days after the last dose of study treatment.

Table 3 Study Flow Chart: Phase I and Phase II Studies － End of Treatment and Follow-up

Study Phase	End of Treatment Visit	Safety Follow-up Visit	Disease/Survival Follow-up *	Remarks
	Study Treatment Discontinuation	30 days after last dose	Every 8 weeks after safety follow-up visit	
**Visit Window (Days)**	NA	± 3	± 7	**End of treatment visit should occur within 14 days of decision.**
**Pregnancy test**	×	×		Urine pregnancy test, serum test may be performed if necessary.
**Physical examination**	×	×		
**ECOG**	×			
**Vital signs**	×	×		
**Hematology**	×	×		Tests performed within 7 days need not be repeated, but tests related to AEs should be repeated at the investigator's discretion.
**Urinalysis**	×	×		
**Blood chemistry**	×	×		
**Coagulation function**	×	×		
**Electrocardiogram**	×	×		
**Oncologic imaging**	×			Imaging performed within 4 weeks does not need to be repeated; subjects who ended treatment for reasons other than disease progression/start of other

<table>
<tr><th rowspan="2">Study Phase</th><th>End of Treatment Visit</th><th>Safety Follow-up Visit</th><th>Disease/Survival Follow-up *</th><th rowspan="2">Remarks</th></tr>
<tr><th>Study Treatment Discontinuation</th><th>30 days after last dose</th><th>Every 8 weeks after safety follow-up visit</th></tr>
<tr><td></td><td></td><td></td><td></td><td>antineoplastic therapy will undergo subsequent tumor imaging as scheduled until disease progression or start of new antineoplastic therapy or withdrawal from the study.</td></tr>
<tr><td>Concomitant medications</td><td colspan="2">×</td><td></td><td>Additional concomitant medications and treatment measures will be collected after initiation of study treatment.</td></tr>
<tr><td>AE collection</td><td colspan="2">×</td><td></td><td>AEs will be collected up to 30 days after the last dose.</td></tr>
<tr><td>SAE collection</td><td colspan="2">×</td><td></td><td>SAEs will be collected up to 30 days after the last dose.</td></tr>
<tr><td>Subsequent disease and survival information collection</td><td>×</td><td>×</td><td>×</td><td>Required for Phase I and Phase II, including information on disease progression, subsequent antineoplastic therapy, and survival.</td></tr>
</table>

* If no safety visit is performed, disease/survival follow-up was every 8 weeks after confirmed discontinuation of study treatment

CONTENTS

SPONSOR SIGNATURE PAGE *2*

Investigator Signature Page *3*

Synopsis *4*

Table 1. Study Flow Chart: Phase Ia Study - Treatment Phase *18*

Table 2 Study Flow Chart: Phase Ib and Phase II Studies – Treatment Phase *22*

Table 3 Study Flow Chart: Phase I and Phase II Studies – End of Treatment and Follow-up *27*

CONTENTS *29*

List of Tables *32*

List of Figures *33*

Abbreviations *34*

1 Study Background *37*

1.1. Introduction to biological mechanism of KRAS and KRAS G12C mutation **37**

1.2. Introduction of KRAS G12C mutated tumors **37**

1.3. Investigational drug **41**

1.4. Risk/Benefit Assessment **45**

2 Study Objective and Endpoints *47*

3 Study Design *49*

3.1. Overall design **49**

3.2. Phase Ia part: Dose escalation and dose expansion study of GFH925 alone in patients with advanced solid tumors **50**

3.3. Phase Ib Part **55**

3.4. Phase II part: Efficacy study of GFH925 alone in patients with advanced NSCLC ... **56**

3.5. Subject Study Procedures **56**

3.6. Study Design Rationale **58**

3.7. Definition of End of Study **60**

3.8. Clinical Criteria for Study Discontinuation/Early Termination **60**

4 Study Population *62*

4.1. Criteria for inclusion **62**

4.2. Exclusion Criteria **64**

4.3. Study Restrictions and Considerations ... 66
4.4. Subject Screening ... 67
4.5. Discontinuation and Withdrawal of Subjects from the Study ... 68
4.6. Lost to follow-up ... 69
5 Study and Concomitant Treatments ... 70
5.1 Treatment Regimen ... 70
5.2 Investigational drug ... 73
5.3 Dose Modification ... 74
5.4 Continued after disease progression ... 79
5.5 Prior and Concomitant Therapy ... 80
5.6 Treatment Compliance ... 82
5.7 Drug Product Management ... 82
5.8 Documentation of Study Drug ... 83
5.9 Complaint Handling ... 83
6 Study Assessments and Procedures ... 83
6.1. Safety Assessments ... 83
6.2. Pharmacokinetic Evaluation ... 86
6.3. Efficacy Evaluation Value ... 89
6.4. Biomarkers and Mechanisms of Resistance Exploration ... 90
6.5. Storage and Destruction of Biological Samples ... 92
6.6. Other Procedures ... 92
7 Safety Reporting and Adverse Event Management ... 93
7.1. Definition of Adverse Events ... 93
7.2. Definition of Serious Adverse Events ... 93
7.3. Assessment of Severity of Adverse Events ... 94
7.4. Judgment of Causal Relationship between Adverse Events and Investigational Drug 95
7.5. Recording of Adverse Events ... 95
7.6. SAE, Pregnancy and Abnormal Hepatic Function Event Reporting ... 99
8 Statistical Test ... 101
8.1. Statistical Analysis Plan ... 101

8.2. Statistical Hypothesis Testing and Sample Size Calculation....101
8.3. Statistics Analysis Populations....103
8.4. Statistical Analysis Methods....104
8.5. Measures to Control Bias....110
9 Research Quality Assurance and Quality Control....110
9.1. Clinical Monitoring....110
9.2. Data Management....111
9.3. Quality Assurance Audits....112
10 Ethics....113
10.1. Ethics Committee....113
10.2. Performed In Study Ethics....113
10.3. Subject Information and Informed Consent....114
10.4. Data Protection....114
10.5. Protocol Deviations....115
11 Study Management....115
11.1. Data Processing and Record Keeping....115
11.2. Raw Data/Document Access....115
11.3. Protocol Amendment....116
11.4. Investigator Responsibilities....116
11.5. Publication Policy....116
11.6. Finance and Insurance....117
12 References....118
13 APPENDICES....121
Appendix 1 Protocol Revision History....121
Appendix 2 Response Evaluation Criteria in Solid Tumors Version 1.1 (RECIST V1.1)....122
Appendix 3 Performance Status Criteria (ECOG PS)....134
Appendix 4 Statistical Performance of Bayesian Optimal Interval (BOIN) Design....135
Appendix 5 Cockcroft-Gault Formula....139
Appendix 6 List of Concomitant Therapies Prohibited Agents....140
Appendix 7 Statistical Methods for Interim Analysis....142

List of Tables

TABLE 1. STUDY FLOW CHART: PHASE IA STUDY - TREATMENT PHASE 18
TABLE 2 STUDY FLOW CHART: PHASE IB PHASE II STUDIES – TREATMENT PHASE 22
TABLE 3 STUDY FLOW CHART: PHASE I AND PHASE II STUDIES – END OF TREATMENT AND FOLLOW-UP....27
TABLE 4 DOSE ESCALATION 51
TABLE 5 BOIN DESIGN DOSE RISE AND RISE DECISION TABLE (TARGET TOXICITY RATE = 0.3)....51
TABLE 6 STUDY TREATMENT REGIMEN 71
TABLE 7 STUDY DRUG DOSE LEVEL 75
TABLE 8 DOSE MODIFICATION PRINCIPLES FOR HEMATOLOGIC TOXICITIES....75
TABLE 9 DOSE MODIFICATION PRINCIPLES FOR NON-HEMATOLOGIC TOXICITIES 77
TABLE 10 LIST OF LABORATORY TESTS....84
TABLE 11 PHARMACOKINETIC PLASMA SAMPLE COLLECTION SCHEDULE (IA DOSE ESCALATION PERIOD - QD/BID) 86
TABLE 12 PHARMACOKINETIC PLASMA SAMPLE COLLECTION SCHEDULE (IA DOSE EXPANSION PERIOD - QD/BID) 84
TABLE 13 SCHEDULE FOR PHARMACOKINETIC PLASMA SAMPLE COLLECTION (PHASE IB AND PHASE II STUDY PARTS)....86
TABLE 14 BIOMARKER SAMPLE COLLECTION AND ANALYSIS....92
TABLE 15 CRITERIA FOR JUDGING THE SEVERITY OF ADVERSE EVENTS....94
TABLE 16 COLLECTION OF AE/SAE/PREGNANCY EVENTS....96
TABLE 17 CRITERIA FOR JUDGING ABNORMAL LIVER FUNCTION TEST....100
TABLE 18 ORR OF SECOND-LINE AND THIRD-LINE MONOTHERAPY FOR NON-SMALL CELL LUNG CANCER 102

List of Figures

FIGURE 1 OVERALL STUDY DESIGN 50
FIGURE 2 SCHEMATIC DIAGRAM OF SUBJECT STUDY FLOW 57

Abbreviations

Abbreviation	English full name
AE	Adverse Event
ALT	Alanine Aminotransferase
ANC	Absolute Neutrophil Count
APTT	Activated Partial Thromboplastin Time
AST	Aspartate Aminotransferase
AUC	Area under the curve
BID, bid	Bis in die
BOIN	Bayesian Optimal Interval
BOR	Best of Response
C_{max}	Maximal concentration
C_{trough}	Trough concentration
CI	Confidence Interval
CL	Clearance
CL_r	Renal Clearance
CNS	Central Nervous System
Cr	Creatinine
CR	Complete Response
CRC	Colorectal Cancer
CRO	Contract Research Organization
CSCO	Chinese Society of Clinical Oncology
CT	Computed Tomography
CTCAE	Common Terminology Criteria for Adverse Events
CYPs	Cytochrome P450 Proteins
D	Day
DCR	Disease Control Rate
DDS	Dose Determination Set
DLT	Dose-Limiting Toxicity
DNA	Deoxyribonucleic Acid
DoR	Duration of Response
DRF	Dose Range Finding
ECG	Electrocardiogram
ECOG	Eastern Cooperative Oncology Group
EDC	Electronic Data Capture
EGFR	Epidermal Growth Factor Receptor
ECRF	Electronic Case Report Form
FAS	Full Analysis Set
FDG-PET	Fluorodeoxyglucose Positron Emission Tomography
G12C	Gly12Cys
GAP	GTPase-Activating Protein
GCP	Good Clinical Practice

Abbreviation	English full name
GDP	Guanosine Diphosphate
GEF	Guanine Nucleotide Exchange Factor
GGT	Gamma-Glutamyl Transpeptidase
GTP	Guanosine Triphosphate
GLP	Good Laboratory Practice
GPCR	G Protein-Coupled Receptor
HBV	Hepatitis B Virus
HCV	Hepatitis C Virus
HIV	Human Immunodeficiency Virus
HNSTD	Highest Non-Severely Toxic Dose
HR	Hazard Ratio
ICH-GCP	International Council for Harmonization
INR	International Normalized Ratio
IrAE	Immune-related Adverse Events
IRRC	Independent Radiological Review Committee
LLOQ	Lower Limit of Quantification
K^{+}	Serum Potassium
KRAS	Kirsten Rat Sarcoma Viral Oncogene
MAPK	Mitogen-Activated Protein Kinase
MedDRA	Medical Dictionary for Regulatory Activities
MRI	Magnetic Resonance Imaging
MRSD	Maximal Recommended Starting Dose
MSI-H	High Microsatellite Instability
MTD	Maximum Tolerated Dose
NOAEL	No-Observed-Adverse-Effect Level
NSCLC	Non-Small Cell Lung Cancer
ORR	Objective Response Rate
OS	Overall Survival
PD	Progression Disease
PFS	Progression Free Survival
P-gp	P-glucoprotein
PLT	Blood Platelet
PK	Pharmacokinetic
PKS	Pharmacokinetic Set
PN	Preferred name
PO, po, p.o.	Oral Administration
PPoS	Predictive Probability of Success
PPS	Per Protocol Set
PR	Partial Response
PRO	Protein in Urine
PS	Physical performance

Abbreviation	English full name
PT	Preferred Term
PT	Prothrombin Time
QD, qd	Quaque die
RECIST	Response Evaluation Criteria in Solid Tumors
RP2D	Recommended Phase II dose
SAE	Serious Adverse Event
SAP	Statistical Analysis Plan
SD	Stable Disease
SOC	System Organ Class
SS	Safety Set
STD10	Severely Toxic Dose in 10% of the Animals
$T_{1/2}$	Elimination half-life
T_{max}	Time to reach maximum concentration
TEAEs	Treatment Emergent Adverse Event
TRAE	Treatment Related Adverse Event
T-BIL	Total Bilirubin
TTP	Time to Progression
TTR	Time to Response
ULN	Upper Limits of Normal
V_d	Volume of distribution

1 Study Background

1.1. Introduction to biological mechanism of KRAS and KRAS G12C mutation

The murine sarcoma viral oncogene homolog (RAS) gene is a proto-oncogene that contains isoforms of KRAS, NRAS, and HRAS. RAS mutations are the most common mutations in tumors and involve various cancer types, such as colorectal cancer, pancreatic cancer, lung cancer, melanoma, and some hematological tumors. KRAS mutations occur predominantly in lung adenocarcinoma (approximately 25% of patients), pancreatic cancer (approximately 95% of patients), and colorectal cancer (approximately 35% of patients). Most KRAS gene mutations are point mutations, and the main mutation sites are amino acids 12, 13 and 61, with mutations at amino acid 12 (amino acid G to D/C/V/R/A/S) being the most common[1] [2] .

KRAS is a small GTP-hydrolase with a molecular weight of approximately 21 KD located on the inner side of the cell membrane, downstream of the epidermal growth factor receptor (EGFR) family [3][4]. When receptors such as EGFR on the cell membrane form dimers, phosphorylation of dimers can promote the formation of Grb2-Shc-SOS complexes, thereby activating the guanine nucleotide exchange factor (GEF) protein SOS to recruit non-activated KRAS that binds guanosine diphosphate (GDP), converting it into activated KRAS that binds guanosine triphosphate (GTP), and then activating the mitogen-activated protein kinase (MAPK) pathway, PI3K signaling pathway, RALGDS – RAL signaling pathway, etc., these signaling pathways play an important role in promoting cell survival and proliferation. The balance of KRAS and GDP/GTP binding is regulated by GEF and GTPase activating protein (GAP)[5] [6], GEF catalyzes the displacement of bound GDP to GTP on KRAS, and GAP can promote hydrolysis of KRAS-bound GTP to GDP. Mutations in KRAS, containing a glycine to cysteine substitution (G12C) mutation at position 12, break the normal balance of GDP/GTP binding, reduce KRAS binding to GAP, promote KRAS in a GTP-bound hyperactivated state, and then promote tumor development and growth. At the same time, KRAS gene mutation can also have an impact on the tumor microenvironment, inhibit anti-tumor immune response, and play a role in promoting tumors[7] .

1.2. Introduction of KRAS G12C mutated tumors

1.2.1 KRAS G12C mutated tumor type

According to literature [2], KRAS G12C mutations occur in approximately 13% of

lung adenocarcinoma patients worldwide, and are carried in a low proportion of patients with other solid tumors, such as colorectal cancer in approximately 3%, uterine cancer in approximately 2%, mesothelioma in approximately 1%, and pancreatic cancer in < 1%.

In Chinese patients, the results of 2 large-sample studies in Chinese patients published in 2020 suggest that KRAS G12C mutation frequency is slightly lower than in Western populations. A retrospective study of 11,951 tumor samples from Chinese patients showed that KRAS G12C mutations were common in non-small cell lung cancer (NSCLC) (4.3%), colorectal cancer (CRC) (2.5%), and other digestive tract tumors (cholangiocarcinoma 2.3% , small intestinal cancer 1.4% , pancreatic cancer 0.9% , etc.)[8] . Another retrospective study including 40, 804 Chinese NSCLC patients showed that the incidence of KRAS mutation was 9.8% in all patients, 29.5% of them were G12C mutations, i.e. the incidence of KRAS G12C in 40804 Chinese NSCLC patients was 2.89%[9] .

In summary, the predominant tumor types harboring KRAS G12C mutations are non-small cell lung cancer and colorectal cancer.

According to the latest global cancer burden data released by the World Health Organization International Agency for Research on Cancer (IARC) for 2020, there were 4.57 million new cancer cases and 3 million cancer deaths in China in 2020. Among them, there were 815,563 new cases of lung cancer and 714,699 deaths; 555,477 new cases of colorectal cancer and 28,6162 deaths. Lung cancer is the first new cancer type and cause of death in Chinese men; the second new cancer type and the first cause of death in Chinese women. Colorectal cancer is the third most common type of new cancer in both men and women in China, and the causes of death are the fifth and second, respectively.

1.2.2 KRAS G12C mutated advanced NSCLC and digestive tract cancer treatment status

- **Advanced NSCLC**

Treatment of advanced NSCLC varies according to the presence or absence of driver mutations. If EGFR, ALK, ROS and other driver genes are positive, the corresponding targeted drugs or platinum-based doublet chemotherapy and chemotherapy combined with anti-vascular therapy can be selected. KRAS mutations are rarely co-present with these driver mutations, so patients with advanced NSCLC with KRAS G12C mutations often do not benefit from marketed multiple targeted agents for these mutations or

rearrangements, with similar treatments as patients with "driver gene-negative NSCLC". CSCO Guidelines for the Diagnosis and Treatment of Common Malignant Tumors 2020-Guidelines for the Diagnosis and Treatment of Non-Small Cell Lung Cancer recommends NSCLC treatment options as:

- For first-line treatment, if PS 0-1, platinum-based doublet chemotherapy regimen, bevacizumab combined with platinum-based doublet chemotherapy, pembrolizumab alone, and pembrolizumab in combination with pemetrexed and platinum can be selected. In case of PS 2, single agent chemotherapy was used.

- Second line treatment, if PS 0-2, nivolumab or docetaxel or pemetrexed (if not receiving the same drug in the first line).

- Third-line treatment, if PS 0-2, nivolumab or docetaxel or pemetrexed (if not previously treated with the same drug), anlotinib (after failure of 2 chemotherapy regimens).

In second-line and above treatment regimens, the results of multiple large phase III clinical trials and clinical practice showed ORR between 8% and 22.9%; PFS for chemotherapy and chemotherapy plus anti-vascular drugs was 2.8 ~ 4.2 months and 4.8 ~ 5.4 months, respectively; OS ranged from 6 to 11.4 months and 9.9 to 12.6 months, respectively (see Section 1.7.1.8.2.2 for details). Given the potential for increased toxicity with combination therapy, doublet chemotherapy, as well as other combinations, requires adequate assessment of patient tolerability in second and further lines of therapy.

Data on immune checkpoint inhibitors in KRAS mutated NSCLC patients came mainly from subgroup analysis reports of large phase III clinical studies, in which CheckMate057 and OAK studies reported that KRAS mutated patients were able to achieve better overall survival (OS) benefit from nivolumab or atezolizumab treatment compared with docetaxel alone, respectively (HR = 0.52 , Fig. 95% CI 0.29-0.95 and HR = 0.71 , 95% CI 0.38-1.35)[10][11] .

No clear conclusion has been reached on the prognostic value of the KRAS G12C mutation in patients treated for advanced NSCLC. A retrospective study of 1,456 patients with advanced NSCLC from Guangdong Lung Cancer Institute showed OS of 18.3 and 26.7 months in patients with KRAS G12C mutation and wild-type patients, respectively, and KRAS G12C mutation suggests a potentially worse prognosis[9] .

With the exception of Sotorasib which has received accelerated FDA approval for marketing for the treatment of patients with locally advanced or metastatic non-small cell lung cancer harboring KRAS G12C mutations who have received at least one prior systemic therapy, most of the current product development for the KRAS G12C target remains in preclinical or clinical research, and clinical trials of MEK1/2 or CDK4/6 inhibitors of downstream signaling pathways in KRAS mutations have not been successful[12] .

- **Advanced CRC**

Treatment of advanced CRC also varies according to the presence or absence of mutations in RAS, BRAF, HER2 and other genes. When either RAS or BRAF is wild-type, the treatment used is monotherapy or multiagent chemotherapy in combination with cetuximab (left-sided lesion) or bevacizumab (right-sided).

For patients with KRAS mutated colorectal cancer, the second-line standard treatment is FOLFOX regimen (oxaliplatin + 5- fluorouracil + leucovorin) or FOLFIRI regimen (irinotecan + fluorouracil + leucovorin), with or without anti-vascular VEGF monoclonal antibody (bevacizumab is generally used for patients with KRAS mutation), and the overall ORR is around 6% , PFS ranged from 5.3 to 7.7 months, OS ranged from 11.2 to 14.1 months[13][17] . Regorafenib, fruquintinib and TAS-102 are approved in China for the selection of second-line treatment after progression, but the benefit of these regimens is not satisfactory, ORR 1 ~ 4%, PFS 2 ~ 3 months, OS 6 ~ 9 months according to the package inserts.

Although immunotherapy has been approved for the treatment of CRC patients with MSI-H , the proportion of patients with MSI-H in CRC is low, accounting for only about 5% to 15%[18] -[20] , and there remains a huge unmet clinical need.

Compared with CRC, other gastrointestinal tumors (cholangiocarcinoma, gastric cancer, pancreatic cancer, etc.) have more limited treatment options, and the main treatment options are still single-agent chemotherapy or multi-agent chemotherapy. In addition, regorafenib in cholangiocarcinoma achieved an advantage in PFS compared with chemotherapy in the REACHIN study, Pemazyre could be used for FGFR gene fusion or rearrangement, and immunotherapy against MSI-H/dMMR and targeted therapy against NTRK were still in clinical research; trastuzumab could be selected in combination with chemotherapy if HER2 was positive in gastric cancer, otherwise anti-

vascular therapy could be selected, and nivolumab was also approved for third-line treatment; Tarceva in pancreatic cancer are scarcely used due to limited efficacy, c olaparib could be selected if there was a BRCA mutation. Compared with other solid tumors, more treatments for digestive tract tumors other than chemotherapy are still under clinical study, so there are a large number of unmet clinical needs.

1.3. Investigational drug

1.3.1. GFH925

1.3.1.1 Drug development targeting KRAS G12C mutation development

Drug development for the KRAS G12C mutation has seen a breakthrough in recent years, combining with the mutation of GDP 's KRAS protein 12 position to cysteine to reveal a pocket of SWITCH II, which small molecules can occupy and covalently modify with this cysteine. This covalent modification hinders GEF -catalyzed GDP-GTP turnover, locking KRAS G12C in the inactivated state[21][23] . Based on this mechanism of action, there are currently multiple covalent inhibitors targeting KRAS G12C small molecules in clinical trials, including sotorasib/AMG510 , Adagrasib/MRTX849 , JNJ-74699157/ARS-3248 , GDC-6036 , D-1553 , and JDQ443 , Indications are solid tumors with KRAS G12C mutations; Sotorasib (AMG-510 , trade name: Lumakras) has received FDA accelerated approval for marketing as the first and only targeted therapy approved for patients with locally advanced or metastatic NSCLC with KRAS G12C mutations who have received at least one prior systemic therapy.

Efficacy data for the fastest progressing products Sorotasib and Adagrasib in NSCLC and C RC are summarized below and compared to the aforementioned data for efficacy determination.

Product name	Indications	Study phase	Sample Size	ORR	DoR (Months)	PFS (Months)
Sotorasib	NSCLC	Phase II	126 [i]	37.1%	10.0	6.8
	CRC	Stage I	42 [ii]	7.1%	-	4.0
Adagrasib	NSCLC	Phase I/II	51 [iii]	45%	-	-
	CRC	Phase I/II	18 [iii]	17%	-	-

Data sources: i. 2020 World Conference on Lung Cancer (WCLC); ii. 2020 European Society for Medical Oncology Asian Congress (ESMO Asia Congress); iii. 2020 International Symposium on Molecular Targets and Cancer Therapeutics (EORTC-NCI-AACR).

1.3.1.2 GFH925 Preclinical Studies

GFH925 is a KRAS G12C inhibitor developed by the sponsor sponsor and has undergone comprehensive preclinical studies, which are summarized below. For further details please refer to the GFH925 Investigator 's Brochure.

[redacted]

[redacted]

[redacted]

[redacted]

[redacted]

[redacted]

[redacted]

[redacted]

[redacted]

[redacted]

[redacted]

[redacted]

[redacted]

[redacted],

[redacted]

[redacted]

[redacted]

[redacted]

[redacted]

[redacted]

[redacted]

[redacted]

[redacted]

[redacted]

[redacted]

[redacted]

[redacted]

[redacted]

[redacted]

[redacted]

[redacted]

In conclusion, this study was designed with a close safety monitoring and may be expected to provide benefit to patients in the premise of subject safety, and the overall benefit/risk assessment supports the conduct of this study.

Further details on the potential benefits and risks of GFH925 can be found in the GFH925 Investigator 's Brochure and Development Safety Updated Report. Unexpected adverse reactions are unknown at this time. In the event of any discomfort, or new changes in the subject 's condition, or any unexpected circumstances during the study, whether or not related to the drug, the investigator should make prompt judgment and medical treatment.

2 Study Objective and Endpoints

This study is a Phase I/II study.

The objectives and endpoints of the Phase Ia include:

Study objectives	Study Endpoints
**Primary objective**<br>• To evaluate the safety/tolerability of GFH925 in patients with KRAS G12C-mutated advanced solid tumors<br>• To estimate the maximum tolerated dose (MTD) and/or recommended Phase II dose (RP2D) of GFH925	**Primary Endpoint**<br>• Incidence and severity of adverse events (AEs) and serious adverse events (SAEs); changes in laboratory tests, vital signs, physical examinations, and electrocardiograms (ECGs);<br>• Incidence of dose-limiting toxicity (DLT) events.
**Secondary objectives**<br>• To evaluate the pharmacokinetic (PK) profile of GFH925 in patients with KRAS G12C mutated advanced solid tumors;<br>• To preliminarily evaluate the efficacy of GFH925 in patients with advanced solid tumors with KRAS G12C mutation.	**Secondary Endpoints**<br>• The PK parameters of GFH925 included but not limited to: C_{max}, T_{max}, AUC, $t_{1/2}$, CL/F and Vd/F;<br>• Objective response rate (ORR) per Response Evaluation Criteria in Solid Tumors (RECIST) 1.1, disease control rate (DCR), duration of response (DoR), time to response (TTR), progression-free survival (PFS)<br>• ;<br>• Overall survival (OS).
**Exploratory objectives**<br>• To explore potential mechanisms of primary and secondary resistance to GFH925.	**Exploratory Endpoints**<br>• Correlation between efficacy and genetic mutation/gene fusion in tumor tissue and peripheral blood samples obtained at baseline and/or disease progression.

Objectives and endpoints for the Phase Ib portion of the study include:

Study objectives	Study Endpoints
**Primary objective**	**Primary Endpoint**
• To evaluate the efficacy of GFH925 in patients with KRAS G12C mutated advanced colorectal cancer or other solid tumor;	• ORR per RECIST 1.1;
**Secondary objectives**	**Secondary Endpoints**
• To evaluate the efficacy of GFH925 in patients with KRAS G12C mutated advanced colorectal cancer or other solid tumors using additional efficacy measures;<br>• To evaluate the safety of GFH925 in subjects with KRAS G12C-mutated advanced colorectal cancer or other solid tumors<br>• other solid tumors;<br>• To evaluate the PK profile of GFH925 in subjects with KRAS G12C- mutated advanced colorectal cancer or other solid tumors.	• DCR, DoR, TTR, PFS per RECIST 1.1, progression-free survival rate at 6 and 12 months;<br>• OS, survival at 12 months;<br>• Incidence and severity of AEs, SAEs, AEs leading to treatment interruption, and AEs leading to treatment discontinuation;<br>• Plasma concentration (including C_{trough}) after multiple dose administration in subjects.
**Exploratory objectives**	**Exploratory Endpoints**
• To explore potential mechanisms of primary and secondary resistance to GFH925.	• Correlation between efficacy and genetic mutation/gene fusion in tumor tissue and peripheral blood samples obtained at baseline and/or disease progression.

[redacted]

[redacted]	[redacted]
[redacted]	[redacted]
[redacted]	[redacted]
[redacted]	[redacted]

3 Study Design

3.1. Overall design

This study was an open-label, multicenter, Phase I/II clinical study to assess the safety/tolerability, pharmacokinetics and efficacy of GFH925 in patients with advanced solid tumors with KRAS G12C mutations.

The overall study consists of Phase I and Phase II Phase Ia parts of an open-label, multicenter dose escalation and dose expansion study in which eligible patients with KRAS G12C mutated advanced solid tumors will be enrolled to receive GFH925 monotherapy to assess the safety/tolerability of GFH925 in KRAS G12C mutated advanced solid tumors, MTD and/or RP2D of GFH925 were also determined. The Phase

Ib and Phase II parts are open-label, multicenter, single-arm studies in which Phase I b and II will enroll patients with KRAS G12C mutated advanced NSCLC , advanced colorectal cancer or other solid tumors to receive GFH925 monotherapy to assess the efficacy of GFH925 monotherapy in patients with KRAS G12C mutated advanced NSCLC , advanced colorectal cancer or other solid tumors, As well as safety and PK profile. Phase Ib and Phase II can be conducted simultaneously after the RP2D is determined.

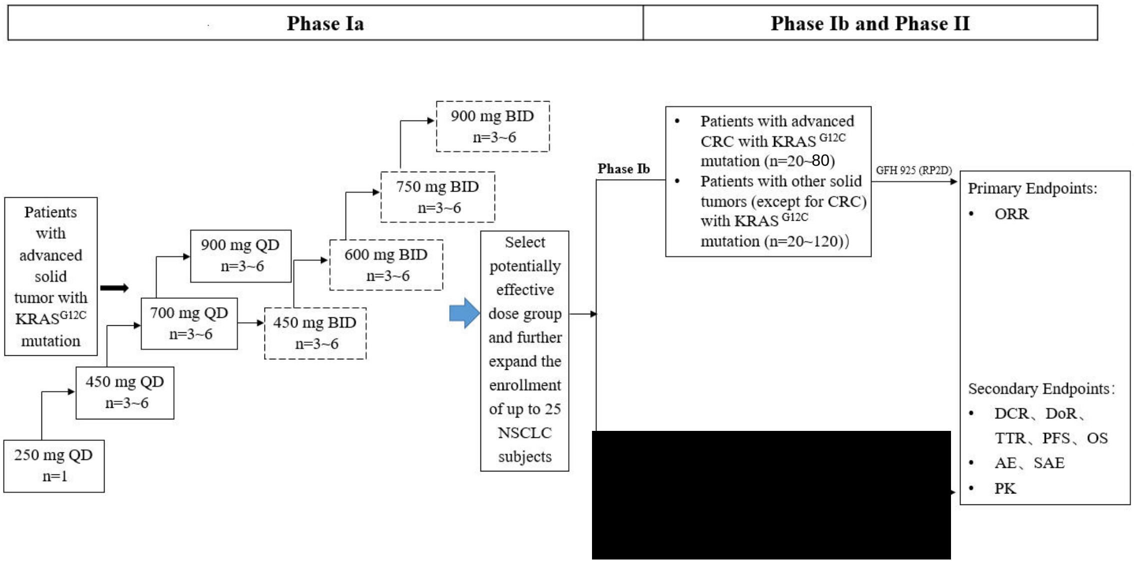

Figure 1 Overall Study Design

3.2. Phase Ia part: Dose escalation and dose expansion study of GFH925 alone in patients with advanced solid tumors

Phase Ia is an open-label, multicenter dose escalation and dose expansion study in which eligible patients with KRAS G12C mutated advanced solid tumors will be enrolled to receive GFH925 monotherapy to assess the safety tolerability/of GFH925 in KRAS G12C mutated advanced solid tumor patients and to determine the MTD and/or RP2D of GFH925 as well as to assess the pharmacokinetic profile.

Subjects will receive daily oral treatment with GFH925 at the designated dose after enrollment, and the first 21 days of treatment will serve as the DLT observation period. In the absence of a DLT event, subjects will continue to receive treatment and will be allowed to undergo dose escalation as permitted by this protocol and treatment will continue until disease progression or unacceptable toxicity or other conditions that lead to withdrawal or discontinuation.

During the dose escalation phase, 4 sequentially increasing QD dose levels (Cohorts

1-4): 250 mg QD, 450 mg QD, 700 mg QD, 900 mg QD, and 4 sequentially increasing BID dose levels (Cohorts 5-8): 450 mg BID, 600 mg BID, 750 mg BID, 900 mg BID are initially proposed. After completion of DLT observation and confirmation of safety data in the Cohort 3 (700 mg QD) dose group, dose escalation will be performed simultaneously in Cohort 4 (900 mg QD) and Cohort 5 (450 mg BID) (900 mg total) (see Table 4).

Adjustments to the dose regimen (dose and interval) will be made during dose escalation based on various factors including preclinical in vitro and in vivo activity, clinical PK, safety, efficacy data, and data from clinical trials with the same therapeutic target. The anticipated benefit of GFH925 will be assessed dynamically and comprehensively, which may lead to actions including but not limited to, cessation of dose escalation at the discretion of the sponsor and the investigator upon confirmation of drug absorption saturation.

Table 4 Dose Escalation

QD Cohort	Cohort 1	Cohort 2	Cohort 3	Cohort 4			
Dose	250 mg QD	450 mg QD	700 mg QD	900 mg QD			
BID Cohort				Cohort 5	Cohort 6	Cohort 7	Cohort 8
Dose				450 mg BID	600 mg BID	750 mg BID	900 mg BID

Dose expansion phase: The sponsor may select potentially effective dose groups based on PK, preliminary efficacy and safety data, and further expand the enrollment of up to 25 NSCLC subjects after completion of DLT observation and confirmation of safety.

3.2.1 DLT Definition

<u>Definition of DLT</u>

DLT is defined as an adverse event related to the study drug GFH925 occurring within 21 days of the first dose, and meeting the following severity criteria. Severity of adverse events are graded according to Common Terminology Criteria for Adverse Events (CTCAE) version 5.0.

Non-hematological toxicity:

- Vomiting or diarrhea ≥ Grade 4;
- Vomiting or diarrhea ≥ Grade 3, for more than 3 days despite optimal medical support;
- Nausea ≥ grade 3, for more than 3 days despite optimal medical support;

- Other ≥ Grade 3 non-hematologic adverse events.

Hematologic toxicity:

- Grade 4 neutrophil count (ANC) decreasing for more than 5 days; or Grade 3 febrile neutrophil count decreasing (ANC < 1.0 × 10^9/L with body temperature > 38.3℃); or neutrophil count decreasing with infection requiring clinical intervention;
- Grade 4 thrombocytopenia or Grade 3 thrombocytopenia with bleeding requiring clinical intervention;
- Grade 4 anemia.

In addition, in the event of any other treatment-related toxicity that may be considered as DLT, the investigators and the sponsor will decide jointly whether it is DLT or not after discussion; toxicities beyond the DLT observation period but meeting the above definition will also be important factors in confirming the safety of the current dose.

3.2.2 Definition of Maximum Tolerated Dose (MTD)/Recommended Phase II Dose (RP2D)

MTD is defined as the dose level at which the incidence of DLT is closest to the target toxicity rate (ie, 0.3) from the time the subject receives the first dose of study drug to Cycle 1. At the end of the dose escalation phase, isotonic regression analysis will be used to determine the MTD. Analysis was performed using online BOIN software at httpwww.trialdesign.org: the dose level estimated by isotonic regression analysis with the resulting probability of toxicity closest to the target toxicity rate (0.3) was the MTD. If there are 2 or more dose levels that meet both conditions, the higher dose level is selected when the estimated value is below the target toxicity rate, and the lower dose level is selected when the estimated value is greater than or equal to the target toxicity rate.

The RP2D will be determined based on all data obtained during the dose-escalation part, including safety, PK, biomarkers, and preliminary efficacy data, and a minimum of 6 subjects evaluable for safety is required for the RP2D.

3.2.3 Dose Escalation Level Design

Planned dose escalation arms were as follows:

**Dose Group**	**GFH925 Dose (mg)**	**GFH925 Total daily dose mg**	**Dose Escalation Magnitude (%)**
1	250 QD	250	-
2	450 QD	450	80

Dose Group	GFH925 Dose (mg)	GFH925 Total daily dose mg	Dose Escalation Magnitude (%)
3	700 QD	700	55.6
4	900 QD	900	28.6
5	450 BID		
6	600 BID	1200	33.3
7	750 BID	1500	25
8	900 BID	1800	20

In the conduct of the study, based on available safety/tolerability and pharmacokinetic data, it is possible to begin exploring other regimens at exposures for which safety has been established, such as increasing intermediate doses between adjacent dose levels as planned above.

3.2.4 Dose escalation rules

The dose escalation part will use accelerated titration combined with a Bayesian optimal interval (BOIN) design to determine the MTD.

Accelerated titration was employed at the first dose level and Bayesian optimal interval (BOIN) design was employed for subsequent dose levels. That is, if 1 subject is enrolled at the first dose level, if there is no adverse event related to the study drug with severity ≥ 2 during the DLT observation period (except for AEs meeting the definition of DLT), the study starts to enroll new subjects at the second dose level, and BOIN design is used for this group and subsequent dose escalation; otherwise, if the first subject has other adverse events related to the study drug with severity ≥ 2, the accelerated titration method is abandoned and directly converted to BOIN design.

BOIN design is easy to operate, similar to traditional 3 + 3 design, but has excellent statistical performance. Via BOIN online software (Fig. http://www.trialdesign.org) The results of 10,000 simulations showed that the BOIN design had a high probability of selecting the true MTD if the MTD existed and assigning more subjects to the dose level closest to the target toxicity rate (see Appendix 4). The target toxicity rate for the dose escalation part of this study was 0.3 for the maximum tolerated dose, with a planned maximum sample size of 45 patients. The MTD is defined as the dose level at which the incidence of DLTs is closest to the target toxicity rate (ie, 0.3) within 21 days of the subject 's first dose of study drug. Subjects were enrolled and treated at 3 to 6 per group.

As shown in the BOIN design flowchart (see Appendix 4 Figure A-1), the BOIN design uses the following optimization rules for dose escalation and descalation decisions:

- If the estimated DLT rate at the current dose is ≤ 0.236, raise the dose to the next higher dose level;
- If the estimated DLT rate at the current dose is ≥ 0.359, reduce the dose to an adjacent lower dose level;
- Otherwise, keep the current dose.

To prevent exposing subjects to an overdo(p_j >se, Pr 0.3 | observed data)> 0.95 (wherep_j is the true toxicity rate of dose j ,j = 1,2) and at least 3 subjects have been treated, dose levels j and higher doses were removed from subsequent trials and no longer used to treat patients. Here the posterior probability is estimated based on the beta-binomial model $y_j \mid p_j \sim \text{binomial}(p_j)$ and $p_j \sim \text{uniform}(0,1)$, y_j the number of subjects with DLT at dose level j. If the lowest dose was excluded, the trial was terminated early to ensure the safety of the subjects.

Specific steps for conducting a clinical trial using the BOIN design are as follows:

1. First group assigned to lowest dose level
2. Doses were assigned to the next group of subjects according to the dose escalation and reduction rules shown in Table . When using Table , pay attention to the following:
   a. "Removal" refers to removing the current dose and a higher dose from the trial. The excluded dose is an overdose and will not be used to treat any newly enrolled patients.
   b. Patients were treated with automatic dose reduction to the next lower level if the current dose was eliminated. If the lowest dose was excluded, the trial was terminated early to ensure subject safety. In this case, the MTD cannot be determined.
   c. If no decision (ie, dose increase, decrease, or elimination) was met, the current dose was continued for the next group of subjects.
   d. If the current dose is the lowest dose but dose reduction is still required by rules, new patients will still be treated at the current lowest dose. If the

number of subjects with DLT reached the exclusion boundary, the trial was terminated early to ensure the safety of the subjects.

e. If the current dose was the highest dose but a regular dose escalation was still required, the newly enrolled subject was still treated at the current highest dose.

3. Repeat step 2 until the set planned maximum sample size of 45 is reached or the number of subjects treated at the current dose is ⩾ 9 And the trial was terminated prematurely when the current decision was to maintain the current dose according toTable 's rise and fall rules.

Table 5 BOIN DESIGN DOSE RISE AND RISE DECISION TABLE (Target Toxicity Rate = 0.3)

Decision	Total number of evaluable subjects treated on current dose								
	1	2	3	4	5	6	7	8	9
Dose increased, if DLT Number of subjects ≤	0	0	0	0	1	1	1	1	2
Dose reduction, if DLT Number of subjects ≥	1	1	2	2	2	3	3	3	4
Dose eliminated if DLT Number of subjects ≥	NA	NA	3	3	4	4	5	5	5

3.3. Phase Ib Part

The Phase Ib part was an open-label, multicenter, single-arm study. Following achievement of the recommended Phase II dose (RP2D) in Phase Ia, 40 to 200 subjects with advanced colorectal cancer or other tumor types with KRAS G12C mutations will be enrolled and administered with GFH925 tablets at RP2D in 21-day cycles to evaluate the efficacy .

- CRC Cohort:20-80 patients with advanced colorectal cancer harboring KRAS G12C mutation were enrolled;
- Cohort for non-CRC solid tumors:20 to 120 patients with advanced tumors other than colorectal cancer harboring KRAS G12C mutation were enrolled.

[redacted]

[redacted]

[redacted]

[redacted]

3.5. Subject Study Procedures

The subject flow for both Phase I and Phase II studies can be divided into 3 periods.

1) Screening period: Day -28 to Day -1 before the first dose. After the subject signed the informed consent form, relevant information was provided and relevant examinations were performed according to the protocol requirements.

2) Treatment period: Following successful screening, subjects will receive GFH925 until disease progression confirmed by RECIST 1.1 criteria or intolerable toxicity or other conditions leading to withdrawal or discontinuation of treatment. Subjects were monitored regularly for safety during the study as planned; imaging examination was performed every 6 weeks ± 1 week for the first 48 weeks, and every 12 weeks ± 1 week thereafter at intervals, and efficacy was evaluated according to RECIST 1.1 by the investigator (Phase I and Phase II parts of the study) and IRRC (Phase II part); and pharmacokinetic and biomarker sampling were performed according to the protocol.

3) Follow-up period: Subjects will permanently discontinue treatment in the event of disease progression or intolerable toxicity or other conditions leading to withdrawal or discontinuation of treatment, and will undergo a safety follow-up visit (30 days after the last dose), AE collection until 30 days after the last dose, SAE collection until 30 days after the last dose, and survival follow-up (until death or loss to follow-up or end of study). Subjects who permanently discontinue treatment for reasons other than disease progression or initiation of other antineoplastic therapy will be followed for disease progression at intervals of every 6 weeks ± 1 week during the first 48 weeks of the study and every 12 weeks ± 1 weeks thereafter.

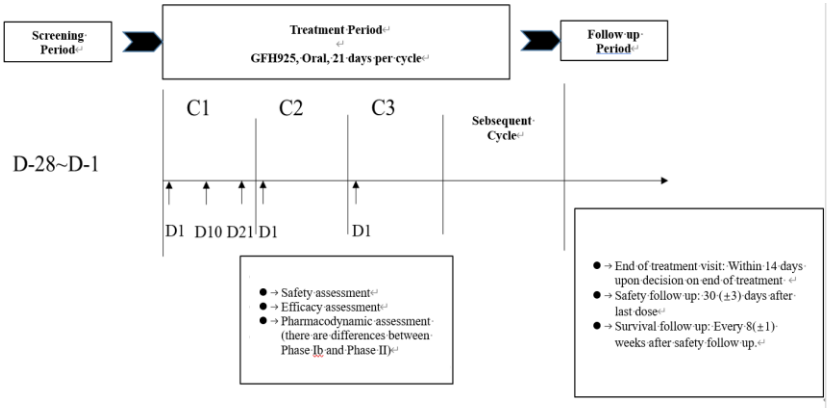

Figure 2 Schematic Diagram of Subject Study Flow

The study will end after the last subject has been treated for 2 years or after the end of treatment, whichever comes first.

At the time of the study, the above doses, dosing regimens, study procedures, blood sampling time points, etc. proposed in this protocol will likely be reconsidered and revised based on the latest available safety and PK/PD data.

3.6. Study Design Rationale

3.6.1 Justification for Starting Dose

The selection of the starting dose in Phase Ia was based on the results of preclinical toxicology studies and the starting dose was calculated according to the method of the maximum recommended starting dose (MRSD) in ICH S9.

[redacted]

[redacted]

In conclusion, considering the safety result in toxicology studies and anti-tumor activity in pharmacodynamic studies, in combination with the GFH925 tablet strength, 250 mg/day dose level was selected as the starting dose for the first-in-human trial of GFH925.

3.6.2 Selection basis of maximum escalation dose

[redacted]

3.6.3 Phase Ib and Phase II Dose Justification

RP2D (RP2D requires at least 6 safety evaluable subjects) will be determined based on a comprehensive consideration of information obtained from the Phase Ia study part, including safety, PK, and preliminary efficacy data, and this dose will be used to conduct Phase Ib Cohort A and Phase II studies. Safety-evaluable participants were defined as participants who met the minimum exposure requirement (defined as participants who completed 80% of the planned total dose in the first cycle) and were assessed DLT adequately or had DLT during the DLT observation period.

[redacted]

[redacted]

[redacted]

[redacted]

[redacted]

[redacted]

[redacted]

[redacted]

[redacted]

[redacted]

3.7. Definition of End of Study

The end of the study is defined as the last subject who completed treatment for 2 years or ended treatment, whichever comes first.

3.8. Clinical Criteria for Study Discontinuation/Early Termination

The study may also be terminated prematurely or suspended. This may be based on a regulatory authority decision, an ethics committee opinion, or a joint discussion between the sponsor and the investigator. In addition, the Sponsor reserves the right to discontinue the development of GFH925.

Reasons for early termination or suspension of the study may include:

- Identified unexpected, significant, or unacceptable risks to subjects;
- Available efficacy results support early termination of the study;
- Compliance with protocol requirements was low.

The party who decides to suspend/terminate the study will give written notice documenting the reason for study termination or suspension to the investigator, sponsor and regulatory authorities. If the study is prematurely terminated or suspended, the investigator should immediately inform the Ethics Committee and the sponsor and provide the relevant reasons.

The study will continue once the safety of the drug causing suspension, protocol

compliance and other issues mentioned above have been resolved and agreed by the sponsor, ethics committee or regulatory authority.

4 Study Population

Subjects must meet all inclusion criteria and none of the exclusion criteria.

Additional criteria for subject selection should not be used. Protocol deviations specific to inclusion and exclusion criteria are not allowed to be pre-approved. A maximum of one retest may be allowed when an abnormal test result due to laboratory testing error is considered.

4.1. Criteria for inclusion

Subjects participating in this study were required to meet all of the following criteria:

1. Voluntary participation and signed informed consent.
2. Age > 18 years at the time of informed consent.
3. Had histologically or cytologically confirmed advanced neoplasia and met the following requirements:
   - Phase I part: advanced NSCLC or other tumors, progression after standard treatment, or unsuitable for standard treatment due to intolerable toxicity, or refusal of standard treatment;

   [redacted]

4. Valid report documenting the presence of KRAS G12C mutation:

   • Phase I part: With documented KRAS G12C mutation and can provid qualified archival tumor tissue samples or undergo biopsy prior to treatment; if archival tissue samplesor biopsy samples is not available, enrollment is allowed based on other written materials approved by the investigator and the sponsor;

   [redacted]

5. Patients in Phase Ia and Phase II must have a measurable lesion according to RECIST 1.1 criteria. Phase Ib allows patients with at least one radiographically evaluable lesion to be enrolled.

6. Adequate organ function, including:

   - Adequate hematopoietic function, ie, absolute neutrophil count (ANC) ≥ 1.5 x 10^{9}/L, platelet count ≥ 75 x 10^{9}/L, hemoglobin ≥ 9 g/dL . Blood transfusion or granulocyte colony-stimulating factor, thrombopoietin, erythropoietin and other treatments should not be received within 14 days before blood routine examination.

   - Adequate liver function, ie, total bilirubin (TBIL) < 1.5 x upper limit of normal (ULN), aspartate transferase (AST), and alanine aminotransferase (ALT) < 2.5 x ULN; subject with Gilbert's syndrome must have total bilirubin < 2 × ULN; subject with liver metastases must have AST and ALT < 5.0 × ULN; TBIL < 3.0 × ULN is permitted if the subject's direct bilirubin (DBIL) suggests extrahepatic obstruction.

   - Adequate renal function, ie creatinine (Cr) ≤ 1.5 × ULN or creatinine clearance (CrCl) ≥ 60 calculated using the Cockcroft-Gault formul (see Appendix 5) when Cr > 1.5 × ULN mL/min.

   - Adequate coagulation function, ie, prothrombin time (PT) and activated partial thromboplastin time (APTT) < 1.5 × ULN, and international normalized ratio (INR) < 1.5 or within the target range for anticoagulant therapy.

7. Prior anticancer therapy toxicities must have recovered to baseline (except residual alopecia) or ≤ Grade 1 to be enrolled (neurotoxicity acceptable ≤ Grade 2). Patients with endocrine-related immune-related adverse events (irAEs) caused by previous immunotherapy, such as immune-related hypothyroidism, which are stable and asymptomatic after treatment, still require stable dose of hormone replacement or physiological dose of corticosteroids, and can be enrolled after the investigator evaluates that it does not affect the study drug administration and safety assessment.

8. Eastern Cooperative Oncology Group (ECOG) performance status (PS) 0 to 1.

9. Expected survival time ≥ 12 weeks.

10. Female or male subjects of childbearing potential agree to practice effective methods of contraception from signing of informed consent until 30 days after the last dose of GFH925. Female subjects of childbearing potential should have a negative blood pregnancy test within 7 days (inclusive) prior to dosing.

11. The investigator judged that the subject was able to communicate well, followed

up regularly, and completed the study according to the protocol.

[redacted]

4.2. Exclusion Criteria

Subjects who met any of the following criteria could not be enrolled in the study:

1. Significant cardiovascular disease, such as:
   - Definite cardiovascular abnormality within 6 months, such as myocardial infarction, angina pectoris, heart failure, severe arrhythmia, or angioplasty, vascular stent implantation, coronary artery bypass surgery;
   - Clinically significant QT/QTcF interval prolongation (QTcF > 470 ms in females or QTcF > 450 ms in males).
2. Subjects with unstable brain metastases diagnosed by investigators. Subjects with brain metastases incidentally discovered during screening may also be considered for enrollment if they are asymptomatic and do not require treatment; subjects with stable controlled brain metastases diagnosed by investigators with stable hormonal doses, (prednisone doses ≤ 10 mg/day or corresponding equivalent doses if other steroids are used) may be allowed to enroll.
3. Patients with significant gastrointestinal diseases, such as intractable hiccup, nausea, vomiting, severe gastrointestinal ulcers, liver cirrhosis, active gastrointestinal bleeding, or other diseases affecting swallowing tablets or significantly affecting the absorption of oral drugs; patients with liver cancer, such as Budd-Chiari syndrome or severe portal hypertension caused by portal emboli, also need to be excluded.
4. Presence of serious acute or chronic infections, including:
   - Active infection requiring systemic therapy;
   - Positive human immunodeficiency virus antibody (HIV-Ab) at baseline;
   - Active hepatitis B virus infection (hepatitis B surface antigen HBsAg positive, HBV-DNA positive); HBsAg negative, HBcAb positive, further examination HBV-DNA level is required. Exclude from enrollment if positive for HBV-DNA;
   - Active hepatitis C virus infection (hepatitis C antibody HCV-Ab positive and

HCV-RNA positive);

- Active pulmonary tuberculosis;

5. Pleural and peritoneal effusion or pericardial effusion with repeated drainage or significant symptoms.
6. Other uncontrolled systemic diseases even after standard treatment, such as uncontrolled hypertension (systolic blood pressure ≥160 mmHg or diastolic blood pressure ≥ 100 mmHg) and diabetes.
7. Other malignancies within 2 years prior to study entry, except appropriately treated cervical carcinoma in situ, focal cutaneous squamous cell carcinoma, basal cell carcinoma, prostate cancer not requiring treatment, ductal carcinoma in situ of the breast, and superficial non-muscle invasive urothelial carcinoma.
8. Current clinically significant interstitial lung disease requiring treatment, radiation pneumonitis, or drug-related pneumonia.
9. [redacted]
10. Prior treatment with KRAS G12C inhibitors.
11. Received therapeutic or palliative radiation therapy within 14 days prior to dosing in this study.
12. Chemotherapy, targeted therapy, endocrine therapy, immunotherapy, other investigational agents, or investigational device therapy within 28 days or 5 half-lives (whichever is shorter) prior to dosing in this study, except for maintenance endocrine therapy. Treatment with Chinese medicines with clear antitumor effect within 7 days prior to dosing in this study.
13. Surgical procedures (excluding needle biopsies) within 28 days prior to enrollment that could affect dosing or study assessments in this study.
14. Use of strong inhibitors or strong inducers of CYP3A4 or P-gp (see Appendix 6) within 14 days or 5 half-lives of the GFH925 (whichever is longer) prior to initiation of study treatment in this study or took traditional Chinese medicine within 7 days prior to initiation of study treatment in this study.
15. Received known CYP2D6 and CYP3A4 sensitive substrate within 14 days or 5 half-lives of the GFH925 (whichever is longer) prior to initiation of study treatment, and the substrate has a narrow therapeutic window; enrollment will not be allowed unless approved by the investigators and the sponsor (see Appendix 6).
16. Received known proton pump inhibitors and H2 receptor impedance agents within

7 days prior to dosing in this study (see Section 5.5.2.2).

17. Pregnant or lactating women.

18. Known allergy to the study drug or any component of its formulation.

19. Other conditions that, in the opinion of the investigator, would make participation in this study inappropriate.

4.3. Study Restrictions and Considerations

During this study, subjects were required to:

1. Grapefruit fruits and beverages (eg, red wine, citrus, grapes, or grape juice) were prohibited from at least 7 days prior to the first dose until the end of study treatment.
2. Alcohol was withheld for 24 hours prior to each dose until final PK and/or pharmacodynamic samples were collected.
3. Female subjects of childbearing potential, and male subjects with partners of childbearing potential, must use one of the following contraceptive measures from signing of informed consent until 30 days after the last dose of study treatment GFH925:
    - Abstinence (avoiding heterosexual intercourse).
    - Use (or have their partner use) reliable, effective methods of contraception during heterosexual intercourse such as:
        i. Single contraception (use any of the following)
            a) Intrauterine device
            b) Vasectomized male partner of female subject
            c) Subcutaneous implant contraception
        ii. Condom and oral contraceptives combined with contraception.

    Male subjects and partners with azoospermia (caused by vasectomy or other underlying disease) are not required to use contraception. Female subjects were considered to be of non-childbearing potential if:

    i. Postmenopausal (defined as no menses for at least 12 months without an alternative medical cause; testing follicle stimulating hormone (FSH) level alone is insufficient to confirm postmenopausal status in the absence of menses for 12 months);

ii. Or hysterectomy and/or bilateral oophorectomy, bilateral salpingectomy or bilateral tubal ligation/occlusion at least 6 weeks prior to screening;

iii. Or congenital or acquired conditions causing infertility.

4.4. Subject Screening

4.4.1. Inclusion Procedure

The investigator will enroll subjects as follows:

1. Obtain Informed Consent Form (ICF) signed by the subject or guardian prior to performing any study-related procedures.

2. The principal investigator or appropriately trained designee formally determined the subject 's eligibility after reviewing the inclusion/exclusion criteria.

Subjects who do not meet the criteria associated with this study (screen failure) may be rescreened. If re-screening of a subject is considered, the investigator must contact the sponsor 's medical responsible person. Each participant may be rescreened once with the agreement of the sponsor 's medical responsible person at rescreening. At re-screening, the subject must be re-consented and will be reassigned an identification number. Assessments performed during initial screening will remain acceptable without repeat testing if they remain within the study-defined timeframe and if the results meet the inclusion and exclusion criteria.

4.4.2. Procedures for Handling Incorrectly Enrolled Subjects

Inclusion and exclusion criteria must be strictly followed. If it is discovered that a subject who does not meet the inclusion and exclusion criteria is enrolled, the sponsor 's medical responsible and investigator need to discuss and determine whether to continue the subject' s participation in the study with or without the study drug. If the investigator considers it appropriate from a medical point of view to continue the subject 's participation in the study and the sponsor medical director agrees with the investigator' s decision, the subject may continue to participate in the study and receive treatment with the study drug. If the investigator considers it medically appropriate for the subject to continue participation in the study, but the sponsor medical director disagrees with the investigator 's decision, the subject should not continue participation in the study (whether

receiving or not receiving study drug). The investigator was not permitted to continue participation in the study for subjects who were inadvertently enrolled in the study until written approval was received from the sponsor.

4.5. Discontinuation and Withdrawal of Subjects from the Study

4.5.1. Subject Discontinued

Discontinuation of study treatment does not mean withdrawal from the study. Subjects who discontinue study treatment should complete the end-of-treatment visit and subsequent follow-up visits as required by the protocol.

Possible reasons for discontinuation of study treatment included:

1) If the subject develops disease progression requiring discontinuation of treatment, if the investigator judges that the subject is still benefiting from continued medication, the study treatment may be continued after discussion with the sponsor;
2) Subject develops intolerable toxicity;
3) Subject started other anti-tumor therapy;
4) The subject seriously fails to comply with the requirements of the study protocol;
5) Subject is pregnant;
6) Subject lost to follow-up;
7) Subject died;
8) The subject requests to discontinue treatment, but receives subsequent follow-up;
9) Subject withdrew consent;
10) Investigator believes it is in the best interest of the subject to discontinue study treatment;
11) End or discontinuation of the entire clinical study.

In case of the above, the end of treatment visit should be completed 14 days after the decision to discontinue the study treatment, safety follow-up should be completed, and adverse events and outcomes should be completely recorded; survival follow-up will be performed subsequently. Subjects who discontinue treatment for reasons other than disease progression will also be followed for disease progression at intervals of every 6

weeks ± 1 week during the first 48 weeks of the study and every 12 weeks ± 1 week thereafter until disease progression or start of new antineoplastic therapy, or withdrawal from the study (see Section 4.5.2). These visits were not applicable for subjects who were lost to follow-up, died, or withdrew consent.

The investigator may provide advice or alternative treatment to the subject based on his/her actual condition.

4.5.2. Subject Withdrawal

Subjects could voluntarily withdraw from the study at any time. Reasons for withdrawal may include:

1) Subject withdraws consent, refuses to perform study procedures, refuses to be contacted in the future, and provides information;
2) Subject died;
3) Subject lost to follow-up;
4) The entire study ends or discontinues.

If a subject fails to return for follow-up as agreed, every effort should be made to reach out to the subject in a timely manner and to reschedule the missed visit as soon as possible. The investigator should ask about the reason for withdrawal and ask the subject to return to the site as far as possible to complete the visit and follow up the unended adverse events. In any case, every effort should be made to document the subject 's outcome, whenever possible.

If a subject refuse to attend the study site for further visits, his/her disease and survival should continue to be followed up and collected unless the subject withdraws consent (ie, refuses to be contacted). In this case, no further study evaluations should be performed and no further data should be collected.

4.6. Lost to follow-up

Subjects who fail to return to the site for 2 consecutive scheduled visits and cannot be contacted by site staff will be considered lost to follow-up.

The following actions must be taken if a subject fails to return to the study site for a specified study visit:

- The site attempted to reach out to the subject, reschedule the missed visit,

explain the importance of following the visit schedule to the subject, and confirm whether or not the subject was willing and/or should continue in the study.

- Before a subject is deemed lost to follow-up, the investigator or designee will make every effort to regain contact with the subject (at least two attempts should be made to reach the subject by telephone, and if contact remains unavailable, a letter should be sent to the subject 's most recently updated contact address). These attempts to reach the subject should be documented in the subject 's medical record or study documentation. The investigator was to make every effort to reach out to the subject before the end of the study.

Subjects will be considered lost to follow-up if they remain unreachable. All attempts to reach out should be documented in the source documents.

5 Study and Concomitant Treatments

5.1 Treatment Regimen

Investigational product in the study is defined as GFH925. The treatment regimen investigated is shown in Table .

Table 6 Study Treatment Regimen

Cohort	Investigational drug	Dose/Dosage	Dose Frequency	Course/Treatment Cycle	Usage method	Remarks
Phase Ia	GFH925	250 mg	QD	Every 21 day cycle	Oral	The first cycle following the first treatment will serve as the observation period for dose limiting toxicity (DLT), in which PK sampling needs to be discontinued on Day 2 . In the absence of DLT events, subjects will remain on treatment and dose modifications will be allowed as permitted per this protocol until disease progression, death, or intolerable toxicity, or other conditions that lead to withdrawal or discontinuation from treatment. Subjects will undergo safety assessments, PK/pharmacodynamic (PD) sampling, and efficacy assessments during the study as required by the protocol.
	GFH925	450 mg	QD	Every 21 day cycle	Oral	
	GFH925	700 mg	QD	Every 21 day cycle	Oral	
	GFH925	900 mg	QD	Every 21 day cycle	Oral	
	GFH925	450 mg	BID	Every 21 day cycle	Oral	
	GFH925	600 mg	BID	Every 21 day cycle	Oral	
	GFH925	750 mg	BID	Every 21 day cycle	Oral	
	GFH925	900 mg	BID	Every 21 day cycle	Oral	

Cohort	Investigational drug	Dose/Dosage	Dose Frequency	Course/Treatment Cycle	Usage method	Remarks
Phase Ib	GFH925	RP2D	BID	Every 21 day cycle	Oral	Assessments such as tumor evaluations and safety tests were performed periodically as required by the protocol, and dose modifications were allowed per the protocol, with treatment continued until disease progression or intolerable toxicity or other conditions leading to withdrawal or treatment discontinuation.
[redacted]	[redacted]	[redacted]	[redacted]	[redacted]	[redacted]	[redacted]

5.2 Investigational drug

5.2.1 Description of GFH925 Tablets

Investigational drug	GFH925 Tablets
Active ingredient	GFH925
Excipients	Microcrystalline cellulose, Lactose Monohydrate, croscarmellose sodium, magnesium stearate
Type	Chemical drug
Dosage form	Tablet
Strength	[redacted]
Route of administration	Oral
Storage Requirements	Sealed at room temperature
Shelf Life	Tentative 24 months
Packaging and Labeling	Study drug will be provided to each subject in high-density polyethylene bottles. Each HDPE bottle will be labeled according to local regulatory requirements

5.2.2 Treatment assignment and drug dispensing

A subject number will be assigned at the time the subject signs the written informed consent form and will be used in all study documents and case report forms (CRFs). After completing the screening process, whether to formally enroll in the study was judged according to the inclusion and exclusion criteria. All enrolled subjects will receive study drug.

The investigator or designee must ensure that all medications or related items used for study treatment are maintained under appropriate temperature conditions during shipment. In case of any deviation, it is required to report and solve the problems before performing the study treatment.

Only subjects enrolled in the study may receive study treatment and only authorized site personnel may supply or manage study treatment products. All study intervention medications or related items must be stored in a secure, environmentally controlled, and monitorable (manual or automated) area in accordance with the storage

conditions indicated on the label and access to study treatment products must be restricted to the investigator and authorized site personnel.

5.2.3 Study Drug Use

5.2.3.1 GFH925 administrtion

Administered orally with approximately 240 mL warm water. Subjects must be informed of the condition that the tablets must be swallowed in a whole and not chewed or broken apart. Patients will be fasted at least two hours before each dose and remain fasted one-hour post dose. The administration time of each day should be close as far as possible. If a PK blood sample (pre-dose sample) is scheduled to be collected in the morning, patients should take GFH925 tablet after completing the predose sampling. Patients can take GFH925 tablets around 8:00 and 20:00 respectively every day when they were administered twice daily.

Patients enrolled in phase Ib should be fasted overnight (water is allowed) for at least 10 hours prior to the first doses planned on C1D0 and C1D21 and keep fasted for at least one-hour post first doses on those days. In dose expansion phase, if intensive blood sampling is scheduled to be collected, the fasting regimen for corresponding patients is the same as dose escalation phase.

If a missing dose occurs, it can be made up within 4 hours after the scheduled timepoints. In this case, the actual dosing time should be recorded, and the subsequent dosing timepoints will remain on schedule. If it has been more than 4 hours, the exact doseshould be skipped, and the patient should continue the subsequent dose treatment at the scheduled timepoints. If vomiting occurs after administration, re-dosing is not allowed before the next scheduled dose. All actual dosing timepoints are recorded in the subject diary or recorded as ‘missing doses’.

5.3 Dose Modification

5.3.1 General principle

The Phase Ia part of this study allowed intrasubject dose escalation. If a subject experience no DLT events or other adverse events ≥ Grade 2 in severity and related to the study drug during the treatment period, the investigator and the sponsor will review all data to determine whether the subject can enter the treatment at a higher dose level that has been confirmed as safe. Following dose escalation, subjects will be required to follow

the first cycle visit schedule, undergo close safety monitoring and PK sampling (without discontinuation), but tumor imaging should continue to follow the previous schedule.

Subjects should be closely monitored for toxicities following the use of study drug during the study, and best supportive care is recommended to manage toxicities according to their site practice. A maximum of 2 dose reductions were allowed for the same subject (Table). In exceptional circumstances, the investigator should reach agreement with the sponsor through further discussion. Refer to Table 8 and Table 9 for details of dose modifications.

Table 7 Study Drug Dose Level

**GFH925**	Original dose	450 mg QD	700 mg QD	900 mg QD	450 mg BID	600 mg BID
	-1 dose	300 mg QD	600 mg QD	750 mg QD	750 mg QD	450 mg BID
	-2 Dose	-	450 mg QD	600 mg QD	600 mg QD	750 mg QD
Cetuximab	Original dose	500 mg/m ^{2}				
	-1 dose	250 mg/m ^{2}				
	-2 Dose	200 mg/m ^{2}				
	-3 Doses	150 mg/m ^{2}				

5.3.2 Medication Modification with GFH925

5.3.2.1 Dose Hold and Permanent Discontinuation

Any subject who develops a condition during the study that is related to the study treatment and meets the protocol 3.2.1 Adverse events defined in Section DLT or meeting any of the discontinuation criteria in Table , Table 9 should stop GFH925 treatment and be followed until resolution or return to baseline. If the subject has already responded to oncologic therapy or the investigator judges continued treatment to be beneficial, study treatment may be resumed at the same dose or at a lower dose after full communication with the sponsor and the toxicities have recovered to the following levels while continuing close monitoring for associated toxicities. If treatment is restarted at the same dose level, patients should be closely monitored after restart. If the severity of an adverse event

related to the study treatment does not meet the criteria for DLT definition but meets any of the following criteria, suspension of GFH925 administration is required:

- Thrombocytopenia ≥ Grade 3
- Leukopenia ≥ Grade 3
- Neutropenia ≥ Grade 3
- Anemia ≥ Grade 3
- Non-hematological toxicities ≥ Grade 3

In case of the above hematological toxicities, blood routine should be repeated within 3 days, and recovery should continue to be closely monitored thereafter. Resumption of treatment may be considered if it recovers to the following criteria: non-hematological toxicities recover to ⩽ Grade 1 or baseline; hematological toxicities recover to ANC ⩾ 1.0×10^9/L, PLT ⩾ 75×10^9/L, Hb ⩾ 8 g/dL.

Permanent discontinuation of treatment should be considered if a subject 's adverse event remains unmanageable within 4 weeks or the same toxicity requires treatment interruption occurs again after resumption of the drug.

Permanent discontinuation of treatment should be considered in case of adverse events related to GFH925 treatment according to the following criteria unless agreed upon by the investigator and sponsor:

- ALT or AST > 8 × ULN;
- ALT or AST > 5 × ULN for more than 2 weeks;
- ALT or AST > 3 × ULN with (TBL > 2 × ULN or INR > 1.5);
- ALT or AST > 3 × ULN with signs and symptoms associated with hepatitis such as fatigue, nausea, vomiting, right upper quadrant pain or tenderness, fever, rash, and/or eosinophilia (> 5%).

Subsequent study procedures for permanent discontinuation are provided in Protocol 4.5.1.

Please refer to Table and Table 9 for detailed dose modification principles to reach agreement with the sponsor for further discussion in case of special circumstances. All dose modifications must be documented in the source documents and entered into the eCRF as required.

[redacted]

[redacted]	[redacted]	[redacted]
[redacted]	[redacted]	
[redacted] [redacted] [redacted]	[redacted]	[redacted]
	[redacted]	[redacted]
	[redacted] [redacted] [redacted] [redacted]	[redacted]
	[redacted] [redacted] [redacted]	[redacted]
[redacted]	[redacted]	
[redacted]	[redacted]	[redacted]
	[redacted]	[redacted]
[redacted]	[redacted]	
	[redacted]	[redacted]
	[redacted]	[redacted]
[redacted]	[redacted]	
	[redacted]	[redacted]
	[redacted]	[redacted] [redacted] [redacted]

[redacted]

[redacted]	[redacted]	[redacted]
[redacted]		
	[redacted]	[redacted]
	[redacted]	[redacted]
[redacted] [redacted]	[redacted]	
[redacted]	[redacted]	[redacted]
	[redacted]	[redacted]
[redacted]	[redacted]	
[redacted]	[redacted]	[redacted]
	[redacted]	[redacted]
[redacted]	[redacted]	[redacted]
	[redacted]	[redacted]
[redacted]	[redacted]	
[redacted]	[redacted]	[redacted]
	[redacted]	[redacted]
[redacted]	[redacted]	
	[redacted]	[redacted]
[redacted]		[redacted]

[redacted]	[redacted]	[redacted]
	[redacted]	[redacted]
	[redacted]	[redacted]
	[redacted]	[redacted]
[redacted]	[redacted]	
[redacted]	[redacted]	[redacted]
	[redacted]	[redacted]

5.4 Continued after disease progression

Following disease progression, if the investigator judges that the subject will continue to benefit, treatment with GFH925 will be allowed to continue with full informed consent until the criteria for treatment termination are met (see Section 4.5.1 of the protocol).

Continuation of medication beyond disease progression requires continued data collection at study designed visits.

5.5 Prior and Concomitant Therapy

5.5.1 Prior Therapy

Refer to Protocol section 4.2 for prior therapies prohibited prior to subject participation in this study.

After giving informed consent, subject's all prior lines of medication for the disease under study (advanced solid tumors) will be collected, as well as other medications 30 days prior to the first dose of study drug.

5.5.2 Concomitant Therapy

All concomitant therapies, blood products, non-pharmacological interventions (eg, punctures) received by the subject will be recorded from the start of the first study treatment through the Safety Follow-up Visit.

5.5.2.1 Permitted/Cautious Concomitant Therapy

During the study, the investigator should follow the principles below and carefully use concomitant drugs to maximize the safety of subjects.

Palliative care and best supportive care for disease symptoms were permitted during the study, but concomitant treatment during DLT observation should avoid the influence on judgment of DLT. Palliative and supportive care for disease-related symptoms will depend on the investigator 's judgment and relevant guidelines (eg, American Society of Clinical Oncology Guildlines). For example, palliative local radiation therapy for painful bone lesions with the intent of relieving symptoms is permitted provided that these lesions are known to be present at enrollment and are not the only target lesions. The investigator should determine and document whether the use of radiotherapy is associated with disease progression prior to performing radiotherapy.

During the study, if the subject is complicated with other diseases, the investigator should determine whether drug treatment is required, and avoid the drugs that have great influence on the judgment of study results as far as possible, so as to avoid affecting the judgment of safety and tolerability of the subject. In case of serious adverse reactions or serious adverse events, or deterioration of the original condition or concurrent other serious diseases, the investigator should promptly give concomitant treatment and active treatment. Subject withdrawal will be appropriately scheduled if criteria for withdrawal are met.

- If the investigator is unable to determine whether concomitant treatment impacts the safety of the subject, or whether the use of the drug impacts the judgment of the safety and tolerability of the subject, or whether the use of the drug impacts the eligibility of the subject to be enrolled and the evaluability of the data, it should be discussed and agreed with the sponsor before the use of concomitant treatment.

Subjects should cautiously take concomitant known proton pump inhibitors and H2-receptor antagonists during the study, including but not limited to omeprazole, lansoprazole, pantoprazole, rabeprazole, esomeprazole, ilaprazole, cimetidine, famotidine, nizatidine, roxatidine. If gastric acid reducing agents must be used, GFH925 may be administered 2 hours before or after treatment with known antacids, including hydrotalcite chewable tablets, aluminum phosphate gel, aluminum hydroxide, magnesium hydroxide, calcium carbonate and simethicone.

5.5.2.2 Prohibited concomitant therapy

Subjects should not receive the following treatments during the study:

1) Any other anti-tumor therapy (chemotherapy, immunotherapy, biological products, extensive radiotherapy, hormonal therapy, targeted therapy, surgery, traditional Chinese medicine with approved anti-tumor indications) other than the study treatment, including investigational therapy or approved therapy. Antagonism Patients taking gonadotropin-releasing hormone (GnRH) for prostate cancer, oral contraceptives, or hormone replacement therapy may continue to take this drug.

2) Traditional Chinese medicine (TCM) use was prohibited within 7 days prior to the first dose, and was not recommended until the final PK collection for the study (C3D1) was completed. The use of traditional Chinese medicines for the management of adverse events or diseases must be reviewed and approved by the sponsor before use.

3) Granulocyte colony-stimulating factor drugs were used as prophylaxis. Such drugs may be used for the treatment of adverse reactions only at the investigator 's discretion.

4) Any other drug under clinical investigation other than the study treatment.

5) From Two weeks or 5 half-lives (whichever is longer) prior to first dose until two weeks after last dose, known strong inhibitors and inducers of CYP3A4 and

P-gp are prohibited (see Appendix 6).

6) Known sensitive substrates of CYP2D6 and CYP3A4 (see Appendix 6) were prohibited within 2 weeks before the first dose of study drug or within 5 half-lives of the drug, whichever was longer, and for 2 weeks after the last dose unless the drug was reviewed and approved by the investigator and the sponsor 's medical director during the screening period.

5.6 Treatment Compliance

Subjects will return all untaken GFH925 tablets and packs from the previous cycle on the designated visit day following the investigator 's instructions and return the completed Medication Log. The quantity returned by the subject will be counted, recorded and filed, and the medication compliance will be calculated according to the actual medication.

5.7 Drug Product Management

5.7.1 Investigational Product Receipt and Accountability

Study drug will be provided by the sponsor according to the site 's anticipated enrollment plan. Study drug will be shipped to the site via a third-party logistics company qualified for shipment. Authorized site staff will sign the delivery note to confirm receipt.

The study drug should only be used in this study and managed only by a person authorized by the investigator. To fully control the dispensing and use of study drug, the quantity was registered at each visit.

5.7.2 STORAGE AND MANAGEMENT OF STUDY DRUG

The investigator or other authorized site personnel (eg, pharmacist) will ensure that all study drug is stored in a controlled access and secure area that meets the storage conditions described in the 5.2.1 section of the protocol and is stored in compliance with applicable regulatory requirements. Subjects receiving GFH925 will follow the instructions of the investigator or authorized personnel for proper storage. If non-compliant storage is identified, the investigator should contact the sponsor for guidance.

5.7.3 Study Drug Recovery and Destruction

Unused study drug and packaging will be recovered and destroyed after accountability by the sponsor sponsor or authorized for destruction by the site. The

sponsor or his/her authorized designee will provide guidance to the site on how to destroy unused study drug. If authorized for destruction at the site, the investigator ensured that all recoveries and destruction were performed and documented in accordance with applicable regulations, guidelines, and rules.

5.8 Documentation of Study Drug

The designated personnel of the study site should keep relevant records of the receipt, distribution, use, inventory, destruction, recovery and destruction of the study drugs in a timely manner according to the requirements of relevant regulations and guidelines as well as the operating procedures of this trial.

5.9 Complaint Handling

To ensure the safety of study participants and the quality of monitoring, and to assist with process and product improvements, the sponsor will collect product complaints related to the study drug used in the clinical trial.

Complaints associated with concomitant medications will be reported directly to the manufacturer in accordance with the product instructions.

The investigator or his/her designee is responsible for completing the following product complaint procedures as outlined in this study:

- Investigation-specific complaint forms were used to document the reported product complaints and associated full descriptions.
- Fax or email the completed Product Complaint Form to the Sponsor or its designee within 24 hours.

If the Investigator is asked to return the Product for investigation, he/she will return a copy of the Product Complaint Form together with the Product.

6 Study Assessments and Procedures

6.1. Safety Assessments

Safety evaluations included vital signs and physical examinations, laboratory tests including pregnancy tests, electrocardiograms, and collection of information on adverse events, serious adverse events, and concomitant therapy.

6.1.1. Laboratory Tests

Hematology, urinalysis, blood biochemistry, coagulation function and pregnancy test will be performed according to the study flow chart. Viral serology was also required during screening to assess eligibility Refer to Table 10 for Laboratory Tests.

If a subject had a laboratory test performed outside the study site and reported an associated AE, the laboratory test result was also collected and entered in the eCRF.

Table 10 List of Laboratory Tests

Inspection item	Outcome Measures
Blood routine	Red blood cell count (RBC), hemoglobin concentration (HGB), hematocrit (HCT), mean corpuscular volume (MCV), mean corpuscular hemoglobin content (MCH), mean corpuscular hemoglobin concentration (MCHC), white blood cell count (WBC), platelet count (PLT), neutrophil count (ANC), lymphocyte (LY) count, monocyte (MO) count, eosinophil (EO) count, basophil (BA) count, neutrophil percentage, lymphocyte percentage, monocyte percentage, eosinophil percentage, basophil percentage
Urine routine	Urinary leukocytes (LEU), urinary nitrite (NIT), urinary pH, specific gravity (SG), urinary protein (PRO), urinary glucose (GLU), urinary ketone body (KET), urobilinogen (UBG), urinary bilirubin (BIL), urinary occult blood (BLD); 24-hour urinary protein (if necessary)
Coagulation function	Prothrombin time (PT), activated partial thromboplastin time (APTT), international normalized ratio (INR)
Blood biochemistry	Aspartate aminotransferase (AST), alanine aminotransferase (ALT), alkaline phosphatase (ALP), lactate dehydrogenase (LDH), glutamyl transpeptidase (GGT), urea nitrogen (UREA)/urea nitrogen, serum creatinine (CREA), total protein (TP), albumin (ALB), total bilirubin (TBIL), direct bilirubin (DBIL), creatine kinase (CK), pancreatic amylase (AMY), fasting plasma glucose (FPG), total cholesterol (TCHO), low-density lipoprotein cholesterol (LDL-C), high-density lipoprotein cholesterol (HDL-C), triglycerides (TG), calcium, sodium, chloride, potassium
Viral Serology Testing	Hepatitis B surface antigen (HBsAg), hepatitis B surface antibody (HBsAb), hepatitis B core antibody (HBcAb), H BV surface antigen, HBV-DNA (if necessary), hepatitis C virus antibody (HCV antibody), HCV-RNA (if necessary), HIV antibody
Pregnancy test	Female subjects of childbearing potential will receive serum β -HCG pregnancy test at baseline and urine pregnancy test at the rest visits

6.1.2. Clinical Examination

6.1.2.1. Vital Signs and Physical Examination

Vital signs (respiration, pulse, blood pressure, and temperature) will be recorded, physical examinations including general examinations and physical examinations of disease-related systems will be performed, and ECOG scores will be performed if necessary. Perform a complete physical examination including general physical examination, skin and lymph nodes, head and neck, chest, abdomen, spine extremities, neurological system, genito-anal and rectal examination as necessary.

6.1.2.2. 12 Lead ECG

The 12 lead ECG will be completed according to the date and time requirements specified in the Study Flow Chart. 2 hours postdose and ± 30 minutes for 4 hours postdose.

Phase Ia Dose Escalation

Subjects will perform ECG examination on C1D1 (predose, 2 hours postdose, and 4 hours postdose), C1D3 (predose), C1D10 (predose), C1D21 (predose, 2 hours postdose, and 4 hours postdose), C2D1 (predose), C3D1 (predose), and D1 of subsequent cycles.

Phase Ia Dose Extension

Subjects with sparse PK sampling will have ECG at C1D1 (predose, 2 hours postdose), C2D1 (predose), C3D1 (predose), and D1 for subsequent cycles.

Subjects who underwent intensive PK sample collection had ECG examinations performed in the same manner as in the Ia dose-escalation phase.

Phase Ib and Phase II study parts

ECG examination Time points are: C1D1 2 hours (± 30 min) after the first dose on the same day, C2D1: before the first dose on the same day, 2 hours (± 30 min) after the first dose on the same day, C1D8, C1D14, C3D1 and subsequent cycle visits, ECG examinations are recommended to be performed within 1 to 4 hours after GFH925 administration. Electrocardiograms should be performed after the subject has rested in a quiet recumbent position for at least 5 minutes. Including at least heart rate, QT, QTc (Fridericia formula, i.e. QTcF) and P-R time. 3 times (at least 5 minutes apart each time) will be checked during the screening period, and the mean 3 times QTcF will serve as the

baseline QTcF. The study doctor will compare the change in ECG from baseline during the study for safety assessments. If the QTcF increases from baseline > 30 msec , or an absolute QTcF value ≥ 450 msec occurs in any of the specified ECG measurements, then an additional 2 ECG tests will need to be averaged and separated by at least 5 minutes to ensure the accuracy of the measurement .

6.1.3. Adverse Events and Concomitant Therapy

Adverse events were assessed by name, severity (graded according to CTCAE version 5.0), start and end time, whether serious, relationship to study treatment, action taken with study treatment, and outcome.

AEs occurring after signing the informed consent form and up to 30 days after the last dose of the study will be recorded in the original medical records and collected in the eCRF. Other medications or therapies taken by the subject while receiving study treatment were documented in the original medical records as concomitant therapies and collected in the eCRF.

6.2. Pharmacokinetic Evaluation

6.2.1 Blood Sampling

- **Phase Ia Dose Escalation**

Serial pharmacokinetic samples will be collected at C1D1 (single dose) and C1D21 (steady state) to assess the pharmacokinetics of GFH925. See for specific time points. Pre-dose trough samples will be collected on C1D10 and C3D1.

Table 11 Pharmacokinetic Plasma Sample Collection Schedule (Ia Dose Escalation Period - QD/BID)

Cycle	Day	Planned Time Point (Hours)	Sampling Window
**Single-dose PK**			
1	1	Pre-dose	-1 hour
1	1	0.5 postdose (same below relative to C1D1 postdose)	± 5 minutes
1	1	1	± 10 minutes
1	1	2	± 10 minutes
1	1	4	± 15 minutes
1	1	8	± 30 minutes

Cycle	Day	Planned Time Point (Hours)	Sampling Window
1	1	12	± 30 minutes
1	2	24	± 1 hour
1	3	48 (predose on Day 3)	± 2 hours
**Multiple-dose PK**			
1	10 [1]	Pre-dose	- 1 hour
1	21	Pre-dose	- 1 hour
1	21	0.5 postdose (same below relative to C1D21 postdose)	± 5 minutes
1	21	1	± 10 minutes
1	21	2	± 10 minutes
1	21	4	± 15 minutes
1	21	8	± 30 minutes
1	21 [2]	12	± 30 minutes
2	1	24 (C2D1 pre-dose)	- 1 hour
**Cycle 3**			
3	1 [1]	Pre-dose	- 1 hour

Note: 1. C1D10 , C3D1 allowed ± 1 day (e.g. C1D10 allowable sampling at C1D9 predose 1 hour or C1D11 predose 1 hour), other sampling times must be collected at the time points specified in Table 11.2.When administering BID, C1D21 PK sampling point will be collected within 1 hour before the start of the second dose.

- **Phase Ia Dose Extension**

See Table 12 for PK sparse sampling time points for enrolled patients. Blood sampling will be collected prior to Day 1 dosing in Cycle 1, 2 and 3.

For dose groups in Phase Ia extension phase, if the number of patients who have intensive PK samples is less than 6, it is recommended to supplement at least 6. Intensive PK sample collection schedule is the same as Ia dose escalation phase in Table 11.

Table 12 Pharmacokinetic Plasma Sample Collection Schedule (Ia Dose Expansion Period - QD/BID)

Cycle	Day	Scheduled Time Point (hours) [2]	Sampling Window
1	1	Pre-dose	- 1 hour
2	1 [1]	Pre-dose	- 1 hour
3	1 [1]	Pre-dose	- 1 hour

Note: 1. C2D1 , C3D1 allowed ± 1 day.

2. When administering BID, the scheduled time point will be the pre-dose or post-dose time point for the first dose of GFH925 on that day.

- **Phase Ib and Phase II Study Parts**

Specific time points for PK sampling in enrolled patients are shown in Table 13.

Table 13 Schedule for Pharmacokinetic Plasma Sample Collection (Phase Ib and Phase II Study Parts)

Cycle	Day	Scheduled Time Point (hours) [2]	Sampling Window
1	1	Pre-dose	- 1 hour
2 [1]	1	Pre-dose	- 1 hour
2 [1]	1	2h post-dose	± 10 minutes
3 [1]	1	Pre-dose	- 1 hour

Remarks:

1. C2D1, C3D1 allow ± 1 day.
2. Scheduled time points were pre-dose or post-dose time points for the first dose of GFH925 on the day.

- **Unscheduled PK Blood Sampling**

When a patient had a serious adverse event, at the moment when the investigator is aware of the event, if possible, a blood sample at that time should be collected for GFH925 concentration determination.

During the study, the sponsor may adjust, reduce, or stop PK sampling and/or determination based on clinical development needs, overall development strategy, and protection for subjects, and these adjustments will take effect upon receipt at each site of an official email or protocol clarification letter from the sponsor, whichever is received first.

6.2.2 Sample Analysis Method

Concentrations of GFH925 will be determined using a validated liquid chromatography tandem mass spectrometry (LC-MS/MS) method. Concentrations below the lower limit of quantitation (LLOQ) will be reported as 0 ng/mL and flagged accordingly for missing samples. Details regarding the analysis will be documented in the bioanalytical report.

The collection and processing methods of biological samples are detailed in the laboratory

operation manual.

6.2.3 Calculation of Pharmacokinetic Parameters

Pharmacokinetic parameters of GFH925 after single dose and multiple dose administration will be calculated using a non-compartmental model. If data permit, all PK plasma concentration - time data from this study will be combined with useful PK data from other studies and a separate population PK analysis will be performed using a population pharmacokinetic model-based approach. PK analysis results are summarized in a separate report.

6.3. Efficacy Evaluation Value

Evaluation of tumor response will include all known or suspected sites of disease. Imaging included computed tomography (CT) or magnetic resonance imaging (MRI) scans of the chest, abdomen, or pelvis (contrast enhancement unless contraindicated); cranial contrast-enhanced CT or MRI for known or suspected brain metastases in subjects; and bone scans and/or bone X-rays for known or suspected bone metastases in subjects. Cavity imaging of head, chest, abdomen and pelvis within 28 days prior to the first dose and bone scan within 42 days prior to the first dose are acceptable.

Subjects with lung cancer must undergo enhanced head CT or MR scanning during the screening period to assess central nervous system (CNS) metastases.

If lesions were present at baseline, they were followed up during the visit using the same scanning modality and scanning parameters. For subjects without corresponding lesion in baseline cranial imaging and bone scan, investigator may decide to add corresponding examinations if clinically indicated during treatment.

Imaging and tumor assessments will be performed every 6 weeks ± 1 week for the first 48 weeks of the study and then every 12 weeks ± 1 week thereafter. In the subsequent tumor evaluation, the same imaging method should be used for lesions of the same type as in the screening period, and the examination should be performed on the same imaging equipment as far as possible. The evaluation of antitumor activity will be performed during the screening period and during the course of treatment by radiographic imaging according to the study flow chart; it should also be performed when disease progression (e.g., symptomatic deterioration) is suspected and the subject withdraws from treatment (imaging performed within 4 weeks without repeating).

[redacted]

Disease response evaluation will be performed according to RECIST Version 1.1 (Appendix 2). Tumor evaluation will be performed by the investigator in the dose escalation and dose expansion part of Phase Ia and Phase Ib, [redacted]

All subjects must have radiographic imaging consistent with RECIST criteria, and all materials should be properly retained and available for source verification and peer review

6.4. Biomarkers and Mechanisms of Resistance Exploration

Pre-treatment FFPE tissue samples from patients in the clinical Phase II trial were sequenced at Burning Rock Center Laboratory using the "Human 9-Gene Mutation Joint Detection Assay (Reversible Termination Sequencing)". And KRAS G12C mutation-positive patients can be enrolled in the clinical trial.

This study will explore possible mechanisms of primary and secondary resistance at the genetic level. Potential primary and secondary resistance mechanisms will be investigated by comparing circulating tumor DNA at baseline and at disease progression as well as selected gene mutations or fusions in tissue samples. Refer to Table 14 for sample types, collection time.

During the course of the clinical study, the Sponsor may reduce or stop sampling and/or testing based on clinical development needs, overall development strategy, and purpose of protecting subjects. These adjustments will take effect as soon as each site receives an official email or protocol clarification letter from the Sponsor (whichever received first).

Please refer to the laboratory manual for sample preparation and processing details.

For tumor tissue samples, if limited samples are obtained at baseline, testing will be scheduled according to the priority order of detection of KRAS G12C mutation over testing of associated oncogene or tumor suppressor gene mutation or fusion. Tumor tissue samples should also be collected whenever possible upon disease progression. If tumor

tissue sample cannot be obtained after disease progression, it will not be considered as protocol deviation.

Table 14 Biomarker Sample Collection and Analysis

Sample type	Detected	Collection Time Point	Test Indicators	Test method
Plasma	Circulating tumor DNA	• **Baseline** : Cycle 1 D1 Predose<br>• **Progressive disease** : within one week upon diagnosis of radiographic progression by the investigator	Mutations or fusions in oncogenes or tumor suppressor genes associated with tumor development and progression, including genes in the following categories:<br>• Receptor tyrosine kinases and their downstream genes<br>• Cell proliferation, apoptosis/anti-apoptosis related genes<br>• Genes related to tumor nutrition and energy metabolism<br>• DNA damage repair associated gene<br>• Angiogenesis related gene	Next generation DNA targeted sequencing method (mutation or fusion)
Tissue	Tumor tissue DNA	• **Baseline** : archival tissue sample or pre-treatment fresh biopsy<br>• **Progressive disease** : within one week upon diagnosis of radiographic progression by the investigator		

6.5. Storage and Destruction of Biological Samples

Samples will be disposed of or destroyed as well as undergo pooled anonymization. Additional analyses may be performed on anonymized and pooled samples to further assess and validate the analytical method. Any results obtained from these analyses may be reported separately from the Clinical Study Report (CSR).

Sample reproducibility analysis (if performed) will be performed concurrently with the bioanalysis of the samples. Results of these assessments will not be reported in the CSR, but will be presented in a separate bioanalytical report.

6.6. Other Procedures

6.6.1. Unscheduled Visit

If the investigator judges that due to the need for safety assessments, follow-up of

safety events, or the presence of clinical symptoms suggestive of disease progression, the investigator may arrange appropriate follow-up visits for further examinations and assessments.

7 Safety Reporting and Adverse Event Management

7.1. Definition of Adverse Events

Collection of AE information start from the time subjects sign the informed consent form until the end of the safety follow-up period (30 days after the last dose) (see Protocol Section7.5). An AE may present with symptoms, signs, diseases, or laboratory abnormalities, but does not necessarily have a causal relationship with the investigational product, including, but not limited to:

- Worsening of pre-existing (prior to entry into the clinical study) medical conditions/diseases (including worsening of symptoms, signs, laboratory abnormalities);
- Any new AE: any new unfavourable medical condition (including symptoms, signs, newly diagnosed diseases);
- Abnormal clinically significant laboratory findings.

7.2. Definition of Serious Adverse Events

An SAE refers to an untoward medical occurrence in a subject that meets one or more of the following criteria during the study:

- results in death,
- is Life-threatening (defined as an event in which the subject was at risk of death at the time of the event and does not refer to an event which hypothetically might have caused death if it were more severe),
- requires inpatient hospitalization or prolongation of existing hospitalization, excluding the following:
  - ✓ rehabilitation facility,
  - ✓ nursing homes,
  - ✓ routine emergency room admission (less than 24 hours),
  - ✓ same-day surgery (eg, outpatient/same-day/ambulatory surgery).
  - ✓ hospitalization or prolongation of hospitalization not associated with worsening of an AE is not itself an SAE. For example, the following are not considered SAEs:

  - admission to the hospital for treatment of the underlying disease, which is not associated with a new AE and does not involve exacerbation of the underlying disease (eg, for diagnostic testing of the laboratory abnormalities that persist until before the study),
  - hospitalization for administrative reasons (eg, routine annual physical exam),
  - protocol-specified hospitalization during the clinical study (eg, operating as required by the study protocol),
  - elective hospitalization not associated with an AE (eg, elective cosmetic surgery),
  - pre-planned treatment or surgical procedure (documented throughout the study protocol and/or individual subject baseline data),
  - admitted only for blood product use.

  Diagnostic or therapeutic invasive (eg, surgical), noninvasive procedures should not be reported as AEs, but disease conditions that result in the procedures should be reported if they meet the definition of an AE. For example, acute appendicitis with onset during the AE reporting period should be reported as an AE, and appendectomy should therefore be recorded as the treatment for that AE.

- results in persistent or significant disability/incapacity,
- is a congenital anomaly or birth defect,
- is an important medical event.

  Medical and scientific judgment must be exercised in deciding whether other situations should be considered SAEs, such as important medical events that may not be immediately life-threatening or result in death or hospitalization but may jeopardize the patient or may require intervention to prevent one of the outcomes listed in the definition above. Examples of such events are intensive treatment in an emergency room or at home for allergic bronchospasm; blood dyscrasias or convulsions that do not result in hospitalisation; or development of drug dependency or drug abuse.

7.3. Assessment of Severity of Adverse Events

Refer to CTCAE Version 5.0 grading criteria for AEs. In case of AEs with no specific terms not listed in CTCAE, the following criteria may be used:

Table 15 Criteria for judging the severity of adverse events

Grade	Clinical description of severity
1	Mild; asymptomatic or mild symptoms; clinical or diagnostic observations only; intervention not indicated.
2	Moderate; minimal, local or noninvasive intervention indicated; limiting age-appropriate instrumental activities of daily living; instrumental activities of daily living refer to preparing meals, shopping for groceries or clothes, using the telephone, managing money, etc.
3	Severe or medically significant but not life-threatening; hospitalization or prolongation of hospitalization indicated; disabling; limiting self care activities of daily living. Self-care activities of daily living refer to bathing, dressing and undressing, feeding self, using the toilet, taking medications, and not bedridden.
4	Life-threatening consequences; urgent intervention indicated.
5	Death related to AE.

7.4. Judgment of Causal Relationship between Adverse Events and Investigational Drug

Causal relationship of an AE to the investigational drug is to be determined by a comprehensive assessment whether there is a reasonable possibility that the investigational drug caused or contributed to the AE. Factors that the investigator should consider include whether there is a reasonable temporal relationship between the onset of the AE and the administration of the investigational drug, the properties of the investigational drug, the toxicological and pharmacological effects of the investigational drug, the use of concomitant therapy, the subject 's underlying disease, medical history, family history, etc. Facts and grounds for determining causality should generally be provided.

In this study, the causal relationship between AE and the investigational drug will be evaluated as "related" or "unrelated" by the investigator using a dichotomy.

7.5. Recording of Adverse Events

The investigator should record AEs or SAEs using medical terminology/concepts, and should avoid colloquialisms and abbreviations. All AEs, including SAEs, should be recorded on the Adverse Event Form in eCRF.

7.5.1. Adverse Events Collection and Collection Period

Collection of AE information starts from the time the subjects sign the informed consent form until the end of the safety follow-up period.

Table 16 Collection of AEs/SAEs/Pregnancy Events

Collection Period	Collection Requirements
From the time the subjects sign the informed consent form until the end of the safety follow-up period	all AEs/SAEs/Pregnancy Events,<br>non-SAEs not related to the investigational drug will not be collected if new anticancer therapy is started before the end of safety follow-up,<br>pregnancy events: collection starts after first dose of the investigational drug.
After the above period	SAEs related to the investigational drug

Abbreviations: AE = adverse event, SAE = serious adverse event.

7.5.2. Follow-up of Adverse Events

At each study visit, the investigator should assess whether the subject experienced an adverse event. All adverse events should be followed up until resolution, or return to baseline, or stabilization and judged as irreversible by the investigator, or reasonably explained (eg, loss to follow-up, death), or until the event is conclusively determined to be unrelated to the study drug or the study procedure by the end of the safety follow-up period. Every effort should be made to ensure that the subject has the best outcome and a definitive causality assessment.

All AEs should be recorded in detail on the AE page of the eCRF, including: event term, severity (graded according to CTCAE version 5.0), onset and end time, whether it is a SAE, relationship to the investigational drug, action taken with the investigational drug, and outcome.

7.5.3. Contents of Adverse Event Records

The investigator should completely record any AEs, including diagnosis (if no diagnosis, record the symptoms and signs including laboratory abnormalities), onset and end dates and time (if applicable), CTCAE severity grade and change (Grade 3 or above events), whether it is a SAE, measures taken for the investigational drug, treatment given due to AE and the outcome of the event, and the relationship between the AE and the investigational drug.

For a SAE, the investigator should also provide the date on which the AE meets SAE criteria, the date on which the investigator became aware of SAE, the rationale for the AE being SAE , the date of hospitalization, the date of discharge, the possible cause of death, the date of death, whether an autopsy was performed, the causality assessment with the

study procedures, the causality assessment with other drugs, and other possible causes of SAE . The investigator should also provide the rationale for causality determination and SAE description. In the SAE description, it also needs to include the subject 's number, age, gender, height and weight; the subject's indication for the investigational drug treatment, disease stage and relevant general conditions; the clinical course such as occurrence, development and outcome; laboratory test results related to SAE (the examination time, unit and normal range must be provided); the relevant past history, concomitant diseases and their occurrence and duration; the relevant medication history, concomitant drugs and their start, duration and dosage; and details such as the start, duration and dosage of the investigational drug treatment.

Matters concerning the AE record are described below:

Diagnosis, symptoms and signs

If a diagnosis has already been made, the AE term should be recorded as the diagnosis rather than the individual signs and symptoms (eg, record as liver failure rather than jaundice, elevated transaminases, and asterixis). If symptoms and signs cannot be determined to be caused by the diagnosis at the time of reporting, they will be recorded as a separate AE/SAE. If it is determined during AE/SAE follow-up that the symptoms and signs are caused by the diagnosis, only the diagnosis will be reported separately, and the symptoms and signs will be included in the diagnosis and will no longer be recorded as AE. For SAE, a follow-up report will be sent.

Adverse events secondary to other events

In general, if adverse events are secondary to other events (e.g., caused by other events or clinical sequelae of other events), the primary events should be recorded unless the secondary events are severe or serious adverse events. However, secondary events of significant clinical significance should be recorded as independent adverse events in the eCRF if they occur at a different time from the primary events. If the relationship between the events is unclear, it should be recorded separately in the eCRF.

Persistent or recurrent adverse events

Persistent adverse events refer to those adverse events that have not resolved between the two evaluation time points of the subject and have persisted.

Recurrent adverse events refer to adverse events that have resolved between two

evaluation time points but subsequently recurred. The occurrence of the event should be documented separately.

Laboratory Abnormalities

Clinically significant laboratory abnormalities should be reported as AEs. It is the responsibility of the investigator to review all laboratory abnormalities and make medical judgment as to whether each laboratory abnormality should be reported as an AE.

Pre-existing Medical Status

Subjects' pre-existing symptoms/signs during the screening period of the trial should be recorded and reported as AEs only if there is worsening in severity, frequency or nature (except worsening of the disease condition being investigated) after entering the trial. Changes from previous states such as "increased frequency of headache" should be reflected in the records.

Death

Deaths that occur throughout the trial, whether considered related to the investigational drug or not, should be recorded on the death report form in the eCRF and promptly reported to the sponsor.

If the subject died during the adverse event collection period (see Section 7.5.1), the cause of death will be recorded as an adverse event (except for deaths due to disease progression) when the cause of death is identified, and the outcome of this adverse event will be death, and the event will also be reported as an SAE; if the cause of death is unknown at the time of reporting, it should be recorded as "unexplained death" on the adverse event form, and "unexplained death" will be reported as an SAE first, followed by further investigation into the exact cause of death. Subject deaths outside the adverse event collection period are not to be reported as SAEs unless considered related to the investigational drug.

Disease progression

Clinical symptoms resulting from malignant tumor progression may be recorded as AEs if they cannot be fully and exclusively determined to be due to disease progression under study or are not consistent with the symptom profile of disease progression under study. If clearly consistent with suspicion of disease progression, it should not be recorded as an AE. Malignant neoplasm progression per se is not to be reported as an AE.

Clinical symptoms resulting from malignant tumor progression will be reported as SAEs if they meet the criteria for SAEs and can not be fully and exclusively determined to be due to disease progression under study or do not match the symptom profile of disease progression under study. An event will not be reported as an SAE if it meets SAE criteria and is clearly consistent with suspected progression of the disease under study.

Drug overdose

There are no data on overdose with GFH925 in humans. If a patient mistakenly takes more than a specified dose, it is an overdose. Investigators will be advised to closely monitor any subject receiving a higher than specified dose and document the actual dose and date used, manage with appropriate supportive care, and perform scheduled follow-up.

In the event of an overdose of the investigational drug, the investigator should:

1. contact Sponsor immediately,
2. closely monitor subjects for AEs/SAEs and laboratory abnormalities, as appropriate.
3. document the overdose and corresponding duration in the eCRF.
4. make decisions regarding the need for dose interruptions or modifications in consultation with the sponsor based on the clinical evaluation of the subject.

7.6. SAE, Pregnancy and Abnormal Hepatic Function Event Reporting

7.6.1. SAE Report

In case of an SAE, whether it is an initial report or a follow-up report, the investigator must immediately fill out Innovent 's *Serious Adverse Event Reporting Form*, sign and date, immediately notify the sponsor within 24 hours of the investigator' s awareness, and report to the relevant unit in a timely manner according to local regulatory requirements. SAEs occurring after the safety follow-up period should be collected from those suspected to be related to the study drug. For SAEs, symptoms, severity, correlation with the study drug, occurrence time, treatment time, measures taken, follow-up time and method, and outcome should be recorded in detail. If the investigator considers an SAE not related to the investigational drug but potentially related to study conditions (eg, discontinuation of original treatment, or comorbidities during the course of the study), the

relationship should be specified in the narrative section of the *SAE Report Form*.

The email address for receiving SAE and pregnancy reports from the sponsor of this project is:

[redacted]

7.6.2. Pregnancy Report

If a female subject becomes pregnant during the clinical study, the subject must discontinue study treatment and withdraw from the study; if the partner of a male subject becomes pregnant during the clinical study, the subject may continue the clinical study. The investigator should complete the *Pregnancy Report* and report it to the sponsor within 24 hours of awareness of the pregnancy event.

The investigator should follow up the pregnancy until the final outcome (including any premature termination or delivery), 1 month after delivery of the mother, and report the pregnancy outcome to the sponsor. If the pregnancy outcome meets the criteria for SAE (eg, ectopic pregnancy, spontaneous abortion, intrauterine fetal death, neonatal death, or congenital anomaly), it needs to be reported following the SAE procedure.

If a subject experience a SAE during pregnancy at the same time, it must also be reported following SAE reporting procedures.

7.6.3. Reporting of Abnormal Hepatic Function Events

Abnormal AST and/or ALT levels concurrent with abnormal elevations in total bilirubin levels that meet the following (1) (2) (3) conditions and with no other causes for this abnormality, should always be reported as SAEs following the SAE reporting process.

Table 17 Criteria for judging abnormal liver function

Conditions met	Criteria for judgment
(1) abnormal ALT or AST	1) normal baseline: ALT or AST >3×ULN during treatment<br>abnormal baseline: ALT or AST >3×baseline during treatment
(2) abnormal TBIL	2) normal baseline: TBIL > 2×ULN during treatment<br>abnormal baseline: TBIL > 2×baseline during treatment
(3) alkaline phosphatase < 2×ULN (or information not available)	

Abbreviations: ALT = alanine aminotransferase, AST = aspartate aminotransferase, TBIL = total bilirubin, ULN = upper limit of normal.

Patients with abnormal AST and/or ALT levels concurrent with abnormal elevations in total bilirubin levels during the treatment or follow-up periods should return to the study site for examination and evaluation as soon as possible after learning of the abnormal results. Relevant liver function laboratory tests, detailed history and physical examination should be included and the possibility of liver tumor (primary or secondary) should be considered.

In case of hepatic impairment meeting the above criteria, dose modification or discontinuation should be done accordingly following 5.3.1 and 5.3.2.

8 Statistical Test

8.1. Statistical Analysis Plan

Statistical analyses of study data will be programmed using SAS Version 9.4 or higher. Statistical analysis methods will be described more specifically in the Statistical Analysis Plan (SAP).

8.2. Statistical Hypothesis Testing and Sample Size Calculation

8.2.1. Phase I Studies

Phase Ia was a dose-escalation and dose-expansion design without verification of statistical hypotheses.

Dose escalation part:

With the exception of the accelerated titration dose groups, subjects were enrolled 3 to 6 per group for treatment, with a planned maximum sample size of approximately 45 . Additional subjects may be enrolled for treatment if additional dose levels or other dosing schedules are to be explored beyond the planned dose level.

Dose expansion part:

Up to 200 subjects are expected to be enrolled.

Phase Ib study, no statistical hypothesis testing. It is expected to enroll 40~200 subjects,.

[redacted]

[redacted]

[redacted]

[redacted]

[redacted]

[redacted]

[redacted]

[redacted]

[redacted]		[redacted]			[redacted]
[redacted]	[redacted]		[redacted]		[redacted]
[redacted]	[redacted]				[redacted]
[redacted]	[redacted]	[redacted]	[redacted]	[redacted]	[redacted]

[redacted]	[redacted]	[redacted]	[redacted]	[redacted]	[redacted]

[redacted]

[redacted]

[redacted]

[redacted]

[redacted]

GFH925.

[redacted]

[redacted]

[redacted]

[redacted].

8.4. Statistical Analysis Methods

8.4.1. General Methods of Statistical Analysis

Descriptive statistics will be mainly used for summarization: measurement data will be described using number of cases, mean, standard deviation, median, minimum and maximum; enumeration data will be described using number of cases and percentage. For Time-to-event variables (event time variables such as PFS, OS and DoR), Kaplan-Meier Plot, median and 95% confidence interval will be used for statistical description. Unless otherwise specified, study data will be summarized in stages by dose and cohort (as appropriate).

The number of decimal places for the minimum and maximum values will be consistent with that recorded in the database. Means and medians will be presented to one

more decimal place than the raw data recorded in the database, and standard deviations, coefficients of variation, and confidence intervals will be presented to two more decimal places than the raw data recorded in the database. Calculated frequencies, percentages, and confidence intervals for count and rank variables will be presented to one decimal place. P values will be presented to 4 decimal places.

8.4.2. Efficacy Analysis

Tumor assessments were performed by the investigator in Phase I [redacted]. Efficacy endpoints in Phase Ia included investigator-assessed ORR, DCR, DoR, TTR, PFS, and OS. The primary efficacy endpoint of Phase Ib study is investigator-assessed ORR. The primary efficacy endpoint in Phase II was ORR as assessed by IRRC.

BOR was summarized as the number and percentage of subjects with CR, PR, SD, PD. Confirmed ORR was calculated and 2-sided 95% CI was calculated using Clopper-Pearson method. The efficacy of GFH925 was considered statistically significant if the lower limit of 95% CI of ORR in Phase II study was greater than 23%.

Secondary efficacy endpoints in Phase Ib and Phase II studies included DCR, DoR, PFS, TTR, and OS. Confirmed DCR was calculated and 95% CI was calculated using Clopper-Pearson method. DoR, PFS and OS were analyzed using the Kaplan-Meier (K-M) method to estimate the median and 95% CI, and the corresponding K-M curves were plotted. Descriptive statistics were provided for TTR.

Efficacy analysis will be performed based on FAS in Phase I study. [redacted]. Sensitivity analyses will also be performed based on tumor assessments provided by the investigator as supportive rationale. The primary analysis of PFS and OS will be performed on the FAS according to IRRC 's tumor assessment and sensitivity analysis will be performed on PPS. Sensitivity analyses will also be performed based on tumor assessments provided by the investigator as supportive rationale.

Each of these efficacy endpoints is defined as follows:

- Objective response rate (ORR): proportion of participants achieving a

complete response (CR) or partial response (PR).

- Disease control rate (DCR): proportion of subjects achieving CR , PR or stable disease (SD).

- Duration of Response (DoR): The time from the subject 's first evidence of confirmed CR or PR (ie, the first time that CR or PR assessment criteria are met) to disease progression or death, whichever occurs first.

- Progression-free survival (PFS): the time from the first GFH925 treatment until disease progression or death, whichever came first.

- Time to response (TTR): the time from the first treatment with GFH925 until the first evidence of a confirmed CR or PR (ie, the first time that CR or PR assessment criteria are met).

- Overall survival (OS): the time from the first GFH925 treatment until death from any cause.

8.4.3. Subject Disposition

Subject disposition was summarized based on all subjects who participated in screening. Subjects' screening results, receiving drugs, reasons for ending treatment, reasons for withdrawing from the study, and inclusion in each analysis dataset were summarized.

Subjects' screening failure, treatment termination, study withdrawal and reasons for exclusion from each analysis dataset will be listed in detail.

8.4.4. Protocol Deviations

Major protocol deviations during the trial were summarized and listed by protocol deviation category based on FAS. Subjects with multiple major protocol deviations in different categories were counted in each corresponding category (by number).

8.4.5. Prior and Concomitant Medications/Therapy

Subjects' previous anti-tumor therapies (including drug therapy, radiotherapy, surgical treatment, etc.) will be summarized and listed. Prior and concomitant medications will be summarized and listed by subject.

8.4.6. Concomitant Therapy

Treatment concomitant medications were coded according to the World Health Organization Drug Dictionary (WHODrug) and summarized according to the Anatomic Therapeutic Chemical Classification System (ATC) and Preferred Name (PN).

8.4.7. Safety Analysis

Safety data will be statistically analyzed according to dose group, cohort (if applicable) , and study phase (Phase Ia/Phase Ib/Phase II). All safety analyses will be analyzed according to SS except for DLT analysis according to DDS.

8.4.7.1. Drug Exposure

Based on the SS, the total dose, exposure time , dose intensity, and relative dose intensity of the study drug actually taken by the subject were summarized and listed. The number and percentage of subjects were summarized for each relative dose intensity category.

8.4.7.2. Adverse Events

Adverse events will be handled in the statistical analysis after coding according to the Medical Dictionary for Regulatory Activities (MedDRA). Number and percentage of subjects with treatment-emergent adverse events (TEAEs), AEs related to study drug, SAEs, AEs leading to study treatment discontinuation, AEs leading to study treatment discontinuation, AEs leading to study treatment discontinuation, and AEs leading to dose adjustment will be summarized for each group. The above adverse events will be summarized by System Organ Class (SOC), Preferred Term (PT) and/or CTCAE grade, and a list of adverse events will be provided.

In addition to the above analyses, DLTs in the escalation phase of Phase Ia study will be summarized according to the DDS and listed.

8.4.7.3. Laboratory Tests

Descriptive statistics were performed for laboratory test results and changes from baseline by visit. Cross classification tables were used to describe changes in laboratory test results before and after dosing. Laboratory results were listed for all subjects.

8.4.7.4. 12 Lead ECG

Descriptive statistics were performed on 12-lead ECG results and changes from baseline by visit. Changes in clinical judgment results before and after administration were summarized. A listing of 12-lead ECG results was presented for all subjects.

12-lead ECG will be performed 3 times (at least 5 minutes apart each time) during the screening period, and the mean of the 3 times will serve as the baseline value (see6.1.2.2 Section).

8.4.7.5. Vital signs

Descriptive statistics were performed for each vital sign result and change from baseline by visit. Vital signs were listed for all subjects.

8.4.8. Subject Baseline Characteristics

Demographic data (including gender, age, height, weight) and baseline characteristics (including disease condition, past medical history) of subjects will be summarized and listed based on FAS.

8.4.9. Pharmacokinetic Analysis

GFH925 plasma concentrations were analysed descriptively by sampling time point. Individual and mean concentration-time curves were plotted. Descriptive analyses were performed for each PK parameter. Listings of plasma concentrations and PK parameters were presented for all subjects.

8.4.10. Biomarker Analysis

Gene mutations in tumor tissue and peripheral blood at baseline and at disease progression will be tabulated. Correlations between efficacy and biomarkers may be further explored if necessary.

KRAS G12C mutation will be detected by LungCure assay at Burning Rock Center Laboratory. The consistency verification study of diagnostic reagents will be completed by Burning Rock. The test results from this study will support the prediction of response of GFH925 in advanced NSCLC patients with KRAS G12C mutations.

8.4.11. Analysis Node

8.4.11.1Phase I Data Analysis

Critical data will be summarized and reported following Phase I study completion.

[redacted]

[redacted]

[redacted]

[redacted]

[redacted]

[redacted]

[redacted]

[redacted]

[redacted]

8.4.13. Multiple comparisons and multiplicity adjustment

Not applicable.

8.5. Measures to Control Bias

8.5.1. Randomization and Blinding

This study is a single-arm open-label study and is not applicable.

8.5.2. Assessment of Blinded Maintenance

Not applicable.

8.5.3. Unblinding and Emergency Unblinding

Not applicable.

9 Research Quality Assurance and Quality Control

According to the guidelines of Good Clinical Practice (GCP), the sponsor is responsible for implementing and maintaining the quality assurance and quality control system according to the corresponding standard operating procedures to ensure the implementation of the clinical trial and the authenticity of the data, and collecting, recording and reporting in accordance with the protocol, GCP and corresponding regulatory requirements.

9.1. Clinical Monitoring

The Sponsor or a contract research organization (CRO) authorized by the Sponsor will conduct clinical monitoring of this study. The CRA shall conduct monitoring according to the standard operating procedures of the Sponsor or CRO and have the same rights and responsibilities as the Sponsor 's monitor. The CRA should maintain regular communication with the investigator, authorized personnel of the trial and the sponsor.

Prior to the start of the study, the monitor will assess each site 's competence and report problems regarding facilities, technical equipment, or medical personnel to the sponsor. During the study, the monitor will be responsible for monitoring whether the investigator has obtained written informed consent from all subjects and whether the data records are correct and complete. At the same time, the monitor will also compare the data entered into the eCRF with the original data and inform the investigator of any errors or omissions. The monitor will also control compliance with the study site 's protocol and

trial procedures, arrange for the supply of study medication, and ensure that the medication is maintained under appropriate conditions.

Monitoring visits will be conducted in accordance with applicable laws and regulations. Each site will receive regular monitoring visits from the time of subject enrollment. After each visit to the investigator, the monitor should submit a written report to the sponsor.

9.2. Data Management

An electronic data capture (EDC) system will be used for this study and study data will be entered into the eCRF by the investigator or authorized study personnel. Prior to site initiation or data entry, appropriate training will be provided to the investigator and authorized study personnel, and appropriate security measures will be taken for computers and other equipment used.

Data entry into the eCRF should be completed during or as soon as possible after the visit and updated at any time to ensure that it reflects the latest developments in the subjects participating in the study. To avoid differences in outcome assessments by different assessors, it is recommended that the baseline and all subsequent efficacy and safety assessments be completed by the same person for the same subject. The investigator must review the data to ensure accuracy and correctness of all data entered into the eCRF. If some assessments were not performed during the course of the study, or some information was unavailable, not applicable, or unknown, the investigator was to record it in the eCRF. The investigator should electronically sign the verified data.

The clinical research associate (CRA) will review the eCRFs against the source documents and assess their completeness and consistency, and the CRA will compare the eCRFs against the source documents to ensure consistency of all data. All data entry, correction, and modification will be the responsibility of the investigator or his/her designee. Data from the eCRFs will be submitted to the EDC database and any changes to the data will be documented in the audit trail, i.e. the reason for the change, the operator user name, date and time of the change will all be documented. The roles and authorities of the site staff responsible for data entry will be predetermined. In case of data query, CRA or data management personnel will issue a query in EDC and relevant site personnel will be responsible for answering the query. The EDC system will document the audit trail of the query, including user name, date and time.

Unless otherwise specified, the eCRF will be used only as a form for collecting data and not as source data. Source documents are those used by the investigator or hospital and related to the subject, and can prove the existence of the subject, inclusion and exclusion criteria and all records of the subject participating in this study, including laboratory records, ECG results, medication records, subject folders, etc.

The investigator is responsible for maintaining all source documents and providing them to the CRA for monitoring at each visit. In addition, the investigator must submit a complete eCRF for each enrolled subject regardless of the duration of study participation. The protocol number and subject number in all supporting documents (eg, laboratory records or hospital records) submitted together with the eCRF should be carefully verified, and all personal privacy information (including the subject 's name) should be removed or rendered illegible to protect the subject' s privacy. The investigator documented by electronic signature that he/she had reviewed all eCRF data to ensure the validity, completeness, and accuracy of the data. The electronic signature will be completed using the investigator 's user ID and password, and the system will automatically attach the date and time of signature at the same time. The investigator shall not share the user ID and password with other personnel. Changes to the data in the eCRF will be made according to the workflow defined by the EDC system. All changes and reasons for changes will be documented on the audit trail.

9.3. Quality Assurance Audits

During the course of the study, the sponsor or its authorized representative may conduct quality assurance audits of the study site, the study database, and related study documentation, and appropriate regulatory authorities may conduct inspections of the study site, the study database, and related study documentation at their discretion. The investigator was to notify the sponsor immediately upon notification of an inspection by a regulatory authority.

The Sponsor 's Quality Assurance Unit conducted audits of the investigational sites. Audits include: drug supply, required trial documentation, documentation of the informed consent process, and consistency of case report forms with source documents. Audit content and scope may also be increased as appropriate. After reasonable notice, the investigator should allow the auditors entrusted by the sponsor to conduct trial-related audits and inspections by regulatory authorities. The primary purpose of the audit or

inspection was to verify that the rights or well-being of subjects participating in the trial were protected, that the informed consent was signed and that the trial was conducted correctly, and that all data related to the evaluation of the study drug were processed and reported in accordance with preplanned arrangements, the protocol, facilities, ethical standard operating procedures, GCP, and applicable regulatory requirements. The investigator shall have direct access to all trial documents, original records and original data.

10 Ethics

10.1. Ethics Committee

The sponsor or its authorized representative will prepare the relevant documents to be submitted to the EC of the site, including the trial protocol, informed consent form, investigator brochure, subject recruitment materials or advertisements and other documents required by laws and regulations, and submit them to the corresponding EC for review and approval. Written approval from the site EC must be obtained and provided to the Sponsor prior to the start of the study. The EC approval letter must specify the name, number and version number of the study protocol as well as the version number (e.g., informed consent form) and approval date of other documents. The investigator should inform the sponsor of the EC 's written comments regarding the delay, suspension and re-approval.

The site must comply with the requirements of the site EC. This may include submission to the EC for review and approval of protocol amendments, ICF amendments, and amendments to subject recruitment materials, local safety reporting requirements, periodic reporting and updates according to EC regulations, and submission of final reports. All of the above documents and EC approvals must be provided to the sponsor or its designee.

10.2. Performed In Study Ethics

The study process and acquisition of informed consent should comply with the Declaration of Helsinki, relevant GCP requirements and relevant laws and regulations on drug and data protection in China.

GCP provides ethical and scientific global quality standards for the design, conduct, recording and reporting of clinical studies involving human subjects. This study will be

conducted in compliance with GCP and applicable national regulations and in accordance with the ethical principles that have their origin in the Declaration of Helsinki to protect the rights, safety and well-being of the subjects.

The investigator should follow the procedures specified in this trial protocol and should not change it without the permission of the sponsor. Any protocol deviations will be reported to the EC, Sponsor, or regulatory authorities.

10.3. Subject Information and Informed Consent

Prior to initiation of any study procedures, the possible risks and benefits of the study will be explained to potential participants using the ICF, and the informed consent language should be simple and understandable. The ICF statement should clarify that signing the informed consent is voluntary, that participation in this study may entail risks and benefits, and that the subject is free to withdraw from the study at any time. The investigator may enroll a subject only after adequate explanation of the details of the study, satisfactory answers to the subject 's questions, and sufficient time to consider them, and written consent from the subject or his/her guardian. All signed informed consent forms must be in the investigator 's file or subject folder.

The investigator is responsible for explaining the content of the informed consent to the subject and obtaining signed and dated informed consent from the subject or his/her guardian prior to the start of the study. After signing, the investigator should give the subject a copy of the signed informed consent form. The investigator should record the informed consent process in the trial source documents.

The initial informed consent form, any subsequent amendments to the written informed consent form, and any written information provided to subjects were to obtain the opinion of the IRB/IEC prior to use. If new information becomes available that may be relevant to the subject 's willingness to continue participation in the trial, the subject or his/her guardian should be informed promptly. Communication of this information will be provided and documented either through the revised informed consent form or the original informed consent appendix (obtaining a dated signature from the subject or a dated signature from the subject 's legal guardian).

10.4. Data Protection

The ICF will contain (or in some cases, together with the use of separate files)

information on data protection and privacy protection.

Precautions were taken to ensure confidentiality of the documents and to prevent identification of the subjects. However, under special circumstances, certain persons may see genetic data and personal identifiers for a subject. For example, in the event of a medical emergency, the sponsor, its representative physician, or the investigator will be aware of the subject identification code and have access to the genetic data of that subject. In addition, relevant regulatory authorities require access to relevant documents.

10.5. Protocol Deviations

Protocol deviations are defined as any noncompliance with the clinical trial protocol, International Conference on Harmonisation Good Clinical Practice (ICH GCP), or Manual of Operations. Non-compliance may arise from the subject, investigator, or site staff. In response to violations, corrective actions should be taken and completed in a timely manner.

11 Study Management

11.1. Data Processing and Record Keeping

Documents in the clinical trial (protocol and protocol amendments, completed eCRF, signed ICF, etc.) should be retained and managed according to GCP requirements. The site should retain these documents until 5 years after the end of the study.

Study documents should be reasonably retained for future access or data traceability. Safety and environmental risks should be considered when saving the document.

No study documents may be destroyed without the written approval of the Sponsor and the Investigator. The investigator/site may transfer the study documentation to another party complying with the document retention requirements or to another location complying with the requirements only after notifying the sponsor and obtaining his/her written consent.

11.2. Raw Data/Document Access

The Investigator agrees that the Sponsor, CRO and relevant authorized regulatory authorities will have direct access to all study-related documents, including subject medical records.

11.3. Protocol Amendment

Any amendment to the protocol during the conduct of the study requires communication and agreement between the sponsor and the investigator. Sponsor shall ensure timely submission of protocol amendments to management.

All amendments to the protocol should be maintained as protocol addenda. Any amendment to the protocol should be submitted to the Ethics Committee for approval or filing according to the provisions of the Ethics Committee. If required, it shall also be submitted to regulatory authorities for review and approval, and may not be implemented until EC and regulatory authority (if required) approval (except for changes to the protocol to eliminate an immediate hazard to trial subjects).

11.4. Investigator Responsibilities

The investigator will conduct this study in compliance with the protocol, the ethical principles of the Declaration of Helsinki, GCP in China, and applicable regulatory requirements.

The detailed responsibilities of relevant investigators are listed in Chapter 5 of GCP (2020 No. 57) in China.

11.5. Publication Policy

All data generated in this study are confidential information of the sponsor, and the sponsor has the right to publish the study results. Information regarding the publication policy between the sponsor and the investigator will be described in the clinical trial agreement.

All information about this trial (not limited to the following documents: protocol and investigator 's brochure) must be kept strictly confidential. The investigator must recognize that the scientific or medical conclusions drawn from this trial may be of commercial value to the sponsor. The investigator shall keep confidential the information and data related to this trial. In order to publish the data related to this trial or the conclusions drawn from this trial, the investigator shall negotiate with the sponsor in advance and obtain the written consent of the sponsor. In order to protect their own rights and interests, the sponsor may require the investigator not to publish relevant trial data before the investigational product is approved for marketing.

The sponsor has the right to publish or publish the information or data related to this trial or submit them to the drug regulatory authority. If the sponsor needs to include the investigator 's name in a publication, publication or advertisement, the investigator' s consent should be obtained.

11.6. Finance and Insurance

The sponsor will purchase insurance for subjects participating in this study in accordance with local regulations and minimum requirements. Insurance provisions will be maintained in the study folder.

12 References

[1] MOORE A R, ROSENBERG S C, MCCORMICK F, et al. RAS-targeted therapies: is the undruggable drugged? [J]. Nat Rev Drug Discov, 2020, 19 (8): 533-552.

[2] KIM D, XUE J Y, LITO P. Targeting KRAS (G12C): From Inhibitory Mechanism to Modulation of Antitumor Effects in Patients [J]. Cell, 2020, 183 (4): 850-859.

[3] DOWNWARD J. Targeting RAS signalling pathways in cancer therapy [J]. Nat Rev Cancer, 2003, 3 (1): 11-22.

[4] WATERS A M, Der CJ. KRAS: The Critical Driver and Therapeutic Target for Pancreatic Cancer [J]. Cold Spring Harb Perspect Med, 2018, 8 (9).

[5] PRIOR I A, LEWIS P D, MATTOS C. A comprehensive survey of Ras mutations in cancer [J]. Cancer Res, 2012, 72 (10): 2457-2467.

[6] OSTREM J M, SHOKAT K M. Direct small-molecule inhibitors of KRAS: from structural insights to mechanism-based design [J]. Nat Rev Drug Discov, 2016, 15 (11): 771-785.

[7] DIAS C P, GUIMARAES C F, CARDOSO A P, et al. KRAS Oncogenic Signaling Extends Cancer Cells to Orchestrate the Microenvironment [J]. Cancer Res, 2018, 78 (1): 7-14.

[8] LOONG H H, Du N, CHENG C, et al. KRAS G12C mutations in Asia: a landscape analysis of 11, 951 Chinese tumor samples [J]. Transl Lung Cancer Res, 2020, 9 (5): 1759-1769.

[9] LIU S Y, SUN H, ZHOU J Y, et al. Clinical characteristics and prognostic value of the KRAS G12C mutation in Chinese non-small cell lung cancer patients [J]. Biomark Res, 2020, 8:22.

[10] BORGHAEI H, PAZ-ARES L, HORN L, et al. Nivolumab versus Docetaxel in Advanced Nonsquamous Non-Small-Cell Lung Cancer [J]. N Engl J Med, 2015, 373 (17): 1627-1639.

[11] RITTMEYER A, BARLESI F, WATERKAMP D, et al. Atezolizumab versus docetaxel in patients with previously treated non-small-cell lung cancer (OAK): a randomised controlled trial [J]. Lancet, 2017, 389 (10066): 255-265.

[12] ROMAN M, BARAIBAR I, LOPEZ I, et al. KRAS oncogene in non-small cell lung cancer: clinical perspectives on the treatment of an old target [J]. Mol Cancer, 2018, 17 (1): 33.

[13] MASI G, SALVATORE L, BONI L, et al. Continuation or reintroduction of bevacizumab progression to beyond first-line therapy in metastatic colorectal

cancer: final results of the randomized BEBYP trial [J]. Ann Oncol, 2015, 26 (4): 724-730.

[14] IWAMOTO S, TAKAHASHI T, TAMAGAWA H, et al. FOLFIRI plus bevacizumab as second-line therapy in patients with metastatic colorectal cancer after first-line bevacizumab plus oxaliplatin-based therapy: the randomized phase III EAGLE study [J]. Ann Oncol, 2015, 26 (7): 1427-1433.

[15] BENNOUNA J, SASTRE J, ARNOLD D, et al. Continuation of bevacizumab after first progression in metastatic colorectal cancer (ML18147): a randomised phase 3 trial [J]. Lancet Oncol, 2013, 14 (1): 29-37.

[16] GIANTONIO B J, CATALANO P J, MEROPOL N J, et al. Bevacizumab in combination with oxaliplatin, fluorouracil, and leucovorin (FOLFOX4) for previously treated metastatic colorectal cancer: results from the Eastern Cooperative Oncology Group Study E3200 [J]. J Clin Oncol, 2007, 25 (12): 1539-1544.

[17] TOURNIGAND C, ANDRE T, ACHILLE E, et al. FOLFIRI followed by FOLFOX6 or the reverse sequence in advanced colorectal cancer: a randomized GERCOR study [J]. J Clin Oncol, 2004, 22 (2): 229-237.

[18] FABRIZIO D A, GEORGE T J, DUNNE R F, et al. Beyond microsatellite testing: assessment of tumor mutational burden identifies colorectal cancer who may respond to immune suppression [J]. J Gastrointest Oncol, 2018, 9 (4): 610-617.

[19] VANDERWALDE A, SPETZLER D, XIAO N, et al. Microsatellite instability status determined by next-generation sequencing and compared with PD-L1 and tumor mutational burden in 11, 348 patients [J]. Cancer Med, 2018, 7 (3): 746-756.

[20] VILAR E, GRUBER S B. Microsatellite instability in colorectal cancer-stable evidence [J]. Nat Rev Clin Oncol, 2010, 7 (3): 153-162.

[21] FOSSELLA F V, DEVORE R, KERR R R N, et al. Randomized phase III trial of docetaxel versus vinorelbine or ifosfamide in patients with advanced non-small-cell lung cancer previously treated with platinum-containing chemotherapy regimens. The TAX 320 Non-Small Cell Lung Cancer Study Group [J]. J Clin Oncol, 2000, 18 (12): 2354-2362.

[22] CANON J, REX K, SAIKI A Y, et al. The clinical KRAS (G12C) inhibitor AMG 510 drives anti-tumour immunity [J]. Nature, 2019, 575 (7781): 217-223.

[23] FELL J B, FISCHER J P, BAER B R, et al. Identification of the Clinical Development Candidate MRTX849, a Covalent KRAS (G12C) Inhibitor for the

Treatment of Cancer [J]. J Med Chem, 2020, 63 (13): 6679-6693.

[24] HANNA N, SHEPHERD F A, FOSSELLA F V, et al. Randomized phase III trial of pemetrexed versus docetaxel in patients with non-small-cell lung cancer treated with chemotherapy [J]. J Clin Oncol, 2004, 22 (9): 1589-1597.

[25] WU Y L, LU S, CHENG Y, et al. Nivolumab Versus Docetaxel in a Predominantly Treated Chinese Patient Population With Previously Advanced NSCLC: CheckMate 078 Randomized Phase III Clinical Trial [J]. J Thorac Oncol, 2019, 14 (5): 867-875.

[26] HERBST R S, BAAS P, KIM D W, et al. Pembrolizumab versus docetaxel for previously treated, PD-L1-positive, advanced non-small-cell lung cancer (KEYNOTE-010): a randomised controlled trial [J]. Lancet, 2016, 387 (10027): 1540-1550.

[27] CHENG Y, HAN B, LI K, et al. Effect of anlotinib as a third- or further-line therapy in advanced non-small cell lung cancer patients with histologic different types: Subgroup analysis in the ALTER0303 trial [J]. Cancer Med, 2020, 9 (8): 2621-2630.

[28] HU C, WANG Y, CHEN J, et al. Tumor response and clinical toxicity associated with second-line chemotherapy regimens for advanced non-squamous non-small cell lung cancer: A retrospective cohort study [J]. Thorac Cancer, 2014, 5 (5): 365-376.

[29] MO H, HAO X, LIU Y, et al. A prognostic model for platinum-doublet as second-line chemotherapy in advanced non-small-cell lung cancer patients [J]. Cancer Med, 2016, 5 (6): 1116-1124.

[30] GRIDELLI C, de CASTRO C J, DINGEMANS A C, et al. Safety and Efficacy of Bevacizumab Plus Standard-of-Care Beyond Disease Progression in Randomized Patients With Advanced Non-Small Cell Lung Cancer: The AvaALL Clinical Trial [J]. JAMA Oncol, 2018, 4 (12): e183486.

[31] HERBST R S, O 'NEILL V J, FEHRENBACHER L, et al. Phase II study of efficacy and safety of bevacizumab in combination with chemotherapy or erlotinib compared with chemotherapy alone for treatment of recurrent or refractory small-cell lung cancer [J]. J Clin Oncol, 2007, 25 (30): 4743-4750.

[32] B Garon, Tudor-Eliade Ciulu, Oscar Arrieta, et al. Ramucirumab plus randomised versus placebo plus docetaxel for second-line treatment of stage IV non-small-cell lung cancer after disease progression on platinum-based therapy (REVEL): a

multicentre trial, randomised, phase 3 trial. The Lancet, Edward on line 2014, 6:1-9.

[33] LEE J J J, LIU D D. A predictive probability design for phase II cancer clinical trials [J]. Clin Trials, 2008, 5 (2): 93-106.

13 APPENDICES

Appendix 1 Protocol Revision History

Protocol Revision History

Version No.	Date	Description of Major Changes	Remarks
V1.0	April 15 2021 , Year	Not applicable	Added protocol
V1.1	2021 May 20th Year	• Modify ECG examination requirements;<br>• Revise the expression of exclusion criterion 10;<br>• Addition of strong inhibitors and inducers of common CYP3A4/5 and P-gp and communication between the investigator and the sponsor for questionable concomitant medications;<br>• Adjust blood collection window.	• Enhancing the operability of study procedures.<br>• Improving the comprehensibility of the presentation.<br>• Ensure study quality and enforceability.<br>• Increased enforceability of blood collection procedures.
V1.2	10 December 2021	See Protocol Amendment Record	
V2.0	05 MM 2022 YYYY 01 MM	See Protocol Amendment Record	
V3 .0	Dated 06 Month 07 2022	See Protocol Amendment Record	
V4.0	Month 17 2022 06	See Protocol Amendment Record	
V5.0	26 August, 2022	See Protocol Amendment Record	
V6.0	09-Oct-2022	See Protocol Amendment Record	

Appendix 2 Response Evaluation Criteria in Solid Tumors Version 1.1 (RECIST V1.1)

Note: This translation is for reference only and the final version is based on the English version.

1. measurability of tumor at baseline

1.1 Definition

At baseline, tumor lesions/lymph nodes will be categorized as measurable or non-measurable as follows:

Measurable lesions

Tumor lesions: must be accurately measured in at least one dimension (longest diameter to be recorded) with a minimum length of:

- 10 mm by CT scan (CT scan slice thickness no greater than 5 mm)
- 10 mm by clinical routine examination instrument (tumor lesions that cannot be accurately measured with calipers should be recorded as non-measurable)
- Chest X-ray 20 mm
- Malignant lymph nodes: Pathologically enlarged and measurable lymph nodes must be ≥ 15 mm in short axis on CT scan (CT scan slice thickness recommended to be no greater than 5 mm). At baseline and follow-up, only the short axis will be measured and followed.

Non-measurable lesions

All other lesions, including small lesions (longest diameter < 10 mm or pathological lymph nodes with short axis ≥ 10 mm to < 15 mm) and non-measurable lesions. Lesions that could not be measured included: meningeal disease, ascites, pleural or pericardial effusion, inflammatory breast cancer, lymphangitic carcinomatosis of the skin/lung, abdominal masses that could not be diagnosed and followed by imaging, and cystic lesions.

Special considerations regarding lesion measurement

Bone lesions, cystic lesions and lesions previously treated with local therapy need

to be specified:

Bone lesions:

- Bone scan, PET scan or plain films are not suitable for measuring bone lesions, but can be used to confirm the presence or disappearance of bone lesions;
- Lytic bone lesions or mixed lytic/osteoblastic lesions, with identifiable soft tissue components, that meet the definition of measurability described above may be considered measurable if they can be evaluated by cross-sectional imaging techniques such as CT or MRI;
- Osteoblastic lesions are non-measurable.
- Cystic lesions:
- Lesions that meet the criteria for radiographically defined simple cysts should not be considered as malignant lesions since they are defined as simple cysts and are neither measurable nor non-measurable;
- Cystic metastases that meet the definition of measurability described above may be considered measurable. However, if noncystic lesions are present in the same patient, these are preferred for selection as target lesions.

Locally treated lesions;

- Lesions situated in a previously irradiated area, or in an area subjected to other locoregional therapy, are generally not measurable unless there has been unequivocal progression of the lesion. The protocol should detail the conditions under which such lesions would be considered measurable.

1.2 Description of measurement method

Lesion Measurements

All tumor measurements were recorded in metric notation at the time of clinical evaluation. All baseline assessments of tumor lesion size should be performed as close as possible to the start of treatment and must be performed within 28 days (4 weeks) before the start of treatment.

Evaluation method

The same technique and method should be used for baseline assessment and subsequent measurement of lesions. All lesions must be evaluated by imaging except those that cannot be imaged but can only be evaluated by clinical examination.

Clinical lesions: Clinical lesions will only be considered measurable when they are superficial and ≥ 10 mm in diameter when measured (e.g. skin nodules). For patients with skin lesions, documentation by color photography including a ruler to measure lesion size is recommended. When lesions are evaluated by both imaging and clinical examination, imaging evaluation should be selected whenever possible because imaging is more objective and can be reviewed repeatedly at the end of the study.

Chest X-ray: When tumor progression is an important endpoint, chest CT is preferred because CT is more sensitive than X-ray, particularly for new lesions. Chest X-ray is indicated only when the measured lesion is well circumscribed and the lung is well ventilated.

CT, MRI: CT is currently the best available and reproducible method for response evaluation. This guideline defines measurability based on CT scans with slice thickness ≤ 5 mm. Measurable lesions should be at least twice the slice thickness if the CT slice thickness is greater than 5 mm. Enhanced CT is recommended for response assessment unless contrast is contraindicated. MRI is also acceptable in some cases (e.g., for body scans).

Ultrasound: Ultrasound should not be used as a method of measurement to measure lesion size. Because sonography is operation-dependent, it is not reproducible after the end of measurement and does not ensure the identity of techniques and measurements between different measurements. If new lesions are identified by ultrasound in the course of the study, confirmation by CT or MRI is advised. MRI may be used instead if radiation exposure from CT is taken into account.

Endoscopy, laparoscopy: The use of these techniques for objective tumor evaluation is not recommended, but they can be used to confirm CR when biopsies are obtained or to confirm relapse in studies where the study endpoint is relapse from CR or surgical resection.

Tumor markers: Tumor markers alone cannot be used to evaluate objective tumor response. However, if markers are present at baseline above the upper limit of normal, they must normalize for evaluation of a complete response. Because tumor markers vary

by disease, this factor needs to be taken into account when writing the measurement criteria into the protocol. Specific criteria for CA-125 response (recurrent ovarian cancer) and PSA response (recurrent prostate cancer) have been published. In addition, the Gynecologic Cancer Intergroup has developed CA-125 progression criteria, which are to be added to objective tumor evaluation criteria for first-line treatment regimens for ovarian cancer.

Cytology/histology techniques: These techniques may be used to identify PR and CR in protocol-specified specific situations (eg, residual benign tumor tissue in lesions of germ cell tumors). When effusions are known to be a potential adverse effect of treatment (e.g. with taxane compounds or angiogenesis inhibitors), the cytological confirmation of the neoplastic origin of any effusion that appears or worsens during treatment can be considered if the measurable tumor has met criteria for response or stable disease in order to differentiate between response (or stable disease) and progressive disease.

2. Assessment of tumor response

2.1 Target Lesion Assessment

Complete Response (CR): Disappearance of all target lesions and reduction in short axis of all pathological lymph nodes (including target and non-target nodes) to < 10 mm.

Partial Response (PR): At least a 30% decrease from baseline in the sum of diameters of target lesions.

Progressive Disease (PD): At least a 20% increase in the sum of diameters of target lesions, taking as reference the smallest sum on study (this includes the baseline sum if that is the smallest on study); in addition to the relative increase of 20%, the sum must also demonstrate an absolute increase of at least 5 mm (the appearance of one or more new lesions is also considered progression).

Stable Disease (SD): Neither sufficient shrinkage to qualify for PR nor sufficient increase to qualify for PD, taking as reference the smallest sum diameters while on study.

2.2 Precautions for Target Lesion Assessment

Lymph nodes: Lymph nodes identified as target lesions should always have the actual short axis measurement recorded (measured in the same anatomical plane as the baseline examination), even if the nodes regress to below 10 mm on study. This means

that if lymph nodes are included as target lesions, they cannot be said to have completely disappeared even if complete response criteria are met, since a normal lymph node is defined as having a short axis of < 10 mm. Target nodal lesions will be specifically recorded in the CRF or other modalities at specific locations: for CR, all nodes must have a short axis < 10 mm; for PR, SD and PD, the actual short axis measurement of the target node will be included in the sum of target lesion diameters.

Smaller than non-measurable target lesions: In clinical studies, all lesions (nodal or non-nodal) recorded at baseline should have their actual measurements recorded at each subsequent evaluation, even when very small (eg, 2 mm). However, sometimes lesions or lymph nodes which are recorded as target lesions at baseline become so faint on CT scan that the radiologist may not feel comfortable assigning an exact measure and may report them as being 'too small to measure'. When this occurs, it is important to record a value on the CRF. If it is the opinion of the radiologist that the lesion has likely disappeared, it should also be recorded as 0 mm. If the lesion is believed to be present and is faintly seen but too small to measure, a default value of 5 mm should be assigned. (Note: It is unlikely that lymph nodes will be involved because they usually have a measurable size when normal, or tend to be surrounded by adipose tissue as often as they are in the retroperitoneal space; however, if such lymph nodes do not give a measurement, a default value of 5 mm should be assigned as well). The default value of 5 mm is derived from the cut thickness of the CT scan (this value does not change depending on the different cut thickness values of the CT). Because there is little chance of repeated occurrences of the same measurement, providing this default value will reduce the risk of erroneous evaluation. To reiterate, however, if the radiologist is able to give an exact value for lesion size, the actual value must be recorded even if the lesion diameter is less than 5 mm.

Lesions that split or coalesce: When non-nodal lesions fragment, the longest diameters of the fragmented portions should be added together to calculate the sum of the lesion diameters. Similarly, in the case of combined lesions, the plane between the combined sections was used to distinguish them and then calculate their maximal diameters. However, if the combination is inseparable, the longest diameter should be taken as the longest diameter of the fusion lesion as a whole.

2.3 Assessment of Non-Target Lesions

Criteria for tumor response in non-target lesions are defined in this section. Although some non-target lesions are actually measurable, they do not need to be measured and only qualitatively assessed at protocol-specified time points.

Complete response (CR): disappearance of all non-target lesions and normalization of tumor markers. All lymph nodes were non-pathological in size (< 10 mm short axis).

Non-CR/Non-PD: Persistence of one or more non-target lesion (s) and/or maintenance of tumor marker level above normal.

Progressive disease: Unequivocal progression of existing non-target lesions. Note: The appearance of one or more new lesions is also considered progression.

2.4 Special notes regarding assessment of progression of non-target lesions

The definition of progression of non-target disease is additionally explained as follows: When a patient has measurable non-target disease, to achieve unequivocal progression on the basis of non-target disease, it must be satisfied that the overall extent of worsening of non-target disease has reached the point at which treatment must be discontinued, even if the target lesion is assessed as stable or partially responsive. Because modest increases in the size of one or more non-target lesions tend not to be sufficient to meet the criteria for progression, it is almost rare for overall tumor progression to be defined by changes in non-target lesions alone when target lesions are stable or partially responsive.

When patients have non-measurable non-target disease: This occurs in some Phase 3 studies when it is not specified in the inclusion criteria that measurable disease must be present. The overall assessment is based on the above criteria, but there are no measurable lesions in this case. Because worsening in non-target disease cannot be easily assessed (by definition: if all non-target lesions are truly non-measurable), an effective method of measurement is needed to assess when the change in non-target disease results in an increase in overall disease burden to an extent that corresponds to disease progression in target disease. If described as an increase in tumor burden corresponds to an additional 73% increase in volume (which corresponds to a 20% increase in the diameter of a measurable lesion). Examples include peritoneal exudate ranging from "trace" to "large"; lymphangitic disease ranging from "localized" to "widespread dissemination"; or described as "sufficient to change therapy" in protocols. Examples include pleural

effusions ranging from trace to large, lymphatic involvement spreading from the primary site to distant sites, or may be described in protocols as "necessary to change in treatment". If unequivocal progression is identified, the patient should be considered to have overall disease progression at that time point. Objective criteria are preferred for assessment of non-measurable disease and increased criteria must be reliable.

2.5 New Lesions

The appearance of new malignant lesions denotes disease progression; therefore, some evaluation of new lesions is important. There are no specific criteria for radiographic lesions, however the finding of a new lesion should be unequivocal. For example, progression cannot be attributed to differences in imaging techniques, changes in imaging modality, or lesions other than tumor (e.g., some so-called new bone lesions are simply healing of the original lesion or recurrence of the original lesion). This is important when the patient 's baseline lesions show partial or complete response, for example, necrosis of a liver lesion may qualify as a new cystic lesion on a CT report rather than a new one.

Lesions detected at follow-up that are not detected at baseline will be considered new and will indicate disease progression. An example of this is the patient who has visceral disease at baseline and while on study has a CT or MRI brain ordered which finds metastases, the patient 's brain metastases will be considered evidence of disease progression even if he/she did not have brain ordered at baseline.

If a new lesion is equivocal, for example because of its small size, further treatment and follow-up evaluation will be required to confirm whether it represents a new lesion. If repeat scans confirm there is definitely a new lesion, then progression should be declared using the date of the initial finding.

FDG-PET assessment of lesions generally requires additional testing for supplemental confirmation, and it is reasonable to combine FDG-PET testing and supplemental CT testing to assess progression (particularly for new suspected disease). New lesions may be identified by FDG-PET according to the following procedures:

Negative FDG-PET at baseline and positive FDG-PET at the next follow-up visit indicates disease progression.

No FDG-PET at baseline and a positive FDG-PET at follow-up:

If the positive FDG-PET at follow-up corresponds to a new site of disease on CT, this is PD.

If a positive FDG-PET at follow-up is not confirmed as a new site of disease on CT, additional follow-up CT scans are needed to confirm this (if so, the date of PD will be the date of the initial abnormal FDG-PET scan).

If the positive FDG-PET at follow-up corresponds to a lesion already present on CT that is not progressing on imaging, there is no disease progression.

2.6 Missing assessments and non-evaluable designation

If lesion imaging or measurement is not available at a particular time point, the patient is not evaluable at that time point. If only a subset of lesion measurements are made at an assessment, usually the case is also considered NE at that time point, unless a convincing argument can be made that the contribution of the individual missing lesion (s) would not change the assigned time point response.

2.7 Special Reminders for Efficacy Assessments

When nodal lesions are included in the overall target lesion assessment and the size of the nodule decreases to "normal" size (< 10 mm), they will still have a lesion size scan report. To avoid overestimation based on increased nodule size, measurements will be recorded even if the nodule is normal. As mentioned earlier, this means that subjects with CR will not have a total sum of 'zero' on the CRF.

Repeated "non-measurable" time points complicate best response assessments if confirmation of response is required during the study. The analysis plan for the study must state that these missing data/assessments can be accounted for in the determination of efficacy. For example, in most studies, response to PR-NE-PR may be confirmed in a subject.

Subjects with a global deterioration of health status requiring discontinuation of treatment without objective evidence of disease progression should be reported as having symptomatic progression. Every effort should be made to assess objective progression even after treatment discontinuation. Symptomatic deterioration is not a descriptor of an objective response and is the reason for stopping treatment. The objective response status of such subjects will be assessed by target and non-target lesions as shown in Attached Tables 1 – 3.

Conditions defined as early progression, early death, and non-evaluability are study specific and should be clearly described in each protocol (depending on treatment interval and treatment cycle).

In some cases, it may be difficult to discern local disease from normal tissue. When complete response assessment is based on such a definition, biopsy is recommended prior to response assessment for complete response of local disease. FDG-PET may be used to confirm a response to a CR in a manner similar to a biopsy in cases where a local radiographic abnormality is thought to represent fibrosis or scarring. In such cases, the use of FDG-PET should be prospectively described in the protocol and supported by specialist medical literature for the indication. It must be acknowledged, however, that both approaches may lead to false positive CR due to limitations of FDG-PET and biopsy resolution and sensitivity.

Attached Table1 . Time Point Response - Subjects with Target Lesions (Including or Excluding Non-Target Lesions)

Target lesions	Non-target lesions	New Lesion	Overall response
CR	CR	None	CR
CR	Non-CR/Non-PD	None	PR
CR	Not evaluable	None	PR
PR	Non-progressive or not fully evaluable	None	PR
SD	Non-progressive or not fully evaluable	None	SD
Not fully evaluated	Non-Progression	None	NE
PD<br>Any condition<br>Any condition	Any condition<br>PD<br>Any condition	Yes or No<br>Yes or No<br>Yes	PD<br>PD<br>PD

Note: CR = complete response, PR = partial response, SD = stable disease, PD = progressive disease, NE = not evaluable.

Attached Table2 . Time Point Response – Subjects with Non-Target Lesions Only

Non-target lesions	New Lesion	Overall response
CR	None	CR
Non-CR or Non-PD	None	Non-CR or Non-PD
Not fully evaluated	None	Not evaluable

Unequivocal PD	Yes or No	PD
Any condition	Yes	PD

Note: For non-target lesions, 'non-CR/non-PD' refers to efficacy superior to SD. Because SD is increasingly used as an endpoint to evaluate efficacy, a response of non-CR/non-PD is established to target when no lesions are measurable.

For equivocal findings of progression (eg, very small indeterminate new lesions; cystic degeneration or necrotic lesions in existing lesions), treatment may continue until the next assessment. If at the next assessment disease progression is confirmed, the date of progression should be the earlier date when progression was suspected.

Attached Table3 . Best Overall Response Confirmed for CR and PR

**Overall response at first time point**	**Overall response at later time points**	**Best overall response**
CR	CR	CR
CR	PR	SD, PD or PRa
CR	SD	SD provided SD persists for sufficient duration, otherwise PD
CR	PD	SD provided SD persists for sufficient duration, otherwise PD
CR	NE	SD provided SD persists for sufficient duration, otherwise NE
PR	CR	PR
PR	PR	PR
PR	SD	SD
PR	PD	SD provided SD persists for sufficient duration, otherwise PD
PR	NE	SD provided SD persists for sufficient duration, otherwise NE
NE	NE	NE

Note: CR means complete response, PR means partial response, SD means stable disease, PD means progressive disease, and NE means not evaluable. Superscript "a": If a CR is truly met at first time point, then any disease seen at a subsequent time point, even disease meeting PR criteria relative to baseline, makes the disease PD at that point (since disease will reappear after CR). Best response depends on whether SD occurs within the shortest treatment interval. However, sometimes' CR 'may be claimed at the first assessment, but subsequent scans at time points suggest small lesions still appear

to be present and in fact the subject should have achieved PR rather than CR at the first time point. In this case, the first CR should be changed to PR and the best response is PR.

2.8 Efficacy Assessments/Confirmation of Response

Confirmation of efficacy

For non-randomized clinical studies where tumor response is the primary endpoint, confirmation of PR and CR is required to ensure that response is not the result of evaluation error. In studies where stable disease or disease progression are the primary endpoints, confirmation of response is no longer required as it would not be valuable for interpretation of study results. In the case of SD, at least 1 measurement met the SD criteria as specified in the protocol at the minimum interval after the start of the study (generally no less than 6 to 8 weeks).

Overall Response Period

Overall response is measured from the time measurement criteria are first met for CR or PR (whichever is first recorded) until the first date that recurrent or progressive disease is objectively documented (taking as reference for progressive disease the smallest measurements recorded on study). The duration of overall complete response is measured from the time measurement criteria are first met for CR until the first date that recurrent or progressive disease is objectively documented.

Stable disease

Time from start of treatment to disease progression (in randomized studies, from time of randomization) taking as reference the smallest sum on study (if the baseline sum is the smallest, this is the reference for PD calculation). The clinical relevance of stable disease varies between studies and diseases. If the proportion of patients achieving stable disease for a minimum period of time is an endpoint in a particular study, the protocol should specify the minimum time interval between two measurements in the definition of SD.

Note: The duration of response, stabilization, and PFS are influenced by the frequency of follow-up after baseline evaluation. Defining a standard follow-up frequency is outside the scope of this guideline. The frequency of follow-up should take into account many factors, such as disease type and stage, treatment cycle and standard practice. However, limitations in the accuracy of these endpoints should be taken into

account if comparisons between studies are needed.

2.9 PFS/TTP

Many studies in advanced cancer have used PFS or TTP as primary endpoints. If the protocol requires that all patients have measurable disease, evaluation of progression is relatively straightforward. An increasing number of studies have allowed patients with measurable disease and patients without measurable disease to enter the study. In such cases, clinical findings of disease progression in patients without measurable disease must be described in detail and clearly. Because progression dates often deviate from established deviations, observation timepoints should be scheduled the same for each study arm.

Appendix 3 Performance Status Criteria (ECOG PS)

Score	Criteria
0	Fully active, able to carry on all pre-disease performance without restriction
1	Ambulatory and able to carry out work of a light or sedentary nature, eg, light housework or office work, but unable to carry out work of a heavy or sedentary nature
2	Ambulatory and capable of all self-care but unable to carry out any work activities; up and about more than 50% of waking hours
3	Capable of only limited self-care, confined to bed or chair more than 50% of waking hours
4	Bedridden, unable to carry on any selfcare
5	Death

Appendix 4 Statistical Performance of Bayesian Optimal Interval (BOIN) Design

Table A-1 presents the data generated by the BOIN online software (Fig. http://www.trialdesign.org) to simulate the statistical performance obtained from 10,000 BOIN trial designs. The results of this simulation indicate that if the MTD exists, the BOIN design has a high probability of selecting the true MTD and allocating more subjects to the dose level closest to the target toxicity rate 0.3 .

Table A-1. Statistical Performance of Bayesian Optimal Interval (BOIN) Design

	**Dose 1**	**Dose 2**	**Dose 3**	**Dose 4**	**Dose 5**	**Dose 6**	**Dose 7**	**Dose 8**	**Mean Sample Size**	**Early Termination Percent%**
**Scenario 1**										
True DLT Rate	0.3	0.45	0.49	0.52	0.56	0.6	0.63	0.67	14.9	12.53
Dose Selection %	65.61	17.8	3.41	0.57	0.07	0.01	0	0		
% Subjects	58.9	31.8	7.4	1.5	0.3	0	0	0		
**Scenario 2**										
True DLT Rate	0.13	0.3	0.47	0.51	0.56	0.6	0.65	0.7	21.3	0.57
Dose Selection %	23.15	57.71	15.57	2.64	0.31	0.05	0	0		
% Subjects	32.4	42.1	20.3	4.3	0.7	0.1	0	0		
**Scenario 3**										
True DLT Rate	0.05	0.15	0.3	0.45	0.49	0.53	0.57	0.62	24.9	0.02
Dose Selection %	1.73	24.76	53.13	16.69	3.09	0.49	0.07	0.02		
% Subjects	15.8	28.2	34.1	16.8	4.1	0.8	0.1	0		
**Scenario 4**									28.2	0.01

True DLT Rate	0.06	0.1	0.14	0.3	0.44	0.53	0.61	0.69		
Dose Selection %	1.23	4.17	22.49	51.5	17.9	2.46	0.23	0.01		
% Subjects	13.4	15.7	23.3	28.9	14.8	3.4	0.5	0		
**Scenario 5**										
True DLT Rate	0.05	0.07	0.09	0.11	0.3	0.44	0.57	0.7		
Dose Selection %	0.58	1.4	3	18.68	54.3	20.14	1.88	0.01	31.4	0.01
% Subjects	11.3	12.2	13	19.7	26.6	13.9	3	0.3		
**Scenario 6**										
True DLT Rate	0.05	0.07	0.09	0.11	0.12	0.3	0.48	0.7		
Dose Selection %	0.67	1.53	2.92	4.75	19.61	54.64	15.6	0.28	33.8	0
% Subjects	10.6	11.3	12	12.5	17.7	23.4	10.9	1.5		
**Scenario 7**										
True DLT Rate	0.05	0.07	0.09	0.1	0.12	0.14	0.3	0.48		
Dose Selection %	0.66	1.51	2.97	3.73	5.67	20.87	49.37	15.2	35.8	0.02
% Subjects	9.9	10.7	11.3	11.5	11.9	16	19.5	9.2		
**Scenario 8**										
True DLT Rate	0.03	0.04	0.06	0.07	0.09	0.1	0.12	0.3		
Dose Selection %	0.16	0.24	1.06	1.36	3.06	3.6	19.38	71.14	35.7	0
% Subjects	9.2	9.6	10.3	10.6	11.3	11.3	16.1	21.5		

Note: "% Early Termination" refers to an early termination trial triggered by excessive toxicity.

**Test Parameter**	**Value**
Number of doses	8

Starting dose	1
Max sample size	45
Cohort size	3
Stop trial if # assigned patients to single dose reaches reaches reaches this number (stop trial if number of patients on a single dose)	9
Use accelerated titration	FALSE (No)
Target probability toxicity	0.3
Use the default alternatives to minimize decision errors	TRUE (Yes)
Alternative high toxicity for optimization	Default Value
Alternative low toxicity for optimization	Default Value
Eliminate dose threshold (critical probability value excluding toxic dose, pE)	0.95
Impose a more stringent safety stopping rule: (stricter stopping rule)	FALSE (No)
Require the isotonic estimate of the DLT probability for the dose selected as the MTD less than the de-escalation boundary (Requires ordinal regression estimates of toxicity rates below dose reduction margins) FALSE (No)	
Number of repetitions per scenario	10000
Random number generator seed	2021

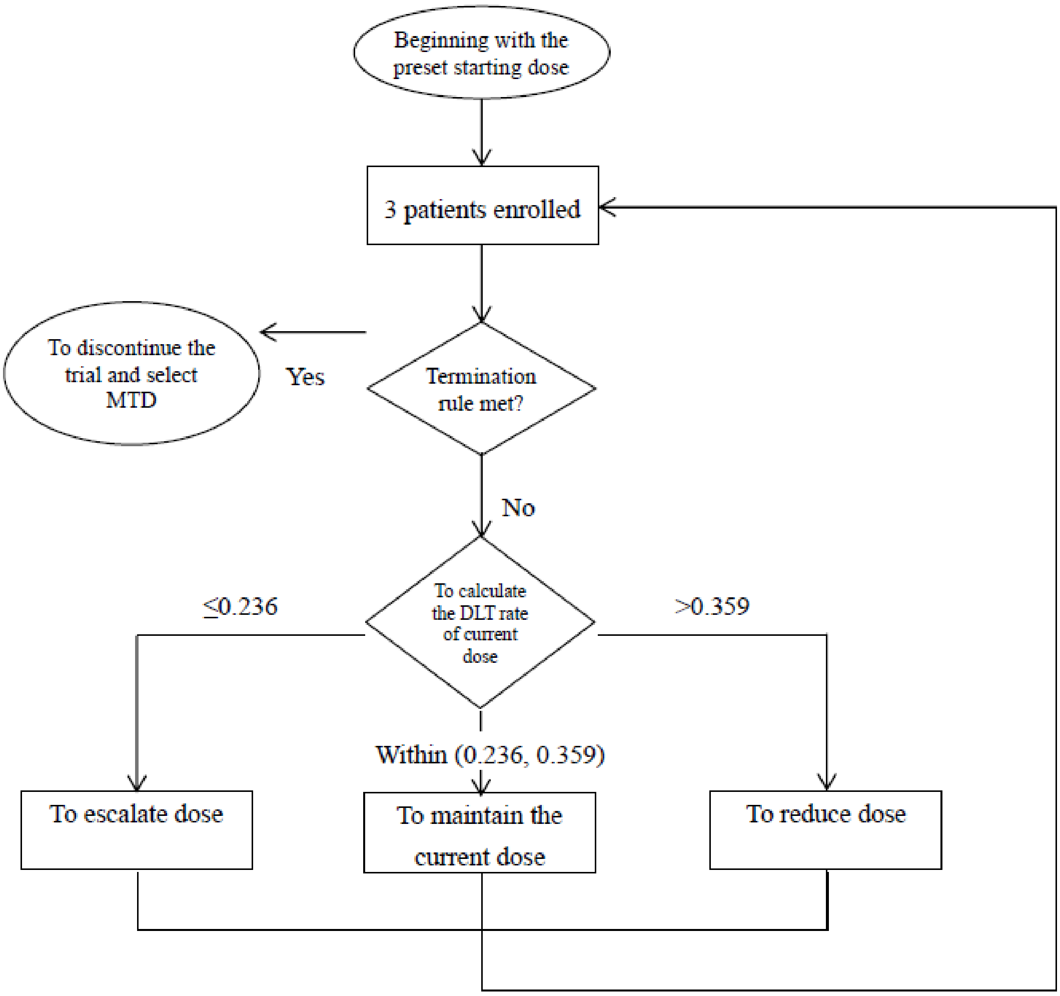

$$* \text{ DLT rate} = \frac{\text{Number of patients who have experienced DLT at the current dose}}{\text{Total number of patients that can be evaluated at the current dose}}$$

Figure A-1. Flow Chart for Bayesian Optimal Interval (BOIN) Design

Appendix 5 Cockcroft-Gault Formula

Formula for calculating serum creatinine concentration in mg/dL:

$$\text{Creatinine clearance (mL/min) for males} = \frac{(140\text{-age}) \times (\text{body weight})^{a}}{72 \text{ x serum creatinine}}$$

$$\text{Creatinine clearance in females (mL/min)} = \frac{0.85 \text{ x } (140\text{-age}) \text{ x } (\text{weight})^{a}}{72 \text{ x serum creatinine}}$$

Formula for calculating serum creatinine concentration inm mol/L:

$$\text{Creatinine clearance (mL/min) for males} = \frac{(140\text{-age}) \times (\text{body weight})^{a}}{0.818 \text{ x serum creatinine}}$$

$$\text{Creatinine clearance in females (mL/min)} = \frac{0.85 \text{ x } (140\text{-age}) \text{ x } (\text{weight})^{a}}{0.818 \text{ x serum creatinine}}$$

A: Age in years and weight in kg.

Appendix 6 List of Concomitant Therapies Prohibited Agents

Interaction mechanism	Drug name
CYP3A4 AND Strong P-gp inducers	Carbamazepine, phenytoin, rifampin, St. John 's Wort, enzalutamide, apalutamide, mitotane
CYP3A4 AND Strong P-gp inhibitors	Boldenavir, clarithromycin, cobicistat, grapefruit juice (> 1 L/day, high concentration), indinavir, itraconazole, ketoconazole, lopinavir and ritonavir, nefazodone, nelfinavir, posaconazole, quinupristin, ritonavir, saquinavir, telaprevir, telithromycin, voriconazole, amiodarone, carvedilol, diltiazem, dronedarone, glecaprevir, lamitententan, ledipasaprevir, ledipasvir, quinidine, ranolazine, cimeprevir, ticagrelor, verapamil, verapamil, vanciclosporin, voriconavir, danoprevir, elvitegravir, olaprevir and/or dasabuvir, tipranavir, tilazaprevir, troglitamprenavir, troglumycin, valprothromycin, ivalarix, lapatinib propafenone
P-gp sensitive substrate	Dabigatran etexilate, digoxin
BCRP sensitive substrate	Sulfasalazine, rosuvastatin
Sensitive CYP2D6 Substrates	Dextromethorphan, tolterodine, metoprolol, desipramine, amphetamines, aripiprazole, atomoxetine, desipramine, iloperidone, nebivolol, perphenazine, risperidone, timolol, tolterodine, venlafaxine, vortioxetine, thioridazine, eliglukast, nortriptyline, encainide, imipramine, propranolol, tramadol, trimipramine, venlafaxine
CYP3A4 Sensitive Substrates	Levonorgestrel (LNG) and ulipristal acetate (UPA), ivacaftor, abeciclib, midostaurin, neratinib, felodipine, midazolam, buspirone, sildenafil, simvastatin, budesonide, fluticasone, nifedipine, tolvaptan, isavuconazole, ivonib, apalutamide, cobimetinib, temsirolimus, triazolam, eplerenone, alprazolam, almotriptan, apremilast, nisoldipine, avanafil, roflumilast, bromocriptine mesylate, eszopiclone, flibanserin, isradipine, alprazolam, apretan, atorvastatin, colchicine, rilpivirine, rivaroxaban, tadalafil, Pimozide, alfentanil, conivaptan, darifenacin, dargravir, ebastine, everolimus, ibrutinib, lomitapide, lovastatin, sodium Nisoldipine, sirolimus, tacrolimus, edenafil,

	dasatinib, eletriptan, lurasidone, maraviroc, guatiapine, ticagrelor, linagliptin, pethidine, tamoxifen

Listed in the table are common potent inhibitors and inducers of CYP3A4 and P-gp , CYP2D6 and CYP3A4 , P-gp and B CRP sensitive substrates. If there are questions about other possible concomitant medications, the investigator may communicate with the sponsor sponsor .

[redacted]

[redacted]

[redacted]

[redacted].

[redacted]

[redacted]	[redacted]
[redacted]	
[redacted]	[redacted]
[redacted]	[redacted]
[redacted]	[redacted]
[redacted]	[redacted]
[redacted]	[redacted]
[redacted]	

[redacted]	[redacted]
[redacted]	[redacted]
[redacted]	[redacted]
[redacted]	[redacted]
[redacted]	
[redacted]	[redacted]

[redacted]

[redacted]	[redacted]	[redacted]
[redacted]	[redacted]	[redacted]

[redacted]

[redacted]	[redacted]	[redacted]	[redacted]	[redacted]	[redacted]	[redacted]
[redacted]	[redacted]	[redacted]	[redacted]	[redacted]	[redacted]	[redacted]
[redacted]	[redacted]	[redacted]	[redacted]	[redacted]	[redacted]	[redacted]

[redacted]

[redacted]				[redacted]		
[redacted]	[redacted]	[redacted]	[redacted]	[redacted]	[redacted]	[redacted]
[redacted]	[redacted]	[redacted]	[redacted]	[redacted]	[redacted]	
[redacted]	[redacted]	[redacted]	[redacted]	[redacted]	[redacted]	[redacted]
[redacted]	[redacted]	[redacted]	[redacted]	[redacted]	[redacted]	[redacted]
