# Supplementary material for: Efficacy and safety of IBI351 (fulzerasib) monotherapy in KRASG12C inhibitor-naïve Chinese patients with KRASG12C-mutated metastatic colorectal cancer: a pooled analysis from phase I part of two studies
Source: Signal Transduct Target Ther. 2025 Jul 25;10:241. doi: 10.1038/s41392-025-02315-7 (PMC12297441; doi:10.1038/s41392-025-02315-7)
Supplement: Supplementary file 3 — Protocol for NCT05497336 [file 41392_2025_2315_MOESM3_ESM.pdf]

Clinical Study Protocol

**Study Title**	An Open-label, Multicenter, Phase Ib/III Study to Evaluate the Efficacy and Safety of IBI351 in Combination with Cetuximab in Patients with Metastatic Colorectal Cancer with KRAS G12C Mutation
**Protocol Number:**	CIBI351B301
**Version date and version number:**	Jul 18 2022/Version 2.0
**Product Name:**	IBI351
**Study Phase:**	Phase Ib/III
**Sponsor:**	Innovent Biologics (Suzhou) Co., Ltd<br>No. 168 Dongping Street, Suzhou Industrial Park, Jiangsu Province, China
**Sponsor Contact:**	[redacted]<br>[redacted]<br>[redacted]

Confidentiality Statement

This document contains the confidential information of Innovent Biologics (Suzhou) Co., Ltd.

The content of this document shall not be disclosed to any person other than the Investigators, research consultants or related personnel, and Institutional Review Board/Independent Ethics Committee.

The information contained in this document must not be used for any purpose without the written consent of the Sponsor, except for the evaluation or conduct of this study.

Sponsor Signature Page

Study Title: An Open-label, Multicenter, Phase Ib/III Study to Evaluate the Efficacy and Safety of IBI351 in Combination with Cetuximab in Patients with Metastatic Colorectal Cancer with KRAS G12C Mutation

Protocol No.: CIBI351B301

Title	Name	Signature	Date
[redacted]	[redacted]	______________________	______________________
[redacted]	[redacted]	______________________	______________________

Investigator Signature Page

Study Title: An Open-label, Multicenter, Phase Ib/III Study to Evaluate the Efficacy and Safety of IBI351 in Combination with Cetuximab in Patients with Metastatic Colorectal Cancer with KRAS G12C Mutation

Protocol Number: CIBI351B301

This protocol is a confidential document of Innovent Biologics (Suzhou) Co., Ltd. I have read through and fully understand this protocol and will conduct this study in accordance with its requirements and Good Clinical Practice. Furthermore, I will abide by the ethics principles in Declaration of Helsinki and GLP and applicable laws and regulations during the study. I promise not to disclose any confidential information in this study to any third party without the written consent of Innovent Biologics (Suzhou) Co., Ltd.

Instructions for Investigators:<br>Please sign and date this signature page, print the Investigator's name and title, site name/address, and send it back to Innovent Biologics (Suzhou) Co., Ltd. after signing.

I have read the entire contents of this protocol and warrant that this study will be conducted as required:

Signature of Investigator: ____________________ Date ________________

Printed Name: ____________________

Investigator Title: ____________________

Tel.: ____________________

Site Name/Address: ____

____

____

Protocol Summary

<table>
<tr><td>Protocol Number</td><td>CIBI351B301</td></tr>
<tr><td>Sponsor</td><td>Innovent Biologics (Suzhou) Co., Ltd.</td></tr>
<tr><td>Study drug</td><td>IBI351</td></tr>
<tr><td>Active ingredient</td><td>GFH925</td></tr>
<tr><td>Study Title</td><td>An Open-label, Multicenter, Phase Ib/III Study to Evaluate the Efficacy and Safety of IBI351 in Combination with Cetuximab in Patients with Metastatic Colorectal Cancer with KRAS G12C Mutation</td></tr>
<tr><td>Study Phase</td><td>Phase Ib/III</td></tr>
<tr><td>Study Objectives, Endpoints and Estimands:</td><td>This is a Phase Ib/III study.
Phase Ib Objectives, Endpoints, and Estimands:
<table>
<tr><th>Study Objectives</th><th>Study Endpoints</th></tr>
<tr><td>Primary Objectives</td><td>Primary Endpoints</td></tr>
<tr><td>• [redacted]
• To evaluate the efficacy of IBI351 as a single agent or in combination with cetuximab in metastatic colorectal cancer with KRAS G12C mutation in terms of Objective Response Rate (ORR).</td><td>• [redacted]
• Objective response rate (ORR) during the dose expansion phase as assessed by the investigator according to RECIST v1.1 criteria.</td></tr>
<tr><td>Secondary Objectives</td><td>Secondary Endpoints</td></tr>
<tr><td>• To assess the safety/tolerability of IBI351 as a single agent or in combination with cetuximab for the treatment of metastatic colorectal cancer with KRAS G12C mutation;</td><td>• Incidence, relatedness and severity of treatment-emergent adverse events (TEAE), treatment-related adverse events (TRAE) and serious adverse events (SAE) (CTCAE v5.0 criteria). Changes in vital signs, laboratory test results</td></tr>
</table>
</td></tr>
</table>

	• To characterize the pharmacokinetics (PK) of IBI351 as a single agent or in combination with cetuximab in metastatic colorectal cancer with KRAS G12C mutation;<br>• To evaluate additional efficacy of IBI351 as a single agent or in combination with cetuximab in metastatic colorectal cancer with KRAS G12C mutation.	and 12-lead ECG values before, during and after study treatment;<br>• Pharmacokinetics: Pharmacokinetic parameters of subjects in the test group, including but not limited to maximum concentration (C_{max}), area under the drug concentration-time curve (AUC), half-life ($t_{1/2}$), clearance (CL/F) and volume of distribution (V/F);<br>• Progression Free Surival (PFS) assessed by the investigator according to RECIST v1.1 criteria;<br>• Overall survival (OS);<br>• Disease Control Rate (DCR), Duration of Response (DoR) and Time to Response (TTR) assessed by the investigator according to RECIST v1.1 criteria.
	**Exploratory Objectives**	**Exploratory Endpoints**
	• To explore potential mechanisms of IBI351 resistance.	• Relationship between gene mutations/fusions in tumor tissues of subjects at baseline and efficacy.
	[redacted]<br>[redacted]<br>• [redacted]<br>• [redacted].	

- [redacted]
- [redacted]

[redacted]		
[redacted]	[redacted]	[redacted]
[redacted]	[redacted]	[redacted]
[redacted]	[redacted]	[redacted]

- [redacted]

(2) Primary Estimand 2 (ORR assessed by investigator per RECIST v1.1 during the dose expansion phase)

The primary clinical concern was the ORR (as assessed by the investigator per RECIST v1.1) in subjects treated for KRAS G12C mutant metastatic colorectal cancer, regardless of the impact of treatment discontinuation for any reason.

Definition of Estimand:

- [redacted].

- Endpoint: Confirmed Complete Response (CR) or Partial Response (PR) according to RECIST v1.1 criteria prior to initiation of new antineoplastic therapy, first disease progression, or death due to any cause, whichever occurs first.
- Treatment: IBI351 (Maximum Administered Dose (MAD) [if Maximum Tolerated Dose (MTD) or MTD is not reached]) alone or in combination with cetuximab until disease progression, intolerable toxicity, withdrawal of consent, or other reason for discontinuation of study treatment, whichever occurs first.
- [redacted]

[redacted]		
[redacted]	[redacted]	[redacted]
[redacted]	[redacted]	[redacted]
[redacted]	[redacted]	[redacted]

- Population level summary: the proportion of subjects with CR/PR in the analysis population (ORR) and confidence interval will be calculated.
- Considerations for the selection of this estimand: ORR is a reliable reflection of the antitumor activity of a drug and is a common surrogate endpoint in exploratory studies. A hypothetical strategy for starting a new antineoplastic therapy is to consider that the new antineoplastic therapy will affect the assessment of the therapeutic effect of interest.

Phase 3 Study Objectives, Endpoints, and Estimands:

[redacted]	[redacted]
[redacted]	[redacted]

	• [redacted]	[redacted]
	[redacted]	[redacted]
[redacted]	[redacted]	[redacted]
	[redacted]	[redacted]s

• [redacted]	[redacted]

[redacted]ies:

[redacted]	[redacted]	[redacted]
[redacted]	[redacted]	[redacted]

		[redacted]
[redacted]	[redacted]	[redacted]
[redacted]	[redacted]	[redacted]

- [redacted]

[redacted]

[redacted]	[redacted]	[redacted]
[redacted]	[redacted]	[redacted]

<table>
<tr>
<td></td>
<td>
<table>
<tr><td></td><td>[redacted]</td><td></td></tr>
<tr><td>[redacted]</td><td>[redacted]</td><td>[redacted]</td></tr>
</table>
[redacted]
[redacted].
</td>
</tr>
<tr>
<td>Study Design</td>
<td>
Phase Ib
The recommended Phase II dose (RP2D) has been determined to be 600mg BID based on comprehensive analysis of PK, efficacy and safety of IBI351 monotherapy in a previous dose escalation study. This Phase Ib study is to explore the safety, tolerability and preliminary efficacy of IBI351 in combination with cetuximab in metastatic colorectal cancer with KRAS G12C mutation.
[redacted]
Phase Ib consists of the following three cohorts:
[redacted]
[redacted]
Cohort C: Subjects with metastatic colorectal cancer harboring the KRAS G12C mutation who have failed or are intolerant to or refuse systemic therapy.
[redacted]
</td>
</tr>
</table>

	[redacted]<br>[redacted]<br>[redacted] i[redacted]<br>Cohort C will be treated with IBI351 600 mg BID monotherapy without dose escalation and will be expanded to 10-30 subjects for preliminary efficacy evaluation. Subjects will receive IBI351 until disease progression, intolerable toxicity, withdrawal of consent, or other reason for discontinuation of study treatment, whichever occurs first.<br>[redacted]<br>[redacted]<br>[redacted]<br>[redacted]<br>[redacted]<br>[redacted]

	[redacted]. [redacted]

	[redacted].<br>In the Phase Ib study, the investigator will evaluate the response according to RECIST v1.1, and in the Phase III study, the independent imaging review committee (IRRC) and the investigator will evaluate the response according to RECIST v1.1, respectively. Subjects will undergo tumor imaging assessments every 6 weeks (± 7 days) for 48 weeks after the first dose (Phase Ib) or randomization (Phase III), and then every 12 weeks (± 7 days) until disease progression, withdrawal of consent, lost to follow-up, death, or study termination, whichever occurs first. An End-of-Treatment Visit will be performed as soon as possible after the end of study treatment or premature discontinuation of study drug for any reason. Safety Follow-up will occur 30 ± 7 days after the last dose and will then enter Survival Follow-up (every 12 weeks ± 7 days).
**Planned sample size**	Phase Ib: 39-102<br>[redacted]
**Inclusion Criteria**	**Subjects were required to meet all of the following criteria to participate in this study:**<br>1. Voluntary participation in the study and signed informed consent.<br>2. Male or female ≥ 18 years of age and ≤ 75 years of age.<br>3. Have histologically or cytologically pathologically confirmed unresectable metastatic colorectal cancer.<br>4. Subjects in Phase Ib:<br>[redacted];<br>[redacted]<br>Cohort C: Subjects with metastatic colorectal cancer harboring KRAS G12C mutation who have failed systemic therapy or are intolerant to systemic therapy or who refuse systemic therapy;<br>[redacted]

	[redacted]<br>5. [redacted]<br>6. [redacted]<br>7. Have at least one measurable lesion (per RECIST v1.1 criteria).<br>8. Have adequate organ function including:<br>• Adequate hematopoiesis as defined by absolute neutrophil count (ANC) ⩾ 1.5 × 10^9/L, platelet count ⩾ 100 × 10^9/L, and hemoglobin ⩾ 9 g/dL. Blood transfusion or treatment with granulocyte colony-stimulating factor, thrombopoietin, erythropoietin, etc. are not allowed within 14 days before blood routine examination.<br>• Adequate liver function, i.e., total bilirubin (TBIL) < 1.5 × Upper Limit of Normal Value (ULN), aspartate aminotransferase (AST) and alanine aminotransferase (ALT) < 2.5 × ULN; If Gilbert's syndrome, total bilirubin < 2 × ULN; In case of liver metastasis, AST and ALT should be < 5.0 × ULN; Direct bilirubin (DBIL) < 3.0 × ULN is allowed if it suggests extrahepatic obstruction. Albumin ⩾ 30 g/L.<br>• Adequate renal function as defined by creatinine (Cr) ⩽ 1.5 × ULN, or calculated creatinine clearance (CrCl) ⩾ 60 mL/min using the Cockcroft-Gault formula when Cr > 1.5 × ULN.<br>9. Toxicities caused by previous anti-tumor treatment should be recovered to CTCAE grade ⩽ 1 before enrollment (except for alopecia, the values specified in the inclusion criteria have been met, or the toxicity of grade 2 that is clinically stable and does not affect the safety of study drug treatment as determined by the investigator).<br>10. Eastern Cooperative Oncology Group (ECOG) performance status (PS)

	0-1.<br>11. Expected survival time ≥ 12 weeks.<br>12. Female or male subjects of childbearing potential must agree to use an effective method of contraception from signing of informed consent until 6 months after the last dose of study drug. Female subjects of childbearing potential should have a negative blood pregnancy test within 7 days prior to dosing (inclusive).<br>13. The investigator judges that the subject can communicate well, follow up on schedule, and complete the study in accordance with the protocol.
**Exclusion Criteria**	**Subjects who meet any of the following criteria will not be enrolled in the study:**<br>1. [redacted]<br>2. Known central nervous system metastases or known leptomeningeal disease.<br>3. Any cerebral arterial thromboembolic event, such as cerebrovascular accident or transient ischemic attack, occurred within 6 months prior to treatment.<br>4. History of deep vein thrombosis or any other serious thromboembolism within 3 months prior to enrollment. (Implantable venous access port or catheter-derived thrombosis, or superficial venous thrombosis would not be considered as "serious" thromboembolism).<br>5. Have significant cardiovascular disease, such as:<br>• Patients who have had definite abnormal cardiovascular events within 6 months, such as myocardial infarction, angina pectoris, heart failure, severe arrhythmia, or have undergone angioplasty, vascular stent implantation, coronary artery bridging surgery, etc.;<br>• Clinically significant QT/QTcF interval prolongation (QTcF > 470ms for females or > 450ms for males).<br>6. History of radiation pneumonitis, idiopathic pneumonitis, active pneumonitis, pulmonary fibrosis, diffuse interstitial lung disease, or organizing pneumonia (eg, bronchiolitis obliterans).<br>7. Presence of significant gastrointestinal diseases, such as intractable hiccups, nausea, vomiting, severe gastrointestinal ulcers, liver cirrhosis, active gastrointestinal bleeding, inflammatory bowel disease leading to prolonged diarrhea (such as colitis or Crohn's disease, etc.) or other diseases that affect the swallowing of tablets or significantly affect the absorption of oral drugs.

	8. Esophageal or gastric varices requiring immediate intervention (e.g., banding or sclerotherapy) or evidence of portal hypertension that is considered to be at high risk of bleeding in the opinion of the investigator.<br>9. Subjects with a risk of intestinal obstruction (excluding bowel obstruction that has been surgically cured or completely resolved) or intestinal perforation (including but not limited to history of acute diverticulitis, abdominal abscess, abdominal cancer) within 28 days prior to the first dose of this study.<br>10. Concomitant with other poorly controlled systemic diseases, such as uncontrolled hypertension (systolic blood pressure ≥ 150 mmHg or diastolic blood pressure ≥ 100 mmHg) despite standard treatment, diabetes, etc.<br>11. Have significant acute or chronic infections, including:<br>• Active infection requiring systemic treatment.<br>• Patients with positive hepatitis B surface antigen (HBsAg) or hepatitis B core antibody (HBcAb) should be tested for hepatitis B virus (HBV) DNA. If the HBV DNA copy number is ≤ 2.5×10^3 copies/ml or ≤ 500 IU/ml or below the lower limit of detection, they can be enrolled. Acute or chronic active Hepatitis C Virus (HCV), i.e., HCV antibody positive and HCV RNA level above the lower limit of detection. Human immunodeficiency virus antibody (HIV-Ab) positive.<br>• Active pulmonary tuberculosis.<br>12. Other malignancies within 2 years prior to study entry, with the exception of adequately treated carcinoma in situ of the cervix, focal squamous cell carcinoma of the skin, basal cell carcinoma, prostate cancer not requiring treatment, ductal carcinoma in situ of the breast, and superficial non-muscle invasive urothelial carcinoma.<br>13. Prior treatment with a KRAS G12C inhibitor, cetuximab, or other anti-EGFR antibody or small molecule EGFR TKI (e.g., erlotinib, etc.). Note: Cohort C allows enrollment of subjects who have previously received cetuximab.<br>14. [redacted]<br>15. Known contraindication to study treatment:<br>• [redacted]

	[redacted]<br>16. Has had a surgical procedure (excluding punch biopsy) within 28 days prior to enrollment in this study that may affect the administration of study drugs or study assessments.<br>17. Received chemotherapy, targeted therapy, endocrine therapy, immunotherapy, other investigational drug or investigational device therapy within 28 days or 5 half-lives (whichever is shorter) prior to dosing in this study, with the exception of maintenance endocrine therapy.<br>18. Therapeutic or palliative radiation therapy within 14 days prior to dosing in this study.<br>19. Has received potent inhibitors or inducers of CYP3A4 or P-gp within 14 days or 5 half-lives of the drug (whichever is longer) prior to dosing in this study.<br>20. Known sensitive substrates of CYP2D6 and CYP3A4 within 14 days or 5 half-lives of the drug (whichever is longer) prior to dosing in this study, with a narrow therapeutic window for such substrates, unless enrollment is agreed upon by the investigator and sponsor.<br>21. Received known proton pump inhibitors and H2 receptor antagonists within 7 days prior to dosing in this study.<br>22. Patients are expected to receive other anti-tumor therapy during the study treatment, such as resection of metastatic lesions.<br>23. Long-term treatment with daily high-dose aspirin (> 325 mg/day).<br>24. Pregnant or lactating women.<br>25. Known hypersensitivity to the study treatment or any component of its formulation.<br>26. With uncontrolled third space effusion requiring repeated drainage, such as pleural effusion, ascites, pericardial effusion, etc.<br>27. Other conditions that, in the opinion of the investigator, are not suitable for participation in this study.
**Study Drug Strength**	IBI351<br>- Strength: 150mg/tablet<br>- Method of administration: Oral use

**/Method of administration**	[redacted]<br>- [redacted]
**Statistical Methods**	**Sample Size Estimation**<br>**Phase Ib**<br>Phase Ib is divided into a combination dose escalation phase and a dose expansion phase, and no formal statistical hypothesis testing will be performed. A "3 +3" escalation design will be used in the dose escalation phase, with a total of two dose levels, and it is expected that 9 ~ 12 subjects will be enrolled.<br>In the dose expansion phase, it is planned to expand the recommended dose group to 10 ~ 30 subjects in each cohort, with a total of 30 ~ 90 subjects in three cohorts to observe the safety and preliminary efficacy of the study treatment.<br>**Phase III**<br>[redacted]

	[redacted].<br>**Statistical Hypothesis**<br>No statistical assumptions were made for Phase Ib.<br>[redacted]<br>**Statistical Analysis Methods**<br>Descriptive summaries of continuous variables will include, but are not limited to, the number of cases, mean, standard deviation, median, minimum, and

	maximum. Descriptive summaries of categorical variables will include the number and percentage of subjects in each category.<br>Efficacy Analysis<br>Phase Ib<br>ORR: The number and percentage of subjects who achieved objective response were summarized and Clopper-Pearson 95% CI for ORR was calculated.<br>DCR: The analysis method is the same as that of ORR.<br>PFS: Median PFS will be estimated using Kaplan-Meier method, Broolmeyer-Crowley 95% CI will be provided, and survival curve will be plotted; Progression-free survival rates at different time points were estimated.<br>OS: The analysis method is the same as for PFS.<br>DoR: The analysis method is the same as for PFS.<br>TTR: TTR indicators were summarized descriptively, including number of cases, mean, standard deviation, median, minimum and maximum.<br>[redacted].<br>Safety Analysis (Phase Ib/III)

	Subject exposure to study drug was summarized.<br>The incidence and severity of TEAE, TRAE and SAE will be summarized. Laboratory test indicators, vital signs and ECG results will be summarized descriptively. The baseline measurements will be presented in a cross-tabulation with the worst result in the trial.<br><u>PK Analysis (Phase Ib/III)</u><br>Population pharmacokinetic characteristics of IBI351 will be analyzed using nonlinear mixed effects kinetic modeling to estimate population PK parameters and, if necessary, descriptive statistics will be performed for plasma concentrations and PK parameters at each time point in Phase Ib, including but not limited to Cmax, AUC, t1/2, CL/F, V/F.<br>[redacted].<br><u>Biomarker Analysis (Phase Ib/III)</u><br>Biomarkers potentially predictive of response in tumor tissue will be summarized descriptively, and the relationship between biomarkers and response will be explored and analyzed.

Study Visit Flow Chart

Table 1. Phase Ib Study Visit Flow Chart

Phase	Screening period	Treatment Period				End of treatment Visit[17]	Safety Follow-up [18]	Survival follow-up [19]
		Cycle 1 (every 28 days)		Cycle 2 and Subsequent Cycles (every 28 days)				
Days	-28 ~-1	1	15	1	15	Study Treatment Discontinuation	30 days after last dose	Every 12 weeks after safety follow-up
Window (days)	-28 ~-1	NA	± 3	± 3	± 3	+7	± 7	± 7
**General Study Procedures**								
Written informed consent[1]	X							
Inclusion/Exclusion Criteria	X							
Demographics/Past Medical History/Prior Therapy[2]	X							
Vital signs[3]	X	X	X	X	X	X	X	
Weight/Height[4]	X	X	X	X	X	X		
Physical examination	X	X	X	X	X	X	X	
ECOG PS score	X	X	X	X	X	X	X	

Phase	Screening period	Treatment Period				End of treatment Visit[17]	Safety Follow-up [18]	Survival follow-up [19]
		Cycle 1 (every 28 days)		Cycle 2 and Subsequent Cycles (every 28 days)				
Days	-28 ~-1	1	15	1	15	Study Treatment Discontinuation	30 days after last dose	Every 12 weeks after safety follow-up
Window (days)	-28 ~-1	NA	± 3	± 3	± 3	+7	± 7	± 7
12-lead ECG[5]	X	X	X	X	X	X	X	
**Laboratory Tests**								
Hematology/blood chemistry[6]	X	X	X	X	X	X	X	
Urinalysis [7]	X	X	X	X	X	X	X	
Pregnancy Test[8]	X	X				X		
HIV, HBV, HCV[9]	X							
**Safety Assessments**								
Assessment of Adverse Events[10]	X	X				X	X	
Concomitant Medications[11]	X	X				X	X	
**Efficacy Assessments**								
Tumor Imaging Assessment[12]	X	X				X		

Phase	Screening period	Treatment Period				End of treatment Visit[17]	Safety Follow-up [18]	Survival follow-up [19]
		Cycle 1 (every 28 days)		Cycle 2 and Subsequent Cycles (every 28 days)				
Days	-28 ~-1	1	15	1	15	Study Treatment Discontinuation	30 days after last dose	Every 12 weeks after safety follow-up
Window (days)	-28 ~-1	NA	± 3	± 3	± 3	+7	± 7	± 7
**Study drug administration**								
IBI351[13]		X	X	X	X			
Cetuximab [14]		X	X	X	X			
**PK**								
PK[15]			See Table 3 and Table 4 for details					
**Biomarker Exploration**								
Tumor Tissue Specimens [16]	X							

Remarks:

1 Signature of the Informed Consent Form (ICF) should be performed prior to any study procedures. Qualified imaging performed prior to obtaining informed consent may be used for the screening assessments with no need to be repeated.

2 Prior therapies included: therapies for the study disease, including chemotherapy, radiation therapy, and surgery, as well as therapies for prior concomitant diseases within 30 days prior to the first dose.

3 Vital signs include: temperature, pulse, respiratory rate, and blood pressure.

4 Height will be measured only at screening. Body weight will be measured prior to each scheduled dose during the study.If the subject’s weight fluctuates less than 10% from baseline (day of first dose of study treatment), the baseline weight will be used to calculate the dose. Otherwise, the actual dose will be calculated according to the body weight on the scheduled day of administration.

5 12-lead ECG will be performed at screening (3 times, at least 5 minutes apart, the mean value of the 3 times will be used as the baseline value), treatment period, end-of-treatment visit and safety follow-up visit, and when clinically indicated. In Phase Ib combination dose escalation, ECG will be performed at 2 hours (± 30 min) after IBI351 administration on C1D1, before and 2h (± 30 min) after IBI351 administration on C2D1, and within 1 to 4 hours after IBI351 administration is recommended for the rest of the treatment period visits. The timepoints for PK collection of subjects in Phase Ib dose expansion are the same as that of subjects in Phase Ib combination dose escalation. The ECG of other subjects in Phase Ib dose expansion is recommended to be performed within 1 to 4 hours after IBI351 administration.

6 Hematology includes: Red Blood Cell (RBC), Hemoglobin (HGB), White Blood Cell (WBC), Platelet (PLT), white blood cell differential [Lymphocyte (LYM), Absolute Neutrophil Count (ANC), Eosinophil count (EOS), Basophil count (BASO)].

Blood biochemistry includes: liver function [Total Bilirubin (TBIL), Alanine Transaminase (ALT), Aspartate Amino Transferase (AST), Gamma-glutamyl transferase (γ-GT), Alkaline phosphatase (ALP), Albumin (ALB), Total Protein (TP), Lactate Dehydrogenase (LDH)], renal function [Urea, serum creatinine (Cr)], blood electrolytes [Sodium (Na), Potassium (K), Chloride (Cl), Magnesium (Mg), Calcium (Ca), Phosphate (P)].

Hematology and blood chemistry will be performed at screening, within 3 days prior to dosing in Cycle 1 (may not be repeated if the screening examinations meet the time window requirements), within 3 days prior to dosing on Day 15, 3 days prior to dosing on Day 1 and Day 15 of subsequent cycles, at the End of Treatment Visit, at the Safety Follow-up Visit, and as clinically indicated.

7 Urinalysis includes: pH, urine specific gravity (SG), urine glucose (UGLU), urine protein (UPRO), urine red blood cells (URBC), urine white blood cells (UWBC), urine bilirubin (UBIL), urobilinogen (URO) and blood (BLD). Urinalysis will be performed at screening, within 3 days prior to dosing in Cycle 1 (may not be repeated if the screening examination meets the time window), within 3 days prior to dosing on Day 15, 3 days prior to dosing on Day 1 and Day 15 of subsequent cycles, at the End of Treatment Visit, at the Safety Follow-up Visit, and as clinically indicated.

8 A serum pregnancy test will be performed for women of childbearing potential within 7 days prior to the first dose, and a urine pregnancy test may be performed for the following visits. Test results within 7 days of baseline are acceptable and with no need to repeated. Pregnancy tests will be performed every 12 weeks thereafter.

9 Tests for Human Immunodeficiency Virus (HIV) antibody, hepatitis B virus [hepatitis B surface antigen (HBsAg), hepatitis B surface antibody (HBsAb), hepatitis B virus core antibody (HBcAb), hepatitis B e antigen (HBeAg), hepatitis B e antibody (HBeAb)] and hepatitis C virus (HCV) antibody will be performed at screening. For HBsAg and/or HBcAb positive subjects, HBV-DNA should be further determined. For HCV antibody positive subjects, HCV-RNA should be further determined. After the screening period, the investigator may determine whether to re-examine as clinically indicated.

10 Collect all adverse events, including serious adverse events (SAE), whether observed by the investigator or spontaneously reported by the subject, from the time of signing the informed consent to 30 days after the last dose. No later than 30 days after the last dose, the investigator should report serious adverse events that are considered related to the study drug or study procedures. AE and laboratory safety assessments will be assessed according to NCI CTCAE v5.0. Refer to the description in Section 7 of the protocol for the definition, recording, relatedness judgment, severity judgment, reporting time limit and handling of AE and SAE.

11 Concomitant medications were collected during the Screening Period, Treatment Period, End of Treatment Visit, and Safety Follow-up Visit.

12 Tumor assessments will be performed by the investigator according to RECIST v1.1. Tumor imaging examination usually includes contrast-enhanced CT or MRI, and the examination sites include chest, abdomen and pelvic cavity; Enhanced CT or MRI of the head and neck can be performed if necessary. The same imaging technique should be performed on the same subject during the study. Bone scan must be performed in patients with suspected bone metastases at baseline. Baseline assessment will be performed within 28 days prior to the first dose, and the investigator can collect imaging results within 28 days prior to the first dose for assessment. Tumor imaging assessments will be performed every 6 weeks (± 7 days) for 48 weeks after the first dose of study drug and every 12 weeks (± 7 days) thereafter. For subjects with an initial documented response [complete response (CR) or partial response (PR)], radiographic assessment will be performed after 4 weeks (+7 days) for response confirmation and thereafter at the scheduled assessment cycle until radiographic disease progression is documented. For subjects who discontinue treatment for reasons other than radiographic disease progression, radiographic assessments should continue until disease progression.

13 IBI351 tablets will be taken on designated visit days and will be taken orally as scheduled. Cetuximab will be administrated intravenously following intake of IBI351.

14 Cetuximab 500mg/m^2 IV, repeated every 2 weeks. Prophylaxis with antihistamines and corticosteroids is recommended at least 1 hour prior to the first dose and subsequent doses of cetuximab.

15 The time points and time windows for PK sample collection are shown in Table 3 and Table 4.

16 Subjects will provide eligible archival or fresh tumor tissue slides for KRAS testing and exploratory research (optional) during the screening period, as described in the Laboratory Operations Manual.

17 The End of Treatment Visit will be performed at the time of confirmation of discontinuation of study treatment. If the subject has the corresponding laboratory test results within the end of treatment visit window, it is not necessary to repeat the test.

18 Safety Follow-up 30 days (± 7 days) after the last dose; The date of safety follow-up is within 7 days of the date of end of treatment follow-up, and safety follow-up is not required.

19 Survival follow-up will be performed every 12 weeks (± 7 days) after the end of Safety Follow-up or every 12 weeks (± 7 days) after confirmed discontinuation if the subject did not have Safety Follow-up.

Note: Cycle 1 and subsequent Day 15 visits are not required for Cohort C.

Table 2. Phase 3 Study Visit Flow Chart

[redacted]	[redacted]	[redacted]				[redacted]	[redacted]	[redacted]
		[redacted]		[redacted]				
[redacted]	[redacted]	[redacted]	[redacted]	[redacted]	[redacted]	[redacted]	[redacted]	[redacted]
[redacted]	[redacted]	[redacted]	[redacted]	[redacted]	[redacted]	[redacted]	[redacted]	[redacted]
[redacted]								
[redacted]	[redacted]							
[redacted]	[redacted]							
[redacted]	[redacted]							
[redacted]	[redacted]	[redacted]	[redacted]	[redacted]	[redacted]	[redacted]	[redacted]	
[redacted]	[redacted]	[redacted]	[redacted]	[redacted]	[redacted]	[redacted]		
[redacted]	[redacted]	[redacted]	[redacted]	[redacted]	[redacted]	[redacted]	[redacted]	
[redacted]	[redacted]	[redacted]	[redacted]	[redacted]	[redacted]	[redacted]	[redacted]	
[redacted]	[redacted]	[redacted]	[redacted]	[redacted]	[redacted]	[redacted]	[redacted]	
[redacted]								
[redacted]	[redacted]	[redacted]	[redacted]	[redacted]	[redacted]	[redacted]	[redacted]	
[redacted]	[redacted]	[redacted]	[redacted]	[redacted]	[redacted]	[redacted]	[redacted]	

[redacted]	[redacted]	[redacted]				[redacted]	[redacted]	[redacted]
		[redacted]		[redacted]				
[redacted]	[redacted]	[redacted]	[redacted]	[redacted]	[redacted]	[redacted]	[redacted]	[redacted]
[redacted]	[redacted]	[redacted]	[redacted]	[redacted]	[redacted]	[redacted]	[redacted]	[redacted]
[redacted]	[redacted]	[redacted]				[redacted]		
[redacted]	[redacted]							
[redacted]								
[redacted]	[redacted]	[redacted]				[redacted]	[redacted]	
[redacted]	[redacted]	[redacted]				[redacted]	[redacted]	
[redacted]								
[redacted]		[redacted]				[redacted]	[redacted]	
[redacted]								
[redacted]	[redacted]	[redacted]				[redacted]		
[redacted]								
[redacted]		[redacted]						
[redacted]								
[redacted]		[redacted]	[redacted]	[redacted]	[redacted]			
[redacted]		[redacted]	[redacted]	[redacted]	[redacted]			
[redacted]		[redacted]	[redacted]	[redacted]	[redacted]			

[redacted]	[redacted]	[redacted]				[redacted]	[redacted]	[redacted]
		[redacted]		[redacted]				
[redacted]	[redacted]	[redacted]	[redacted]	[redacted]	[redacted]	[redacted]	[redacted]	[redacted]
[redacted]	[redacted]	[redacted]	[redacted]	[redacted]	[redacted]	[redacted]	[redacted]	[redacted]
[redacted]		[redacted]	[redacted]	[redacted]	[redacted]			
[redacted]		[redacted]	[redacted]	[redacted]	[redacted]			
[redacted]		[redacted]	[redacted]	[redacted]	[redacted]			
[redacted]								
[redacted]		[redacted]						
[redacted]								
[redacted]	[redacted]							

[redacted]

[redacted]

[redacted]

[redacted]

Table 3. PK Sampling Schedule-Ib Dose Escalation

[redacted]		[redacted]	[redacted]
[redacted]	[redacted]	[redacted]	[redacted]
[redacted]	[redacted]	[redacted]	[redacted]
[redacted]	[redacted]	[redacted]	[redacted]
[redacted]	[redacted]	[redacted]	[redacted]
[redacted]	[redacted]	[redacted]	[redacted]
[redacted]	[redacted]	[redacted]	[redacted]
[redacted]	[redacted]	[redacted]	[redacted]
[redacted]	[redacted]	[redacted]	[redacted]
[redacted]	[redacted]	[redacted]	[redacted]
[redacted]	[redacted]	[redacted]	[redacted]
[redacted]	[redacted]	[redacted]	[redacted]

[redacted]

[redacted]

Table 4. PK Sampling Schedule-Ib Dose Expansion

Cycle	Day	Scheduled Time Point (Hour) 1	Sampling window 1
1	1	Pre-dose	-1 h
1	1	2 h post-dose	± 5 min
2	1	Pre-dose	-1 h
2	1	2 h post-dose	± 5 min
3	1	Pre-dose	-1h

Remarks:

1. Scheduled timepoints were pre-dose or post-dose timepoints of the first IBI351 dose on the day.
2. In dose expansion phase Ib, PK collection will be performed for a total of 16 subjects in Cohorts A and B, respectively.

Table 5. PK Sampling Schedule-Phase 3

[redacted]	[redacted]	[redacted]	[redacted]
[redacted]	[redacted]	[redacted]	
[redacted]	[redacted]	[redacted]	[redacted]
[redacted]	[redacted]	[redacted]	
[redacted]	[redacted]	[redacted]	[redacted]

[redacted]

[redacted]

[redacted]

Study Design Diagram

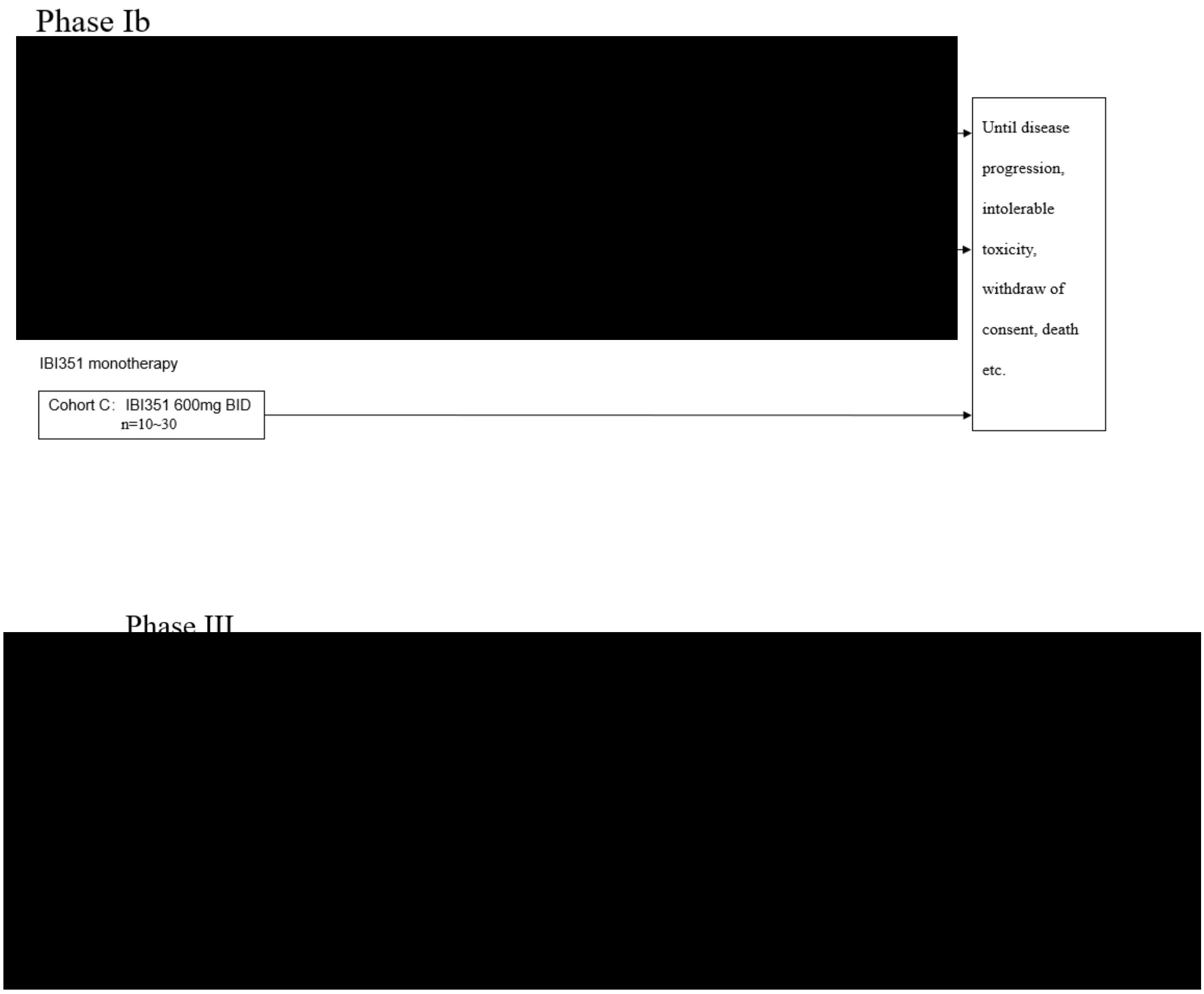

Figure 1. Study Design Diagram

Table of Contents

Sponsor Signature Page 2
Investigator Signature Page 3
Protocol Summary 4
Study Visit Flow Chart 23
Study Design Diagram 37
Table of Contents 38
Table of Tables 41
List of Figures 41
List of Abbreviations and Definitions of Terms 42
1. Introduction 46
1.1. Study Background 46
1.2. Risk/Benefit Assessment 52
2. Study Objectives, Endpoints, and Estimated Objectives 53
2.1 Phase Ib 53
2.2 Phase III 57
3. Study Design 60
3.1. Overall Design 60
3.2. Study Design Rationale 63
3.3. Independent Data Monitoring Committee 66
3.4. Definition of End of Study 66
3.5. Clinical Criteria for Study Discontinuation/Early Termination 66
4. Study Population 67
4.1. Inclusion Criteria 67
4.2. Exclusion Criteria 69
4.3. Study Restrictions and Considerations 72
4.4. Subject Screening 73
4.5. Subject Discontinuation from Treatment/Study 74
4.6. Lost to follow-up 75
5. Study Treatment and Concomitant Therapy 76
5.1. Treatment Regimen 76
5.2. Study drug 80
5.3. Dose Modification 81
5.4. Concomitant Therapy 92
5.5. Drug-related effects 93
5.6. Treatment compliance 94
5.7. Drug management 94
5.8. Records of Study Drug 95
5.9. Complaint Handling 95
6. Study Assessments and Procedures 95

6.1. Safety Assessments 95
6.2. Efficacy evaluation 97
6.3. Pharmacokinetic Specimen Collection and Analysis 98
6.4. Quality of life assessment 98
6.5. Biomarker Specimen Collection and Assessment 98
6.6. Other Processes 99
7. Safety Reporting and Adverse Event Management 99
7.1. Definition of Adverse Events 99
7.2. Definition of Serious Adverse Events 99
7.3. Assessment of Severity of Adverse Events 100
7.4. Causal relationship judgment between adverse event and study drug 101
7.5. Recording of Adverse Events 101
7.6. SAE, Pregnancy, and Hepatic Function Abnormal Event Reporting 104
8. Statistical Considerations 106
8.1. Statistical Analysis Plan 106
8.2. Hypothesis testing 106
8.3. Sample Size Estimation 108
8.4. Statistical Analysis Populations 109
8.5. Statistical Analysis Methods 111
8.6. Control of bias 118
9. Study Quality Assurance and Quality Control 118
9.1. Clinical Monitoring 118
9.2. Data Management 119
9.3. Quality Assurance Audit 120
10. Ethics 121
10.1. Ethics Committee 121
10.2. Ethical conduct in the study 121
10.3. Subject Information and Informed Consent 121
10.4. Data Protection 122
10.5. Protocol Deviations 122
11. Study Management 122
11.1. Data Handling and Record Retention 122
11.2. Access to Raw Data/Documents 123
11.3. Protocol Amendment 123
11.4. Investigator Responsibilities 123
11.5. Publication Policy 123
11.6. Finance and Insurance 124
12. References 124
13. Appendices 127
Appendix 1: Protocol Amendment History 127

Appendix 2: Performance Status Scoring Criteria (ECOG PS) 128
Appendix 3: Response Evaluation Criteria in Solid Tumors version 1.1 (RECIST v1.1) 129
Appendix 4: List of Causality Judgment between Adverse Events and Study Drug 142
Appendix 5: List of Prohibited Medications for Concomitant Therapy 144
Appendix 6: EQ-5D-5L Life Scale 145
Appendix 7: EORTC QLQ-C30 (V3) Life Scale 148

Table of Tables

Table 1. Phase Ib Study Visit Flow Chart....23
Table 2. Phase 3 Study Visit Flow Chart....29
Table 3. PK Sampling Schedule-Ib Dose Escalation....35
Table 4. PK Sampling Schedule-Ib Dose Expansion....35
Table 5. PK Sampling Schedule-Phase 3....35
Table 6. Effective methods of contraception (at least 1 method must be used)....72
Table 7. Treatment Regimens....77
Table 8. Study Drug Dose Modification Grades....81
Table 9. IBI351 Dose Level....82
Table 10. Dose Modification Principles for Hematologic Toxicity....83
Table 11. Dose Modification Principles for Non-Hematologic Toxicities....85
Table 12. Recommended dose modifications for cetuximab-related adverse events....88
Table 13. Dose Modifications of Chemotherapy Agents Based on the Worst Toxicity Associated with Chemotherapy in the Previous Cycle....89
Table 14. Oxaliplatin Sensory Neurotoxicity Grading Criteria and Dose Modifications....90
Table 15. Routine Laboratory Tests....95
Table 16. Criteria for judging abnormal liver function tests....105
Table 17. Analysis Boundaries for PFS and OS at Different Potential Alpha Testing Levels....108

List of Figures

FIGURE 1. STUDY DESIGN DIAGRAM....37
FIGURE 2. ALPHA REASSIGNMENT STRATEGY FOR PFS AND OS ENDPOINTS....107

List of Abbreviations and Definitions of Terms

Abbreviations	English full name
AE	Adverse Event
ALB	Albumin
ALP	Alkaline Phosphatase
ALT	Alanine Transaminase
ANC	Absolute Neutrophil Count
AR	Accumulation Ratio
AST	Aspartate Amino Transferase
AUC	Area Under Curve
BASO	Basophil
Ca	Calcium
CI	Confidence Interval
CL	Clearance
Cl	Chlorine
Cmax	Maximum Serum Concentration of Drug
CR	Complete Response
Cr	Creatinine
CRA	Clinical Research Associate
CRO	Contract Research Organization
CSCO	Chinese Society of Clinical Oncology
CSR	Clinical Study Report
CT	Computed Tomography
CTCAE	Common Terminology Criteria for Adverse Events
DBIL	Total Bilirubin
DC	Dendritic Cell
DCR	Disease Control Rate
DDS	Dose Determination Set
DLT	Dose-limiting Toxicity
DoR	Duration of Response
EC	Ethics Committee
ECG	Electrocardiogram
ECOG PS	Eastern Cooperative Oncology Group Performance Status
eCRF	Electronic Case Report Form
EDC	Electronic Data Capture

Abbreviations	English full name
EGFR	Epidermal Growth Gactor Receptor
EORTC	European Organization for Research and
QLQ	Treatment of Cancer Quality of Life Questionnaire
EOS	Eosinophilic Granulocyte
EOT	End of Treatment
ESMO	European Society for Medical Oncology
FAS	Full Analysis Set
FBG	Fasting Blood Glucose
FDA	Food and Drug Administration
mFOLFOX	Modified Folinic Acid/Fluororacil/Oxaliplatin
FU	Fluorouracil
GCP	Good Clinical Practice
G-CSF	Granulocyte-colony Stimulating Factor
HBcAb	Hepatitis B Core Antibody
HBeAb	Hepatitis B E Antibody
HBeAg	Hepatosis B E Antigen
HBsAb	Hepatitis B Surface Antibody
HBsAg	Hepatosis B Surface Antigen
HBV	Hepatitis B Virus
HCV	Hepatitis C Virus
HGB	Hemoglobin
HIV	Human Immunodeficiency Virus
HNSTD	Highest Non-severely Toxic Dose
HR	Heart Rate
IC50	Inhibition Concentration 50%
ICF	Informed Consent Form
ICH	International Conference on Harmonisation
INR	International Normalized Ratio
IRRC	Independent Radiographic Review Committee
ITT	Intentions-to-Treat
IV	Intravenous
K	Potassium
LDH	Lactate Dehydrogenase
LLOQ	Lower Limited of Quantitation
LYM	Lymphocyte
MAD	Maximum Administered Dose

Abbreviations	English full name
MedDRA	Medical Dictionary for Regulatory Activities
Mg	Magnesium
MRI	Magnetic Resonance Imaging
MTD	Maximum Tolerated Dose
Na	Natrium
NCCN	National Comprehensive Cancer Network
NCI	National Cancer Institute
NOAEL	No Observed Adverse Effect Level
ORR	Overall Response Rate
OS	Overall Survival
P	Phosphate
PD	Progressive Disease
PET	Positron Emission Tomography
PFS	Progress Free Survival
PK	Pharmacokinetic
PLT	Platelet
PPS	Per Protocol Set
PR	Partial Response
PT	Preferred Term
Q2W	Every 2 Weeks
Q3W	Every 3 Weeks
RBC	Red Blood Cell
RECIST	Response Evaluation Criteria in Solid Tumors
RP2D	Recommended Phase 2 Dose
SAE	Serious Adverse Event
SAP	Statistical Analysis Plan
SC	Subcutaneous
SD	Stable Disease
SOC	System Organ Class
SS	Safety Set
TBIL	Total Bilirubin
TCR	T-cell Receptor
TEAE	Treatment Emergent Adverse Event
TKI	Tyrosine Kinase Inhibitor
TP	Total Protein
TRAE	Treatment Related Adverse Event

Abbreviations	English full name
TTR	Time to Response
UC	Urothelial Carcinoma
UGLU	Urinary Glucose
ULN	Upper Limited of Normal Value
UPRO	Urine Protein
URBC	Urinary Red Blood Cells
UREA	Urea
UWBC	Urinary White Blood Cells
V	Apparent Volume of Distribution
WBC	White Blood Cell
WHO	World Health Organization
WT	Wild Type
γ-GT	Gama-glutamyl Transpeptidase

1. Introduction

1.1. Study Background

1.1.1. Disease Background

Colorectal carcinoma (CRC) is one of the most common malignant tumors in digestive system, which seriously endangers human health. In the world, the incidence of colorectal cancer ranks the third and the mortality ranks the fourth in malignant tumors [1]. China accounts for 18.6% of all new cases of colorectal cancer in the world [2]. In China, the incidence and mortality of colorectal cancer are increasing year by year. According to the statistics of the National Cancer Center, the number of new cases of colorectal cancer in China in 2015 was 388,000, the incidence rate was 28.2/100000, the standardized incidence rate was 18.02/100000, the number of colorectal cancer deaths was 187,000, the mortality rate was 13.61/100000, and the standardized mortality rate was 8.21/100000 [3]. Colorectal cancer ranks the fifth in the incidence of malignant tumors in Chinese population, with the fifth in males and the fourth in females, and the fifth in the death cause of tumors, with the fifth in both males and females [4].

Despite some progress in the treatment of colorectal cancer in the past few years, the toxicity of chemotherapeutic drugs and the resistance of some patients to traditional chemotherapeutic drugs are still serious problems in the treatment of colorectal cancer. The 5-year survival rate of colorectal cancer in Chinese patients is only 32%, which is much lower than the 64% and 41% in the United States and Europe. About 35% of colorectal cancer patients in China are metastatic patients (mCRC), which can only receive palliative treatment without radical surgery [5] [6].

Treatment of advanced CRC also varies according to the presence or absence of mutations in genes such as RAS, BRAF, and HER2. When both RAS or BRAF wild-type, the treatment used was single-agent chemotherapy or multiagent chemotherapy in combination with cetuximab or bevacizumab.

In current clinical practice, mFOLFOX6 regimen (oxaliplatin + 5-fluorouracil + folinic acid) or FOLFIRI regimen (irinotecan + fluorouracil + folinic acid) with or without anti-vascular drug VEGF (generally select bevacizumab for patients with KRAS mutation) is the second-line standard treatment for patients with colorectal cancer with KRAS mutation. The objective response rate (ORR) is 3-15%, progression-free survival (PFS) is 4.1-5.5 months, and overall survival (OS) is 10.0-11.1 months [7] [8]. For the choice after second-line treatment progression, the approved treatment regimens in China include regorafenib, fruquintinib and TAS-102, but the benefits of these regimens are not satisfactory, with ORR of 1-4%, PFS of 2-3 months and OS of 6-9 months according to the package inserts. In conclusion, patients with KRAS-mutant

mCRC have rapid disease progression and lack of effective targeted therapy, and novel drugs and treatment options are urgently needed to improve the prognosis of patients.

In recent years, there has been a breakthrough in the development of drugs targeting the KRAS G12C mutation. Mutation to cysteine at position 12 of the GDP-bound KRAS protein exposes a pocket of SWITCH II that can be occupied by small molecules and form covalent modifications with this cysteine. This covalent modification hinders GEF-catalyzed GDP-GTP turnover, locking KRAS G12C in the inactive state [9]-[11]. Based on this mechanism of action, several small molecule covalent inhibitors targeting KRAS G12C are currently in clinical trials, including Sotorasib/AMG510, Adagrasib/MRTX849, JNJ-74699157/ARS-3248, GDC-6036, D-1553, and JDQ443, in solid tumors harboring the KRAS G12C mutation. Sotorasib has received accelerated FDA approval for the treatment of patients with locally advanced or metastatic non-small cell lung cancer harboring the KRAS G12C mutation who have received at least one prior systemic therapy. In addition, Sotorasib also showed some efficacy in patients with colorectal cancer harboring the KRAS G12C mutation. CodeBreaK100 is a single-arm, phase II clinical study of 62 patients with colorectal cancer with KRAS G12C mutation who received at least one dose of Sotorasib monotherapy. The results showed that ORR was 9.7% (6/62, 95% CI 3.6-19.9), all of which were partial response (PR), disease control rate (DCR) was 82.3% (95% CI 70.5-90.8), median PFS was 4.0 months, and median OS was 10.6 months. In terms of safety, 6 (10%) patients had Grade 3 TRAE, most commonly diarrhea (3%), 1 (2%) subject had a Grade 4 TEAE (blood creatine phosphokinase increased), no deaths occurred, and 2 (3%) patients had serious TRAE (back pain and acute kidney injury) [12].

In colorectal cancer, on the one hand, KRAS mutations allow persistent aberrant activation of the RAS-RAF-MAPK signaling pathway independent of EGFR upstream signaling, and on the other hand, in vitro studies have demonstrated that the sustained effects of KRAS G12C may reduce single-agent efficacy through EGFR-mediated adaptive drug resistance mechanisms, and therefore, drugs targeting KRAS G12C may have synergistic effects in combination with anti-EGFR monoclonal antibodies such as cetuximab to improve efficacy [13]. At the 2021 ESMO Congress, KRYSTAL-1 was a multi-cohort Phase 1/2 study to evaluate the efficacy and safety of Adagrasib in patients with KRAS G12 C-mutated metastatic solid tumors in which the CRC cohort consisted of Adagrasib 600 mg BID monotherapy and Adagrasib 600 mg BID + cetuximab 400 mg/m^2 followed by 250 mg/m^2 QW or 500 mg/m^2 Q2W cohorts [14]. A total of 46 CRC patients were treated with Adagrasib monotherapy (median follow-up of 8.9 months), of which 45 patients were evaluable for efficacy, with an ORR of 22%, a DCR (disease control rate) of 87%, a median duration of response (DoR) of 4.2 months, and a median PFS of 5.6 months. Grade 3/4 adverse events occurred in 30% of patients and no Grade 5 adverse events occurred. In addition, 32 CRC patients were treated with Adagrasib +

cetuximab (median follow-up of 7 months). Among the 28 patients evaluable for clinical efficacy, the ORR was 43% and the DCR was as high as 100%. Grade 3/4 adverse events occurred in 16% of patients, and no Grade 5 adverse events occurred. The results of the dose-finding phase were published on 16 September 2021 in another CodeBreaK 101 study, which administered sotorasib at 960 mg QD and panitumumab (anti-EGFR monoclonal antibody) at 6 mg/kg IV Q2W in patients with mCRC after multiple lines of therapy [15]. The results showed an ORR of 27% and a DCR of 81% in 26 evaluable patients (including 5 patients who progressed after prior treatment with Sotorasib). In terms of safety, the incidence of grade 3/4 TRAEs was 13%. In addition, a global, open-label, randomized Phase 3 study (KRYSTAL-10) evaluating the efficacy and safety of Adagrasib (600 mg BID) in combination with cetuximab (500 mg/m^2 Q2W) compared with chemotherapy in patients with KRAS G12C-mutated mCRC after multiple lines of therapy is ongoing [16]. In summary, the current study preliminarily shows that the combination of agents targeting KRAS G12C and cetuximab as second-line/third-line treatment for metastatic colorectal cancer with KRAS G12C mutation has a good clinical prospect.

1.1.2. Mechanism of study drug

The murine sarcoma viral oncogene homolog (RAS) gene is a class of proto-oncogenes, which contains isoforms of KRAS, NRAS and HRAS genes. RAS mutations are the most common mutations in tumors, involving a variety of cancer types, such as colorectal cancer, pancreatic cancer, lung cancer, melanoma and some blood tumors. KRAS mutations occur mainly in lung adenocarcinoma (about 25% of patients), pancreatic cancer (about 95% of patients), and colorectal cancer (about 35% of patients). Most KRAS gene mutations are point mutations, and the main mutation sites are amino acid positions 12, 13 and 61, of which the mutation at amino acid position 12 (amino acid G to D/C/V/R/A/S) is the most common [17] [18].

KRAS is a small GTP hydrolase with a molecular weight of approximately 21KD, located on the inner side of the cell membrane, downstream of the epidermal growth factor receptor (EGFR) family [19] [20]. When EGFR and other receptors on the cell membrane form a dimer, phosphorylation of the dimer can promote the formation of Grb2-Shc-SOS complex, which activates the guanine nucleotide exchange factor (GEF) protein SOS to recruit guanosine diphosphate (GDP)-bound non-activated KRAS, which converts to guanosine triphosphate (GTP)-bound activated KRAS, and then activates mitogen-activated protein kinase (MAPK) pathway, PI3K signaling pathway, RALGDS-RAL signaling pathway, etc. These signaling pathways play an important role in promoting cell survival and proliferation. The equilibrium of KRAS and GDP/GTP binding is regulated by GEFs [21] [22], which catalyze the displacement of KRAS-bound GDP to GTP, and GTPase activating proteins (GAPs), which facilitate the hydrolysis of KRAS-bound GTP to GDP. The mutation of KRAS, including the

substitution of glycine to cysteine at position 12 (G12C), will break the normal balance of GDP/GTP binding, reduce the binding of KRAS to GAP, and promote the hyperactivation of KRAS in the GTP-bound state, thus promoting the occurrence and growth of tumors. At the same time, KRAS gene mutation can also affect the tumor microenvironment, inhibit anti-tumor immune response, and play a role in promoting tumor [23].

IBI351 is a small molecule covalent irreversible inhibitor of KRAS G12C that potently inhibits GTP/GDP exchange at KRAS G12C, locking KRAS in a GDP-bound inactive state and blocking downstream signaling. IBI351 has been shown in preclinical studies to effectively inhibit the growth of lung, pancreatic and colorectal cancer tumor models harboring the KRAS G12C mutation, with a favorable preclinical pharmacokinetic and safety profile.

1.1.3. Results of preclinical studies of the study drug

IBI351, a KRAS G12C inhibitor developed by the Sponsor, has undergone comprehensive preclinical studies, which are summarized below. Please refer to the IBI351 Investigator's Brochure for further details.

(1) Summary of Pharmacology Studies

- **Primary Pharmacodynamics**

IBI351, a small molecule covalent irreversible inhibitor of KRAS G12C, potently inhibited SOS1-mediated GTP/GDP turnover on KRAS G12C recombinant protein [redacted]

[redacted]

[redacted]

[redacted]

[redacted]

[redacted]

[redacted]

[redacted]

[redacted]

[redacted]

[redacted]

[redacted]

[redacted]

[redacted]

[redacted]

[redacted]

[redacted]

[redacted]

[redacted]

[redacted]

[redacted]

[redacted]

[redacted]

1.2. Risk/Benefit Assessment

1.2.1. Potential Risks

Preclinical toxicology data showed that the target organs of toxicity of IBI351 in repeat-dose experiments in rats were ovaries, uterus and vagina. In addition, adverse reactions have also been reported for approved Sotorasib and products under clinical development, including Adagrasib. The most common treatment-related adverse reactions include liver enzyme elevation and gastrointestinal adverse reactions, showing a well-controlled safety profile.

1.2.2. Potential Benefits

For patients with KRAS mutant colorectal cancer, the second-line standard treatment is mFOLFOX6 regimen (oxaliplatin + 5-fluorouracil + folinic acid) or FOLFIRI regimen (irinotecan + fluorouracil + folinic acid), with or without anti-vascular drug VEGF monoclonal antibody (generally select bevacizumab for patients with KRAS mutant). ORR is about 6%, PFS is 4.1-5.5 months, and OS is 10.0-11.1 months. At the 2021 ESMO Congress, KRYSTAL-1 was a multi-cohort Phase 1/2 study to evaluate the efficacy and safety of Adagrasib in patients with KRAS G12C-mutated metastatic solid tumors in which the CRC cohort consisted of Adagrasib 600 mg BID monotherapy and Adagrasib 600 mg BID + cetuximab 400 mg/m^2 followed by 250 mg/m^2 QW or 500 mg/m^2 Q2W cohorts [14]. A total of 46 CRC patients were treated with Adagrasib monotherapy (median follow-up of 8.9 months), of which 45 patients were evaluable for efficacy, with an ORR of 22%, a DCR of 87%, a median DOR of

4.2 months, and a median PFS of 5.6 months. In addition, 32 CRC patients were treated with Adagrasib + cetuximab (median follow-up of 7 months). Among the 28 patients evaluable for clinical efficacy, the ORR was 43% and the DCR was as high as 100%.

The results of the dose-finding phase were published on 16 September 2021 in another CodeBreaK 101 study, which administered sotorasib at 960 mg QD and panitumumab (anti-EGFR monoclonal antibody) at 6 mg/kg IV Q2W to patients with mCRC after multiple lines of therapy [15]. The results showed an ORR of 27% and a DCR of 81% in 26 evaluable patients (including 5 patients who progressed after prior treatment with Sotorasib). In addition, a global, open-label, randomized Phase 3 study (KRYSTAL-10) evaluating the efficacy and safety of Adagrasib (600 mg BID) in combination with cetuximab (500 mg/m^2 Q2W) compared with chemotherapy in patients with KRAS G12C-mutated mCRC after multiple lines of therapy is ongoing [16].

In summary, the current study preliminarily shows that the combination of agents targeting KRAS G12C and cetuximab has a good clinical prospect for the treatment of metastatic colorectal cancer with KRAS G12C mutation.

1.2.3. Evaluation of Potential Risks and Benefits

Based on the biological characteristics of the KRAS G12C target and the product characteristics of IBI351, as well as the aforementioned available non-clinical data of IBI351 and published clinical data of similar targets, the protocol has designed the corresponding eligibility criteria, lifestyle requirements, safety examination and follow-up procedures. The risks to the subjects will be minimized as far as possible by strict protocol design, selection of study sites, collection of drug safety information and timely updating of drug safety information.

Based on the current clinical data of products with the same mechanism of action, under the close safety monitoring of this study design, it is expected that the benefit may be provided to the subjects with the premise of subject safety, and the overall benefit/risk assessment supports the conduct of this study.

Further details on the potential benefits and risks of IBI351 in combination with cetuximab can be found in the Development Safety Update Report. Unexpected adverse reactions are not known at this time. If the subject experiences any discomfort, or new changes in his/her condition, or any unexpected situation during the study, regardless of whether it is related to the drug, the investigator should make timely judgment and make medical treatment.

2. Study Objectives, Endpoints, and Estimands

2.1 Phase Ib

Study Objectives	Study Endpoints
**Primary Objectives**	**Primary Endpoints**
• [redacted]<br>• To evaluate the efficacy of IBI351 as a single agent or in combination with cetuximab in metastatic colorectal cancer with KRAS G12C mutation in terms of Objective Response Rate (ORR).	• [redacted]<br>• Objective response rate (ORR) during the dose expansion phase as assessed by the investigator according to RECIST v1.1 criteria.
**Secondary Objectives**	**Secondary Endpoints**
• To assess the safety/tolerability of IBI351 as a single agent or in combination with cetuximab for the treatment of metastatic colorectal cancer with KRAS G12C mutation;<br>• To characterize the pharmacokinetics (PK) of IBI351 as a single agent or in combination with cetuximab in metastatic colorectal cancer with KRAS G12C mutation;<br>• To evaluate additional efficacy of IBI351 as a single agent or in combination with cetuximab in metastatic colorectal cancer with KRAS G12C mutation.	• Incidence, relatedness and severity of treatment-emergent adverse events (TEAE), treatment-related adverse events (TRAE) and serious adverse events (SAE) (CTCAE v5.0 criteria). Changes in vital signs, laboratory test results and 12-lead ECG values before, during and after study treatment;<br>• Pharmacokinetics: Pharmacokinetic parameters of subjects in the test group, including but not limited to maximum concentration (Cmax), area under the drug concentration-time curve (AUC), half-life (t1/2), clearance (CL/F) and volume of distribution (V/F);<br>• Progression Free Surival (PFS) assessed by the investigator according to RECIST v1.1 criteria;<br>• Overall Survival (OS);<br>• Disease Control Rate (DCR), Duration of Response (DoR) and Time to Response (TTR) assessed by the investigator according to RECIST v1.1 criteria.
**Exploratory Objectives**	**Exploratory Endpoints**
• To explore potential mechanisms of IBI351 resistance.	• Relationship between gene mutations/fusions in tumor tissues of subjects at baseline and efficacy.

[redacted]

[redacted]

[redacted]

[redacted]

[redacted]

[redacted]

[redacted]

[redacted]	[redacted]	[redacted]
[redacted]	[redacted]	[redacted]
[redacted]	[redacted]	[redacted]
[redacted]	[redacted]	[redacted]

[redacted]

[redacted]

(2) Primary Estimand 2 (ORR assessed by investigator per RECIST v1.1 during the dose expansion phase)

The primary clinical concern was the ORR (as assessed by the investigator per RECIST v1.1) in subjects treated for KRAS G12C mutant metastatic colorectal cancer, regardless of the impact of treatment discontinuation for any reason.

Definition of Estimand:

- Population: Subjects with KRAS G12C mutant metastatic colorectal cancer who failed or were intolerant to or refused one or more prior systemic therapies and met the eligibility criteria. See sections 4.1 and 4.2 for details. Includes subjects who received at least one dose of IBI351 during the dose expansion phase.
- Endpoint: Confirmed Complete Response (CR) or Partial Response (PR) according to RECIST v1.1 criteria prior to initiation of new antineoplastic therapy, first disease progression, or death due to any cause, whichever occurs first.
- Treatment: IBI351 (Maximum Administered Dose (MAD) [if Maximum Tolerated Dose (MTD) or MTD is not reached]) alone or in combination with cetuximab until disease progression, intolerable toxicity, withdrawal of consent, or other reason for discontinuation of study treatment, whichever occurs first.

[redacted]

[redacted]	[redacted]	[redacted]
[redacted]	[redacted]	[redacted]
[redacted]	[redacted]	[redacted]
[redacted]	[redacted]	[redacted]

- Population level summary: the proportion of subjects with CR/PR in the analysis population (ORR) and confidence interval will be calculated.

- Considerations for the selection of this estimand: ORR is a reliable reflection of the antitumor activity of a drug and is a common surrogate endpoint in exploratory studies. A hypothetical strategy for starting a new antineoplastic therapy is to consider that the new antineoplastic therapy will affect the assessment of the therapeutic effect of interest.

2.2 Phase III

[redacted]	[redacted]
[redacted]	[redacted]
[redacted]	[redacted]
[redacted]	[redacted]
[redacted]	[redacted]
[redacted]	[redacted]

[redacted]	[redacted]

[redacted]

[redacted]		
[redacted]	[redacted]	[redacted]

[redacted]		[redacted]
		[redacted]
[redacted]	[redacted]	[redacted]
[redacted]	[redacted]	[redacted]

[redacted]

[redacted]

[redacted]

[redacted]

[redacted]

[redacted]

[redacted]

[redacted]

[redacted]

[redacted]		[redacted]
[redacted]	[redacted]	[redacted]

[redacted]		
[redacted]	[redacted]	[redacted]

[redacted]

3. Study Design

3.1. Overall Design

3.1.1. Phase Ib Study Design

The recommended Phase II dose (RP2D) has been determined to be 600mg BID based on comprehensive analysis of PK, efficacy and safety of IBI351 monotherapy in a previous dose escalation study. This Phase Ib study is to explore the safety, tolerability and preliminary efficacy of IBI351 in combination with cetuximab in metastatic colorectal cancer with KRAS G12C mutation.

[redacted]

Phase Ib consists of the following three cohorts:

[redacted]

Cohort C: Subjects with metastatic colorectal cancer harboring the KRAS G12C mutation who have failed or are intolerant to or refuse systemic therapy.

[redacted].

[redacted]

[redacted]

[redacted]

[redacted].

3.1.1.1 Cohort C will be treated with IBI351 600 mg BID monotherapy without dose escalation and will be expanded to 10-30 subjects for preliminary efficacy evaluation. Subjects will receive IBI351 until disease progression, intolerable toxicity, withdrawal of consent, or other reason for discontinuation of study treatment, whichever occurs first. Dose limiting toxicity

[redacted]

[redacted]

[redacted]

[redacted]

[redacted]

[redacted]

[redacted]

[redacted]

[redacted]

[redacted]

[redacted]

[redacted]

[redacted]

[redacted]

[redacted]

[redacted]

[redacted]

[redacted]

[redacted]

[redacted]

[redacted]

[redacted].

3.1.2. Phase 3 Study Design

[redacted]

[redacted]

[redacted]

[redacted]

3.2. Study Design Rationale

3.2.1. Scientific Rationale for Study Design

Multiple clinical studies have demonstrated that KRAS-mutant mCRC is a distinct histologic subtype with rapid disease progression and poor patient survival. Because of its special structure, KRAS was once considered as an unavailable drug target, and there was a lack of effective targeted drugs for a long time. In addition, KRAS mutations result in persistent aberrant activation of the RAS-RAF-MAPK signaling pathway that is independent of upstream EGFR signaling, and consequently, the traditional mCRC-targeted agent cetuximab has little efficacy in patients with KRAS-mutant mCRC. However, in recent years, there has been a breakthrough in drug development for KRAS G12C mutations, and several small molecule covalent inhibitors targeting KRAS G12C

are currently in clinical trials, including Sotorasib/AMG510, Adagrasib/MRTX849, JNJ-74699157/ARS-3248, GDC-6036, D-1553, and JDQ443, for solid tumors with KRAS G12C mutations. Sotorasib has been approved by FDA for marketing. On the one hand, targeting KRAS combined with anti-EGFR monoclonal antibody may completely block RAS-RAF-MAPK signaling pathway from the perspective of mechanism of combination therapy, on the other hand, targeting KRAS combined with anti-EGFR monoclonal antibody has shown promising prospect in several clinical studies of mCRC from the perspective of clinical data. In summary, on the basis of full consideration of the theory of combination therapy and comprehensive evaluation according to the characteristics of clinical trial data, we carried out this study.

3.2.2. Rationale for Primary Endpoint Selection

Phase Ib: It is important to evaluate the safety of the combination in the early clinical development of a new drug. Therefore, the incidence of DLT was selected as one of the primary study endpoints in this study. In addition, according to the guidance of China Food and Drug Administration "Technical Guidelines for Clinical Trials of Combination Therapy with Antineoplastic Drugs", the exploratory trial phase of combination therapy with antineoplastic drugs aims to explore the dose of combination therapy (including timing of administration), the population with potential benefit, and to explore whether the combination therapy is effective and synergistic, so as to provide a reasonable basis for the design of combination therapy regimen to enter the confirmatory trial. Objective response rate (ORR) is the proportion of subjects whose tumor volume shrinks to a pre-specified value and can be maintained for a minimum time period, and is a direct measure of anti-tumor activity of a drug. In summary, safety and ORR were selected as primary endpoints in the Phase 1b study.

[redacted]

[redacted]

[redacted]

[redacted]

[redacted]

[redacted].

[redacted]

[redacted]

[redacted]

[redacted]

[redacted]

[redacted]

[redacted]

[redacted]

[redacted]

Based on the above integrated analysis, the RP2D was 600mg BID. It is expected that the clinical benefits of the subjects can be realized on the premise of the safety of the subjects.

3.3. Independent Data Monitoring Committee

For the interim analysis of the primary efficacy endpoint of OS and the analysis of the primary endpoint of PFS in the Phase III part, the efficacy and safety results of the interim analysis will be provided by a third-party unblinded statistician to the IDMC for review, and the IDMC will judge the efficacy and make recommendations to the sponsor based on the estimated efficacy boundary of the trial. The responsibilities and associated procedures of the IDMC members will be defined in the IDMC charter. The IDMC charter will be finalized and approved by the IDMC and the sponsor prior to the interim analysis.

3.4. Definition of End of Study

A subject was considered to have completed the study if he/she completed survival follow-up or withdrew from the study.

End of study is defined as the last subject completing survival follow-up.

3.5. Clinical Criteria for Study Discontinuation/Early Termination

The study may be suspended or prematurely terminated if there are sufficient reasonable reasons. The Party suspending or terminating the Study shall provide written notice to the Subjects, Investigators, Funding Institution and Regulatory Authorities and document the reason for the suspension or termination of the Study. If the study is prematurely terminated or suspended, the principal investigator shall promptly inform

the subjects and the Ethics Committee (EC), and provide the reasons for the termination or suspension of the study. If applicable, the investigator will contact the subject and notify the subject of changes in the scheduled timing of the visit.

Situations that may require termination or suspension include, but are not limited to:

- Determine that there is an unexpected, significant or unacceptable risk to the subject;
- The justification for effectiveness should be termination/suspension of the study;
- Inability to meet the requirements for protocol compliance;
- Incomplete and/or insufficient data for evaluation;
- Determine that the primary endpoint has been met;
- Planned change or discontinuation of study drug development.

The study will be continued only if issues related to safety, protocol compliance, and data quality are addressed and the requirements of the EC and/or the National Medicines Administration are met.

If the sponsor decides no longer to supply study drug, sufficient notification will be made to allow appropriate adjustments to the subject's treatment.

4. Study Population

4.1. Inclusion Criteria

Subjects were required to meet all of the following criteria to participate in this study:

1. Voluntary participation in the study and signed informed consent.
2. Male or female ≥ 18 years of age and ≤ 75 years of age.
3. Have histologically or cytologically pathologically confirmed unresectable metastatic colorectal cancer.
4. Subjects in Phase Ib:

[redacted]

Cohort C: Subjects with metastatic colorectal cancer harboring KRAS G12C

mutation who have failed systemic therapy or are intolerant to systemic therapy or who refuse systemic therapy;

[redacted]

5. [redacted]

6. [redacted]

7. Have at least one measurable lesion (per RECIST v1.1 criteria).

8. Have adequate organ function including:

- Adequate hematopoiesis as defined by absolute neutrophil count (ANC) ≥ 1.5 × 10^9/L, platelet count ≥ 100 × 10^9/L, and hemoglobin ≥ 9 g/dL. Blood transfusion or treatment with granulocyte colony-stimulating factor, thrombopoietin, erythropoietin, etc. are not allowed within 14 days before blood routine examination.
- Adequate liver function, i.e., total bilirubin (TBIL) < 1.5 × upper limit of normal (ULN), aspartate aminotransferase (AST) and alanine aminotransferase (ALT) < 2.5 × ULN; If Gilbert's syndrome, total bilirubin < 2 × ULN; In case of liver metastasis, AST and ALT should be < 5.0 × ULN; Direct bilirubin (DBIL) < 3.0 × ULN is allowed if it suggests extrahepatic obstruction. Albumin ≥ 30 g/L.
- Adequate renal function as defined by creatinine (Cr) ≤ 1.5 × ULN, or calculated creatinine clearance (CrCl) ≥ 60 mL/min using the Cockcroft-Gault formula when Cr > 1.5 × ULN.

9. Toxicities caused by previous anti-tumor treatment should be recovered to CTCAE grade ≤ 1 before enrollment (except for alopecia, the values specified in the inclusion criteria have been met, or the toxicity of grade 2 that is clinically

stable and does not affect the safety of study drug treatment as determined by the investigator).

10. Eastern Cooperative Oncology Group (ECOG) performance status (PS) 0-1.
11. Expected survival time ≥ 12 weeks.
12. Female or male subjects of childbearing potential must agree to use an effective method of contraception from signing of informed consent until 6 months after the last dose of study drug. Female subjects of childbearing potential should have a negative blood pregnancy test within 7 days prior to dosing (inclusive).
13. The investigator judges that the subject can communicate well, follow up on schedule, and complete the study in accordance with the protocol.

4.2. Exclusion Criteria

Subjects who meet any of the following criteria will not be enrolled in the study:

1. [redacted].
2. Known central nervous system metastases or known leptomeningeal disease.
3. Any cerebral arterial thromboembolic event, such as cerebrovascular accident or transient ischemic attack, occurred within 6 months prior to treatment.
4. History of deep vein thrombosis or any other serious thromboembolism within 3 months prior to enrollment. (Thrombosis of implantable venous access port or catheter origin, or superficial vein thrombosis would not be considered "serious" thromboembolism).
5. Have significant cardiovascular disease, such as:
   - Patients who have had definite cardiovascular abnormalities within 6 months, such as myocardial infarction, angina pectoris, heart failure, severe arrhythmia, or have undergone angioplasty, vascular stent implantation, coronary artery bridging surgery, etc.
   - Clinically significant QT/QTcF interval prolongation (QTcF > 470ms for females or > 450ms for males).
6. History of radiation pneumonitis, idiopathic pneumonitis, active pneumonitis, pulmonary fibrosis, diffuse interstitial lung disease, or organizing pneumonia (eg, bronchiolitis obliterans).
7. Presence of significant gastrointestinal diseases, such as intractable hiccups, nausea, vomiting, severe gastrointestinal ulcers, liver cirrhosis, active gastrointestinal bleeding, inflammatory bowel disease leading to prolonged diarrhea (such as colitis or Crohn's disease, etc.) or other diseases that affect the

swallowing of tablets or significantly affect the absorption of oral drugs.

8. Esophageal or gastric varices requiring immediate intervention (e.g., banding or sclerotherapy) or evidence of portal hypertension that is considered to be at high risk of bleeding in the opinion of the investigator.
9. Subjects with a risk of intestinal obstruction (excluding bowel obstruction that has been surgically cured or completely resolved) or intestinal perforation (including but not limited to history of acute diverticulitis, abdominal abscess, abdominal cancer) within 28 days prior to the first dose of this study.
10. Concomitant with other poorly controlled systemic diseases, such as uncontrolled hypertension (systolic blood pressure ⩾ 150 mmHg or diastolic blood pressure ⩾ 100 mmHg) despite standard treatment, diabetes, etc.
11. Have significant acute or chronic infections, including:
    - Active infection requiring systemic treatment.
    - Patients with positive hepatitis B surface antigen (HBsAg) or hepatitis B core antibody (HBcAb) should be tested for hepatitis B virus (HBV) DNA. If the HBV DNA copy number is ⩽ 2.5×10^3 copies/ml or ⩽ 500 IU/ml or below the lower limit of detection, they can be enrolled. Acute or chronic active Hepatitis C Virus (HCV), i.e., HCV antibody positive and HCV RNA level above the lower limit of detection. Human immunodeficiency virus antibody (HIV-Ab) positive.
    - Active pulmonary tuberculosis.
12. Other malignancies within 2 years prior to study entry, with the exception of adequately treated carcinoma in situ of the cervix, focal squamous cell carcinoma of the skin, basal cell carcinoma, prostate cancer not requiring treatment, ductal carcinoma in situ of the breast, and superficial non-muscle invasive urothelial carcinoma.
13. Prior treatment with a KRAS G12C inhibitor, cetuximab, or other anti-EGFR antibody or small molecule EGFR TKI (e.g., erlotinib, etc.). Note: Cohort C allows enrollment of subjects who have previously received cetuximab.
14. [redacted]
15. Known contraindication to study treatment:

[redacted]

[redacted]

[redacted]

16. Has had a surgical procedure (excluding punch biopsy) within 28 days prior to enrollment in this study that may affect the administration of study drugs or study assessments.
17. Received chemotherapy, targeted therapy, endocrine therapy, immunotherapy, other investigational drug or investigational device therapy within 28 days or 5 half-lives (whichever is shorter) prior to dosing in this study, with the exception of maintenance endocrine therapy.
18. Therapeutic or palliative radiation therapy within 14 days prior to dosing in this study.
19. Has received potent inhibitors or inducers of CYP3A4 or P-gp within 14 days or 5 half-lives of the drug (whichever is longer) prior to dosing in this study.
20. Known sensitive substrates of CYP2D6 and CYP3A4 within 14 days or 5 half-lives of the drug (whichever is longer) prior to dosing in this study, with a narrow therapeutic window for such substrates, unless enrollment is agreed upon by the investigator and sponsor.
21. Received known proton pump inhibitors and H2 receptor antagonists within 7 days prior to dosing in this study.
22. Patients are expected to receive other anti-tumor therapy during the study treatment, such as resection of metastatic lesions.
23. Long-term treatment with daily high-dose aspirin (> 325 mg/day).
24. Pregnant or lactating women.
25. Known hypersensitivity to the study treatment or any component of its formulation.
26. With uncontrolled third space effusion requiring repeated drainage, such as pleural effusion, ascites, pericardial effusion, etc.
27. Other conditions that, in the opinion of the investigator, are not suitable for participation in this study.

4.3. Study Restrictions and Considerations

4.3.1. Meal and dietary restrictions

Grapefruit and beverages (eg, red wine, citrus, grapes, or grape juice) were prohibited from at least 7 days prior to the first dose until the end of study treatment.

4.3.2. Other restrictions

4.3.2.1. Childbearing age

Female subjects of childbearing potential who are sexually active with a non-sterilized male partner, and non-sterile male subjects who are sexually active with a female partner of childbearing potential must use at least 1 of the acceptable effective methods of contraception listed in Table 6 from Screening until 6 months after the last dose of study drug and should discuss their discontinuation with a responsible physician after that time point. Periodic abstinence, rhythm methods, and extracorporeal sperm withdrawal methods are not acceptable methods of contraception. Females of childbearing potential are defined as those who have had menarche, have not undergone sterilization (i.e., bilateral tubal ligation, bilateral salpingectomy, or total hysterectomy), and have not yet reached menopause.

Table 6. Effective methods of contraception (at least 1 method must be used)

Barrier method	IUD method	Hormonal method
Male condom with spermicide	With copper T-ring	Implants
Diaphragm plus spermicide	Progesterone-containing T-ring a	Hormonal contraceptive injection or injection
Diaphragm plus spermicide	Levonorgestrel-releasing IUD system (e.g., Mirena ®) a	Combined contraceptive pill<br>Low-dose oral contraceptive pill<br>Contraceptive patch

a. This is also considered a hormonal approach

Women were considered postmenopausal after 12 months of menopause without an alternative medical cause. The requirements according to age are as follows:

- Women > 50 years of age are considered postmenopausal if they have been amenorrheic for 12 months or more after cessation of exogenous hormone therapy and their luteinizing hormone and follicle-stimulating hormone levels are within the accepted postmenopausal range.
- Women ≤ 50 years of age were considered postmenopausal if they had been amenorrheic for 12 months or more after cessation of all exogenous

hormonal therapy, had had radiation-induced oophorectomy with the last menses occurring > 1 year earlier, had had chemotherapy-induced amenorrheic with the last menses > 1 year apart, or had undergone surgical sterilization (bilateral oophorectomy or hysterectomy).

4.3.2.2. Pregnancy

No studies have been conducted to determine whether IBI351 crosses the placental barrier and is not recommended during pregnancy. Pregnant women cannot be enrolled in this study.

4.3.2.3. Lactation

It is not known whether IBI351 is excreted in breast milk. Given that many drugs are present in human milk, breastfeeding lactating women cannot be enrolled in this study.

4.4. Subject Screening

4.4.1. Enrollment Procedure

The investigator will enroll subjects as follows:

1. An Informed Consent Form (ICF) signed by the subject or guardian was obtained before any study-related procedures were performed.
2. Subject eligibility was formally determined by the Principal Investigator or appropriately trained designee after reviewing the inclusion/exclusion criteria.

Subjects who do not meet the study-related criteria (screen failures) may be rescreened. If re-screening of a subject is considered, the investigator must contact the sponsor's responsible medical officer. Rescreening must be approved by the sponsor's medical director, and each subject may be rescreened once. At the time of rescreening, the subject must re-sign the informed consent form and will be re-assigned an identification number. Assessments performed during the initial screening period were acceptable without repeat testing if they were within the study-specified timeframes and met the eligibility criteria.

4.4.2. Handling Procedures for Incorrectly Enrolled Subjects

The inclusion criteria must be strictly followed. If a subject who does not meet the eligibility criteria is found to be enrolled, the sponsor's responsible medical officer and the investigator should discuss whether to continue the subject in the study, with or without the study drug. A subject may continue in the study and receive study drug if the investigator deems it medically appropriate for the subject to continue participation in the study and the sponsor's responsible medical officer agrees with the investigator's decision. A subject was not allowed to continue in the study (with or without study drug treatment) if the investigator considered it medically appropriate for the subject to

continue participation in the study, but the sponsor's medical director did not agree with the investigator's decision. The Investigator will allow subjects who are accidentally enrolled in the study to continue participation in the study only after receiving written approval from the Sponsor.

4.5. Subject Discontinuation from Treatment/Study

4.5.1. Subject discontinued treatment

Discontinuation of study treatment does not represent withdrawal from the study. "Because data on certain clinical events after discontinuation of study treatment may be important for the study, this information must be collected until the subject & '92; s last scheduled visit, even if the subject has discontinued treatment."

Possible reasons for discontinuation of study treatment include:

1) If the subject has disease progression requiring discontinuation of treatment, if the investigator determines that the subject is still benefiting from continued treatment, the study treatment can be continued after discussion with the sponsor;
2) Subjects with intolerable toxicity;
3) The subject starts other anti-tumor therapy;
4) The subject seriously fails to comply with the requirements of the study protocol;
5) The subject is pregnant;
6) The subject is lost to follow-up;
7) Death of the subject;
8) The subject requests to discontinue the treatment, but receives subsequent follow-up;
9) In the opinion of the investigator, it is in the best interest of the subject to discontinue the study treatment;
10) Early termination or completion of the study by the sponsor.

When a subject discontinues study treatment, all visits scheduled for the End of Treatment Visit should be performed at the time of discontinuation, and any adverse events occurring at the time of discontinuation should be followed according to safety requirements.

The investigator may provide advice or alternative treatment methods to the subject according to his/her actual situation.

4.5.2. Subject Withdrawal from the Study

A subject may withdraw consent at any time for any reason or be withdrawn from the study at the discretion of the investigator. In addition, the investigator or sponsor may withdraw a subject from the study if enrollment into the study is inappropriate, if the protocol is violated, or for administrative and/or other safety reasons.

Subjects were withdrawn from the study if any of the following occurred:

- Withdrawal of consent by the subject or legal representative (e.g., parent or legal guardian);
- The investigator decides to withdraw the subject from the study;
- Death from any cause;
- The subject is lost to follow-up;
- That, in the opinion of the investigator and/or sponsor, continued administration of the study drug would place the subject at undue risk, based on the subject's disease or personal circumstances;
- Early termination or completion of the study by the sponsor.

The reason for withdrawal of a subject from the study should be recorded in the electronic case report form (eCRF).

[redacted]

[redacted]

When a subject withdraws from the study, all visits scheduled for the End of Treatment Visit should be performed at the time of withdrawal, and any adverse events occurring at the time of withdrawal should be followed up according to safety requirements.

4.6. Lost to follow-up

A subject will be considered lost to follow-up if he/she does not return to the study site for 2 consecutive scheduled visits and cannot be contacted by the study site staff.

If a subject does not return to the study site for a specified study visit, the following actions must be taken:

- The site attempted to contact the subject, reschedule missed visits, explain to the subject the importance of adhering to the visit schedule, and confirm if the subject is willing and/or should continue in the study.

- Prior to the subject being deemed lost to follow-up, the investigator or designee will make every effort to recontact the subject (three phone calls if possible, a certified letter to the subject's most recent mailing address if necessary, or a valid local contact information). These attempts to contact the subject should be documented in the subject's medical record or study file.

If a subject still cannot be contacted, he/she will be considered lost to follow-up and withdrawn from the study.

5. Study Treatment and Concomitant Therapy

5.1. Treatment Regimen

[redacted]

Table 7. Treatment Regimens

Staging	Group	Therapeutic drug	Dose/Amount	Usage method	Course/Treatment Cycle	Remarks
[redacted]	[redacted]	[redacted]	[redacted]	[redacted]	[redacted]	[redacted].
		[redacted]		[redacted]		
Phase Ib (Dose Expansion Phase)	[redacted]	[redacted]	[redacted]	[redacted]	[redacted]	[redacted].
		[redacted]		[redacted]		
	Cohort C	IBI351	600mg BID	p.o.	IBI351 twice daily in a 28-day treatment cycle	Until disease progression, intolerable toxicity, withdrawal of consent, or other reason for discontinuation of study treatment, whichever occurs first.
[redacted]	[redacted]	[redacted]	[redacted]	[redacted]	[redacted]	
		[redacted]		[redacted]		

Staging	Group	Therapeutic drug	Dose/Amount	Usage method	Course/Treatment Cycle	Remarks
	[redacted]	[redacted]	[redacted]	[redacted]	[redacted]	[redacted]
		[redacted]	[redacted]	[redacted]	[redacted]	
		[redacted]	[redacted]	[redacted]	[redacted]	
		[redacted]		[redacted]	[redacted]	
	[redacted]	[redacted]		[redacted]	[redacted]	
		[redacted]	[redacted]	[redacted]	[redacted]	

Staging	Group	Therapeutic drug	Dose/Amount	Usage method	Course/Treatment Cycle	Remarks
		[redacted]	[redacted]	[redacted]	[redacted]	
		[redacted]	[redacted]	[redacted]	[redacted]	

5.2. Study drug

5.2.1. Description of Study Drug

**Study drug**	**IBI351 (GFH925) Tablets**
Active ingredient	GFH925
Excipients	Microcrystalline cellulose, lactose, croscarmellose sodium, magnesium stearate
Type	Chemical drugs
Dosage form	Tablet
Specification	[redacted]
Route of Administration	Oral
Storage Requirements	Preserved in tightly closed containers at room temperature
Shelf Life	Provisional 24 months
Packaging and labeling	Study drug will be provided to each subject in high-density polyethylene bottles. Each HDPE bottle will be labeled according to local regulatory requirements

5.2.2. Study drug use

IBI351 will be administered orally with approximately 240mL of warm water. Subjects were instructed to swallow the tablet whole and not to chew or break it. Fasting will be performed from 2h before administration to 1h after administration, and the time of administration will be close to each day as far as possible. If PK blood sampling is required in the morning of the same day, the drug should be taken after the blood sampling. When taking twice daily, try to schedule around 8:00 a.m. and 20:00 p.m.

If a dose is missed, the missed dose may be taken within 4 hours after the scheduled time point, and the actual time of administration shall be recorded, and the subsequent time of administration shall not be changed. If it has been more than 4 hours, do not take the missed dose, and the next dose will be scheduled. If vomiting occurs after dosing, it is not necessary to make up the dose. All actual dosing times should be recorded in the Medication Log, or incidents such as "missed doses" should be recorded.

5.2.3. Use of other study drugs

[redacted]

[redacted]

[redacted]

5.3. Dose Modification

5.3.1. Dose Modification Scheme

5.3. 1.1 General Principles

Before each administration of the study drug on Day 1, the subjects' hematological, liver and kidney functions must meet the administration requirements, and all toxicities judged by the investigator to be related to the study drug must have resolved to Common Terminology Criteria for Adverse Events (CTCAE) V5.0 grade 0 ~ 1 level or baseline specified level (except for alopecia, fatigue, conditions judged by the investigator not to affect the safety of the subject's medication or special provisions for medication adjustment in the protocol), before the corresponding study drug can be started. The different dose levels of study drug are presented in Table 8.

In clinical practice, if the investigator considers that the subject can tolerate the study drug, the investigator can administer the study drug after communicating with the sponsor via email, and closely follow-up and timely deal with the possible changes in the following conditions.

The following dose modification scheme is the implementation practice recommended by the sponsor, and the investigator can make corresponding adjustments based on clinical guidelines, combined with clinical practice, taking subject safety as the first consideration, and the reason for the actual modification should be recorded.

All medication modifications should be documented, including the reasons and methods used.

[redacted]

[redacted]

	[redacted]	[redacted]	[redacted]
[redacted]	[redacted]	[redacted]	[redacted]
[redacted]	[redacted]	[redacted]	[redacted]
[redacted]	[redacted]	[redacted]	[redacted]
[redacted]	[redacted]	[redacted]	[redacted]
[redacted]		[redacted]	[redacted]
[redacted]	[redacted]		

5.3. 1.2 Dose Modifications for IBI351

Subjects should be closely monitored for study drug toxicity during the study and best supportive care is recommended for management of toxicity according to site practice. A maximum of 2 dose reductions were allowed for the same subject [redacted]

[redacted]

[redacted]	[redacted]	[redacted]
[redacted]	[redacted]	[redacted]
[redacted]	[redacted]	[redacted]

Refer to Table 10 and Table 11 for detailed principles of dose modification.

If a subject experiences an AE related to study treatment and meets the definition of DLT in Section 3.1. 1.1 of the protocol or any of the discontinuation criteria in Table 10 and Table 11 during the study, IBI351 treatment should be discontinued and followed until resolution or return to baseline. If the subject has experienced tumor response to treatment, or the investigator judges that continued treatment is beneficial, the study treatment may be resumed at the same dose or at a lower dose after the toxicity has recovered to the following level after full communication with the sponsor, while continuing to closely monitor for relevant toxicities. If treatment is restarted at the same dose level, patients should be closely monitored after reinitiation. Dose interruption of IBI351 will be required if the severity of an AE related to study treatment does not meet the criteria for DLT definition, but meets any of the following criteria:

- Thrombocytopenia ≥ Grade 3
- Leukopenia ≥ Grade 3
- Neutropenia ≥ Grade 3
- Anemia ≥ Grade 3
- Non-hematologic toxicity ≥ Grade 3

In case of such hematological toxicity, blood routine should be repeated within 3 days, and the recovery should be closely monitored thereafter. Resumption of treatment may be considered if recovery to the following criteria: non-hematological toxicity recovered to ≤ Grade 1 or baseline level; Hematologic toxicity recovered to ANC ≥ 1. 0 × 109/L, PLT ≥ 75 × 109/L, Hb ≥ 8 g/dL.

Permanent treatment discontinuation should be considered if the subject's adverse events do not recover to drug-tolerable levels within 4 weeks, or if the same toxicity that requires treatment interruption recurs after resumption of treatment.

Permanent discontinuation of treatment should be considered in the event of an adverse event related to IBI351 treatment according to the following criteria, unless alternative treatment is agreed upon by the investigator and the sponsor after discussion:

- ALT or AST > 8 × ULN;
- ALT or AST > 5 × ULN for more than 2 weeks;
- ALT or AST > 3 × ULN with (TBL > 2 × ULN or INR > 1.5);
- ALT or AST > 3 × ULN with signs and symptoms related to hepatitis such as fatigue, nausea, vomiting, right upper quadrant pain or tenderness, fever, rash, and/or eosinophilia (> 5%).

Refer to Table 10 and Table 11 for detailed dose adjustment principles. If the investigator considers that the subject may have clinical benefit and the safety is manageable, the subject's dose escalation will be allowed after further discussion and agreement with the sponsor. Other special circumstances may be further discussed and agreed with the sponsor.

All dose modifications must be recorded in the source data and entered into the eCRF as required.

[redacted]

[redacted]	[redacted]	[redacted]
[redacted]	[redacted]	
	[redacted]	[redacted]
	[redacted]	[redacted]
	[redacted]	[redacted]

[redacted]

[redacted]	[redacted]	[redacted]
	[redacted]	[redacted]
[redacted]	[redacted]	
	[redacted]	[redacted]
	[redacted]	[redacted]
[redacted]	[redacted]	
	[redacted]	[redacted]
	[redacted]	[redacted]
[redacted]	[redacted]	
	[redacted]	[redacted]
	[redacted]	[redacted]

[redacted]

[redacted]	[redacted]	[redacted]
[redacted]		
	[redacted]	[redacted]
	[redacted]	[redacted]
[redacted]	[redacted]	
	[redacted]	[redacted]
	[redacted]	[redacted]
[redacted]	[redacted]	
	[redacted]	[redacted]
	[redacted]	[redacted]
[redacted]	[redacted]	[redacted]
	[redacted]	[redacted]
[redacted]	[redacted]	
	[redacted]	[redacted]

[redacted]

[redacted]	[redacted]	[redacted]
		[redacted]
	[redacted]	[redacted]
[redacted]	[redacted]	
	[redacted]	[redacted]
[redacted]	[redacted]	[redacted]
	[redacted]	[redacted]
	[redacted]	[redacted]
	[redacted]	[redacted]
[redacted]	[redacted]	
	[redacted]	[redacted]
	[redacted]	[redacted]

After disease progression, treatment with IBI351 was allowed to continue with the subject's full informed and consenting consent if the investigator judged that the subject would continue to benefit until treatment discontinuation criteria were met (refer to Section 4.5. 1 of the protocol). If the drug is continued after disease progression, data will continue to be collected at the visits designed for the study.

[redacted]

[redacted].

[redacted]

[redacted]

[redacted]	[redacted]
[redacted]	
[redacted]	[redacted]
[redacted]	[redacted]
[redacted]	
[redacted]	[redacted]
[redacted]	[redacted]
[redacted]	[redacted]
[redacted]	[redacted]
[redacted]	

[redacted]

[redacted]

[redacted]

[redacted]

[redacted]	
[redacted]	
[redacted]	
[redacted]	[redacted]
[redacted]	[redacted]
[redacted]	[redacted]
[redacted]	[redacted]
[redacted]	
[redacted]	[redacted]
[redacted]	[redacted]
[redacted]	[redacted]
[redacted]	[redacted]
[redacted]	[redacted]
[redacted] [redacted]	[redacted]
[redacted]	[redacted]
[redacted]	
[redacted]	

[redacted]

[redacted]		
[redacted]		
	[redacted]	[redacted]
	[redacted]	[redacted]
	[redacted]	[redacted]
	[redacted]	[redacted]
[redacted]		
	[redacted]	[redacted]
	[redacted]	[redacted]
	[redacted]	[redacted]
	[redacted]	[redacted]
[redacted]		
	[redacted]	[redacted]
	[redacted]	[redacted]
	[redacted]	[redacted]
	[redacted]	[redacted]
	[redacted]	
	[redacted]	
	[redacted]	
	[redacted]	[redacted]
	[redacted]	[redacted]
	[redacted]	[redacted]
	[redacted]	[redacted]
	[redacted]	
	[redacted]	[redacted]
	[redacted]	
	[redacted]	[redacted]
[redacted]	[redacted]	[redacted]
[redacted]		[redacted]

[redacted]

[redacted]

[redacted]

[redacted]

		[redacted]		[redacted]
[redacted]		[redacted]	[redacted]	[redacted]
				[redacted]
	[redacted]	[redacted]	[redacted]	[redacted]
[redacted]	[redacted]	[redacted]	[redacted]	[redacted]
[redacted]		[redacted]	[redacted]	[redacted]
	[redacted]	[redacted]	[redacted]	[redacted]
[redacted]	[redacted]	[redacted]	[redacted]	[redacted]
	[redacted]	[redacted]	[redacted]	[redacted]

[redacted]

[redacted]

[redacted].

[redacted]

[redacted]

[redacted]

[redacted]

5.4. Concomitant Therapy

5.4. 1 Permitted Concomitant Therapies

During the study, the investigator should follow the following principles and use concomitant drugs with caution to ensure the safety of subjects to the maximum extent.

Palliative care and best supportive care for disease symptoms are allowed during the study, [redacted] Palliative and supportive care for disease-related symptoms will depend on the investigator's judgment and relevant guidelines (e.g., American Society of Clinical Oncology Guildlines). For example, palliative local radiotherapy for painful bone lesions for symptom relief is allowed. Prior to radiotherapy, the investigator should determine and record whether the use of radiotherapy is associated with disease progression.

If the subject has other diseases during the study, the investigator should determine whether drug treatment is needed and avoid the drugs that have great influence on the judgment of the study results as far as possible, so as not to affect the judgment of the safety and tolerability of the subjects. In case of serious adverse reaction or serious adverse event, or deterioration of original condition or concurrent other serious diseases, the investigator should timely give concomitant treatment and active treatment. If the criteria for subject withdrawal from the study are met, the withdrawal of the subject should be properly arranged.

- If the investigator is unable to determine whether concomitant treatment will affect the safety of the subject, or whether it will affect the judgment of the

safety and tolerability of the subject after medication, or whether it will affect the enrollment eligibility of the subject and the evaluability of the data after medication, the investigator should discuss and reach an agreement with the sponsor before using concomitant treatment.

Subjects should be cautious about concomitant use of known proton pump inhibitors and H2 receptor impedance agents during the study, including but not limited to omeprazole, lansoprazole, pantoprazole, rabeprazole, esomeprazole, ilaprazole, cimetidine, famotidine, nizatidine, roxatidine. If gastric acid reducing agents must be used, IBI351 may be taken 2 hours before or after treatment with known antacids such as aluminum hydroxide, magnesium hydroxide, calcium carbonate, and simethicone.

5.4.2 Prohibited Concomitant Therapy

Subjects should not receive the following treatments during the study:

1) Any other anti-tumor therapy (chemotherapy, immunotherapy, biologic, extensive radiotherapy, hormonal therapy, targeted therapy, surgery, Chinese patent medicines with approved anti-tumor indications) other than the treatment in this study, including investigational or approved therapies. Subjects taking antagonistic gonadotropin-releasing hormone (GnRH) for prostate cancer, oral contraceptives, or hormone replacement therapy may continue medication.
2) Granulocyte colony-stimulating factor drugs were used as prophylaxis. Such drugs can only be used for the treatment of adverse reactions at the discretion of the investigator.
3) Any other drugs under clinical investigation other than the treatment in this study.
4) Known strong inhibitors and inducers of CYP3A and P-gp are prohibited from 2 weeks or 5 half-lives (whichever is longer) prior to the first dose until 2 weeks after the last dose (see Appendix 5).
5) Known sensitive substrates of CYP2D6 and CYP3A are prohibited within 2 weeks or 5 half-lives of the drug (whichever is longer) prior to the first dose of study drug until 2 weeks after the last dose of study drug (see Appendix 5).

5.5. Drug-related effects

IBI351

There are no data on drug interactions with IBI351.

[redacted]

[redacted]

[redacted]

5.6. Treatment compliance

Study treatment was administered at the study site, and treatment compliance was monitored using drug receipt and dispatch records, subject medical records, and eCRFs.

5.7. Drug management

5.7.1. Study Drug Receipt and Accountability

The sponsor will provide the study drug according to the anticipated enrollment plan of the study site. The study drug will be shipped to the study site via a third party logistics company qualified for shipment. Authorized personnel at the site will sign the dispatch form to acknowledge receipt of the drug.

The study drug should only be used in this study and should only be administered by a person authorized by the investigator. In order to fully control the dispensing and use of the study drug, the quantity should be registered at each visit.

5.7.2. Storage and Management of Study Drug

The investigator or authorized other study site personnel (eg, pharmacist) will ensure that all study drug is stored in a secure, controlled-access area under the storage conditions described in Section 5.2 of the protocol and in accordance with applicable regulatory requirements. After receiving the study drug, the subject will follow the instructions of the investigator or authorized personnel to properly store the study drug. If non-conforming storage conditions are identified, the investigator should contact the sponsor for instructions.

All investigational drugs provided by the Sponsor will be used only for this investigational study and not for purposes other than those specified in this protocol. The investigator must undertake not to supply the study drug to any person unrelated to the study.

5.7.3. Return and Destruction of Study Drug

"In this study, used study drugs and packaging will be returned, and containers of chemotherapy drugs may be destroyed locally according to applicable guidelines and procedures established by the study site and local institutions." If some study sites have difficulty in retrieving study drug and packaging, local destruction of chemotherapy drug containers will be allowed with the agreement of the sponsor.

All unused study drugs should be returned to the sponsor for destruction after completion/termination of the study or expiration of the expiration date. The clinical research associate designated by the sponsor will be responsible for arranging the

recovery of the study drug.

5.8. Records of Study Drug

The designated personnel of the study site should timely record the receipt, distribution, use, inventory, destruction, recovery and damage of the study drug according to the requirements of relevant regulations and guidelines.

5.9. Complaint Handling

In order to ensure the safety and monitoring quality of study participants and to assist in process and drug improvement, the sponsor will collect product complaints related to the study drug used in the clinical trial.

Complaints related to concomitant drugs will be reported directly to the manufacturer according to the product description.

The Investigator or his/her designee is responsible for completing the following product complaint process as specified in this study:

- A study-specific complaint form was used to document the reported product complaints and the associated complete description.
- Fax or email the completed Product Complaint Form to the Sponsor or its designee within 24 hours.

If the investigator is required to return the product for investigation, the investigator should return a copy of the product complaint form with the product.

6. Study Assessments and Procedures

6.1. Safety Assessments

6.1.1. Laboratory Tests

6.1.1.1. Routine laboratory tests

Hematology, urinalysis, blood biochemistry and viral serology will be performed according to the study flow chart. See Table 15 for details.

Table 15. Routine Laboratory Tests

Blood routine	Red blood cell count (RBC), hemoglobin (HGB), white blood cell count (WBC), platelet count (PLT), white blood cell differential [lymphocyte count (LYM), absolute neutrophil count (ANC), eosinophil count (EOS), basophil count (BASO)]
Blood biochemistry	Liver function [serum total bilirubin (TBIL), alanine aminotransferase (ALT), aspartate aminotransferase (AST), gamma-glutamyl transferase (γ -GT), alkaline phosphatase (ALP), albumin (ALB), lactate dehydrogenase (LDH), total

	protein (TP)], renal function [urea (Urea), creatinine (Cr)], blood electrolytes (Na, K, Cl, Mg, Ca, P), and fasting plasma glucose (FBG)
Urinalysis	Urine pH, urine specific gravity, urine glucose, urine protein, urine red blood cells, urine white blood cells, urine bilirubin, urobilinogen, occult blood
Viral serology	HBcAb: hepatitis B core antibody; HBeAb: hepatitis B E antibody; HBeAg: hepatitis B E antigen; HBsAb: hepatitis B surface antibody; HBsAg: hepatitis B surface antigen; HBV: hepatitis B virus; HCV: hepatitis C virus; HIV: human immunodeficiency virus

6.1.1.2. Pregnancy test

For women of childbearing potential (as defined in Section 4.3. 2.1 of the protocol), a serum β-HCG sample pregnancy test will be performed according to the time specified in Table 1 and Table 2 of the Schedule of Visits, whichever is the serum result. If the result is positive, the subject is not eligible/must be discontinued from the study. In addition to the time scheduled in the visit form, a blood pregnancy test should be performed if pregnancy is suspected during the study.

6.1.2. Clinical examination

6.1.2.1. Physical examination

A complete physical examination will include: general condition, respiratory, cardiovascular, abdomen, skin, head and neck (including ears, eyes, nose, and throat), lymph nodes, thyroid, musculoskeletal (including spine and extremities), genital/anal, and neurological assessments.

Refer to Schedule of Visits Table 1 and Table 2 for examination time.

6.1.2.2. Vital Signs

Vital signs will be performed as described in Schedule of Visits Table 1 and Table 2. Vital signs include temperature, pulse, respiratory rate, and blood pressure.

6.1.2.3. 12-lead ECG

Resting 12-lead ECGs will be analyzed locally according to Schedule of Visits Table 1 and Table 2.

A 12-lead ECG will be performed after the subject has rested in a recumbent position for at least 5 minutes. All 12-lead ECGs should be recorded while the subject is resting in a recumbent position. Further ECGs will be performed when clinically indicated, e.g. In the event of a cardiac-related adverse event. The investigator completed the ECG assessment on the day of the examination and recorded the assessment on the ECG. The same method of assessment should be used throughout the study.

The investigator should assess all ECGs according to the clinically significant abnormal/not clinically significant abnormal category. In case of clinically significant abnormal findings, the investigator should record the findings as AE in the eCRF.

6.1.3. Adverse Events and Concomitant Treatments

Adverse events will be assessed for name, severity (graded according to CTCAE version 5.0), start and end times, whether it is a serious adverse event, relationship to study treatment, action taken with study treatment, and outcome.

AE occurring after signing the informed consent form until completion of the safety follow-up visit of the study will be recorded in the original medical records and collected in the eCRF. Other drugs or treatments used by the subject while receiving study treatment were recorded in the original medical records as concomitant treatments and collected in the eCRF.

6.2. Efficacy evaluation

6.2.1. Tumor imaging assessment

In the Phase Ib study, the investigator will evaluate the response according to RECIST v1.1, [redacted]. Tumor imaging examination usually includes contrast-enhanced CT or MRI, and the examination sites include chest, abdomen and pelvic cavity; Enhanced CT or MRI of the head and neck can be performed if necessary. The same imaging technique should be performed on the same subject during the study. Bone scan must be performed for patients suspected of bone metastasis at baseline. Thereafter, routine bone scan is not required. The frequency of bone scan is determined by the investigator according to the actual situation of the subject. Baseline assessments will be performed within 28 days prior to the first dose (Phase Ib) or randomization (Phase III), and the investigator may collect imaging results for assessment within 28 days prior to the first dose (Phase Ib) or randomization (Phase III). Subjects will undergo tumor imaging assessments every 6 weeks (± 7 days) for 48 weeks after the first dose (Phase Ib) or randomization (Phase III), and then every 12 weeks (± 7 days) until disease progression, withdrawal of consent, lost to follow-up, death, or study termination, whichever occurs first. Subjects who discontinue treatment prematurely for reasons other than disease progression should still undergo imaging at the time specified in the protocol. Scheduled tumor imaging should not be postponed due to treatment delays, holidays, or any other reason. If disease progression is suspected based on clinical or laboratory findings before the next scheduled assessment, an unscheduled assessment should be performed. If the dosing visit window overlaps with the tumor assessment window, the tumor assessment will be performed first, and the study treatment will be continued after the assessment is effective. Phase Ib: for

subjects with first documented disease response [complete response (CR) or partial response (PR)], radiographic assessment will be performed for response confirmation after 4 weeks (+7 days) and thereafter at the scheduled assessment cycle until radiographic disease progression is documented.

6.3. Pharmacokinetic Specimen Collection and Analysis

6.3.1. Specimen Collection

Refer to PK sampling schedule 3, Table 4 and Table 5 for PK sampling points.

Refer to the Laboratory Manual provided by the central laboratory designated by the sponsor for details of sampling methods, sample storage, transportation and analysis.

6.3.2. Determination method of plasma drug concentration

The concentration of IBI351 will be determined using a validated liquid chromatography tandem mass spectrometry (LC-MS/MS) method. Concentrations below the Lower Limit of Quantitation (LLOQ) will be reported as 0 ng/mL, and missing samples will be flagged accordingly. Details of the analysis will be documented in the bioanalytical report. Testing was performed by a central laboratory designated by the sponsor. All subjects will be required to have their plasma concentrations measured at the blood collection points specified in the protocol.

[redacted]

[redacted]

6.5. Biomarker Specimen Collection and Assessment

6.5.1. Biomarkers

This study will explore the possible mechanisms of drug resistance at the genetic level. Potential resistance mechanisms were investigated by analyzing baseline tissue samples for mutations/fusions in selected genes.

Refer to the laboratory manual for details of sample preparation and handling.

6.5.2. Storage and Destruction of Biological Samples

Samples will be disposed of or destroyed and consolidated and anonymized. Additional analyses may be performed on anonymized, pooled samples to further evaluate and validate the analytical method. Any results obtained from these analyses may be reported separately from the Clinical Study Report (CSR).

Incurred sample reproducibility analysis, if performed, will be performed

concurrently with the bioanalysis of incurred samples. The results of these assessments will not be reported in the CSR but will be presented separately in a bioanalytical report.

6.6. Other Processes

6.6.1 Unscheduled Visit

An unscheduled visit may be performed according to the requirements of the subject or the investigator. The investigator will perform relevant examinations according to the condition of the subject, including but not limited to: vital signs, targeted physical examination, hematology/blood biochemistry/urinalysis and imaging evaluation. All unscheduled visit test results should be recorded in the eCRF.

7. Safety Reporting and Adverse Event Management

7.1. Definition of Adverse Events

An adverse event (AE) is defined as any untoward medical occurrence in a clinical trial subject starting with the signing of the informed consent form, whether or not causally related to the study drug, which is considered to be an AE, including but not limited to the following:

- Exacerbation of pre-existing (before entering the clinical trial) medical condition/disease (including aggravation of symptoms, signs, laboratory test abnormalities);
- Any newly occurring untoward medical condition (including symptoms, signs, newly diagnosed diseases);
- Abnormal clinically significant laboratory values or results.

7.2. Definition of Serious Adverse Events

A serious adverse event is an adverse event that meets at least one of the following criteria:

- Results in death;
- Is life-threatening ("life-threatening" in the definition is an AE in which the subject was at risk of death at the time of its occurrence and does not include an AE that might have caused death if the event were to worsen);
- Requires inpatient hospitalization or prolongation of existing hospitalization, excluding the following:

✓ Rehabilitation facilities;

✓ Nursing home;

✓ Regular emergency room admissions;

- ✓ Same-day surgery (e.g. Outpatient/same-day/ambulatory surgery);
- ✓ Hospitalization or prolongation of hospitalization not associated with worsening of an AE is not per se an SAE. For example, hospital admission due to pre-existing disease, without occurrence of new adverse events or aggravation of pre-existing disease (e.g., to check for persistent laboratory abnormalities before the trial); Hospitalization for administrative reasons (e.g., routine annual physical examination); Hospitalization specified in the trial protocol during the clinical trial (e.g., operating according to the requirements of the trial protocol); Elective hospitalization (e.g., elective surgery) that is not associated with worsening of the adverse event; Scheduled treatments or surgeries should be recorded throughout the trial protocol and/or in the baseline data of the individual subject; Admitted for blood product use only.

- Results in permanent or significant disability/incapacity (significantly interfering with the ability to perform normal life functions).
- Resulting in a congenital anomaly/birth defect (offspring of a subject using the product).
- Other important medical events: Events that may jeopardize the subject and may require medical or surgical intervention to prevent one of the outcomes listed above, although they do not result in death, are not life-threatening, or require hospitalization, are also considered serious based on appropriate medical judgment.

7.3. Assessment of Severity of Adverse Events

The investigator will assess the severity of AE according to the five-grade criteria specified in NCI CTCAE v5.0.

Adverse event terms not included in the NCI CTCAE V5.0 will be graded according to the following CTCAE grading principles:

- Grade 1 mild; No symptoms or slight signs; Clinical or diagnostic observations only; No medical intervention required;
- Grade 2 moderate; Requires minimal, local or non-invasive treatment; Limitation of age-appropriate activities of daily living (e.g., cooking, shopping, using the telephone and managing money);
- Grade 3 serious or clinically significant but not immediately life-threatening; Hospitalization or prolongation of hospitalization; Disability; Restricted in self-care activities of daily living (e.g., bathing, wearing and undressing, eating, using the toilet, and taking medications), but not bedridden;

- Grade 4 resulting in life-threatening consequences; Need for emergency treatment;
- Deaths related to Grade 5 AE.

7.4. Causal relationship judgment between adverse event and study drug

For adverse events in clinical trials. The medically qualified investigator was required to provide an assessment of the causal relationship between the study drug and the adverse event. The elements in Appendix 4 were used to assess the causal relationship between the study drug and the adverse event.

7.5. Recording of Adverse Events

The investigator should use medical terminology/concepts to record AE or SAE. The use of spoken language and abbreviations should be avoided. All AE (including SAE) should be recorded on the Adverse Event Form of the eCRF.

7.5.1. Collection and timing of adverse events

Collect all adverse events, including serious adverse events (SAE), whether observed by the investigator or spontaneously reported by the subject, from the time of signing the informed consent to 30 days after the last dose. Bevacizumab-related adverse events will continue to be collected within 30-90 days after the last dose of Bevacizumab for patients receiving Bevacizumab.

No later than 30 days after the last dose, the investigator should report serious adverse events that are considered related to the study drug or study procedures.

7.5.2. Follow-up of adverse events

Adverse events should be followed up until they are recovered to baseline or Grade 0-1 or the investigator considers that no further follow-up is required for reasonable reasons (e.g., no recovery or improvement). If an adverse event cannot be recovered, a reasonable explanation should be recorded in the medical records, regardless of whether it is related to the study drug, and the recovery of the subject's AE or SAE and its date should be recorded in the eCRF and medical records.

7.5.3. Contents of Adverse Event Records

The investigator should fully record any adverse event, including diagnosis (if no diagnosis, record symptoms and signs including abnormal laboratory tests), start and stop dates and times (if applicable), CTCAE severity grade and change (Grade 3 or higher events), whether it is a serious adverse event, whether it is an adverse event of special interest, action taken with the study drug, treatment given due to the AE and the outcome of the event, and relationship of the adverse event to the study drug.

For serious adverse events, the investigator should also provide the date the AE

meets the criteria for an SAE, the date the investigator learns of the SAE, the rationale why the AE is an SAE, the hospitalization date, the discharge date, the probable cause of death, the date of death, whether an autopsy was performed, causality assessment with study procedures, causality assessment with other drugs, and other possible causes of the SAE. The investigator should also provide the judgment basis of relatedness and the description of SAE. In the SAE description, the subject's number, age, gender, height and weight should also be included; Indications and disease stages of the subjects treated with the investigational drug and relevant systemic conditions; Occurrence, development, outcome and outcome of clinical course of SAE; Laboratory test results related to SAE (test time, unit and normal range must be provided); Previous history and concomitant diseases related to SAE as well as their occurrence and duration; Medication history related to SAE, concomitant drugs and their treatment initiation, duration, usage and dosage, etc.; Details of initiation, duration, and administration of study drug.

The items regarding AE recording are described below:

Diagnosis, symptoms and signs

If a diagnosis is already available, the diagnosis should be recorded on the eCRF rather than the individual signs and symptoms (e.g., liver failure should be recorded rather than jaundice, elevated transaminases, and asterixis). If signs and symptoms cannot be ascertained to be caused by the diagnosis at the time of reporting, they will be recorded as a separate AE/SAE. If it is determined that the signs and symptoms are caused by the diagnosis, only the diagnosis is reported separately and the symptoms and signs are included in the diagnosis. AE needs to delete the record of symptoms and signs, and SAE needs to send follow-up update report.

Adverse Events Secondary to Other Events

In general, adverse events secondary to other events (e.g., caused by other events or clinical sequelae) should be recorded as the primary event, unless the secondary event is serious or serious. However, secondary events with significant clinical significance should be recorded as separate adverse events in the eCRF if they occur at a different time from the primary event. If the relationship between the events is unclear, they should be recorded separately in the eCRF.

Persistent or Recurrent Adverse Events

A persistent adverse event is an adverse event that persists without resolution between the subject's two evaluation time points.

A recurrent adverse event is an adverse event that has resolved between the two evaluation time points but occurs later. The occurrence of the event should be recorded separately in the eCRF.

Laboratory test abnormality

Clinically significant laboratory abnormalities should be reported as AE. It is the responsibility of the investigator to review all laboratory abnormalities and to make medical judgment as to whether each laboratory abnormality should be reported as an AE.

Pre-existing medical condition

The existing symptoms/signs of subjects during the screening period should be recorded and reported as adverse events only when the severity, frequency and nature of the symptoms/signs are aggravated (except for the deterioration of the disease condition under study) after entering the trial. Changes from the previous state such as "increased headache frequency" should be reflected in the recording.

Death

All deaths (except deaths due to disease progression) occurring throughout the trial, including the 30-day follow-up period after the last dose, regardless of relationship to the study drug, should be recorded in the death report form of the eCRF and reported to the sponsor in a timely manner by completing the SAE report form. Deaths within 30 days of the last dose are not required to be reported as SAE unless considered related to the study drug or study procedures.

When recording death events, if the cause of death is clear, the cause of death will be recorded as an adverse event, the outcome of which is death, and the event will be reported as an SAE; If the cause of death is unknown at the time of reporting, it should be recorded as "death of unknown cause" in the adverse event form of eCRF, and the "death of unknown cause" should be reported as SAE first, and the exact cause of death should be further investigated; Death due to progression of the disease under study itself was not reported as an AE or SAE.

Disease progression

Progressive disease is defined as worsening of the subject's condition due to the primary tumor targeted by the investigational drug, appearance of new lesions relative to the primary tumor, or progression of existing lesions. Disease progression is not to be reported as an AE. Death caused by signs and symptoms of disease progression, which is life-threatening, requires hospitalization or prolongation of hospitalization, results in permanent or significant disability/incapacity, results in congenital anomaly/birth defect, and other important medical events are not to be reported as SAE in an expedited manner.

Hospitalization, prolonged hospitalization, or surgery

Any adverse event that results in hospitalization or prolongation of hospitalization should be recorded and reported as an SAE, with the following exceptions:

- Planned hospitalization or prolongation of hospitalization as required by the protocol (e.g., for dosing, efficacy assessment, etc.).

- Hospitalization due to an unchanged medical condition that was present prior to study participation. E.g. Elective surgery/treatment scheduled prior to study entry.

However, if the condition of the pre-existing disease in the study deteriorates (e.g., surgery/treatment is performed earlier than originally planned), an elective surgery/treatment due to disease worsening will be considered an AE.

Overdose

An overdose was defined as the unintentional or intentional use of a dose in excess of that specified in the protocol. Overdose is not an adverse event per se, but may have contributed to the occurrence of an AE. Any overdose or incorrect use was to be recorded in the eCRF. Exceeding the protocol-specified dose is an overdose and should be reported in the eCRF. Any symptoms (regardless of severity) associated with an accidental or intentional overdose of IBI351 should be notified to the Pharmacovigilance Department of Xinda Biopharmaceuticals (Suzhou) Co., Ltd. Within 24 hours of awareness using the same reporting process as for SAE.

7.6. SAE, Pregnancy, and Hepatic Function Abnormal Event Reporting

7.6.1. SAE Reporting

Reporting period of SAE for serious adverse events that occur from the signing of informed consent to 30 days (inclusive) after the last dose. If a subject experiences an SAE, the investigator should immediately complete the Serious Adverse Event Report Form, sign and date the form after being informed of the SAE, and immediately report to the sponsor: [redacted] within 24 hours after being informed.

For deaths and life-threatening serious adverse events, the investigator should urgently follow up on missing information and provide a complete SAE report.

SAE occurring outside the above period should also be reported to the sponsor if they are considered related to the study drug.

7.6.2. Pregnancy Reporting

All subjects of childbearing potential participating in this clinical study must use effective contraception.

If a pregnancy occurs in a female subject exposed to the study drug during the clinical study, the investigator should report the pregnancy to the sponsor within 24 hours of becoming aware of the pregnancy and complete a Pregnancy Report Form.

If a male subject exposed to the study drug becomes pregnant during the clinical study, the subject may continue in the clinical study. The investigator should report the pregnancy to the sponsor within 24 hours of becoming aware of it and complete the

Pregnancy Report Form.

The investigator should continuously monitor and follow up the pregnancy outcome until 8 weeks after delivery of the mother, and report the outcome to the sponsor.

If the outcome of the pregnancy is stillbirth, spontaneous abortion, fetal anomaly (any congenital anomaly/birth defect), and induced abortion for medical reasons, it is considered as an SAE and needs to be reported according to the procedures and timelines for SAE.

If a subject experiences SAE during pregnancy, a Serious Adverse Event Report Form should be completed and reported according to the SAE reporting procedure.

7.6.3. Reporting of Hepatic Function Abnormal Events

Abnormalities in AST and/or ALT levels accompanied by abnormally elevated total bilirubin levels that meet the following conditions (1) (2) (3) and for which there is no other cause for the abnormality should always be reported as an SAE, following the SAE reporting process.

Table 16. Criteria for judging abnormal liver function tests

Condition satisfied	Judgment criteria
(1) Abnormal ALT or AST	1) Normal at baseline: ALT or AST > 3 × ULN during the treatment period<br>Abnormal at baseline: on-treatment ALT or AST > 3 × baseline level
(2) TBIL abnormal	2) Normal at baseline: TBIL > 2 × ULN during the treatment period<br>Abnormal at baseline: TBIL > 2 × baseline level during the treatment period
(3) Alkaline phosphatase < 2 × ULN (or no information available)	

Abbreviations: ALT=alanine aminotransferase, AST=aspartate aminotransferase, TBIL=total bilirubin, ULN=upper limit of normal.

If a subject has an abnormal AST and/or ALT level concurrent with an abnormally high total bilirubin level during the treatment or follow-up period, whenever possible, he/she should return to the study site for evaluation as soon as possible (preferably within 48 hours) after learning of the abnormal result. The evaluation should include laboratory tests, detailed history and physical examination, and should consider the possibility of liver neoplasia (primary or secondary).

In the event of hepatic impairment that meets the above criteria, appropriate dose modification or treatment discontinuation should be performed as described in Section 5.3.

8. Statistical Considerations

8.1. Statistical Analysis Plan

Statistical analysis of study data will be programmed using SAS v9.4 or higher. The statistical analysis methods will be described more specifically in the Statistical Analysis Plan (SAP).

8.2. Hypothesis testing

（1） Phase Ib

No formal statistical hypothesis testing will be performed in Phase Ib

[redacted]

[redacted]

[redacted]

[redacted]

[redacted]

[redacted]

[redacted]

[redacted]

[redacted]

[redacted]

[redacted]

[redacted]

[redacted]

[redacted]

[redacted]

[redacted]	[redacted]	[redacted]	[redacted]
[redacted]	[redacted]	[redacted]	[redacted]
	[redacted]	[redacted]	[redacted]
	[redacted]	[redacted]	[redacted]
	[redacted]	[redacted]	[redacted]
	[redacted]	[redacted]	[redacted]
[redacted]	[redacted]	[redacted]	[redacted]
[redacted] events: 292	[redacted]	[redacted]	[redacted]
	[redacted]	[redacted]	[redacted]
	[redacted] difference)	[redacted]	[redacted]
	Probability of Passing (when HR=1)	0.0137	0.0183
	Probability of Passing (when HR=0. 72)	0.6144	0.6545
Final Analysis<br>Sample Size: 514<br>Number of death events: 394	Z	2.0984	2.0093
	Nominal α (2-sided)	0.0359	0.0445
	HR (minimum detectable difference)	0.8095	0.8168
	Probability of Passing (when HR=1)	0.0400	0.0500
	Probability of Passing (when HR=0. 72)	0.8700	0.8884

8.3. Sample Size Estimation

Phase Ib

[redacted]

In the dose expansion phase, it is planned to expand the recommended dose group to 10 ~ 30 subjects in each cohort, with a total of 30 ~ 90 subjects in three cohorts to observe the safety and preliminary efficacy of the study treatment.

[redacted]

[redacted]

[redacted]

[redacted]

[redacted]

[redacted]	[redacted]
[redacted]	[redacted]
[redacted]	[redacted]

[redacted]

[redacted]	[redacted]
[redacted]	[redacted]

[redacted]

[redacted]	[redacted]
[redacted]	[redacted]
[redacted]	[redacted]
[redacted]	[redacted]
[redacted]	[redacted]

[redacted]

[redacted]	[redacted]
[redacted]	[redacted]
[redacted]	[redacted]
[redacted]	[redacted]
[redacted]	[redacted]
[redacted]	

[redacted]

[redacted]

[redacted]

[redacted]

[redacted]

[redacted]

[redacted]

[redacted]

[redacted]

[redacted]

[redacted]

[redacted]

[redacted]

[redacted]

[redacted].

8.5.2.2 Primary Analysis Method

Phase Ib

[redacted].

Primary Estimate Objective # 2: to summarize the number and percentage of subjects with secondarily confirmed objective response and calculate Clopper-Pearson 95% CI for ORR.

[redacted]

Phase Ib

Primary endpoint ORR:

Sensitivity Analysis	Unconfirmed ORR will be summarized using the same method as the primary analysis.

[redacted]

Primar[redacted]oint PFS:

[redacted]	[redacted]
[redacted]	[redacted]
[redacted]	[redacted]
[redacted]	[redacted]
[redacted]	[redacted]

[redacted]

[redacted]

[redacted]	[redacted]
[redacted]	[redacted]

8.5.3. Analysis of Secondary Efficacy Estimate Objectives

<u>Phase Ib</u>

PFS: The time from the first dose of study medication to the first occurrence of disease progression prior to initiation of new antineoplastic therapy or death due to any cause, as assessed by the investigator according to RECIST v1.1 criteria, whichever occurs first. Initiation of new antineoplastic therapy prior to a PFS event will be censored

at the last adequate imaging assessment prior to initiation of new antineoplastic therapy. The effect of treatment discontinuation for any reason was ignored.

Kaplan-Meier method will be used to estimate the median PFS, and Broolmeyer-Crowley 95% CI will be provided to plot the survival curve; Progression-free survival rates at different time points were estimated.

OS: Time from first dose of study medication to death due to any cause. Discontinuation of treatment for any reason or the impact of new antineoplastic therapy was ignored. The analysis method is the same as for PFS indicator.

DCR: The proportion of subjects in the analysis population with a best response of complete response (CR), partial response (PR), or stable disease (SD) as assessed by the investigator according to RECIST v1.1 from the first dose of study drug to the start of new antineoplastic therapy, first disease progression, or death, whichever occurs first.

The number and percentage of subjects with best response of CR, PR, or SD will be summarized, and Clopper-Pearson 95% CI for DCR will be calculated.

DoR: defined as the time from the first documented CR or PR to the first disease progression before the start of new antineoplastic therapy or death due to any cause, as assessed by the investigator according to RECIST v1.1 criteria, for subjects with CR or PR. Derivation rules and analysis methods are the same as for PFS metrics.

TTR: defined as the time from the first dose of study drug to the first documented CR or PR, as assessed by the investigator according to RECIST v1.1 criteria, for subjects with CR or PR. TTR indicators will be summarized descriptively, including number of cases, mean, standard deviation, median, minimum and maximum.

[redacted]

[redacted]

[redacted]

[redacted]

[redacted].

[redacted]

[redacted]

[redacted]

[redacted]

[redacted]

8.5.4. Safety Analysis

Safety data will be statistically analyzed by dose group, dose regimen, and study phase (Phase Ib/Phase III). All safety analyses will be analyzed according to the SS, except for DLT analysis according to the DDS.

8.5.4.1. Drug Exposure

Based on SS, total dose of study drug actually taken by subjects, duration of exposure, dose intensity, and relative dose intensity were summarized and listed. The number and percentage of subjects in each relative dose intensity category were summarized.

8.5.4.2. Adverse Events

Adverse events will be coded according to the Medical Dictionary for Regulatory Activities (MedDRA) and processed in the statistical analysis. The number and percentage of subjects with treatment-emergent adverse events (TEAE), including all TEAE, TEAE related to the study drug, serious adverse events during the treatment period, TEAE leading to discontinuation of study treatment, TEAE leading to interruption of study treatment, and TEAE leading to dose modification, were calculated by treatment group. Adverse events of the above categories will be summarized by System Organ Class (SOC), Preferred Term (PT) and/or CTCAE grade, and a list of adverse events will be provided.

8.5.4.3. Laboratory Tests

Descriptive statistics will be performed for laboratory test results and changes from baseline by visit. Shifts in laboratory test results from pre-to post-dose will be described using cross-categorical tables. A listing of laboratory test results was provided for all

subjects.

8.5.4.4. 12-lead ECG examination

Descriptive statistics will be performed for 12-lead ECG results and changes from baseline by visit. Changes in clinical judgment results before and after administration were summarized. A listing of 12-lead ECG results will be provided for all subjects.

In Phase Ib, 12-lead ECG will be performed 3 times (at least 5 minutes apart) at screening, and the mean of the 3 times will be used as the baseline value (see Section 6.1.2.3).

8.5.4.5. Vital Signs, Physical Examinations, and Other Safety-Related Tests

Observed values and changes from baseline will be analyzed using descriptive statistics for each vital sign and other safety-related quantitative values by visit. The baseline and post-baseline results of qualitative indicators will be presented in the form of cross-tabulations. Listings of vital signs, physical examinations, and other safety data were presented for all subjects.

ECOG PS will be analyzed and summarized using descriptive statistics.

8.5.5. Compliance Analysis

Compliance with the protocol will be assessed by summarizing the number and proportion of subjects with major protocol deviations, including the proportion and frequency of subjects who violate the intended dosing regimen. Duration of exposure, actual cumulative dose, and dose intensity and relative dose intensity, duration of administration (number of cycles) were summarized for each exposure.

Study drug compliance will be assessed by summarizing the percentage of subjects with dose reductions, delays, interruptions, or discontinuations and their reasons.

8.5.6. Baseline characteristics of subjects

Descriptive statistics of demographic characteristics (gender, age, height, weight, body mass index, etc.), information on tumor diagnosis and treatment (pathological diagnosis, clinical staging, previous treatment, etc.), medical history, previous and concomitant treatment, etc.

8.5.7. PK Analysis

Population pharmacokinetic characteristics of IBI351 will be analyzed using nonlinear mixed effects kinetic modeling to estimate population PK parameters and, if necessary, descriptive statistics will be performed for plasma concentrations and PK parameters at each time point in Phase Ib, including but not limited to Cmax, AUC, t1/2, CL/F, V/F.

8.5.8. Analysis of Other Exploratory Endpoints

Quality of life indicators (Phase III): Standardized transformation of quality of life scores will be performed. Descriptive statistics will be performed for the results by treatment group, and appropriate statistical methods will be used for inter-group comparison.

Biomarkers (Phase Ib/III): Descriptive statistics of gene mutation/fusion in tumor tissues of subjects will be performed to explore the potential relationship between the above indicators and efficacy.

[redacted]

[redacted]

[redacted]

[redacted]

[redacted]

[redacted]

[redacted]

[redacted]

8.6. Control of bias

8.6.1. Randomization and blinding

Not applicable for Phase Ib.

[redacted]

[redacted]

The study was open-label and the investigator, sponsor, and subjects were not blinded.

8.6.2. Assessment of Blinding Maintenance

Not applicable.

8.6.3. Unblinding and Emergency Unblinding

Not applicable.

9. Study Quality Assurance and Quality Control

In accordance with the guidelines of Good Clinical Practice (GCP), the sponsor is responsible for implementing and maintaining a quality assurance and quality control system according to corresponding standard operating procedures to ensure that the conduct of the clinical trial and the collection, recording and reporting of data comply with the protocol, GCP and applicable regulatory requirements.

9.1. Clinical Monitoring

The sponsor or a contract research organization (CRO) authorized by the sponsor will perform clinical monitoring of this study. CRAs should perform monitoring in accordance with the standard operating procedures of the Sponsor or CRO and have the same rights and responsibilities as the Sponsor's CRAs. The monitor should maintain regular communication with the investigator and the sponsor.

Prior to the start of the study, the monitor will assess the competence of each study

site and report relevant problems with facilities, technical equipment, or medical personnel to the sponsor. During the study, the monitor will be responsible for monitoring whether the investigator has obtained written informed consent from all subjects and whether the data records are correct and complete. At the same time, the monitor will also compare the data entered into the eCRF with the original data and inform the investigator of any errors or omissions. The monitor will also supervise the study site for protocol compliance, arrange for the supply of study drug, and ensure that the drug is stored under appropriate conditions.

Monitoring visits will be conducted as required by applicable laws and regulations. Beginning with subject enrollment, each site will undergo regular monitoring visits. After each visit to the investigator, the monitor should submit a written report to the sponsor.

9.2. Data Management

Electronic Data Capture (EDC) system will be used in this study, and study data will be entered into the eCRF by the investigator or authorized study personnel. Prior to site initiation or data entry, the investigator and authorized study personnel will be appropriately trained and appropriate security measures will be taken for the computers and other equipment used.

Data entry into the eCRF should be completed as soon as possible during or after the visit and updated at any time to ensure that it reflects the latest developments of the subjects participating in the study. To avoid differences in the assessment of results by different evaluators, it is recommended that baseline and all subsequent efficacy and safety assessments for the same subject be performed by the same person. The investigator was required to review the data to ensure the accuracy and validity of all data entered into the eCRF. If certain assessments are not performed during the course of the study, or certain information is not available, not applicable, or unknown, the investigator should record it in the eCRF. The investigator should electronically sign the data after verification.

The Clinical Research Associate (CRA) will review the eCRFs against the source documents and assess their completeness and consistency, and the CRA will compare the eCRFs with the source documents to ensure the consistency of key data. All data entries, corrections, and modifications will be the responsibility of the Investigator or his/her designee. Data from the eCRF will be submitted to the EDC database and any changes to the data will be recorded in the audit trail, i.e. The reason for the modification, operator username, date and time of the modification will be recorded. The roles and permissions of the site personnel responsible for data entry will be pre-determined. In case of any data query, CRA or data management personnel will issue the query in EDC, and relevant personnel of the study site will be responsible for answering the query. The EDC system will record the audit trail of queries, including user name, time, and date.

Unless otherwise specified, the eCRF will only be used as a form for data collection and not as source data. Source documents are all records used by the investigator or the hospital, related to the subject and capable of proving the existence of the subject, inclusion and exclusion criteria and their participation in this study, including laboratory records, ECG results, medication records and subject folders.

The investigator is responsible for maintaining all source documents and for monitoring them by the CRA at each visit. In addition, the investigator was required to submit a completed eCRF for each enrolled subject, regardless of the duration of the enrolled subject's participation in the study. All supporting documents (e.g., laboratory or hospital records) submitted with the eCRF should be carefully verified for the protocol number and subject number, and all personal privacy information (including subject name) should be deleted or illegible to protect subject privacy. The investigator certifies by electronic signature that he/she has reviewed all eCRF data to ensure the validity, completeness, and accuracy of the data. The electronic signature will be completed using the user ID and password of the investigator. The date and time of the signature will be automatically attached by the system. The investigator may not share the user ID and password with other personnel. Changes to data in the eCRF should be made according to the workflow defined in the EDC system. All changes and reasons for changes will be documented in the audit trail.

9.3. Quality Assurance Audit

Quality assurance audits of the study site, study database, and associated study documents may be conducted by the Sponsor or an authorized representative of the Sponsor during the course of the study, and inspections of the study site, study database, and associated study documents may also be conducted at the discretion of the appropriate regulatory authorities. When notified of an inspection by a regulatory authority, the investigator was required to notify the sponsor immediately.

Site audits were conducted by the Sponsor's Quality Assurance Unit. Audits included: drug supplies, required trial documents, records of the informed consent process, and consistency of the case report forms with source documents. Audit content and scope may also be added as appropriate. After reasonable notice, the investigator shall allow auditors entrusted by the sponsor to conduct trial-related audits and inspections by regulatory authorities. The main purpose of the audit or inspection is to verify that the rights or health of the subjects participating in the trial are protected, that the informed consent is signed and the trial process is properly conducted, and that all data related to the evaluation of the study drug are handled and reported in accordance with the pre-planned arrangement, protocol, facilities, ethical standard operating procedures, GCP and applicable regulatory requirements. The investigator should have direct access to all trial documents, original records and raw data.

10. Ethics

10.1. Ethics Committee

The sponsor or its authorized representative will prepare relevant documents to be submitted to the Ethics Committee (EC) of the study site, including the trial protocol, informed consent form, investigator's brochure, subject recruitment materials or advertisements and other documents required by regulations, and submit them to the corresponding EC for review and approval. Written approval from the EC must be obtained and provided to the Sponsor prior to initiation of the study. The EC approval letter must clearly describe the name, number and version number of the study protocol and the version number of other documents (such as informed consent form) and approval date. The Investigator was required to notify the Sponsor of the EC's written comments on the delay, suspension and re-approval.

The site must comply with the requirements of the site's EC. May include protocol amendments, informed consent form amendments and subject recruitment materials amendments to be submitted to EC for review and approval, local safety reporting requirements, periodic reports and updates according to EC regulations, and final report submission. All of the above documents and EC approvals must be provided to the Sponsor or its designee.

10.2. Ethical conduct in the study

The study process and informed consent shall comply with the Declaration of Helsinki, relevant GCP requirements and relevant laws and regulations of China concerning drug and data protection.

GCP provides ethical, scientific and global quality standards for the design, conduct, recording, and reporting of clinical studies involving human subjects. This study will be conducted in accordance with GCP and relevant national regulations and in accordance with the relevant ethical principles in the Declaration of Helsinki to protect the rights, safety and well-being of the subjects.

The investigator is required to comply with the procedures specified in this trial protocol and shall not change them without the permission of the sponsor. Any protocol deviations will be reported to the EC, the Sponsor, or regulatory authorities.

10.3. Subject Information and Informed Consent

Prior to any study procedures, the possible risks and benefits of the study will be explained to potential subjects using an informed consent form (ICF) that will be easily understood. The ICF statement should specify that the informed consent is voluntary and the possible risks and benefits of participating in the study should be specified, and the subject may withdraw from the study at any time. The investigator can only enroll a subject after fully explaining the details of the study, satisfactorily answering the subject's

questions and giving sufficient time for consideration, and obtaining the written consent of the subject or his/her legal representative. All signed informed consent forms must be in the investigator's file or in the subject's folder.

The investigator is responsible for explaining the content of the informed consent to the subject and obtaining the informed consent form signed and dated by the subject or his/her legally acceptable representative prior to the start of the study. After signing, the investigator should send a signed informed consent form to the subject. The investigator should record the informed consent process in the trial source documents.

The initial informed consent form, any subsequent amendments to the written informed consent form, and any written information provided to subjects should be subject to IRB/IEC opinion prior to use. If new information becomes available that may be relevant to the subject's willingness to continue participation in the trial, the subject or his/her legally acceptable representative should be informed in a timely manner. Communication of this information will be provided and documented via a revised informed consent form or an addendum to the original informed consent form (obtaining the subject's dated signature or the subject's legally acceptable representative's dated signature).

10.4. Data Protection

Information on data protection and privacy will be included in the ICF (or, in some cases, along with the use of separate documents).

Precautions were taken to ensure the confidentiality of documents and to prevent identification of subjects. However, under special circumstances, some individuals may see genetic data and personal identification codes for a subject. For example, in the event of a medical emergency, the sponsor, its representative physician, or investigator will be aware of the subject identification code and have access to the subject's genetic data. In addition, access to relevant documents is required by the relevant regulatory authorities.

10.5. Protocol Deviations

A protocol deviation was defined as any non-compliance with the clinical trial protocol, International Conference on Harmonisation Good Clinical Practice (ICH GCP), or operating manual requirements. Non-compliance may come from the subject, investigator, or site personnel. In response to violations, corrective actions shall be taken and completed in a timely manner.

11. Study Management

11.1. Data Handling and Record Retention

The documents in the clinical trial (protocol and protocol amendment, completed eCRF, signed ICF, etc.) should be kept and managed in accordance with the requirements

of GCP. The site should retain these documents for 5 years after the end of the study.

Study documents should be properly retained for future access or data traceability. Safety and environmental risks should be considered when preserving documents.

No study documents will be destroyed without the written permission of the Sponsor and Investigator. Only after notifying and obtaining written consent from the Sponsor, the Investigator/study site may transfer the study documents to other parties that comply with the document retention requirements or to other locations that meet the requirements for storage.

11.2. Access to Raw Data/Documents

The Investigator agrees that the Sponsor, CRO and relevant authorized regulatory authorities have direct access to all study-related documents, including the subject's medical records.

11.3. Protocol Amendment

All amendments to the protocol made during the course of the study were to be communicated and agreed upon by the sponsor and the investigator. The sponsor shall ensure that protocol amendments are submitted to regulatory authorities in a timely manner.

All amendments to the protocol will be retained as protocol supplements. Any amendment to the protocol should be submitted to the Ethics Committee for approval or filing according to the provisions of the Ethics Committee. If required, it should also be submitted to regulatory authorities for approval and, if required, approved by the EC and regulatory authorities before implementation (except for changes to the protocol to eliminate an immediate hazard to trial subjects).

11.4. Investigator Responsibilities

The investigator will carry out this study in accordance with the protocol, ethical principles in the Declaration of Helsinki, China GCP and relevant laws and regulations.

The detailed responsibilities of the relevant investigators are listed in Chapter 5 of the China GCP (2020 No.57).

11.5. Publication Policy

If the Sponsor and the Investigator agree to publish an academic paper based on the results of the study, please write this subsection in accordance with the relevant agreement. This subsection may be deleted if there is no agreement for publication of academic papers.

All data generated in this study are confidential information of the Sponsor and the Sponsor has the right to publish the results of the study. Information on the publishing

policies of the sponsor and investigators will be described in the clinical trial agreement.

All information related to this trial (not limited to the following documents: protocol and investigator's brochure) must be strictly confidential. The investigator must be aware that the scientific or medical conclusions drawn from this trial may be of commercial value to the sponsor. The investigator shall keep the information and data related to this trial confidential. If the data related to this trial or the conclusions drawn from the trial are to be published publicly, the investigator shall negotiate with the sponsor in advance and obtain the written consent of the sponsor. In order to protect their own rights and interests, the sponsor may require the investigator not to publish relevant trial data before the marketing approval of the investigational product is obtained.

The sponsor has the right to publish or publish information or data related to this trial or to report it to the drug regulatory authorities. The sponsor shall obtain the consent of the investigator if the name of the investigator is required to appear in the publication, publication or advertisement.

11.6. Finance and Insurance

The Sponsor will purchase insurance for subjects participating in this study in accordance with local regulations and minimum requirements. The terms of the insurance will be kept in the study binder.

12. References

[1] https://publications.iarc.fr/Databases/Iarc-Cancerbases/GLOBOCAN-2012-Estimated-Cancer-Incidence-Mortality-And-Prevalence-Worldwide-In-2012-V1.0-2012.

[2] Center for Chronic Non-communicable Disease Prevention and Control, Chinese Center for Disease Control and Prevention, Statistical Information Center, National Health and Family Planning Commission. China Cause of Death Surveillance Dataset 2013 [M]. Beijing: Science Popularization Press, 2015; 58.

[3] Zheng Rongshou, Sun Kexin, Zhang Thinking, Zeng Hongmei, Zou Xiaonong, Chen Ru et al. Analysis on the prevalence of malignant tumors in China in 2015. Chinese Journal of Oncology, 2019; 41 (1): 19-28.

[4] Chen W, Zheng R, Baade P D, et al. Cancer statistics in China, 2015. CA Cancer J Clin. 2016; 66 (2): 115-32.

[5] Zhang Yue, Shi Jufang, Huang Huiyao, Ren Jiansong, Li Ni, Dai Min. Burden of Disease of Colorectal Cancer in Chinese Population. Chinese Journal of Epidemiology, 2015, 36 (7): 709-714.

[6] Medical Administration Bureau of Health and Family Planning Commission of the People's Republic of China, Chinese Medical Association Oncology Branch. Chinese Standards for Diagnosis and Treatment of Colorectal Cancer (2017 Edition)

(Excerpt). Journal of Comprehensive Oncology Therapy (Electronic), 2018; 4 (12): 29-37.

[7] Peeters M, Price TJ, Cervantes A, et al. Final results from a randomized phase 3 study of FOLFIRI {+/-} panitumumab for second-line treatment of metastatic colorectal cancer. Ann Oncol. 2014; 25 (1): 107-16.

[8] Kubicka S, Greil R, Andr é T, et al. Bevacizumab Plus Chemotherapy Continued Beyond First Progression in Patients with Metastatic Colorectal Cancer Previous Treatment with Bevacizumab Plus Chemotherapy: ML18147 STUDY KRAS SUBGROUP FINDINGS. Ann Oncol. 2013; 24 (9): 2342-9.

[9] Fossella FV, DeVore R, Kerr RN, et al. Randomised phase III trial of docetaxel versus vinorelbine or ifosfamide in patients with advanced non-small-cell lung cancer previously treated with platinum-containing chemotherapy regimens. The TAX 320 Non-Small Cell Lung Cancer Study Group. J Clin Oncol. 2000; 18 (12): 2354-62.

[10] Canon J, Rex K, Saiki AY, et al. The Clinical KRAS (G12C) Inhibitor AMG 510 Drives Anti-Tumour Immunity. Nature, 2019; 575 (7781): 217-223.

[11] Fell JB, Fischer JP, Baer BR, et al. Identification of the Clinical Development Candidate MRTX849, a Covalent KRAS (G12C) Inhibitor for the Treatment of Cancer. J Med Chem, 2020; 63 (13): 6679-6693.

[12] Fakih MG, Kopetz S, Kuboki Y, et al. Sotorasib for previous treated colorectal cancers with KRASG12C mutation (CodeBreaK100): a specific analysis of a single-arm, phase 2 trial. Lancet Oncol. 2022; 23 (1): 115-124.

[13] Di Nicolantonio F, Vitiello P P, Marsoni S, et al. Precision oncology in metastatic colorectal cancer-from biology to medicine. Nat Rev Clin Oncol. 2021; 18 (8): 506-525.

[14] Weiss J, Yaeger R D, Johnson M L, et al. LBA6 KRYSTAL-1: Adagrasib (MRTX849) as monotherapy or combined with cetuximab (Cetux) in patients (Pts) with colorectal cancer (CRC) Harboringa KRASG12C mutation. Annals of Oncology, 2021; 32 (suppl_5): S1283-S1346.

[15] Marwan G. Fakih, Gerald S, et al. CodeBreaK101 Subprotocol H: Phase 1b Study Evaluating Combination of Sotorasib, a KRASG12C Inhibitor, and panitumumab (PMab), an EGFR Inhibitor, in Advanced KRAS p.G12C-Mutated Colorectal Cancer (CRC). Annals of Oncology, 2021; 32 (suppl_5): S530-S582.

[16] Tabernero J, Bendell J, Corcoran R, et al. P-71 KRYSTAL-10: A randomized phase 3 study of adagrasib (MRTX849) in combination with cetuximab vs chemotherapy in patients with previously treated advanced colorectal cancer with KRASG12C mutation. Annals of Oncology, 2021; 32: S121.

[17] Moore AR, Rosenberg SC, McCormick F, et al. RAS-targeted therapies: is the undruggable drugged? . Nat Rev Drug Discov, 2020; 19 (8): 533-552.

[18] Kim D, Xue JY, Lito P. Targeting KRAS (G12C): From Inhibitory Mechanism to Modulation of Antitumor Effects in Patients. Cell, 2020; 183 (4): 850-859.

[19] Downward J. Targeting RAS signalling pathways in cancer therapy. Nat Rev Cancer, 2003; 3 (1): 11-22.

[20] Waters AM, Der CJ. KRAS: The Critical Driver and Therapeutic Target for Pancreatic Cancer. Cold Spring Harb Perspect Med, 2018; 8 (9): a031435.

[21] Prior IA, Lewis PD, Mattos C. A comprehensive survey of Ras mutations in cancer. Cancer Res, 2012; 72 (10): 2457-2467.

[22] Ostrem JM, Shokat KM. Direct small-molecule inhibitors of KRAS: from structural insights to mechanism-based design. Nat Rev Drug Discov, 2016; 15 (11): 771-785.

[23] Dias Carvalho P, Guimarães CF, Cardoso AP, et al. KRAS Oncogenic Signaling Extensions beyond Cancer Cells to Orchestrate the Microenvironment. Cancer Res, 2018; 78 (1): 7-14.

[24] https://www.fda.gov/drugs/resources-information-approved-drugs/fda-approval-new-dosing-regimen-cetuximab.

[25] Matsuda A, Yamada T, Jamjitstrong S, et al. Comparison Between Biweekly and Weekly Cetuximab in Patients with Metastatic Colorectal Cancer: A Meta-analysis. Anticancer Res. 2020; 40 (6): 3469-3476.

[26] Maurer W, Bretz F. Multiple testing in group sequential trials using graphic applications. Stat Biopharm Res 2013; 5 (4): 311-20.

[27] Zhou H, Lee JJ, Yuan Y. BOP2: Bayesian Optimal Design for Phase II Clinical Trials with Simple and Complex Endpoints. Stat Med. 2017; 36 (21): 3302-3314.

[28]

13. Appendices

Appendix 1: Protocol Amendment History

Attached Table 1. Protocol Amendment History

Version	Date	Description of Major Changes	Remarks
V1.0	02Mar2022	N/A	Added protocol
V2.0	18JUL2022	See Protocol Amendment Record	

Appendix 2: Performance Status Scoring Criteria (ECOG PS)

**Scores**	**Standard**
0	Fully normal mobility without any difference from pre-onset mobility
1	Restricted in physically strenuous activity but ambulatory and able to carry out activities of a light or sedentary nature, including light house work or office work
2	Ambulatory and self-care, incapacitated, able to get up for not less than half of the day
3	Capable of only partial self-care and confined to bed or wheelchair more than half of the day
4	Bedridden and unable to take care of themselves
5	Death

Appendix 3: Response Evaluation Criteria in Solid Tumors version 1.1 (RECIST v1.1)

The following are excerpts from RECIST v1.1 criteria.

1. Measurability of tumor at baseline

1.1 Definitions

At baseline, tumor lesions/lymph nodes will be classified as measurable or non-measurable according to the following definitions:

1.1. 1 Measurable disease

Tumor lesions: at least one diameter that can be accurately measured (to be recorded as the largest diameter) with the following minimum lengths:

- 10 mm on CT scan (CT scan slice thickness no greater than 5mm)
- 10 mm by clinical routine examination (tumor lesions that cannot be accurately measured with calipers should be recorded as non-measurable)
- Chest X-ray 20 mm
- Malignant lymph nodes: Pathologically enlarged and measurable, a lymph node must be ≥ 15 mm in the short axis by CT scan (CT scan slice thickness recommended to be no greater than 5 mm). At baseline and follow-up, only the short axis will be measured and followed.

1.1. 2 Non-measurable disease

All other lesions, including small lesions (longest diameter < 10 mm or pathological lymph nodes ≥ 10 mm to < 15 mm short axis) and non-measurable lesions. Non-measurable lesions include meningeal disease, ascites, pleural or pericardial effusion, inflammatory breast cancer, lymphangitic carcinomatosis of the skin/lung, abdominal masses that cannot be confirmed by imaging and followed up, and cystic lesions.

1.1. 3 Special Considerations for Lesion Measurements

Bone lesions, cystic lesions and lesions previously treated with local therapy should be specifically noted:

Bone lesions:

- Bone scan, PET scan or plain film are not suitable for measuring bone lesions, but can be used to confirm the presence or disappearance of bone lesions;
- Lytic bone lesions or mixed lytic/blastic lesions with identified soft tissue components that meet the definition of measurability described above can be considered

as measurable lesions if they can be evaluated with cross-sectional imaging techniques such as CT or MRI;

- Blastic lesions are non-measurable.

Cystic lesions:

- Lesions that meet the criteria for radiographically defined simple cysts should not be considered malignant lesions because they are simple cysts by definition and are neither measurable nor non-measurable lesions;

- Cystic metastases can be considered as measurable lesions if they meet the definition of measurability described above. However, if non-cystic lesions are present in the same patient, they should be preferred as target lesions.

Locally treated lesions:

- Lesions located in sites that have been irradiated or other loco-regional therapy are generally considered non-measurable unless there is unequivocal progression in the lesion. The protocol should describe in detail the conditions under which these lesions are measurable.

1.2 Description of measurement method

1.2. 1 Lesion Measurements

All tumor measurements will be recorded in the metric system at the time of clinical evaluation. All baseline assessments of tumor lesion size should be performed as close as possible to the start of treatment and must be completed within 28 days (4 weeks) prior to the start of treatment.

1.2. 2 Evaluation method

The same techniques and methods should be used for baseline and subsequent measurements of lesions. All lesions must be evaluated by imaging, except those that cannot be evaluated by imaging but can only be evaluated by clinical examination.

Clinical lesions: Clinical lesions should be considered measurable only if they are superficial and ⩾ 10 mm in diameter when measured (e.g., skin nodules, etc.). For subjects with skin lesions, it is recommended that a color photograph containing a ruler to measure the size of the lesion be used for archiving. When lesions are evaluated by both imaging and clinical examination, imaging evaluation should be selected whenever possible because imaging is more objective and can be repeated at the end of the study.

Chest X-ray: Chest CT should be preferred when tumor progression is an important endpoint because CT is more sensitive than X-ray, especially for new lesions. Chest X-ray is indicated only if the lesion being measured is well-defined and the lungs are well ventilated.

CT, MRI: CT is currently the best reproducible method available for response assessment. The definition of measurability in this guideline is based on CT scan slice thickness ≤ 5 mm. If CT slice thickness is greater than 5 mm, the minimum size of a measurable lesion should be twice the slice thickness. MRI may also be acceptable in some cases (e.g., full body scans).

Ultrasound: Ultrasound should not be used as a method of measurement to measure lesion size. Ultrasonography is not repeatable after the end of the measurement due to its operational dependency and does not guarantee the identity of the technique and measurement between different measurements. If a new lesion is identified by ultrasound during the trial, it should be confirmed by CT or MRI. If radiation exposure from CT is considered, MRI may be used instead.

Endoscopy, laparoscopy: The use of these techniques for objective tumor assessment is not recommended, but they can be used to confirm CR when biopsies are obtained and to confirm recurrence in trials where recurrence after CR or surgical resection is an endpoint.

Tumor markers: Tumor markers alone cannot be used to evaluate objective tumor response. However, if the marker level is above the upper limit of normal at baseline, it must return to normal for evaluation of complete response. Because tumor markers are disease-specific, this factor needs to be taken into account when the criteria for measurement are written in the protocol. Specific criteria for CA-125 response (in recurrent ovarian cancer) and PSA response (in recurrent prostate cancer) have been published. The Gynecologic Cancer Intergroup has developed CA-125 progression criteria that will be added to the objective tumor assessment criteria for first-line treatment of ovarian cancer.

Cytology/histology techniques: These techniques can be used to identify PR and CR in specific circumstances as specified in the protocol (e.g., residual benign tumor tissue is often present in lesions of germ cell tumors). Cytological confirmation of the appearance or worsening of tumor-related effusions during treatment can be used to differentiate between response (or stable disease) and progressive disease when effusions may be a potential side effect of a therapy (e.g., treatment with taxane compounds or angiogenesis inhibitors) and the measurable tumor meets criteria for response or stable disease.

2 Tumor Response Assessment

2.1 Assessment of All Tumors and Measurable Lesions

To evaluate objective response or possible future progression, it is necessary to have a baseline assessment of the total tumor burden of all tumor lesions as a reference for subsequent measurements. In clinical protocols where objective response is the primary

endpoint, only subjects with measurable disease at baseline can be enrolled. Measurable disease is defined as the presence of at least one measurable lesion. For trials where disease progression (time to progression or extent of progression on a fixed date) is the primary endpoint, the protocol inclusion criteria must specify whether subjects with measurable disease are limited to enrollment or subjects without measurable disease are eligible.

2.2 Baseline Documentation of Target and Non-Target Lesions

When more than one measurable lesion is present at baseline, all lesions up to 5 in total (no more than 2 per organ) should be recorded and measured as target lesions representing all involved organs (i.e., a maximum of two or four target lesions will be selected as baseline lesions for subjects with only one or two cumulative organs).

Target lesions must be selected on the basis of size (longest diameter), representative of all involved organs, and must be reproducibly measured. Sometimes when the largest lesion cannot be measured reproducibly, the largest lesion that can be measured reproducibly may be re-selected.

Lymph nodes require special attention because they are normal tissue and can be detected by imaging even in the absence of tumor metastasis. Pathological lymph nodes defined as measurable nodes and even target lesions must meet the following criteria: short axis ⩾ 15 mm by CT. Only the short diameter needs to be tested at baseline. The short diameter of a node is usually used by radiologists to determine whether the node has metastasized. The nodal size is usually expressed in two dimensions (axial plane for CT and one plane from the axial, sagittal, or coronal plane for MRI) as measured by imaging. The minimum value is the short diameter. For example, a 20 mm × 30 mm abdominal node with a short axis of 20 mm can be considered a malignant, measurable node. In this example, 20 mm is the measurement of the node. Nodes ⩾ 10 but < 15 mm should not be considered target lesions. Nodules < 10 mm are not considered pathological and need not be recorded and further observed.

The sum of the diameters of all target lesions (longest for non-nodal lesions and short axis for nodal lesions) will be calculated and reported as the baseline sum diameters. If lymph node diameters are included, only the short diameter will be included as mentioned above. The baseline sum diameters will be used as a reference for the baseline level of disease.

All remaining lesions, including pathological lymph nodes, may be considered non-target lesions and need not be measured, but should be recorded at baseline assessment. If recorded as "present", "absent" or in rare cases "unequivocal progression". Widespread target lesions may be recorded together with target organs (e.g., large enlarged pelvic lymph nodes or large liver metastases).

2.3 Response Criteria

2.3. 1 Target Lesion Assessment

Complete Response (CR): Disappearance of all target lesions and reduction in short axis of all pathological lymph nodes (both target and non-target) to < 10 mm.

Partial Response (PR): at least a 30% decrease from baseline in the sum of diameters of target lesions.

Progressive disease (PD): at least a 20% relative increase in the sum of diameters of target lesions, taking as reference the smallest sum of diameters of all target lesions measured throughout the study (the baseline value will be taken as reference if the baseline value is the smallest); In addition, an absolute increase of at least 5 mm in the sum of diameters must be met (the appearance of one or more new lesions is also considered progressive disease).

Stable disease (SD): Neither a decrease to the level of PR nor an increase to the level of PD in target lesions, taking as reference the smallest sum diameters on study.

2.3. 2 Considerations for Target Lesion Assessment

Lymph nodes: Even if lymph nodes identified as target lesions decrease to within 10 mm, the actual short axis value corresponding to baseline should be recorded at each measurement (in the same anatomical plane as the baseline measurement). This means that if a lymph node is a target lesion, it cannot be said that the lesion has completely disappeared even if the criteria for complete response are met, since the short axis of a normal lymph node is defined as < 10 mm. Target nodal lesions should be recorded in a specific location on the eCRF or other recording method: for CR, all nodal short axis must be < 10 mm; For PR, SD and PD, the actual short axis measurement of target nodes will be included in the sum of the diameters of target lesions.

Target lesions that are too small to measure: in clinical studies, all lesions (nodal or non-nodal) recorded at baseline should have their actual measurements recorded again at subsequent assessments, even if they are very small (e.g., 2 mm). But sometimes it may be too small to make the CT scan so blurry that the radiologist has difficulty defining the exact value and may report it as "too small to measure." When this occurs, it is important to record the previous value on the eCRF form. If in the opinion of the radiologist, the lesion may have disappeared, it should also be recorded as 0 mm. If a lesion is present but blurry and cannot be accurately measured, the default value is 5 mm. (Note: Lymph nodes are unlikely to be present because they typically have a measurable size under normal conditions or are often surrounded by adipose tissue as they are in the retroperitoneum; however, if such a situation does not allow measurement, the default value is 5 mm). The default value of 5 mm is derived from the cut thickness of the CT scan (this value does not change according to the different cut thickness values of CT).

Providing this default value will reduce the risk of erroneous evaluation since the same measurement is not likely to be repeated. However, it should be reiterated that if the radiologist is able to provide an exact value for the size of the lesion, the actual value must be recorded even if the lesion is less than 5 mm in diameter.

Separated or Combined Lesions: When a non-nodal lesion splits into fragments, the longest diameters of the separate portions are added together to calculate the sum of the diameters of the lesions. Similarly, for coalescent lesions, the planes between the coalescent segments can be used to distinguish them, and the respective maximum diameter can be calculated. However, if the combination is inseparable, the longest diameter should take the longest diameter of the whole coalescing lesion.

2.3. 3 Assessment of Non-Target Lesions

This section defines tumor response criteria for non-target lesions. Although some non-target lesions are actually measurable, they do not need to be measured and only need to be assessed qualitatively at the time points specified in the protocol.

Complete Response (CR): Disappearance of all non-target lesions and normalization of tumor markers. All lymph nodes are non-pathological in size (< 10 mm short axis).

Non-CR/Non-PD: Persistence of one or more non-target lesions and/or persistence of tumor marker level above normal.

Progressive Disease: Unequivocal progression of existing non-target lesions. Note: The appearance of one or more new lesions is also considered progressive disease.

2.3. 4 Special Considerations for Assessment of Progression of Non-Target Lesions

The additional explanation for the definition of progression of non-target disease is as follows: When a subject has measurable non-target disease, to define unequivocal progression on the basis of non-target disease, even if the target disease is assessed as stable or partial response, the overall worsening in non-target disease must be sufficient to warrant discontinuation of treatment. A general increase in the size of one or more non-target lesions is often not sufficient to meet the criteria for progression; therefore, it is almost rare for a change in non-target disease alone to define overall tumor progression in the presence of stable or partial response of target disease.

When a subject has non-measurable non-target disease: This occurs in some Phase 3 trials when the inclusion criteria do not specify that measurable disease must be present. The overall assessment will also refer to the above criteria, but in this case there is no measurable disease. Worsening in non-target disease cannot be easily assessed (by definition: all non-target lesions must be truly non-measurable), so when the increase in overall disease burden due to the change in non-target disease is comparable in magnitude to PD in target disease, a valid test is needed to assess unequivocal progression in non-target disease based on the definition of unequivocal progression. This is described as an

increase in tumor burden corresponding to an additional 73% increase in volume (corresponding to a 20% increase in diameter of a measurable lesion). Another example is peritoneal exudation from "trace" to "large"; Lymphangiopathy from "local" to "widespread"; Or described in the protocol as "sufficient to change treatment". Examples include pleural effusion ranging from trace to large, lymphatic involvement spreading from the primary site to distant sites, or may be described in protocols as "warranting a change in therapy". If unequivocal progression is observed, the subject should be considered as having progressed overall at that time point. It is preferable to have objective criteria applicable to the assessment of non-measurable disease, note that the added criteria must be reliable.

2.3. 5 New lesions

The appearance of new malignant lesions predicts disease progression; Therefore, some evaluation of new lesions is very important. There are no specific criteria for radiographic detection of lesions, however the finding of a new lesion should be unequivocal. For example, progression cannot be attributed to differences in imaging techniques, changes in imaging modality, or lesions other than tumor (e.g., some so-called new bone lesions are simply healing of the original lesion, or recurrence of the original lesion). This is important when a patient has a partial or complete response of a baseline lesion. For example, necrosis of a liver lesion may qualify as a new cystic lesion on CT report when it is not.

Lesions detected at follow-up but not at baseline will be considered new lesions and indicate disease progression. For example, a subject with visceral disease at baseline who has metastases on CT or MRI will be considered as evidence of progressive disease, even if he does not have a cranial examination at baseline.

If a new lesion is equivocal, for example due to its small size, further treatment and follow-up evaluation are required to confirm whether it is a new lesion. If repeat testing confirms that it is a new lesion, the time to progression should be counted from the time it was first identified.

FDG-PET assessment of disease generally requires additional testing to complement this, and it is reasonable to combine FDG-PET with CT to assess progression (especially for new suspected disease). New lesions can be identified by FDG-PET using the following procedures:

A negative FDG-PET at baseline followed by a positive FDG-PET at follow-up indicates disease progression.

No baseline FDG-PET and positive follow-up FDG-PET:

If the positive FDG-PET at follow-up identifies a new lesion consistent with the CT scan, this is disease progression.

If a positive FDG-PET at follow-up is not confirmed by CT as a new lesion, additional CT should be performed to confirm the lesion (if confirmed, the time of progression begins with the initial abnormal FDG-PET).

If a positive FDG-PET at follow-up is consistent with a pre-existing lesion on CT that does not progress radiographically, there is no progression.

2.4 Best Overall Response Evaluation

The best overall response assessment is the best response recorded from the start of the trial to the end of the trial, taking into account any necessary conditions for confirmation. Sometimes response occurs after end of treatment, so protocols should specify whether post-treatment response assessments should be considered in the best overall response assessment. Protocols must clarify how any new treatment prior to progression affects the best response. A subject's best response is primarily dependent on the findings of target and non-target lesions and the appearance of new lesions. In addition, it depends on the nature of the trial, protocol requirements, and outcome measures. Specifically, in non-randomized trials where response is the primary objective, confirmation of PR or CR is required to determine which is the best overall response assessment.

2.4. 1 Time point response

It is assumed that there will be efficacy evaluations at specific time points in each protocol. Attached Table 2 provides a summary of the overall response evaluation at each time point for the population of subjects with measurable disease at baseline.

If the subject has no measurable disease (no target lesions), the assessment can be found in Table 3.

2.4. 2 Missing and Not Evaluable Assessments Clarification

If a lesion cannot be imaged or measured at a particular time point, the subject is not evaluable at that time point. If only a subset of lesions can be evaluated at an assessment, this is generally considered not evaluable at that timepoint unless there is evidence that the missing lesions do not affect the response assessment at the given timepoint. This is likely to occur in the event of disease progression. For example, a subject who has 3 lesions at baseline with a sum of 50 mm and then only 2 lesions are evaluable with a sum of 80 mm will be assessed as having progressive disease, regardless of the contribution of the missing lesions.

2.4. 3 Best Overall Response: All Time Points

Once all data are available for a subject, the best overall response can be determined.

Assessment of best overall response when confirmation of complete or partial response is not required in the study: The best response in the trial is the best response at

all time points (e.g., a subject who has SD at Cycle 1, PR at Cycle 2, and PD at the last cycle but has a best overall response of PR). When the best overall response is SD, it must meet the protocol-specified minimum time from baseline. If the minimum time criterion is not met, even the best overall response assessment of SD is not acceptable, the subject's best overall response will depend on subsequent assessments. For example, a subject who has SD at Cycle 1 and PD at Cycle 2, but who does not meet the minimum time for SD, has a best overall response of PD. The same subject lost to follow-up after an SD assessment at Cycle 1 will be considered not evaluable.

Evaluation of best overall response when confirmation of complete or partial response is required in the study: Complete or partial response can only be declared when each subject meets the trial-specified criteria for partial or complete response and is confirmed again at a later time point (generally four weeks later) as specified in the protocol. In this case, the best overall response is described in Table 4.

2.4. 4 Special Notes for Efficacy Assessment

When nodal lesions are included in the overall target lesion assessment and the nodes decrease in size to "normal" size (< 10 mm), they will still have a lesion size scan report. In order to avoid overestimating what is reflected by an increase in nodal size, measurements will be recorded even if the node is normal. As already mentioned, this means that subjects with complete response will not be recorded as 0 on the eCRF.

If confirmation of response is required during the course of the trial, repeated "non-measurable" time points will complicate the best response assessment. The analysis plan for the trial must specify that these missing data/assessments can be accounted for when determining efficacy. For example, in most trials, a subject's response of PR-NE-PR can be considered a confirmed response.

Symptomatic progression should be reported when a subject experiences a global deterioration in his/her health condition requiring discontinuation of treatment without objective evidence. Every effort should be made to assess objective progression even after treatment discontinuation. Symptomatic deterioration is not an evaluable description of an objective response: it is a reason for discontinuation of treatment. The objective response of such subjects will be evaluated by target and non-target lesions as shown in Table 2 to Table 4.

Conditions defined as early progression, early death, and non-evaluability are study specific and should be clearly described in each protocol (depending on treatment interval and treatment cycle).

In some cases, it is difficult to distinguish a local lesion from normal tissue. When the assessment of complete response is based on this definition, it is recommended that a biopsy be performed before a response assessment of complete response of local lesions

is made. FDG-PET is used as a similar assessment to biopsy for response confirmation of complete response when some subjects have abnormal radiographic findings in local lesions that are considered to represent fibrosis or scarring of the lesion. In such cases, the use of FDG-PET should be prospectively described in the protocol, supported by reports in the specialized medical literature for this condition. However, it is important to recognize that the limitations of FDG-PET and biopsy, including their resolution and sensitivity, may lead to false-positive results in the assessment of complete response.

Attached Table 2. Timepoint Efficacy: Subjects with Target Lesions (with or without Non-Target Lesions)

Target Lesions	Non-Target Lesions	New Lesions	Overall response
CR	CR	Non-	CR
CR	Non-CR/Non-PD	Non-	PR
CR	Not evaluable	Non-	PR
PR	Non-progressive or not fully evaluable	Non-	PR
SD	Non-progressive or not fully evaluable	Non-	SD
Not fully assessed	Non-Progressive	Non-	NE
PD	Any condition	Yes or No	PD
Any condition	PD	Yes or No	PD
Any condition	Any condition	Yes	PD
CR = complete response	PR = partial response	SD = stable disease	PD = progressive disease NE = not evaluable

Attached Table 3. Timepoint Response-Subjects with Non-Target Lesions Only

Non-Target Lesions	New Lesions	Overall response
CR	Non-	CR
Non-CR or Non-PD	Non-	Non-CR or Non-PD
Not fully assessed	Non-	Not evaluable
Indeterminate PD	Yes or No	PD
Any condition	Yes	PD

Note: For non-target lesions, "non-CR/non-PD" is defined as a response superior to SD. As SD is increasingly used as an endpoint to evaluate response, a non-CR/non-PD response was developed to address the non-specified absence of measurable disease.

For equivocal findings of progression (eg, very small and uncertain new lesions; cystic or necrotic lesions in pre-existing lesions), treatment may continue until the next assessment. If disease progression is confirmed at the next assessment, the date of progression should be the previous date of suspected progression.

Attached Table 4. Confirmed best overall response required for response of CR and PR

First Time Point Overall Response	Overall Response at Subsequent Timepoints	Best overall response
CR	CR	CR
CR	PR	SD, PD or PRa
CR	SD	SD if SD lasts for sufficient time, otherwise PD
CR	PD	SD if SD lasts for sufficient time, otherwise PD
CR	NE	SD if SD lasts sufficient time, otherwise NE
PR	CR	PR
PR	PR	PR
PR	SD	SD
PR	PD	SD if SD lasts for sufficient time, otherwise PD
PR	NE	SD if SD lasts sufficient time, otherwise NE
NE	NE	NE

Note: CR is complete response, PR is partial response, SD is stable disease, PD is progressive disease, and NE is not evaluable. Superscript "a": If there is a true CR at the first timepoint, any disease at a subsequent timepoint will remain PD at a subsequent timepoint even if the subject meets PR criteria relative to baseline (since disease will reappear after CR). Best response is determined by the occurrence of SD within the shortest treatment interval. However, sometimes the first assessment is CR, but subsequent time point scans suggest that small lesions still appear to be present, so that the subject's response should actually be PR rather than CR at the first time point. In this case, the initial CR determination should be revised to PR and the best response is PR.

2.5. Frequency of tumor re-evaluation

The frequency of tumor re-evaluation during treatment depends on the treatment regimen and should be consistent with the type and schedule of treatment. In phase III trials, follow-up every 6 to 9 weeks (timed at the end of a cycle) is justified, and the length of the interval may be adjusted in specific protocols or circumstances. The protocol should specify which tissue sites need to be assessed at baseline (usually those most likely to be associated with metastatic disease in the tumor type under study) and how often evaluations should be repeated. Normally, both target and non-target lesions should be evaluated at each assessment. In some optional situations, certain non-target lesions may be evaluated less frequently, e.g., bone scans may be repeated only if response assessment

in target disease is confirmed as CR or if progression in bone is suspected.

After the end of treatment, tumor re-evaluation depends on whether response rate or time to occurrence of an event (progression/death) is the clinical trial endpoint. In case of an event (e.g., TTP/DFS1/PFS), routine repeat evaluation as specified in the protocol is required. In particular, in randomised comparative trials, scheduled assessments should be listed in the schedule (e.g., 6 to 8 weeks on treatment, or 3 to 4 months after treatment) and should not be affected by other factors such as treatment delays, dosing intervals, and any other events that may lead to imbalances in the treatment arm in the timing of disease assessments.

2.6. Response Assessment/Confirmation of Response Duration

2.6. 1. Confirmation

For non-randomized clinical studies where efficacy is the primary endpoint, confirmation of response of PR and CR is required to ensure that the response is not the result of evaluation error. This also allows for a reasonable interpretation of the results where historical data are available, but efficacy should also be confirmed in historical data from these trials. However, in all other cases, such as randomised trials (Phase II or III) or studies with stable disease or progressive disease as the primary endpoint, confirmation of response is no longer required because it is not valuable for the interpretation of trial results. However, removal of the requirement for response confirmation makes central review to prevent bias effects more important, especially in unblinded trials.

In the case of SD, at least one measurement met the SD criteria specified in the protocol at the shortest interval after the start of the trial (generally not less than 6 to 8 weeks).

2.6. 2 Overall Response

The duration of overall response is measured from the time the measurement first meets the criteria for CR or PR (whichever is first measured) to the first true documentation of recurrent or progressive disease (taking the smallest measurement recorded in the trial as a reference for progressive disease). The time to overall complete response is the time from the time the measurement first meets the criteria for CR to the first true documentation of disease recurrence or progression.

2.6. 3. Stable disease

Is the time from the start of treatment to disease progression (in randomised trials, from the time of randomisation), taking as reference the smallest sum in the trial (if the baseline sum is the smallest, it is used as reference for the calculation of PD). The clinical relevance of stable disease varies from study to study and from disease to disease. If the proportion of patients who remain stable for a minimum period of time is used as an

endpoint in a particular trial, the protocol should specify the minimum time interval between two measurements in the definition of SD.

Note: The duration of response, stable disease, and PFS are influenced by the frequency of follow-up after baseline evaluation. Defining a standard follow-up frequency is outside the scope of this guideline. The frequency of follow-up should take into account many factors, such as type and stage of disease, duration of treatment, and standard practices. However, limitations in the accuracy of these measured endpoints should be taken into account if comparisons between trials are necessary.

2.7. PFS/PPF

2.7. 1. Phase II clinical trial

This guideline focuses on the use of objective response as an endpoint in phase II clinical trials. In some cases, response rate may not be optimal for evaluating the potential anticancer activity of a new drug/regimen. In these cases, PFS/PPF at the cut-off time point can be considered a suitable surrogate to provide an original signal of biological activity of the new drug. It is clear, however, that in an uncontrolled trial, these evaluations may be questioned because seemingly valuable observations may be related to biological factors such as patient selection rather than the effects of pharmacological interventions. Therefore, phase II trials with these endpoints are best designed as randomized controls. However, the clinical presentation of certain tumors is consistent (usually consistently poor), and non-randomized trials are justified. However, in these cases, due to the lack of an active control, evidence of efficacy should be carefully documented when assessing the expected PFS or PPF.

Appendix 4: List of Causality Judgment between Adverse Events and Study Drug

<table>
<tr><td>Relationship to Sponsor Product</td><td colspan="2">Was the study drug responsible for the adverse event? The medically qualified investigator is required to provide a causal assessment between the study drug and the adverse event. The investigator will sign/date (initials) the source document or worksheet to support the causality assessment on the AE form to ensure a medically qualified causality assessment. This signed document must be retained for the required regulatory timeframe. The following criteria are intended to serve as a reference guide to assist the investigator in assessing the relationship between the investigational product and the occurrence of an adverse event based on the available information.
The following elements were used to assess the relationship between the study drug and the AE; The greater the correlation (in terms of number and/or intensity) between the items and their corresponding elements, the greater the likelihood that the study drug will cause an adverse event;</td></tr>
<tr><td rowspan="4"></td><td>Exposure</td><td>Is there evidence that the subject is indeed exposed to the trial drug, e.g., a true and credible past medical history, acceptable compliance assessments (drug counts, logs, etc.), expected pharmacological effects, measurement of drug/metabolites in in vivo collected specimens?</td></tr>
<tr><td>Time course</td><td>Is there a reasonable temporal sequence between the adverse event and treatment with the study drug?
Did the adverse event occur at a time consistent with a drug-induced adverse event?</td></tr>
<tr><td>Reason other than study drug</td><td>Is there an alternative etiology for the adverse event, such as underlying disease, other drugs/vaccines, or other host or environmental factors</td></tr>
<tr><td>Dechallenge</td><td>Was study drug discontinued or dose/exposure/frequency reduced?
If yes, did the AE resolve or improve?
If yes, a positive dechallenge is indicated. If not, a negative dechallenge is indicated.
Note: This criterion does not apply if: (1) an adverse event results in death or permanent disability; (2) AE recovered/improved despite continued use of study drug; (3) The trial was a single-dose trial of the drug; (4) Only one dose of the study drug.</td></tr>
</table>

	**Rechallenge**	Has the subject been repeatedly exposed to the study drug in this trial?<br>If yes, did the AE resolve or improve? ?<br>If yes, a positive rechallenge. If not, the rechallenge test was negative.<br>Note: This criterion does not apply if: (1) the initial AE resulted in death or permanent disability, (2) the trial was a single-dose clinical trial, and (3) only one dose of study drug was administered.<br>Note: If a rechallenge is planned for an adverse event that is serious and possibly attributable to the investigational product, or if reexposure to the investigational product may pose a serious potential risk to the subject/patient, rechallenge is not recommended, unless it is considered that continuation of the investigational product may be beneficial to the patient and no alternative treatment is available, and may be conducted after prior approval by the sponsor.
	**Consistency with trial treatment characteristics**	Is the clinical/pathological presentation of the adverse event consistent with previous data on the investigational drug or pharmacology and toxicology studies of such drugs?
The medically qualified investigator will record the causality assessment based on his/her best clinical judgment, including consideration of the above causality factors. The causality assessment of the adverse event to the study drug will be recorded as "related" and "not related".		
**Record Causality**		**The table below can be used for causality assessment (not all criteria need to be met)**
**Related**		There was generally evidence of exposure to study drug. An AE with a reasonable temporal sequence from administration of the sponsor product. The occurrence of an AE is more likely to be explained by the study drug than by other causes.
**Not related**		Generally, it means that the subject does not take the study drug, the time between the occurrence of adverse event and the occurrence of adverse event is unreasonable or there are other reasons that can better explain the adverse event, but not the study drug.

Appendix 5: List of Prohibited Medications for Concomitant Therapy

Interaction mechanism	Drug Name
CYP3A and P-gp Strong Inducers	Carbamazepine, phenytoin, rifampicin, St. John's wort, enzalutamide, apalutamide, mitotane
CYP3A and P-gp Strong Inhibitors	Boceprevir, clarithromycin, cobicistat, grapefruit juice (> 1L/day, High concentration), indinavir, itraconazole, ketoconazole, lopinavir and ritonavir, nefazodone, nelfinavir, posaconazole, quinupristin, ritonavir, saquinavir, telaprevir, telithromycin, voriconazole, amiodarone, carvedilol, diltiazem, dronedarone, glacaprevir, lamitentan, ledipasvir, letermovir, palirevir, peramrevir, quinidine, ranolazine, simeprevir, ticagrelor, velpatasvir, verapamil, vociclosporin, vorcivir, danovirvir, elvitegravir, obitasvir and/or dasabuvir, tipranavir, troleandomycin, ivalaprevir, telaprevir and/or Patinib, prapafenone
CYP2D6 Sensitive Substrates	Dextromethorphan, tolterodine, metoprolol, desipramine, amphetamine, aripiprazole, atomoxetine, desipramine, iloperidone, nebivolol, perphenazine, risperidone, timolol, tolterodine, venlafaxine, vortioxetine, thioridazine, eliglustat, nortriptyline, encainide, imipramine, propranolol, tramadol, trimipramine, venlafaxine
Sensitive CYP3A Substrates	Levonorgestrel (LNG) and ulipristal acetate (UPA), ivacaftor, abecinib, midazolam, leratinib, felodipine, midazolam, buspirone, sildenafil, simvastatin, budesonide, fluticasone, nifedipine, tolvaptan, isavuconazole, ivanib, apalutamide, cobictinib, temsirolimus, idelaris, triazolam, eplerenone, alprazolam, almotriptan, apremilast, nisoldipine, avanafil, roflumilast, bromocriptine mesylate, eszopiclone, flibanserin, amlodipine, isradipine, alprazolam, aprepitant, atorvastatin, colchicine, Rilpivirine, rivaroxaban, tadalafil, pimozide, alfentanil, conivaptan, darifenacin, darunavir, ebastine, everolimus, ibrutinib, lometapide, lovastatin, naloxol, nisoldipine, sirolimus, tacrolimus, endenafil, dasatinib, eletriptan, lurasidone, maraviroc, guetiapine, ticagrelor, linagliptin, pethidine, tamoxifen

Listed in the table are common strong inhibitors and inducers of CYP3A and P-gp, and sensitive substrates of CYP2D6 and CYP3A4. In case of doubt about other possible concomitant medications, the investigator may communicate with the sponsor.

Appendix 6: EQ-5D-5L Life Scale

Please indicate which of the following options best reflects your health today and tick the box (√).

Move around

I have no difficulty moving around.

-

I have a little difficulty moving around.

-

I have moderate difficulty moving around.

-

I have severe difficulty moving around.

-

I can't walk around.

-

Self-care

I have no difficulty caring for myself.

-

I have a little difficulty washing my face, brushing my teeth, bathing, or dressing myself.

-

I have moderate difficulty washing my face, brushing my teeth, bathing, or dressing myself.

-

I have serious trouble washing my face, brushing my teeth, bathing, or dressing myself.

-

I cannot wash my face, brush my teeth, bathe or dress myself.

-

Usual activities (such as work, study, housework, home or leisure activities)

I have no difficulty doing my usual activities.

-

I have a little difficulty doing my usual activities.

-

I have moderate difficulty doing my usual activities.

-

I have severe trouble doing my usual activities.

-

I am not able to carry out my usual activities.

-

Pain/discomfort

I don't have any pain or discomfort.

-

I feel a little pain or discomfort.

-

I feel moderate pain or discomfort.

-

I feel severe pain or discomfort.

-

I feel very severe pain or discomfort.

-

Anxiety/depression

I don't have any anxiety or depression.

-

I feel a little anxious or depressed.

-

I feel moderately anxious or depressed.

-

I feel severely anxious or depressed.

-

I feel very anxious or depressed.

-

为了帮助您反映健康状况的好坏，我们画了一个刻度尺(有点象温度计)，在这刻度尺上，100 代表您心目中最好的状况，0 代表您心目中最差的状况。

请在右边的刻度尺上标出您今天的健康状况。请从下面方格中画出一条线，连到刻度尺上最能代表您今天健康状况好坏的那一点。

**您今天的
健康状况**

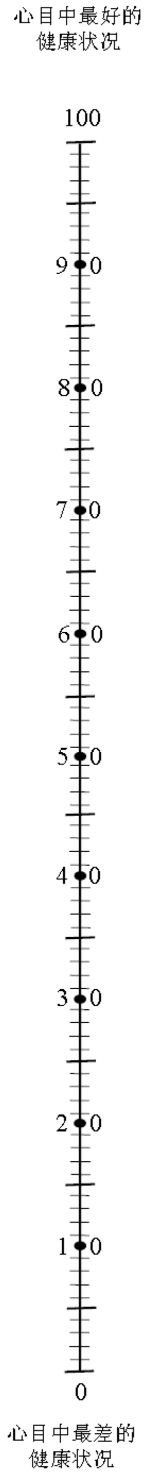

Appendix 7: EORTC QLQ-C30 (V3) Life Scale

We want to know something about you and your health. Please answer all of the following questions personally. There are no "right" or "wrong" answers, only the number that best reflects your situation is circled. The information you provide will be kept strictly confidential.

Please fill in your initials: _____________

Date of Birth: DD MMM YYYY

Today's date: dd mmm yyyy

	None	A	Comparable	Very
1. Do you have trouble doing some strenuous activities,	1	2	3	4
2. Is it difficult for you to walk long distances?	1	2	3	4
3. Is it difficult for you to walk short distances	1	2	3	4
4. Do you need to stay in bed or chair during the	1	2	3	4
5. Do you need help with eating, dressing, bathing,	1	2	3	4

Within the past week:	None	A little	Comparable	Very
6. Are you limited in your work and daily	1	2	3	4
7. Are you restricted in your hobbies or leisure	1	2	3	4
8. Are you short of breath?	1	2	3	4
9. Do you have pain?	1	2	3	4
10. Do you need a break?	1	2	3	4
11. Do you have trouble sleeping?	1	2	3	4
12. Are you feeling weak?	1	2	3	4
13. Do you have loss of appetite (lack of	1	2	3	4
14. Do you feel nauseous?	1	2	3	4
15. Do you vomit?	1	2	3	4
16. Do you have constipation?	1	2	3	4

**Within the past week:**	**None**	**A little**	**Comparable**	**Very**
17. Do you have diarrhea?	1	2	3	4
18. Are you feeling tired?	1	2	3	4
19. Does the pain interfere with your daily	1	2	3	4
20. Do you have trouble concentrating on things,	1	2	3	4
21. Are you feeling nervous?	1	2	3	4
22. Are you worried?	1	2	3	4
23. Do you feel irritable?	1	2	3	4
24. Do you feel depressed (depressed)?	1	2	3	4
25. Do you have trouble remembering?	1	2	3	4
26. Does your medical condition or treatment	1	2	3	4
27. Does your medical condition or treatment	1	2	3	4
28. Has your medical condition or treatment put	1	2	3	4

For the following questions, please circle the number between 1 and 7 that best fits you.

29. How do you rate your overall health during the past week?

1 2 3 4 5 6 7

Very Poor Very Good

30. How would you rate your overall quality of life over the past week?

1 2 3 4 5 6 7

Very Poor Very Good
